# Bemarituzumab as first-line treatment for locally advanced or metastatic gastric/gastroesophageal junction adenocarcinoma: final analysis of the randomized phase 2 FIGHT trial

**DOI:** 10.1007/s10120-024-01466-w

**Published:** 2024-02-03

**Authors:** Zev A. Wainberg, Yoon-Koo Kang, Keun-Wook Lee, Shukui Qin, Kensei Yamaguchi, In-Ho Kim, Anwaar Saeed, Sang Cheul Oh, Jin Li, Haci Mehmet Turk, Alexandra Teixeira, Erika Hitre, Adrian A. Udrea, Giovanni Gerardo Cardellino, Raquel Guardeño Sanchez, Anita Zahlten-Kümeli, Kate Taylor, Peter C. Enzinger

**Affiliations:** 1 Division of Hematology-Oncology, Department of Medicine, University of California Los Angeles Medical Center, David Geffen School of Medicine, 2825 Santa Monica Blvd., Suite 200, Santa Monica, Los Angeles, CA 90404-2429 USA; 2 Asan Medical Centre, University of Ulsan College of Medicine, Seoul, South Korea; 3 Seoul National University College of Medicine, Seoul National University Bundang Hospital, Seongnam, South Korea; 4 Nanjing Tianyinshan Hospital, The 1st Affiliated Hospital of China Pharmaceutical University, Nanjing, China; 5 Gastroenterological Chemotherapy Department, The Cancer Institute Hospital of JFCR, Tokyo, Japan; 6 Department of Oncology, The Catholic University of Korea, Seoul St Mary’s Hospital, Seoul, South Korea; 7 UPMC Hillman Cancer Center, University of Pittsburgh, Pittsburgh, PA USA; 8 Department of Internal Medicine, Korea University Guro Hospital, Seoul, South Korea; 9 Department of Oncology, Shanghai East Hospital, Tongji University School of Medicine, Shanghai, China; 10 Department of Medical Oncology, Bezmialem Vakif University, Istanbul, Turkey; 11 Gastroenterology Division, Hospital da Senhora da Oliveira, Guimarães, Portugal; 12 Department of Medical Oncology and Clinical Pharmacology “B”, National Institute of Oncology, Budapest, Hungary; 13 Medical Oncology, Medisprof Cancer Center, Cluj-Napoca, Romania; 14 Department of Oncology, Azienda Sanitaria Universitaria Friuli Centrale, Udine, Italy; 15 Department of Medical Oncology, Catalan Institute of Oncology, Girona, Spain; 16 Amgen Inc, Thousand Oaks, CA USA; 17 Amgen Inc, Uxbridge, UK; 18 Department of Medicine, Dana-Farber Cancer Institute, Boston, MA USA

**Keywords:** Bemarituzumab, FGFR2b, Gastric cancer, mFOLFOX6, Targeted therapy

## Abstract

**Background:**

We report the final results of the randomized phase 2 FIGHT trial that evaluated bemarituzumab, a humanized monoclonal antibody selective for fibroblast growth factor receptor 2b (FGFR2b), plus mFOLFOX6 in patients with FGFR2b-positive (2 + /3 + membranous staining by immunohistochemistry), HER-2–negative gastric or gastroesophageal junction cancer (GC).

**Methods:**

Patients received bemarituzumab (15 mg/kg) or placebo once every 2 weeks with an additional bemarituzumab (7.5 mg/kg) or placebo dose on cycle 1 day 8. All patients received mFOLFOX6. The primary endpoint was investigator-assessed progression-free survival (PFS). Secondary endpoints included overall survival (OS), objective response rate, and safety. Efficacy was evaluated after a minimum follow-up of 24 months.

**Results:**

In the bemarituzumab-mFOLFOX6 (N = 77) and placebo-mFOLFOX6 (N = 78) arms, respectively, 59.7% and 66.7% of patients were FGFR2b-positive in ≥ 10% of tumor cells. The median PFS (95% confidence interval [CI]) was 9.5 months (7.3–13.7) with bemarituzumab-mFOLFOX6 and 7.4 months (5.7–8.4) with placebo-mFOLFOX6 (hazard ratio [HR], 0.72; 95% CI 0.49–1.08); median OS (95% CI) was 19.2 (13.6–24.2) and 13.5 (9.3–15.9) months, respectively (HR 0.77; 95% CI 0.52–1.14). Observed efficacy in FGFR2b-positive GC in ≥ 10% of tumor cells was: PFS: HR 0.43 (95% CI 0.26–0.73); OS: HR 0.52 (95% CI 0.31–0.85). No new safety findings were reported.

**Conclusions:**

In FGFR2b-positive advanced GC, the combination of bemarituzumab-mFOLFOX6 led to numerically longer median PFS and OS compared with mFOLFOX6 alone. Efficacy was more pronounced with FGFR2b overexpression in ≥ 10% of tumor cells. Confirmatory phase 3 trials are ongoing (NCT05052801, NCT05111626).

**Clinical trial registration:**

NCT03694522.

**Supplementary Information:**

The online version contains supplementary material available at 10.1007/s10120-024-01466-w.

## Introduction

Gastric cancer (GC), including gastroesophageal junction (GEJ) cancer, is often diagnosed at an advanced stage and is associated with poor prognosis [1]. Systemic chemotherapy has been the standard first-line treatment for advanced GC, although the clinical benefits have been limited [2, 3]. Recent therapeutic approaches including immune checkpoint inhibitors or targeted therapies that are directed towards different mechanistic pathways of GC have demonstrated promising outcomes, especially in biomarker-enriched patient populations [4, 5]. Human epidermal growth factor receptor 2 (HER-2)–directed therapies such as trastuzumab and trastuzumab deruxtecan have improved overall survival (OS) outcomes in HER-2–positive GC [6, 7]. However, most targeted therapies, such as bevacizumab, everolimus, or panitumumab and cetuximab, have not demonstrated OS benefits in unselected patients with GC, which has been attributed, in part, to intratumoral heterogeneity or a lack of selective biomarkers [4, 5]. Treatment decisions are therefore best guided by biomarker expression and histological classifications to preselect patients most likely to benefit from targeted therapies and adapt treatments to help improve outcomes for GC. New and effective biomarker-targeted treatment options remain an unmet clinical need for advanced GC.

The fibroblast growth factor/FGF receptor (FGF/FGFR) pathway plays a crucial role in the growth and development of cancer cells, and overexpression of proteins in this pathway could lead to disease progression [8]. The IIIb splice isoform of FGFR2 (FGFR2b) was observed to be overexpressed in approximately 30% of HER-2–negative GC [9–12]. FGFR2b overexpression in GC may be associated with poorly differentiated diffuse-type histology and poor outcomes, including lower OS, and warrants further investigation [10, 11, 13].

Bemarituzumab is a first-in-class, humanized monoclonal antibody specific to human FGFR2b that blocks FGF binding to the receptor. Bemarituzumab acts through a two-pronged approach [14]. First, it selectively inhibits FGFR2b signaling with downstream effects on cancer cell proliferation [14, 15]. Second, the afucosylated structure of bemarituzumab leads to activation of FcγRIIIa/CD16a, which increases the affinity of bemarituzumab for natural killer cells, thereby enhancing its antibody-dependent cellular cytotoxicity against FGFR2b-expressing tumor cells [14, 16].

The first-in-human (FIH) study of bemarituzumab monotherapy showed that it was well tolerated and demonstrated activity as later-line therapy in patients with advanced GC, with a confirmed objective response rate (ORR) of 17.9% (95% confidence interval [CI], 6.1–36.9) in 28 patients with FGFR2b-positive tumors and a median duration of response of 12.6 weeks (range, 9.1–19.1) [17]. The phase 1/2 FIGHT trial (NCT03694522) evaluated the efficacy and safety of bemarituzumab in combination with modified 5-fluorouracil, leucovorin, and oxaliplatin (mFOLFOX6) chemotherapy as first-line treatment for HER-2 non-positive advanced GC with FGFR2b overexpression and/or *FGFR2* gene amplification [18]. The phase 1 safety run-in was an open-label, dose-escalation study of bemarituzumab-mFOLFOX6 in patients with gastrointestinal tumors. The randomized phase 2 study further assessed the efficacy and safety of the combination versus placebo-mFOLFOX6 in HER-2 non-positive FGFR2-selected advanced GC [9]. The primary analysis (median follow-up, 10.9 months) showed that the median progression-free survival (PFS) was 9.5 months (95% CI 7.3–12.9) with bemarituzumab-mFOLFOX6 versus 7.4 months (95% CI 5.8–8.4) with placebo-mFOLFOX6 (hazard ratio [HR], 0.68; 95% CI 0.44–1.04; *P* = 0.07) [9]. Here, we report the final analysis and updated safety results after a minimum follow-up of 24 months.

## Methods

### Study design

The study design and protocol have been described previously [9]. Briefly, the phase 2 portion was a randomized, double-blind, placebo-controlled study conducted at 164 sites across 18 countries and was designed to evaluate the efficacy and safety of bemarituzumab-mFOLFOX6 in patients with advanced HER-2 non-positive GC prescreened for FGFR2b overexpression (via immunohistochemistry [IHC]) and/or *FGFR2* gene amplification (via circulating tumor DNA [ctDNA] assay). Positive FGFR2b overexpression status by IHC was defined as exhibiting any moderate (2 +) to strong (3 +) membranous staining in more than 0% of tumor cells. Patients were randomized (1:1) to receive either bemarituzumab-mFOLFOX6 or placebo-mFOLFOX6. Treatment continued until disease progression, as determined by Response Evaluation Criteria in Solid Tumors version 1.1 (RECIST v1.1), or unacceptable toxicity. During the long-term follow-up period, patients were contacted every 3 (± 1) months for 24 months after the last patient was enrolled, or until death, loss to follow-up, consent withdrawal, or study termination, whichever occurred first. Data cutoff for this final analysis was May 13, 2022.

All patients provided written and informed consent; study protocols (online only) received institutional approval. Complete procedural details are available in the protocol (online only).

### Patients

Eligible patients were ≥ 18 years and had histologically confirmed GC or GEJ adenocarcinoma; unresectable, locally advanced/metastatic disease not amenable to curative therapy; FGFR2b overexpression as determined by a centrally performed IHC tissue test and/or *FGFR2* gene amplification via a centrally performed ctDNA blood-based assay; measurable or non-measurable, but evaluable disease using RECIST v1.1; an Eastern Cooperative Oncology Group (ECOG) performance status of 0 or 1; adequate organ function; and no prior chemotherapy for metastatic or unresectable disease. Patients were permitted, at the discretion of the investigator, to receive a single dose of mFOLFOX6 while awaiting results of centralized FGFR2 testing. Key exclusion criteria included untreated or symptomatic central nervous system metastases; ≥ grade 2 Common Terminology Criteria for Adverse Events (CTCAE) peripheral sensory neuropathy; corneal abnormalities that may increase the risk of developing a corneal ulcer; and known tumor positivity for HER-2 (as identified by a positive IHC test of 3 + or IHC of 2 + with positive fluorescent in situ hybridization). Complete eligibility criteria are available in the online protocol.

### Treatment and randomization

Bemarituzumab or an equivalent placebo was administered at a dose of 15 mg/kg body weight intravenously every 2 weeks (Q2W); an additional dose of 7.5 mg/kg bemarituzumab was administered on cycle 1 day 8. The standard mFOLFOX6 regimen, comprising oxaliplatin (85 mg/m^2^), leucovorin (400 mg/m^2^), and 5-fluorouracil (400 mg/m^2^ bolus followed by 2400 mg/m^2^ over approximately 48 h), was administered Q2W in both arms. Eligible patients were stratified by geographic region, prior treatment status (de novo versus adjuvant/neoadjuvant), and administration of a single dose of mFOLFOX6 before enrollment (yes or no). A permuted block scheme with a block size of four was used for randomization to ensure an equal sample size and a similar distribution of stratification factors.

### Endpoints and assessments

The primary endpoint was PFS, defined as the time from randomization until disease progression based on investigator assessment (using RECIST v1.1) or death, whichever occurred first. Secondary endpoints included OS, defined as the time from randomization until death from any cause; ORR, defined as the proportion of patients with partial response (PR) or complete response (CR) according to investigator assessment of tumor lesions per RECIST v1.1; and incidence of adverse events (AEs). An exploratory endpoint was duration of response (DOR) limited to patients who were responders to treatment, as determined by the investigator per RECIST v1.1, and defined as the time of first response to progression or death from any cause, whichever occurred first. AEs were coded using Medical Dictionary for Regulatory Activities version 25.0 and were graded using CTCAE version 5.0.

### Statistical analysis

The planned enrollment for phase 1 was up to 21 patients, and up to 155 patients were to be enrolled in phase 2, with a total enrollment of approximately 167 patients in the study. Efficacy was assessed in the intention-to-treat (ITT) population, which included all randomized patients, while safety was assessed in the safety analysis set, which included patients who had received at least one dose of the assigned treatment. The Kaplan–Meier method was used to estimate the median PFS and OS and the associated 95% CIs in each treatment arm. HRs and 95% CIs were calculated using a stratified Cox regression model. Formal hypothesis testing was not performed at this final analysis. In a prespecified exploratory analysis, PFS and OS were assessed for subgroups of patients who were FGFR2b-positive in ≥ 10% of tumor cells with moderate to strong staining intensity (2 + /3 +) as assessed by IHC. Data on AEs are presented descriptively by number of patients and frequency. Statistical analyses are detailed in the protocol (online only).

## Results

### Patients

**Fig. 1:**
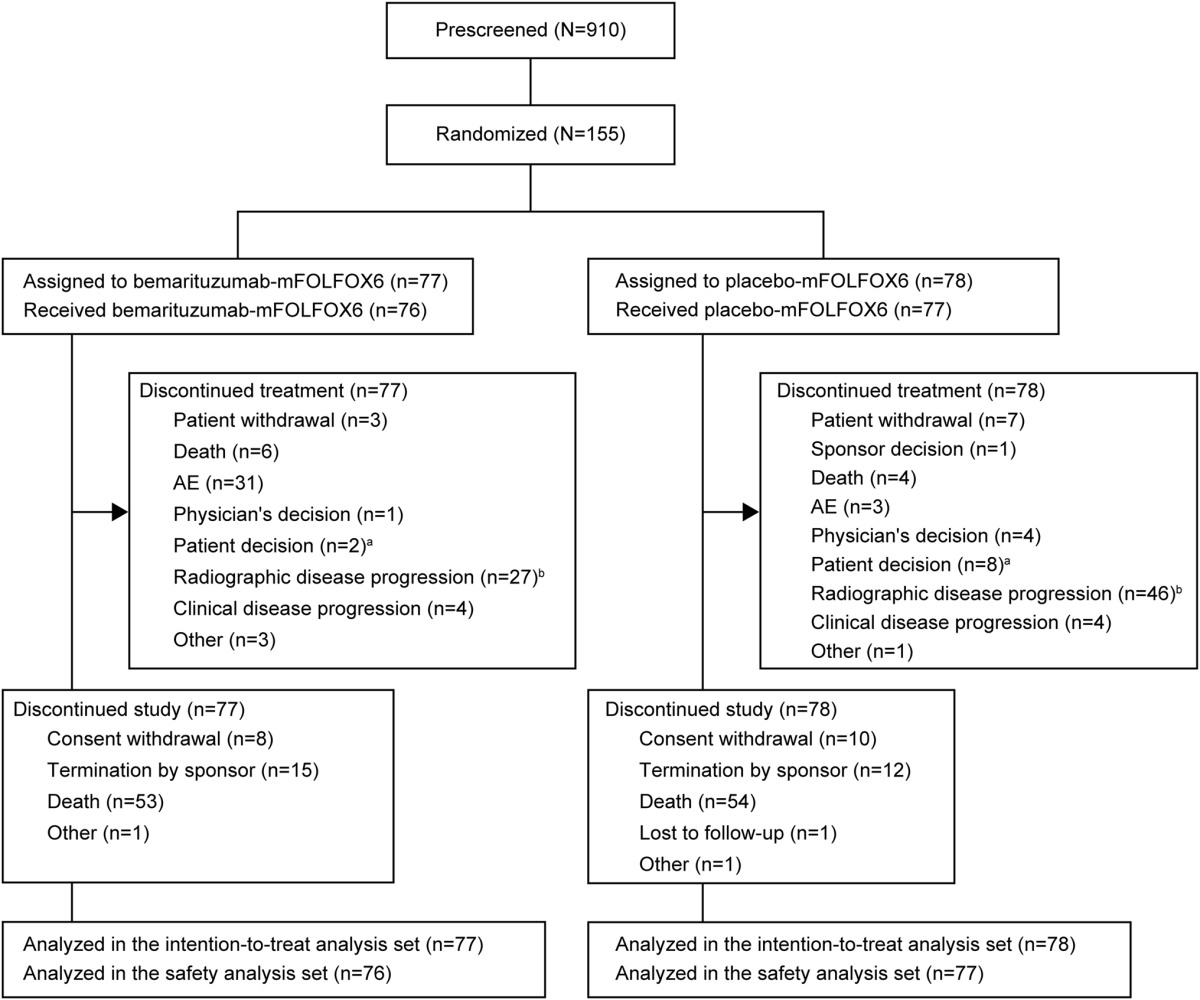
Patient disposition. ^a^Patient chose to discontinue treatment but continued in follow-up. ^b^Radiographic disease progression was assessed as per RECIST version 1.1. AE, adverse event; mFOLFOX6, modified FOLFOX (infusional 5-fluorouracil, leucovorin, and oxaliplatin); RECIST, Response Evaluation Criteria in Solid Tumors

Of the 910 patients prescreened for FGFR2b-positivity, 155 were eligible and were randomized to receive bemarituzumab-mFOLFOX6 (N = 77) or placebo-mFOLFOX6 (N = 78) as part of the ITT analysis set (Fig. 1). The safety analysis set included 153 patients (bemarituzumab-mFOLFOX6, N = 76; placebo-mFOLFOX6, N = 77) who received at least one dose of the study treatment (Fig. 1). At the data cutoff for this final analysis (May 13, 2022), all patients had discontinued the study. The most frequent reasons for treatment discontinuation in the bemarituzumab-mFOLFOX6 arm were AEs (31 [40.3%]) and radiographic disease progression (27 [35.1%]; clinical progression, n = 4), while in the control arm it was radiographic disease progression (46 [59.0%]) (Fig. 1). The primary reason for study discontinuation was death (bemarituzumab-mFOLFOX6, 53 [68.8%]; placebo-mFOLFOX6, 54 [69.2%]), with the most common cause for death being disease progression (bemarituzumab-mFOLFOX6, 39 [50.6%]; placebo-mFOLFOX6, 46 [59.0%]).

Baseline demographics and characteristics were generally balanced between the treatment arms and were reported previously [9]. Of the 155 enrolled patients, 111 (71.6%) were male, and the median (range) age was 60 (23–84) years; 137 (88.4%) had gastric adenocarcinoma and 18 (11.6%) had GEJ adenocarcinoma; 53 (34.2%) and 102 (65.8%) had ECOG performance status of 0 and 1, respectively; 145 (93.5%) had stage IV disease at screening (bemarituzumab-mFOLFOX6, 75 [97.4%]; placebo-mFOLFOX6, 70 [89.7%]); and 71 (45.8%) had received a single dose of mFOLFOX6 before randomization (Table 1). FGFR2b overexpression in ≥ 10% of tumor cells as assessed by IHC (moderate [2 +] to strong [3 +] tumor staining intensity; FGFR2b ≥ 10% subgroup) and regardless of ctDNA gene amplification was found in 46 (59.7%) patients in the bemarituzumab-mFOLFOX6 arm and 52 (66.7%) patients in the placebo-mFOLFOX6 arm. The median duration of exposure was 24.0 weeks (range, 2.0–96.9) in the bemarituzumab-mFOLFOX6 arm and 26.0 weeks (range, 2.0–130.7) in the placebo-mFOLFOX6 arm.

**Table 1:** Demographics and disease characteristics at baseline (ITT analysis set)

Characteristic	Bemarituzumab-mFOLFOX6 (N = 77)	Placebo-mFOLFOX6 (N = 78)
Age		
Median (range), years	60.0 (23–80)	59.5 (33–84)
≥ 65 years	19 (24.7)	25 (32.1)
Male sex	52 (67.5)	59 (75.6)
Race		
Asian	45 (58.4)	44 (56.4)
Black	0	1 (1.3)
American Indian or Alaska Native	0	1 (1.3)
White	30 (39.0)	31 (39.7)
Other	2 (2.6)	1 (1.3)
Site of primary cancer		
Gastric adenocarcinoma	66 (85.7)	71 (91.0)
Gastroesophageal junction adenocarcinoma	11 (14.3)	7 (9.0)
Metastatic disease	73 (94.8)	66 (84.6)
Tumor histology		
Diffuse	28 (36.4)	26 (33.3)
Intestinal	16 (20.8)	15 (19.2)
Mixed	5 (6.5)	12 (15.4)
Unknown	28 (36.4)	25 (32.1)
ECOG performance status		
0	25 (32.5)	28 (35.9)
1	52 (67.5)	50 (64.1)
Administration of a single dose of mFOLFOX6 before randomization	35 (45.5)	36 (46.2)
Prior neoadjuvant or adjuvant therapy	14 (18.2)	13 (16.7)
FGFR2b expression		
Overexpression by IHC regardless of ctDNA (2 + /3 + staining score in any tumor cell)	73 (94.8)	76 (97.4)
IHC staining score of 2 + or 3 + in ≥ 10% of tumor cells regardless of ctDNA	46 (59.7)	52 (66.7)
Amplification by ctDNA regardless of IHC	12 (15.6)	14 (17.9)
Both overexpression by IHC and amplification by ctDNA	8 (10.4)	12 (15.4)

Data are number (%) of patients unless indicated otherwise. The ITT analysis set included all randomized patients

*ctDNA* circulating tumor DNA; *ECOG* Eastern Cooperative Oncology Group; *FGFR2b* IIIb splice isoform of fibroblast growth factor receptor 2; *IHC* immunohistochemistry; *ITT* intention-to-treat; *mFOLFOX6* modified FOLFOX (infusional 5-fluorouracil, leucovorin, and oxaliplatin)

### Progression-free survival

**Fig. 2:**
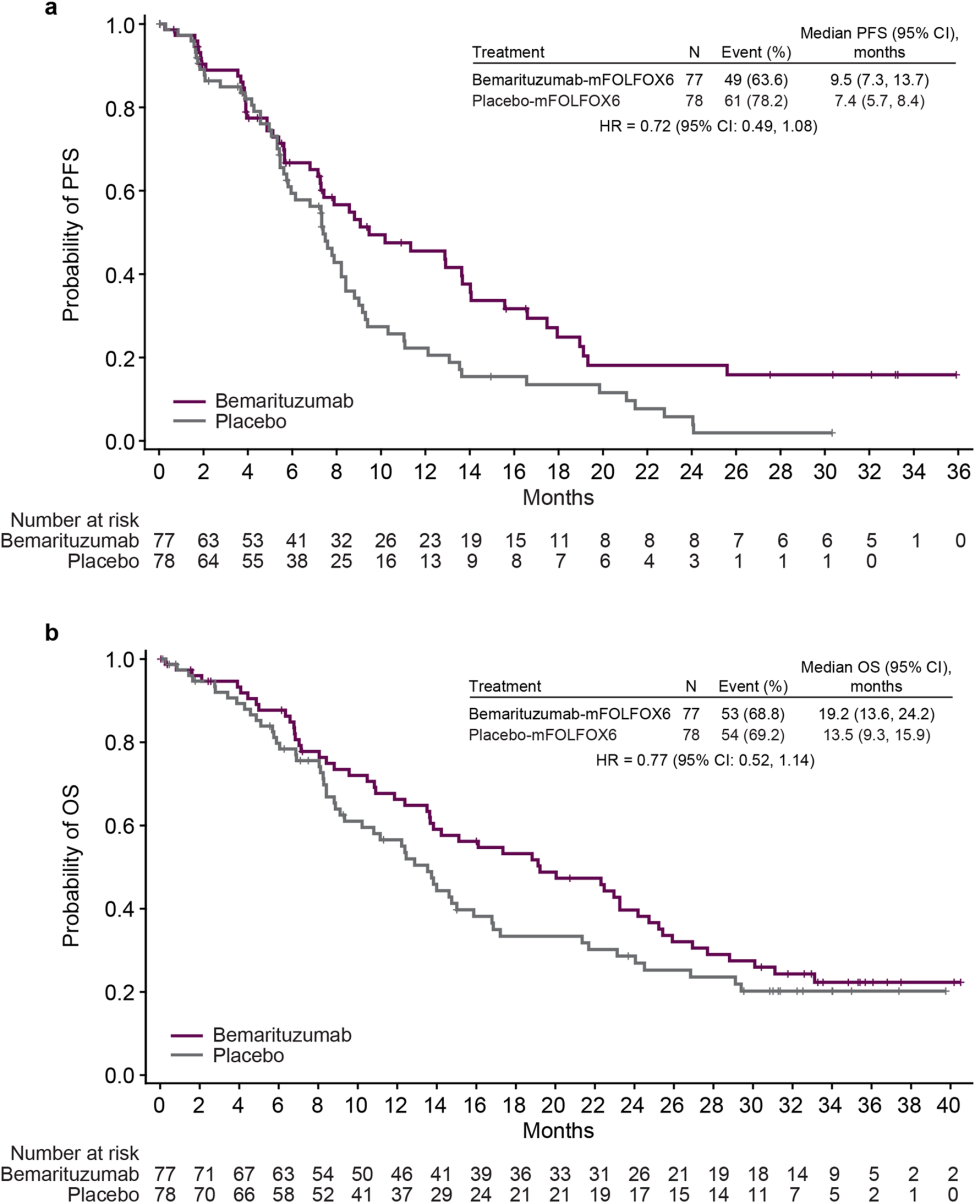
PFS and OS in the ITT population. **a** PFS in the ITT population. **b** OS in the ITT population. The intention-to-treat population included all patients who underwent randomization. HRs and 95% CIs were calculated using the Cox proportional hazards model, adjusted for randomization stratification factors, including administration of mFOLFOX6 single dose prior to randomization and geographical region. Vertical bars indicate censoring. *CI* confidence interval; *HR* hazard ratio; *ITT* intention-to-treat; *mFOLFOX6* modified FOLFOX (infusional 5-fluorouracil, leucovorin, and oxaliplatin); *OS* overall survival; *PFS* progression-free survival

At the data cutoff, the median PFS follow-up time was 6.8 months (range, 0–35.9). PFS events were observed in 49 (63.6%) patients in the bemarituzumab-mFOLFOX6 arm and 61 (78.2%) patients in the placebo-mFOLFOX6 arm. The median PFS was 9.5 months (95% CI 7.3–13.7) in the bemarituzumab-mFOLFOX6 arm and 7.4 months (95% CI 5.7–8.4) in the placebo-mFOLFOX6 arm (HR 0.72; 95% CI 0.49–1.08) (Fig. 2a), with a 12-month estimated PFS rate of 45.5% (95% CI 32.7–57.5) and 22.2% (95% CI 12.8–33.3), respectively.

### Overall survival

At the data cutoff, the median OS follow-up time was 13.5 months (range, 0–40.5). Fifty-three (68.8%) patients in the bemarituzumab-mFOLFOX6 arm and 54 (69.2%) patients in the mFOLFOX6 and placebo-mFOLFOX6 arm died. The median OS was 19.2 months (95% CI 13.6–24.2) with bemarituzumab-mFOLFOX6 and 13.5 months (95% CI 9.3–15.9) with placebo-mFOLFOX6 (HR 0.77; 95% CI 0.52–1.14) (Fig. 2b). The OS landmarks in the bemarituzumab-mFOLFOX6 and placebo-mFOLFOX6 arms, respectively, were 66.3% and 56.5% at 12 months, and 39.7% and 28.6% at 24 months.

### Response rate

Overall, 66 (85.7%) patients in the bemarituzumab-mFOLFOX6 arm and 60 (76.9%) in the placebo-mFOLFOX6 arm had measurable disease at baseline. In the bemarituzumab-mFOLFOX6 arm, the ORR was 48.1% (95% CI 36.5, 59.7), with a CR and PR in 4 (5.2%) and 33 (42.9%) patients, respectively (Table 2). In the placebo-mFOLFOX6 arm, the ORR was 33.3% (95% CI 23.1, 44.9), with a CR and PR in 2 (2.6%) and 24 (30.8%) patients, respectively. The median DOR was 11.9 months (95% CI 6.9, 17.3) with bemarituzumab-mFOLFOX6 (n = 37) and 7.5 months (95% CI 4.3, 13.8) with placebo-mFOLFOX6 (n = 26).

**Table 2:** Tumor response

Variable	ITT set		FGFR2b ≥ 10% subgroup	
	Bemarituzumab-mFOLFOX6 (N = 77)	Placebo-mFOLFOX6 (N = 78)	Bemarituzumab-mFOLFOX6 (N = 46)	Placebo-mFOLFOX6 (N = 52)
Measurable disease at baseline	66 (85.7)	60 (76.9)	41 (89.1)	42 (80.8)
Non-measurable disease at baseline	11 (14.3)	18 (23.1)	5 (10.9)	10 (19.2)
Best overall response				
Complete response	4 (5.2)	2 (2.6)	2 (4.3)	1 (1.9)
Partial response	33 (42.9)	24 (30.8)	24 (52.2)	18 (34.6)
Stable disease	29 (37.7)	38 (48.7)	12 (26.1)	20 (38.5)
Progressive disease	5 (6.5)	6 (7.7)	3 (6.5)	5 (9.6)
Not evaluable	6 (7.8)	8 (10.3)	5 (10.9)	8 (15.4)
Objective response rate (ORR)^a^	37 (48.1)	26 (33.3)	26 (56.5)	19 (36.5)
95% CI^b^	36.5–59.7	23.1–44.9	41.1–71.1	23.6–51.0
Difference in ORR, (95% CI)^b^	14.4 (-1.5–30.3)		20 (0.6–39.4)	
Duration of response^c^				
Number of patients	37	26		
Median (95% CI), months	11.9 (6.9–17.3)	7.5 (4.3–13.8)		

Data are number (%) of patients unless indicated otherwise. The ITT set included all randomized patients. The FGFR2b ≥ 10% subgroup comprised patients with FGFR2b 2 + /3 + IHC staining in ≥ 10% of tumor cells. Overall response is based on RECIST version 1.1

*CI* confidence interval; *CR* complete response; *FGFR2b* IIIb splice isoform of fibroblast growth factor receptor 2; *IHC* immunohistochemistry; ITT, intention-to-treat; *mFOLFOX6* modified FOLFOX (infusional 5-fluorouracil, leucovorin, and oxaliplatin); *PR* partial response; *RECIST* Response Evaluation Criteria in Solid Tumors

^a^ORR is computed as the sum of CR and PR

^b^Two-sided CI based on Clopper-Pearson method

^c^Median duration of response for the FGFR2b ≥ 10% subgroup is not reported because of low patient numbers in each arm for this analysis

### Efficacy for patients in FGFR2b ≥ 10% subgroup

**Fig. 3:**
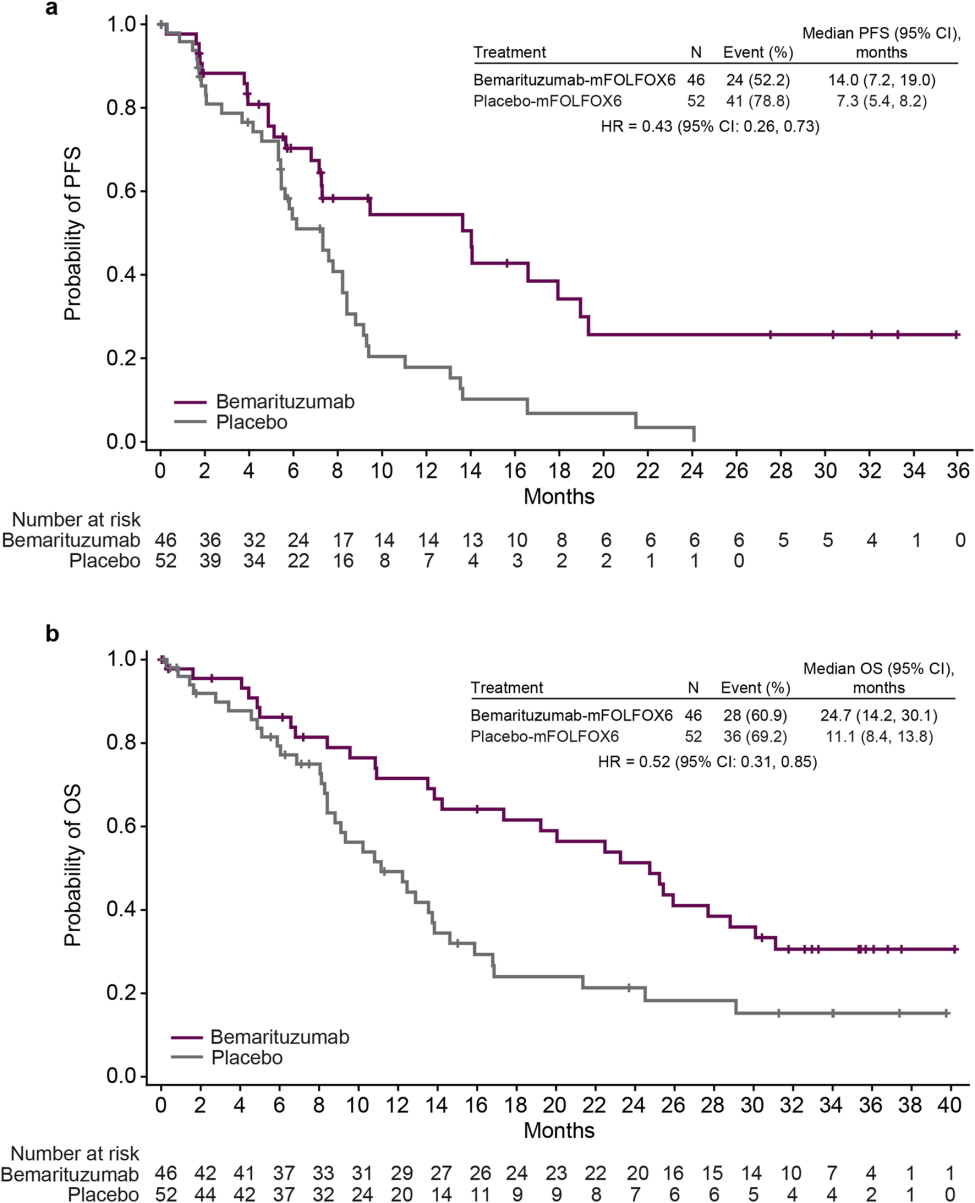
PFS and OS in the FGFR2b ≥ 10% subgroup. **a** PFS in the FGFR2b ≥ 10% subgroup. **b** OS in the FGFR2b ≥ 10% subgroup. The FGFR2b ≥ 10% subgroup included patients with FGFR2b tumor staining score of 2 + or 3 + in at least 10% of tumor cells by immunohistochemistry. HRs and 95% CIs were calculated using the unstratified Cox proportional hazards model. Vertical bars indicate censoring. *CI* confidence interval; *FGFR2b* IIIb splice isoform of fibroblast growth factor receptor 2; *HR* hazard ratio; *mFOLFOX6* modified FOLFOX (infusional 5-fluorouracil, leucovorin, and oxaliplatin); *OS* overall survival; *PFS* progression-free survival

The baseline demographics and disease characteristics were balanced between treatment arms for the ≥ 10% subgroup (data not shown). A prespecified subgroup efficacy analysis was performed for patients in the FGFR2b ≥ 10% subgroup. In these patients, the median PFS was 14.0 months (95% CI 7.2–19.0) with bemarituzumab-mFOLFOX6 and 7.3 months (95% CI 5.4–8.2) with placebo-mFOLFOX6 (HR 0.43; 95% CI 0.26–0.73) (Fig. 3a), with a 12-month estimated PFS rate of 54.4% (95% CI 36.6–69.2) and 17.8% (95% CI 7.9–31.0), respectively. The median OS was 24.7 months (95% CI 14.2–30.1) with bemarituzumab-mFOLFOX6 and 11.1 months (95% CI 8.4–13.8) with placebo-mFOLFOX6 (HR 0.52; 95% CI 0.31–0.85) (Fig. 3b). The OS landmarks for the bemarituzumab-mFOLFOX6 and placebo-mFOLFOX6 arms, respectively, were 71.5% and 49.2% at 12 months, and 51.3% and 21.3% at 24 months. In the bemarituzumab-mFOLFOX6 arm, the ORR was 56.5% (95% CI 41.1–71.1), with a CR and PR in 2 (4.3%) and 24 (52.2%) patients, respectively (Table 2). In the placebo-mFOLFOX6 arm, the ORR was 36.5% (95% CI 23.6–51.0), with a CR and PR in 1 (1.9%) and 18 (34.6%) patients, respectively. The best percentage change in tumor size from baseline is shown in Supplementary Fig. 1.

### Subsequent therapies

In all randomized patients, subsequent therapy after progression was well balanced between treatment arms, with 44 (57.1%) patients receiving at least one other line of therapy in the bemarituzimab-mFOLFOX6 arm versus 45 (57.7%) patients in the placebo-mFOLFOX6 arm (Supplementary Table 1). The most common agents received were taxanes (41.3%), the vascular endothelial growth factor/receptor (VEGF/VEGFR) inhibitor ramucirumab (25.2%), the topoisomerase 1 (TOP1) inhibitor irinotecan (20.6%), and programmed death receptor 1 (PD-1) or its ligand (PD-L1) inhibitors (11.6%). In the FGFR2b ≥ 10% subgroup, 28 (60.9%) and 27 (51.9%) patients received at least one new anticancer therapy in the bemarituzumab-mFOLFOX6 and placebo-mFOLFOX6 arms, respectively (Supplementary Table 2). The most common agents received were taxanes (40.8%), the VEGF/VEGFR inhibitor ramucirumab (27.6%), the TOP1 inhibitor irinotecan (17.3%), and PD-1/PD-L1 inhibitors (11.2%).

### Safety

All 76 patients in the bemarituzumab-mFOLFOX6 arm and 76 (98.7%) patients in the placebo-mFOLFOX6 arm had at least one treatment-emergent AE (TEAE; Table 3). At least one grade ≥ 3 TEAE occurred in 63 (82.9%) and 58 (75.3%) patients in the bemarituzumab-mFOLFOX6 and placebo-mFOLFOX6 arms, respectively (Table 3). TEAEs related (TRAEs) to any study agent were reported in 72 (94.7%) and 73 (94.8%) patients in the bemarituzumab-mFOLFOX6 and placebo-mFOLFOX6 arms, respectively. TEAEs leading to discontinuation of bemarituzumab or placebo were reported in 31 (40.8%) and 4 (5.2%) patients in the bemarituzumab-mFOLFOX6 and placebo-mFOLFOX6 arms, respectively (Table 3). Corneal AEs accounted for the majority of TEAEs that led to discontinuation of bemarituzumab (24/31 [77.4%] patients), whereas no patients discontinued treatment due to corneal AEs in the placebo-mFOLFOX6 arm. TEAEs led to dose reductions and dose delays of bemarituzumab/placebo in 9 (11.8%) and 51 (67.1%) patients, respectively, in the bemarituzumab-mFOLFOX6 arm, and in 7 (9.1%) and 41 (53.2%) patients in the placebo-mFOLFOX6 arm.

**Table 3:** Summary of TEAEs (safety analysis set)

	Bemarituzumab-mFOLFOX6 (N = 76)	Placebo-mFOLFOX6 (N = 77)
Any TEAE	76 (100.0)	76 (98.7)
Serious TEAEs	26 (34.2)	28 (36.4)
Grade ≥ 3 TEAEs	63 (82.9)	58 (75.3)
TEAEs related to any study agent	72 (94.7)	73 (94.8)
Grade ≥ 3 TEAEs related to any study agent	57 (75.0)	48 (62.3)
TEAEs leading to discontinuation of bemarituzumab/placebo	31 (40.8)	4 (5.2)
Deaths	53 (69.7)	54 (70.1)
Fatal TEAEs	5 (6.6)	4 (5.2)

Commonly reported TEAEs	Any grade	Grade ≥ 3	Any grade	Grade ≥ 3
Nausea	37 (48.7)	0	41 (53.2)	3 (3.9)
Neutrophil count decreased	31 (40.8)	23 (30.3)	33 (42.9)	28 (36.4)
Diarrhoea	31 (40.8)	3 (3.9)	24 (31.2)	1 (1.3)
Anaemia	26 (34.2)	7 (9.2)	28 (36.4)	11 (14.3)
Decreased appetite	23 (30.3)	1 (1.3)	29 (37.7)	2 (2.6)
Constipation	23 (30.3)	0	25 (32.5)	1 (1.3)
Vomiting	23 (30.3)	2 (2.6)	24 (31.2)	2 (2.6)
Aspartate aminotransferase increased	24 (31.6)	5 (6.6)	15 (19.5)	2 (2.6)
Abdominal pain	18 (23.7)	2 (2.6)	20 (26.0)	3 (3.9)
Fatigue	16 (21.1)	1 (1.3)	21 (27.3)	3 (3.9)
Asthenia	20 (26.3)	6 (7.9)	16 (20.8)	3 (3.9)
Stomatitis	26 (34.2)	7 (9.2)	10 (13.0)	2 (2.6)
Platelet count decreased	14 (18.4)	1 (1.3)	21 (27.3)	0
Alanine aminotransferase increased	23 (30.3)	2 (2.6)	11 (14.3)	1 (1.3)
Peripheral sensory neuropathy	15 (19.7)	6 (7.9)	15 (19.5)	4 (5.2)
Neutropenia	15 (19.7)	10 (13.2)	13 (16.9)	7 (9.1)
White blood cell count decreased	16 (21.1)	5 (6.6)	12 (15.6)	5 (6.5)
Dry eye	21 (27.6)	2 (2.6)	5 (6.5)	0
Weight decreased	16 (21.1)	0	10 (13.0)	0
Pyrexia	11 (14.5)	0	14 (18.2)	0
Neuropathy peripheral	13 (17.1)	3 (3.9)	11 (14.3)	1 (1.3)
Paraesthesia	13 (17.1)	1 (1.3)	10 (13.0)	1 (1.3)
Epistaxis	17 (22.4)	0	3 (3.9)	0
Leukopenia	9 (11.8)	2 (2.6)	9 (11.7)	5 (6.5)
Cough	8 (10.5)	0	8 (10.4)	0
Dyspepsia	7 (9.2)	0	9 (11.7)	1 (1.3)
Pruritus	8 (10.5)	0	8 (10.4)	0
Thrombocytopenia	11 (14.5)	1 (1.3)	4 (5.2)	1 (1.3)
Hypoalbuminemia	4 (5.3)	0	10 (13.0)	2 (2.6)
Vision blurred	13 (17.1)	0	1 (1.3)	0
Hypokalemia	4 (5.3)	2 (2.6)	8 (10.4)	3 (3.9)
Keratitis	11 (14.5)	3 (3.9)	1 (1.3)	0
Mucosal inflammation	8 (10.5)	1 (1.3)	4 (5.2)	0
Punctate keratitis	10 (13.2)	4 (5.3)	2 (2.6)	0
Corneal disorders	3 (3.9)	3 (3.9)	0	0
Cataract	2 (2.6)	2 (2.6)	0	0
Corneal erosion	2 (2.6)	2 (2.6)	0	0

Data are number (%) of patients. AEs were coded according to MedDRA version 25.0. TEAEs began on or after the study drug start date up to 28 days after the last dose of any study drug. Multiple AEs were counted only once per patient for each PT. PTs were presented by descending order of the total frequencies

*AE* adverse event; *MedDRA* Medical Dictionary for Regulatory Activities; *mFOLFOX6* modified FOLFOX (infusional 5-fluorouracil, leucovorin, and oxaliplatin); *PT* preferred term; *TEAE* treatment-emergent adverse event

Serious TEAEs occurred in 26 (34.2%) and 28 (36.4%) patients in the bemarituzumab-mFOLFOX6 and placebo-mFOLFOX6 arms, respectively. Fatal TEAEs were reported in 5 (6.6%) patients in the bemarituzumab-mFOLFOX6 arm and in 4 (5.2%) patients in the placebo-mFOLFOX6 arm.

Any-grade corneal AEs were reported in 51 (67.1%) patients in the bemarituzumab-mFOLFOX6 arm, with a median time to onset of 16.9 weeks (interquartile range [IQR], 10.1–24.0), and in 8 (10.4%) patients in the placebo-mFOLFOX6 arm, with a median time to onset of 11.6 weeks (IQR, 7.7–16.6); Grade 3 corneal AEs were reported in 21 (27.6%) patients in the bemarituzumab-mFOLFOX6 arm and none were reported in the placebo-mFOLFOX6 arm. No serious or grade ≥ 4 corneal AEs were observed in either arm. In the bemarituzumab-mFOLFOX6 arm, corneal AEs resolved in 27 patients, with a median time to resolution of 24.4 weeks (IQR, 13.0–41.1), and the median time to resolution or downgrade to grade 1 (from grade ≥ 2) was 20.3 weeks (IQR, 9.1–31.1) in 22 patients. In the placebo-mFOLFOX6 arm, corneal AEs resolved in two patients, with a median time to resolution of 1.4 weeks (IQR, 0.9–2.0), and the median time to resolution or downgrade to grade 1 (from grade ≥ 2) was 2.0 weeks (IQR, 2.0–2.0) in one patient. All corneal AEs resolved in 23 (30.3%) patients in the bemarituzumab-mFOLFOX6 arm and in 3 (3.9%) patients in the placebo-mFOLFOX6 arm. Commonly reported corneal TEAEs and time to resolution are summarized in Table 4.

**Table 4:** Summary of corneal TEAEs (safety analysis set)

Category	Bemarituzumab-mFOLFOX6 (N = 76)	Placebo-mFOLFOX6 (N = 77)
Any corneal AE	51 (67.1)	8 (10.4)
Grade ≥ 3 corneal AEs	21 (27.6)	0
Time to onset of any-grade corneal AEs		
Number of patients	51	8
Median (IQR), weeks	16.9 (10.1–24.0)	11.6 (7.7–16.6)
Time to onset of ≥ grade 2 corneal AEs		
Number of patients	37	2
Median (IQR), weeks	23.7 (16.1–33.1)	12.8 (9.0–16.6)
Corneal disorders leading to drug discontinuation	24 (31.6)	0
Commonly reported corneal AEs		
Dry eye	21 (27.6)	5 (6.5)
Keratitis	12 (15.8)	1 (1.3)
Punctate keratitis	11 (14.5)	2 (2.6)
Corneal epithelium defect	9 (11.8)	0
AE resolution category		
All corneal TEAEs resolved	23 (30.3)	3 (3.9)
All corneal TEAEs resolved or downgraded to grade 1	12 (15.8)	4 (5.2)
Time to resolution of any-grade corneal AEs		
Number of patients	27	2
Median (IQR), weeks	24.4 (13.0–41.1)	1.4 (0.9–2.0)
Time to resolution/downgrading of grade ≥ 2 corneal AEs to grade 1		
Number of patients	22	1
Median (IQR), weeks	20.3 (9.1–31.1)	2.0 (2.0–2.0)

Data are number (%) of patients unless indicated otherwise. AEs were coded according to MedDRA version 25.0. Severity grades were defined per CTCAE version 5.0. Time to resolution was calculated among patients with AE onset

*AE* adverse event; *CTCAE* Common Terminology Criteria for Adverse Events; *IQR* interquartile range; *MedDRA* Medical Dictionary for Regulatory Activities; *mFOLFOX6* modified FOLFOX (infusional 5-fluorouracil, leucovorin, and oxaliplatin); *TEAE* treatment-emergent adverse event

## Discussion

This randomized, double-blind, placebo-controlled phase 2 trial was designed to evaluate the bemarituzumab-mFOLFOX6 combination in patients with HER-2 non-positive, FGFR2b-selected, treatment-naïve advanced GC. Because the study design was changed from a confirmatory phase 3 to a phase 2 study, it was not powered to assess statistically significant improvements in PFS and OS [9]. The primary analysis showed a clinically meaningful improvement in PFS with bemarituzumab-mFOLFOX6 treatment compared with placebo-mFOLFOX6 (HR 0.68; 95% CI 0.44–1.04; *P* = 0.07) [9]. At this final analysis conducted after a minimum follow-up of 24 months, bemarituzumab-mFOLFOX6 treatment continued to show promising clinical efficacy and manageable toxicities versus placebo-mFOLFOX6 in FGFR2b-positive advanced GC. The benefits in PFS (HR 0.43; 95% CI 0.26–0.73), OS (HR 0.52; 95% CI 0.31–0.85), and ORR (difference between arms in ORR 20.0%; 95% CI 0.6–39.4) with bemarituzumab-mFOLFOX6 versus placebo-mFOLFOX6 were more pronounced in the FGFR2b ≥ 10% subgroup than in the overall population. No new safety signals were reported. Adequately powered phase 3 confirmatory studies are ongoing to determine whether there are statistically significant improvements in efficacy with this combination strategy.

In the ITT population, the HR for OS at the primary analysis was 0.58 (95% CI 0.35–0·95), and was 0.77 (95% CI 0.52–1.14) at this final analysis [9]. This final analysis HR for OS is similar to point estimates recently reported in successful phase 3 trials involving similar control arms, such as the KEYNOTE-859 (HR 0.78; 95% CI 0.70–0.87), CheckMate 649 (HR 0.79; 95% CI 0.71–0.88), SPOTLIGHT (HR 0.75; 95% CI 0.60–0.94), GLOW (HR 0.76; 95% CI 0.35–1.64), and RATIONALE 305 (HR 0.74; 95% CI 0.59–0.94) trials [19–23]. Compared with the primary analysis, the treatment benefit with bemarituzumab-mFOLFOX6 at this final analysis could have been weakened due to a potential impact of subsequent anticancer therapies on OS reflected in the additional follow-up. In the FGFR2b ≥ 10% subgroup, the HR for OS at the primary analysis increased from 0.41 to 0.52 at this final analysis—a finding that still supports promising activity for bemarituzumab in this subset, with a noteworthy 2-year OS rate in bemarituzumab-mFOLFOX6 more than twice that of placebo-mFOLFOX6 (51.3% versus 21.3%). Collectively, together with the FIH dose-escalation/dose-expansion study showing bemarituzumab single-agent activity in an FGFR2b selected gastroesophageal adenocarcinoma population, these data support FGFR2b as a potentially important predictor of response to treatment with bemarituzumab [17].

The safety results were generally similar to those reported at the primary analysis. No new safety findings were observed with longer follow-up. Treatment discontinuation due to TEAEs was higher in the bemarituzumab-mFOLFOX6 arm compared with the placebo-mFOLFOX6 arm. Corneal TEAEs were the primary reason for treatment discontinuation in the bemarituzumab-mFOLFOX6 arm, which may not necessarily be due to the severity of the AE, but could be an unintended outcome of the protocol design wherein no prophylaxis was mandated and treatment was discontinued for any corneal TEAE that was not resolved or improved to grade 1 within 28 days of treatment initiation [9]. In addition, the median time to onset of grade ≥ 2 corneal events was longer than that for any-grade corneal events (23.7 weeks versus 16.9 weeks), suggesting a possible opportunity for early recognition and active management of AEs by prophylactic measures in future studies. Accordingly, the use of ocular lubricants and eyelid hygiene are being assessed in the ongoing phase 3 studies, which also exclude the 28-day requirement for corneal AE resolution in their study designs [24, 25].

This final analysis should be interpreted considering its strengths and limitations. One limitation of this study is the relatively small sample size. In addition, no prophylaxis or mitigation for corneal toxicity was included as a part of the study design. However, this study has several strengths. First, the trial prospectively enrolled a biomarker-selected population, with evaluation of biomarker-enriched subgroups such as those with ≥ 10% of tumor cells expressing FGFR2b. Second, the use of one optional cycle of “induction” FOLFOX is a novel aspect of this trial that facilitates the inclusion of an upfront biomarker-driven cohort of patients and may be used as a template for other upfront biomarker-driven studies in the future. Third, a long minimum follow-up duration of 24 months permitted sufficient follow-up of PFS and OS endpoint outcomes. Lastly, the consistent treatment benefits with respect to all efficacy endpoints in the FGFR2b ≥ 10% subgroup further support the hypothesis that increased scrutinization of FGFR2b as a biomarker may lead to increased efficacy. This analysis is similar to the subset analysis of the ToGA trial which identified that patients with higher HER-2 expression (3 + by IHC) received enhanced benefit from the trastuzumab-chemotherapy combination, reinforcing the concept that increased protein expression of an IHC biomarker can predict greater efficacy [7].

At this final analysis, bemarituzumab-mFOLFOX6 treatment continued to demonstrate promising clinical efficacy and manageable safety in FGFR2-selected, HER-2 non-positive advanced GC, with more pronounced treatment benefit in the patient subset with FGFR2b overexpression (2 + /3 + staining) in ≥ 10% of tumor cells than in those with FGFR2b overexpression in any tumor cell. These data are encouraging and support further evaluation of the bemarituzumab-mFOLFOX6 combination in patients with FGFR2b overexpressed gastric and GEJ cancer in the frontline setting. Phase 3 trials are ongoing to confirm this observed clinical benefit, with a focus on enhanced biomarker selection (NCT05052801, NCT05111626) [24, 25].

### Supplementary Information

Below is the link to the electronic supplementary material.

Supplementary file1 (DOCX 264 kb)

Supplementary file2 (PDF 3156 kb)

## Data Availability

Qualified researchers may request data from Amgen clinical studies. Complete details are available at the following: http://www.amgen.com/datasharing.
