# Supplementary material for: Bemarituzumab as first-line treatment for locally advanced or metastatic gastric/gastroesophageal junction adenocarcinoma: final analysis of the randomized phase 2 FIGHT trial
Source: Gastric Cancer. 2024 Feb 3;27(3):558–70. doi: 10.1007/s10120-024-01466-w (PMC11016503; doi:10.1007/s10120-024-01466-w)

**Bemarituzumab as first-line treatment for locally advanced or metastatic gastric/gastroesophageal junction adenocarcinoma: Final analysis of the randomized phase 2 FIGHT trial**

**Supplementary Appendix**

**Supplementary Tables and Figures**

**Supplementary Table 1: Summary of subsequent anticancer therapy – ITT set**

| ITT Analysis Set – overall population | Bemarituzumab-mFOLFOX6 | Placebo-mFOLFOX6 | Total |
| --- | --- | --- | --- |
|  | | | |
| Patients randomized | 77 | 78 | 155 |
| Patients received at least 1 new anticancer therapy | 44 (57.1%) | 45 (57.7%) | 89 (57.4%) |
|  | | | |
| All other therapeutic products | 7 (9.1%) | 7 (9.0%) | 14 (9.0%) |
| Detoxifying agents for antineoplastic treatment | 6 (7.8%) | 7 (9.0%) | 13 (8.4%) |
| Calcium folinate | 3 (3.9%) | 0 | 3 (1.9%) |
| Calcium levofolinate | 1 (1.3%) | 1 (1.3%) | 2 (1.3%) |
| Folinic acid | 2 (2.6%) | 3 (3.8%) | 5 (3.2%) |
| Levofolinate sodium | 0 | 2 (2.6%) | 2 (1.3%) |
| Sodium folinate | 0 | 1 (1.3%) | 1 (0.6%) |
| Other therapeutic products | 1 (1.3%) | 0 | 1 (0.6%) |
| Other therapeutic products | 1 (1.3%) | 0 | 1 (0.6%) |
|  | | | |
| Antianemic preparations | 1 (1.3%) | 2 (2.6%) | 3 (1.9%) |
| Folic acid and derivatives | 1 (1.3%) | 2 (2.6%) | 3 (1.9%) |
| Calcium folinate | 1 (1.3%) | 2 (2.6%) | 3 (1.9%) |
|  | | | |
| Antineoplastic agents | 44 (57.1%) | 45 (57.7%) | 89 (57.4%) |
| Anthracyclines and related substances | 1 (1.3%) | 1 (1.3%) | 2 (1.3%) |
| Doxorubicin | 0 | 1 (1.3%) | 1 (0.6%) |
| Epirubicin | 1 (1.3%) | 0 | 1 (0.6%) |
| Antineoplastic agents | 2 (2.6%) | 0 | 2 (1.3%) |
| Antineoplastic agents | 2 (2.6%) | 0 | 2 (1.3%) |
| Combinations of antineoplastic agents | 1 (1.3%) | 1 (1.3%) | 2 (1.3%) |
| Calcium folinate;fluorouracil;irinotecan hydrochloride | 1 (1.3%) | 1 (1.3%) | 2 (1.3%) |
| Folic acid analogues | 1 (1.3%) | 0 | 1 (0.6%) |
| Raltitrexed | 1 (1.3%) | 0 | 1 (0.6%) |
| Other antineoplastic agents | 2 (2.6%) | 1 (1.3%) | 3 (1.9%) |
| Disodium cantharidinate;pyridoxine | 0 | 1 (1.3%) | 1 (0.6%) |
| Endostatin | 1 (1.3%) | 0 | 1 (0.6%) |
| Irinotecan hydrochloride | 1 (1.3%) | 0 | 1 (0.6%) |
| Other cytotoxic antibiotics | 1 (1.3%) | 1 (1.3%) | 2 (1.3%) |
| Mitomycin | 1 (1.3%) | 1 (1.3%) | 2 (1.3%) |
| Other monoclonal antibodies and antibody drug conjugates | 0 | 3 (3.8%) | 3 (1.9%) |
| Ipilimumab | 0 | 1 (1.3%) | 1 (0.6%) |
| Lacnotuzumab | 0 | 1 (1.3%) | 1 (0.6%) |
| Tremelimumab | 0 | 1 (1.3%) | 1 (0.6%) |
| PD-1/PDL-1 (programmed cell death protein 1/death ligand 1) inhibitors | 10 (13.0%) | 8 (10.3%) | 18 (11.6%) |
| Durvalumab | 0 | 1 (1.3%) | 1 (0.6%) |
| Nivolumab | 4 (5.2%) | 4 (5.1%) | 8 (5.2%) |
| Pembrolizumab | 1 (1.3%) | 2 (2.6%) | 3 (1.9%) |
| Sasanlimab | 0 | 1 (1.3%) | 1 (0.6%) |
| Sintilimab | 3 (3.9%) | 0 | 3 (1.9%) |
| Tislelizumab | 2 (2.6%) | 0 | 2 (1.3%) |
| Phosphatidylinositol-3-kinase (PI3K) inhibitors | 0 | 1 (1.3%) | 1 (0.6%) |
| AZD 8186 | 0 | 1 (1.3%) | 1 (0.6%) |
| Plant alkaloids and other natural products | 1 (1.3%) | 0 | 1 (0.6%) |
| Brucea javanica oil | 1 (1.3%) | 0 | 1 (0.6%) |
| Platinum compounds | 9 (11.7%) | 5 (6.4%) | 14 (9.0%) |
| Cisplatin | 3 (3.9%) | 2 (2.6%) | 5 (3.2%) |
| Oxaliplatin | 6 (7.8%) | 3 (3.8%) | 9 (5.8%) |
| Poly (ADP-ribose) polymerase (PARP) inhibitors | 3 (3.9%) | 0 | 3 (1.9%) |
| Olaparib | 2 (2.6%) | 0 | 2 (1.3%) |
| Rucaparib | 1 (1.3%) | 0 | 1 (0.6%) |
| Pyrimidine analogues | 22 (28.6%) | 19 (24.4%) | 41 (26.5%) |
| Capecitabine | 5 (6.5%) | 0 | 5 (3.2%) |
| Fluorouracil | 11 (14.3%) | 14 (17.9%) | 25 (16.1%) |
| Gimeracil;oteracil potassium;tegafur | 2 (2.6%) | 3 (3.8%) | 5 (3.2%) |
| Pyrimidine analogues | 2 (2.6%) | 0 | 2 (1.3%) |
| Tegafur | 0 | 2 (2.6%) | 2 (1.3%) |
| Tipiracil hydrochloride;trifluridine | 1 (1.3%) | 0 | 1 (0.6%) |
| Tipiracil;trifluridine | 1 (1.3%) | 0 | 1 (0.6%) |
| Taxanes | 29 (37.7%) | 35 (44.9%) | 64 (41.3%) |
| Albumin human;paclitaxel | 1 (1.3%) | 0 | 1 (0.6%) |
| Docetaxel | 4 (5.2%) | 4 (5.1%) | 8 (5.2%) |
| Paclitaxel | 22 (28.6%) | 31 (39.7%) | 53 (34.2%) |
| Paclitaxel nanoparticle albumin-bound | 3 (3.9%) | 2 (2.6%) | 5 (3.2%) |
| Topoisomerase 1 (TOP1) inhibitors | 13 (16.9%) | 19 (24.4%) | 32 (20.6%) |
| Irinotecan | 8 (10.4%) | 14 (17.9%) | 22 (14.2%) |
| Irinotecan hydrochloride | 5 (6.5%) | 5 (6.4%) | 10 (6.5%) |
| Vascular endothelial growth factor receptor (VEGFR) tyrosine kinase inhibitors | 5 (6.5%) | 3 (3.8%) | 8 (5.2%) |
| Fruquintinib | 2 (2.6%) | 0 | 2 (1.3%) |
| Rivoceranib | 3 (3.9%) | 2 (2.6%) | 5 (3.2%) |
| Rivoceranib mesylate | 1 (1.3%) | 1 (1.3%) | 2 (1.3%) |
| VEGF/VEGFR (vascular endothelial growth factor) inhibitors | 14 (18.2%) | 25 (32.1%) | 39 (25.2%) |
| Ramucirumab | 14 (18.2%) | 25 (32.1%) | 39 (25.2%) |
|  | | | |
| Cardiac therapy | 0 | 1 (1.3%) | 1 (0.6%) |
| Other cardiac preparations | 0 | 1 (1.3%) | 1 (0.6%) |
| Paclitaxel | 0 | 1 (1.3%) | 1 (0.6%) |
|  | | | |
| Immunostimulants | 0 | 2 (2.6%) | 2 (1.3%) |
| Other immunostimulants | 0 | 2 (2.6%) | 2 (1.3%) |
| Cancer vaccines, therapeutic | 0 | 1 (1.3%) | 1 (0.6%) |
| Tumor necrosis factor nos | 0 | 1 (1.3%) | 1 (0.6%) |
|  | | | |
| Investigational drug | 0 | 1 (1.3%) | 1 (0.6%) |
| Investigational drug | 0 | 1 (1.3%) | 1 (0.6%) |
| Investigational drug | 0 | 1 (1.3%) | 1 (0.6%) |

**Supplementary Table 2: Summary of subsequent anticancer therapy – FGFR2b ≥10% subgroup**

| FGFR2b ≥10% subgroup | Bemarituzumab-mFOLFOX6 | Placebo-mFOLFOX6 | Total |
| --- | --- | --- | --- |
|  | | | |
| Patients randomized | 46 | 52 | 98 |
| Patients received at least 1 new anticancer therapy | 28 (60.9%) | 27 (51.9%) | 55 (56.1%) |
|  | | | |
| All other therapeutic products | 5 (10.9%) | 3 (5.8%) | 8 (8.2%) |
| Detoxifying agents for antineoplastic treatment | 4 (8.7%) | 3 (5.8%) | 7 (7.1%) |
| Calcium folinate | 2 (4.3%) | 0 | 2 (2.0%) |
| Folinic acid | 2 (4.3%) | 1 (1.9%) | 3 (3.1%) |
| Levofolinate sodium | 0 | 1 (1.9%) | 1 (1.0%) |
| Sodium folinate | 0 | 1 (1.9%) | 1 (1.0%) |
| Other therapeutic products | 1 (2.2%) | 0 | 1 (1.0%) |
| Other therapeutic products | 1 (2.2%) | 0 | 1 (1.0%) |
|  | | | |
| Antianemic preparations | 0 | 1 (1.9%) | 1 (1.0%) |
| Folic acid and derivatives | 0 | 1 (1.9%) | 1 (1.0%) |
| Calcium folinate | 0 | 1 (1.9%) | 1 (1.0%) |
|  | | | |
| Antineoplastic agents | 28 (60.9%) | 27 (51.9%) | 55 (56.1%) |
| Anthracyclines and related substances | 1 (2.2%) | 1 (1.9%) | 2 (2.0%) |
| Doxorubicin | 0 | 1 (1.9%) | 1 (1.0%) |
| Epirubicin | 1 (2.2%) | 0 | 1 (1.0%) |
| Antineoplastic agents | 2 (4.3%) | 0 | 2 (2.0%) |
| Antineoplastic agents | 2 (4.3%) | 0 | 2 (2.0%) |
| Combinations of antineoplastic agents | 1 (2.2%) | 1 (1.9%) | 2 (2.0%) |
| Calcium folinate;fluorouracil;irinotecan hydrochloride | 1 (2.2%) | 1 (1.9%) | 2 (2.0%) |
| Folic acid analogues | 1 (2.2%) | 0 | 1 (1.0%) |
| Raltitrexed | 1 (2.2%) | 0 | 1 (1.0%) |
| Other antineoplastic agents | 2 (4.3%) | 1 (1.9%) | 3 (3.1%) |
| Disodium cantharidinate;pyridoxine | 0 | 1 (1.9%) | 1 (1.0%) |
| Endostatin | 1 (2.2%) | 0 | 1 (1.0%) |
| Irinotecan hydrochloride | 1 (2.2%) | 0 | 1 (1.0%) |
| Other cytotoxic antibiotics | 1 (2.2%) | 1 (1.9%) | 2 (2.0%) |
| Mitomycin | 1 (2.2%) | 1 (1.9%) | 2 (2.0%) |
| Other monoclonal antibodies and antibody drug conjugates | 0 | 1 (1.9%) | 1 (1.0%) |
| Lacnotuzumab | 0 | 1 (1.9%) | 1 (1.0%) |
| PD-1/PDL-1 (programmed cell death protein 1/death ligand 1) inhibitors | 7 (15.2%) | 4 (7.7%) | 11 (11.2%) |
| Nivolumab | 3 (6.5%) | 2 (3.8%) | 5 (5.1%) |
| Pembrolizumab | 0 | 2 (3.8%) | 2 (2.0%) |
| Sintilimab | 2 (4.3%) | 0 | 2 (2.0%) |
| Tislelizumab | 2 (4.3%) | 0 | 2 (2.0%) |
| Phosphatidylinositol-3-kinase (PI3K) inhibitors | 0 | 1 (1.9%) | 1 (1.0%) |
| AZD 8186 | 0 | 1 (1.9%) | 1 (1.0%) |
| Platinum compounds | 7 (15.2%) | 3 (5.8%) | 10 (10.2%) |
| Cisplatin | 2 (4.3%) | 2 (3.8%) | 4 (4.1%) |
| Oxaliplatin | 5 (10.9%) | 1 (1.9%) | 6 (6.1%) |
| Poly (ADP-ribose) polymerase (PARP) inhibitors | 3 (6.5%) | 0 | 3 (3.1%) |
| Olaparib | 2 (4.3%) | 0 | 2 (2.0%) |
| Rucaparib | 1 (2.2%) | 0 | 1 (1.0%) |
| Pyrimidine analogues | 17 (37.0%) | 11 (21.2%) | 28 (28.6%) |
| Capecitabine | 5 (10.9%) | 0 | 5 (5.1%) |
| Fluorouracil | 7 (15.2%) | 8 (15.4%) | 15 (15.3%) |
| Gimeracil;oteracil potassium;tegafur | 2 (4.3%) | 2 (3.8%) | 4 (4.1%) |
| Pyrimidine analogues | 1 (2.2%) | 0 | 1 (1.0%) |
| Tegafur | 0 | 1 (1.9%) | 1 (1.0%) |
| Tipiracil hydrochloride;trifluridine | 1 (2.2%) | 0 | 1 (1.0%) |
| Tipiracil;trifluridine | 1 (2.2%) | 0 | 1 (1.0%) |
| Taxanes | 17 (37.0%) | 23 (44.2%) | 40 (40.8%) |
| Albumin human;paclitaxel | 1 (2.2%) | 0 | 1 (1.0%) |
| Docetaxel | 2 (4.3%) | 3 (5.8%) | 5 (5.1%) |
| Paclitaxel | 11 (23.9%) | 19 (36.5%) | 30 (30.6%) |
| Paclitaxel nanoparticle albumin-bound | 3 (6.5%) | 2 (3.8%) | 5 (5.1%) |
| Topoisomerase 1 (TOP1) inhibitors | 6 (13.0%) | 11 (21.2%) | 17 (17.3%) |
| Irinotecan | 4 (8.7%) | 6 (11.5%) | 10 (10.2%) |
| Irinotecan hydrochloride | 2 (4.3%) | 5 (9.6%) | 7 (7.1%) |
| Vascular endothelial growth factor receptor (VEGFR) tyrosine kinase inhibitors | 3 (6.5%) | 1 (1.9%) | 4 (4.1%) |
| Fruquintinib | 1 (2.2%) | 0 | 1 (1.0%) |
| Rivoceranib | 2 (4.3%) | 1 (1.9%) | 3 (3.1%) |
| Rivoceranib mesylate | 1 (2.2%) | 0 | 1 (1.0%) |
| VEGF/VEGFR (vascular endothelial growth factor) inhibitors | 9 (19.6%) | 18 (34.6%) | 27 (27.6%) |
| Ramucirumab | 9 (19.6%) | 18 (34.6%) | 27 (27.6%) |
|  | | | |
| Cardiac therapy | 0 | 1 (1.9%) | 1 (1.0%) |
| Other cardiac preparations | 0 | 1 (1.9%) | 1 (1.0%) |
| Paclitaxel | 0 | 1 (1.9%) | 1 (1.0%) |
|  | | | |
| Immunostimulants | 0 | 1 (1.9%) | 1 (1.0%) |
| Other immunostimulants | 0 | 1 (1.9%) | 1 (1.0%) |
| Tumor necrosis factor nos | 0 | 1 (1.9%) | 1 (1.0%) |
|  | | | |
| Investigational drug | 0 | 1 (1.9%) | 1 (1.0%) |
| Investigational drug | 0 | 1 (1.9%) | 1 (1.0%) |
| Investigational drug | 0 | 1 (1.9%) | 1 (1.0%) |

**Supplementary Figure 1. Waterfall plot for best change in tumor size – FGFR2b ≥10% subgroup**


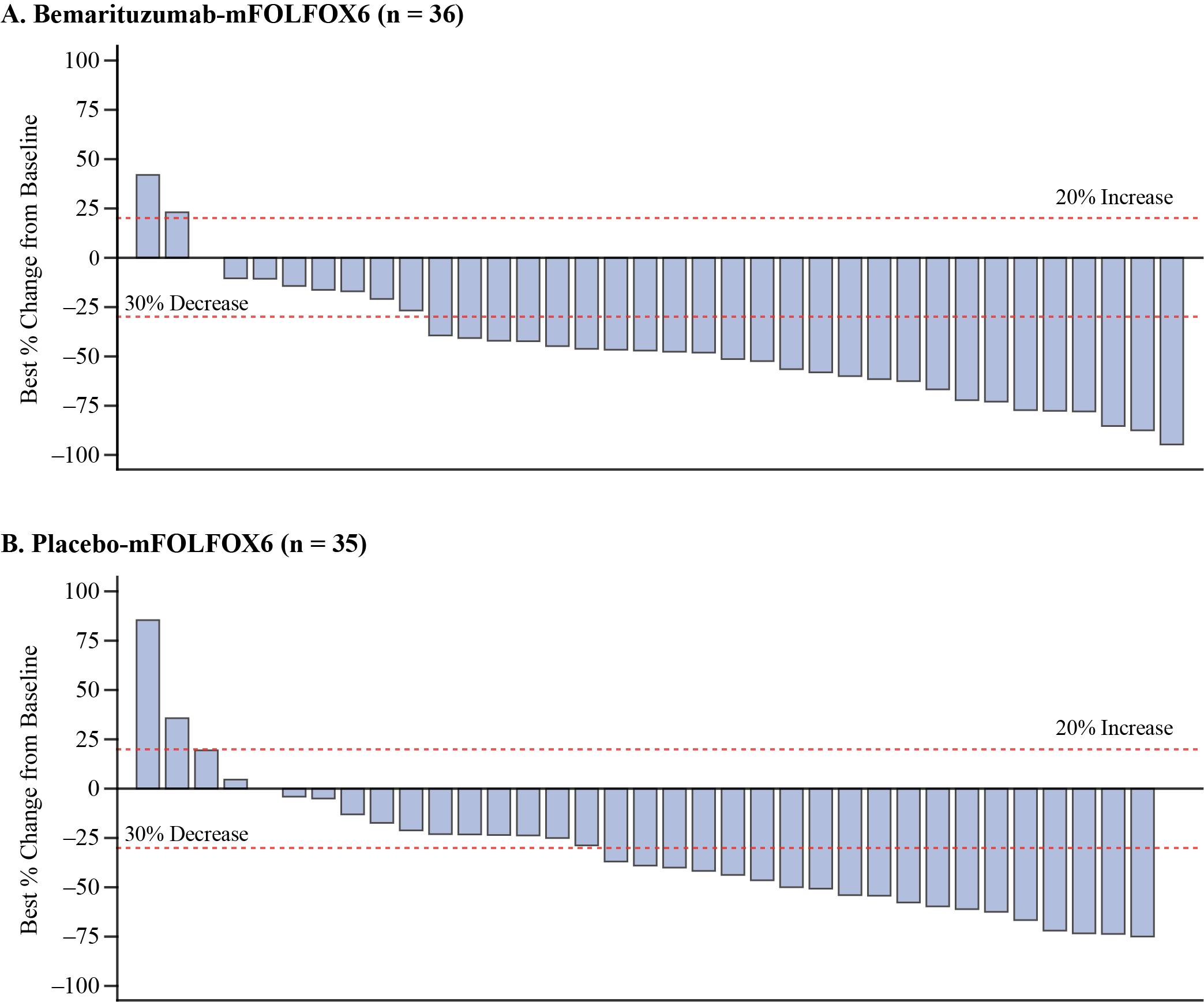

Supplement: Supplementary file 1 — Supplementary file1 (DOCX 264 kb) [file 10120_2024_1466_MOESM1_ESM.docx]
