# Supplementary material for: Bemarituzumab as first-line treatment for locally advanced or metastatic gastric/gastroesophageal junction adenocarcinoma: final analysis of the randomized phase 2 FIGHT trial
Source: Gastric Cancer. 2024 Feb 3;27(3):558–70. doi: 10.1007/s10120-024-01466-w (PMC11016503; doi:10.1007/s10120-024-01466-w)
Supplement: Supplementary file 2 — Supplementary file2 (PDF 3156 kb) [file 10120_2024_1466_MOESM2_ESM.pdf]

Bemarituzumab in FGFR2b+ Gastric/Gastroesophageal Junction Adenocarcinoma

This supplement contains the following items:

1. Original protocol, final protocol
2. Original statistical analysis plan, final statistical analysis plan

FIGHT: A Phase 1/3 Study of FPA144 versus Placebo in Combination with Modified FOLFOX6 in Patients with Previously Untreated Advanced Gastric and Gastroesophageal Cancer

Protocol Number:	FPA144-004
Investigational Product:	FPA144
IND Number:	132384
Development Phase:	Phase 1/3
Indication Studied:	Advanced Gastric and Gastroesophageal Cancer
Protocol Version:	1 – Original
Date of Protocol:	19 June 2017
Sponsor:	Five Prime Therapeutics, Inc. [redacted]
Responsible Medical Officer:	[redacted]

Confidential

This document contains proprietary and confidential information of Five Prime Therapeutics, Inc. and may not be reproduced or disclosed in whole or in part without written authorization from Five Prime Therapeutics, Inc.

Protocol Approval Signature Page

Declaration of Sponsor

A Phase 1/3 Study of FPA144 versus Placebo in Combination with Modified FOLFOX6 in Patients with Previously Untreated Advanced Gastric and Gastroesophageal Cancer

The study will be conducted according to the protocol and in compliance with Good Clinical Practice (GCP), the Declaration of Helsinki, and other applicable regulatory requirements. Essential study documents will be archived in accordance with applicable regulations.

This study protocol was subjected to critical review. The information it contains is consistent with current knowledge of the risks and benefits of the investigational product, as well as with the moral, ethical, and scientific principles governing clinical research as set out in the Declaration of Helsinki, 1989, and the International Conference on Harmonization (ICH) guidelines on GCP.

[redacted]

19 June 2017
Date

Protocol Approval Signature Page

Declaration of Sponsor

A Phase 1/3 Study of FPA144 versus Placebo in Combination with Modified FOLFOX6 in Patients with Previously Untreated Advanced Gastric and Gastroesophageal Cancer

The study will be conducted according to the protocol and in compliance with Good Clinical Practice (GCP), the Declaration of Helsinki, and other applicable regulatory requirements. Essential study documents will be archived in accordance with applicable regulations.

This study protocol was subjected to critical review. The information it contains is consistent with current knowledge of the risks and benefits of the investigational product, as well as with the moral, ethical, and scientific principles governing clinical research as set out in the Declaration of Helsinki, 1989, and the International Conference on Harmonization (ICH) guidelines on GCP.

[redacted]

19 June 2017
Date

Declaration of the Investigator

A Phase 1/3 Study of FPA144 versus Placebo in Combination with Modified FOLFOX6 in Patients with Previously Untreated Advanced Gastric and Gastroesophageal Cancer

All documentation for this study that is supplied to me and that has not been previously published will be kept in the strictest confidence. This documentation includes this study protocol, Investigator's Brochure (IB), electronic case report forms (eCRFs), and other scientific data.

The study will not be commenced without the prior written approval of a properly constituted Institutional Review Board (IRB) or Independent Ethics Committee (IEC). No changes will be made to the study protocol without the prior written approval of the Sponsor and the IRB or IEC, except as necessary to eliminate an immediate hazard to the patients.

I have read and understood and agree to abide by all the conditions and instructions contained in this protocol.

____ _____________________

Principal Investigator's Signature Date

____

Name (printed)

____

Institution or Company Name

Protocol Synopsis

Title: A Phase 1/3 Study of FPA144 versus Placebo in Combination with Modified FOLFOX6 in Patients with Previously Untreated Advanced Gastric and Gastroesophageal Cancer

Protocol Number: FPA144-004

Clinical Phase: 1/3

Sponsor: Five Prime Therapeutics, Inc.

Study Centers: Up to 250 global study centers

Phase 1 Objectives:

Primary:

- To determine the recommended dose (RD) of FPA144 in combination with a fixed dose of 5-fluorouracil (5-FU), leucovorin, and oxaliplatin (mFOLFOX6) in patients with advanced gastrointestinal (GI) tumors

Secondary:

- To evaluate the safety and tolerability of FPA144 in combination with mFOLFOX6 in patients with GI tumors
- To characterize the pharmacokinetic (PK) profile of FPA144 in combination with mFOLFOX6 in patients with GI tumors
- To characterize the immunogenicity of FPA144

Exploratory:

- To characterize the pharmacodynamic (PD) profile of FPA144 in combination with mFOLFOX6, through evaluation of exploratory biomarkers in blood samples from patients with GI tumors

Phase 3 Objectives:

Primary:

- To compare Investigator-assessed progression-free survival (PFS) in patients with FGFR2-selected gastric or gastroesophageal cancer (GC) treated with FPA144 in combination with mFOLFOX6, to those treated with placebo and mFOLFOX6

Secondary:

- To compare overall survival (OS) in patients with FGFR2-selected GC treated with FPA144 in combination with mFOLFOX6, to those treated with placebo and mFOLFOX6
- To compare objective response rate (ORR) in patients with FGFR2-selected GC and measurable disease treated with FPA144 in combination with mFOLFOX6, to those treated with placebo and mFOLFOX6

Secondary (Continued):

- To evaluate the safety and tolerability of FPA144 in combination with mFOLFOX6 compared to placebo and mFOLFOX6 in patients with FGFR2-selected GC
- To characterize PK of FPA144 in combination with mFOLFOX6 in patients with FGFR2-selected GC
- To characterize the immunogenicity of FPA144

Exploratory:

- To compare blinded independent central review (BICR)-assessed PFS in patients with FGFR2-selected GC treated with FPA144 in combination with mFOLFOX6, to those treated with placebo and mFOLFOX6
- To compare one year OS of patients with FGFR2-selected GC treated with FPA144 in combination with mFOLFOX6 to those treated with placebo and mFOLFOX6
- To compare duration of response (DOR) in patients with FGFR2-selected GC treated with FPA144 in combination with mFOLFOX6 to those treated with placebo and mFOLFOX6
- To assess patient reported outcomes (PROs) and quality of life (QOL) outcomes in patients with FGFR2-selected GC receiving FPA144 in combination with mFOLFOX6 compared to placebo and mFOLFOX6
- To explore the association between FGFR2 status (in tumor tissue and/or blood) with clinical outcome
- To explore the concordance between FGFR2 status in tumor tissue and *FGFR2* amplification in blood

Phase 1 Endpoints:

Primary:

- The incidence of Grade 2 or higher adverse events (AEs) assessed as related to FPA144 by the Investigator and the incidence of clinical laboratory abnormalities defined as dose-limiting toxicities (DLTs)

Secondary:

- The incidence of AEs, clinical laboratory abnormalities, corneal and retinal findings, and electrocardiogram (ECG) abnormalities
- PK parameters of FPA144, such as area under serum concentration-time curve (AUC), maximum serum concentration (C_{max}), trough serum concentration (C_{trough}), clearance (CL), terminal half-life ($t_{1/2}$), volume of distribution, and accumulation ratio, will be derived from the serum concentration-time profiles when applicable
- Incidence of treatment emergent anti-FPA144 antibody response

Exploratory:

- PD parameters, including exploratory biomarker analysis of the FGFR pathway, in blood

Phase 3 Endpoints:

Primary:

- PFS, defined as time from randomization until the date of radiological disease progression based on Investigator assessment (per RECIST v.1.1) or death from any cause, whichever comes first

Secondary:

- OS, defined as time from randomization until death from any cause
- ORR, defined as the proportion of patients with partial or complete response (in patients with baseline measurable disease) based on Investigator assessment of tumor lesions per RECIST v1.1
- Incidence of AEs, clinical laboratory abnormalities, corneal and retinal findings, and ECG abnormalities
- C_{max} and C_{trough} of FPA144 in combination with mFOLFOX6 will be reported when applicable
- Incidence of treatment emergent anti-FPA144 antibody response

Exploratory:

- PFS, defined as time from randomization until the date of radiological disease progression based on BICR assessment (per RECIST v.1.1) or death from any cause, whichever comes first
- One year OS, defined as the proportion of patients who are alive one year from randomization
- DOR limited to patients with measurable disease who are responders to treatment as determined by the Investigator per RECIST v1.1 and defined as the time of first response to progression or death from any cause, whichever comes first
- Change from baseline in functional outcomes as measured by EuroQOL-5D-5L (EQ-5D-5L) and the European Organization for Research and Treatment of Cancer Quality of Life Questionnaire Version 3.0 (EORTC QLQ-C30)
- The correlation between identified FGFR2 status in tumor tissue and/or ctDNA blood assay, as determined by immunohistochemistry (IHC) and blood-based molecular diagnostic assay, with PFS, OS, and objective response per RECIST v1.1
- The correlation between identified FGFR2 status in tumor tissue by IHC and *FGFR2* amplification as determined by ctDNA blood assay

**Study Design**	This is a multicenter study to evaluate the safety, tolerability, efficacy, PK, and PD of FPA144 in combination with mFOLFOX6. The study will include an open-label, Phase 1 safety run-in in patients with GI tumors (not FGFR2 selected) followed by a randomized, double-blind, placebo-controlled, Phase 3, in patients with FGFR2-selected GC (as determined by prospective IHC analysis of FGFR2b overexpression and/or a ctDNA blood assay demonstrating *FGFR2* amplification). After an initial screening period, patients will be treated with mFOLFOX6 in combination with FPA144 or placebo in 14-day cycles. In the Phase 3 part of the study, FPA144 or placebo will be referred to hereafter as Investigational Product (IP).
**Study Design (Continued):**	**Phase 1: Dose-Escalation Safety Run-in**<br><br>Phase 1 is an open-label dose-escalation of FPA144 in combination with mFOLFOX6. Eligible patients will have unresectable locally advanced or metastatic GI cancer of any type and be candidates to receive at least 2 doses of mFOLFOX6 chemotherapy. FGFR2 status is not a requirement for enrollment. FGFR2 status will be tested retrospectively by IHC (if tissue is available) and a sample will be obtained for ctDNA blood assay.<br><br>Phase 1 consists of a minimum of 2 dosing cohorts of FPA144 in combination with mFOLFOX6 to determine the RD of FPA144 to be administered in combination with mFOLFOX6. Patients may or may not have initiated or received prior mFOLFOX6 chemotherapy. There is no upper limit on the number of previous mFOLFOX6 doses that patients may have received.<br><br>Each patient enrolled will be observed for 28 days (DLT Period) starting on the first day (Cycle 1, Day 1 [Study Day 1]) of treatment with FPA144, for safety assessments, PK and occurrence of dose-limiting toxicities.<br><br>Cohorts of patients will be treated with escalating doses of FPA144 in combination with a standard dose of a chemotherapy regimen of mFOLFOX6 in 14-day cycles.<br><br>*FPA144 Administration*:<br><br>FPA144 IV is administered every 14 days on Day 1 of each cycle (1 dose = 1 cycle) prior to administration of mFOLFOX6 chemotherapy. FPA144 will be administered as an approximately 30 minute IV infusion via a peripheral vein or central venous catheter. The IV administration set for FPA144 infusion must contain a 0.22-µm in-line filter or a 0.22-µm syringe filter.

mFOLFOX6 Administration:

Administration of mFOLFOX6 chemotherapy will also commence on Cycle 1 Day 1 (Study Day 1) of each treatment cycle 30 minutes after the end of the infusion of FPA144. mFOLFOX6 will be administered every 14 days as follows:

- Day 1: Oxaliplatin 85 mg/m^2 IV infusion over 120 minutes,
- Day 1: Leucovorin 400 mg/m^2 IV infusion over 120 minutes, can be administered concurrently with oxaliplatin if using a Y connector,
- Day 1: Immediately after oxaliplatin and leucovorin, 5 FU 400 mg/m^2 bolus over approximately 5 minutes
- Day 1: Immediately after the 5-FU bolus, 5-FU 2400 mg/m^2 as a continuous IV infusion over 46 hours.

Study Design (Continued)

After the first dose, patients may receive reduced doses based on toxicity as per Section 5.4.4.

Premedication with anti-emetics may be used at the discretion of the Investigator per local standard of care.

Dose Levels in Phase 1

In Phase 1, the first dose cohort of FPA144 to be tested is at 10 mg/kg in a modified 3+3 dose escalation design. The anticipated dose levels are:

Dose level 1: 10 mg/kg FPA144
Dose level 2: 15 mg/kg FPA144
Dose level -1: 6 mg/kg FPA144 (only if dose reduction is required from Dose Level 1)

If the first cohort at 10 mg/kg clears the 28 day DLT period, the second dose cohort at 15 mg/kg will be tested in a modified 3+3 design and open to enroll 6 subjects. Dose escalation decisions will be based on an assessment of DLTs, overall safety, and tolerability, and will be made after the last patient enrolled in each cohort has completed the 28-day DLT Period (completion of 2 treatment cycles of FPA144 and mFOLFOX6). Dose escalation decisions will be agreed upon by the Cohort Review Committee (CRC), consisting of the Sponsor and Investigators. Review of safety and PK parameters may inform decisions to add cohorts with alternative dose levels to reach an optimal target exposure. Dose Level -1 will only be enrolled if ≥ 2 DLTs are observed at Dose Level 1. DLTs are defined in Section 5.4.2.

Study Design (Continued):

The following algorithm will be used for dose escalation decisions (except in the 15mg/kg cohort):

Number of Patients with DLTs	Action
0/3	Open next cohort
1/3	Enroll 3 more patients in same cohort
≥ 2/3	Stop enrollment. Enter 3 more patients at dose level below, if only 3 were previously entered
1/6	Open next cohort
≥ 2/6	Stop enrollment. Enter 3 more patients at dose level below if only 3 were previously entered, to demonstrate that ≤ 1 of 6 patients experience DLT

The RD of FPA144 for Phase 3 will be identified by the CRC based on an evaluation of the overall safety, tolerability, and PK and will not exceed 15 mg/kg administered IV every 14 days. The RD, therefore, may or may not be the same as the identified maximum tolerated dose (MTD). For example, if the MTD is not reached, or if data from subsequent cycles of treatment from Phase 1 provide additional insight on the safety profile, then the RD may be a different, though not higher, dose than the MTD.

The MTD is defined as the maximum dose at which < 33% of patients experience a DLT during the DLT Period. If a DLT is observed in 1 of 3 patients at a given dose level, then 3 additional patients will be enrolled at that same dose level. Dose escalation may continue until 2 of 3 to 6 patients treated at a dose level experience a DLT (dose level not to exceed 15 mg/kg). The next lower dose will then be considered the MTD.

Upon initiation of enrollment into the 15 mg/kg cohort, 6 patients will be enrolled to explore the safety and efficacy. The total enrollment for Phase 1 will be approximately 9 to 12 patients.

Any patient who does not receive 2 doses of FPA144 and 2 doses of mFOLFOX6 during the DLT Period due to a reason that is not a DLT or an AE related to FPA144, will be considered unevaluable and the patient will be replaced. The replaced patient may continue on study at the Investigator’s discretion and after discussion with the Sponsor. No more than 2 doses of FPA144 or 2 doses of mFOLFOX6 should be administered during the 28-day DLT Period.

Study Design (Continued):

Upon completion of the DLT Period, patients may continue receiving FPA144 in combination with mFOLFOX6 at the Investigator's discretion. Additional treatments may be administered every 14 days (1 cycle) until Investigator-assessed radiographic or clinical disease progression, unacceptable toxicity, or until the patient meets any of the other protocol-specified withdrawal criteria.

The first 3 doses of FPA144 should be administered every 14 days (±3 days) regardless of delays in mFOLFOX6 treatment. After the first 3 doses, FPA144 may be delayed up to 7 days to be synchronized with administered mFOLFOX6.

There is no mandated maximum number of doses of FPA144 or mFOLFOX6. Ongoing administration of the mFOLFOX6 regimen beyond the DLT Period will be according to local standard of care. In the Phase 1 portion of the study, if FPA144 is permanently discontinued for any reason, the patient will undergo an end of treatment (EOT) follow-up visit approximately 28 days after the last dose of FPA144. No further follow-up will be conducted for these patients and the end of FPA144 treatment is the end of study. If mFOLFOX6 is discontinued for any reason other than Investigator-assessed radiographic or clinical disease progression, or any of the other protocol-specified withdrawal criteria, FPA144 may be continued as a single agent therapy at the Investigator's discretion.

Phase 3: Randomized, Double-Blind, Placebo-Controlled Portion

Enrollment into Phase 3 will begin when an RD for FPA144, which will not exceed 15 mg/kg, has been identified by the CRC in Phase 1. Patients may enroll into either Phase 1 or Phase 3, but may not enroll in both phases of the study. Opening the Phase 3 portion of the study for enrollment will be at the discretion of the Sponsor.

Eligibility for enrollment requires patients to have unresectable locally advanced or metastatic GC, be candidates for mFOLFOX6 chemotherapy as standard first line therapy, and have a tumor that is FGFR2 positive by a centrally performed IHC tissue test and/or ctDNA blood assay. A Pre-Screening Informed Consent Form (ICF) must be signed by the patients prior to submission of tissue (archival or fresh) and a blood sample for FGFR2 testing. As receiving results of the centralized FGFR2 testing may take approximately 2 weeks, patients are allowed to receive up to one dose of a local standard of care platinum and 5-FU combination during this interim time period (Pre-Screening Period) at the discretion of the Investigator. This one dose of chemotherapy is not a requirement of the study and is not considered part of this clinical study.

Study Design (Continued):

Patients whose tumors test positive for FGFR2b by IHC and/or positive for *FGFR2* amplification by ctDNA blood assay may consent to full study participation (sign the full study ICF) and enter the Screening Period. The time between signing the full study ICF and Enrollment into the study is considered the Screening Period (up to 21 days). During the Screening Period, the patient will undergo protocol specified screening procedures to ensure all eligibility criteria are met.

The Phase 3 portion of the study is randomized, double-blind, and placebo-controlled. Approximately 360 patients will be randomized 1:1 to receive FPA144 at the RD in combination with mFOLFOX6, versus placebo in combination with mFOLFOX6 to evaluate the efficacy of the combination. An unblinded site pharmacist will supply FPA144 or saline as the placebo (details provided in a separate Pharmacy Manual). Patients must receive first administration of study treatment within 3 days of randomization.

Treatment arms consist of:

Arm 1: FPA144 at the RD and mFOLFOX6 administered every 14 days

or

Arm 2: Placebo and mFOLFOX6 administered every 14 days

Discontinuation of one component of the study treatment (mFOLFOX6, a component of mFOLFOX6, or IP) for any reason other than disease progression, does not mandate discontinuation of other components. The exception is the discontinuation of 5-FU for any reason requires discontinuation of oxaliplatin and leucovorin.

Ongoing administration of the mFOLFOX6 regimen will be according to local standard of care. All treatment decisions will be made by the Investigator using local assessments.

The first 3 doses (cycles) of IP should be administered every 14 days (±3 days) regardless of delays in mFOLFOX6 treatment. If mFOLFOX6 is delayed, then after the first 3 doses of IP, IP may be delayed up to 7 days to be synchronized with mFOLFOX6 administration. Synchronization of administration of IP and mFOLFOX6 however is not a protocol requirement. If after 7 days the patient is still unable to receive mFOLFOX6, IP should continue as monotherapy every 14 days (±3 days).

Study Design (Continued):

Patients who discontinue all study treatment (all components of mFOLFOX6 and IP) for any reason other than consent withdrawal will undergo an EOT follow-up visit approximately 28 days after the last dose of the last administered component of treatment (oxaliplatin, leucovorin, 5-FU or IP).

In addition, patients will undergo long-term follow-up for survival by clinic visit or by telephone approximately every 3 months (± 1 month) after the EOT visit until up to 24 months after the last patient is enrolled into the study, or until death, loss to follow-up, withdrawal of consent or study termination by the Sponsor (whichever occurs first).

If a patient discontinues study treatment (IP and/or mFOLFOX6) for reasons other than disease progression or withdrawal of consent, patients will have an EOT visit approximately 28 days after last dose. They will then continue to undergo tumor assessments according to the protocol schedule until radiographic progression or the initiation of additional anti-cancer therapy, at which point they would undergo long-term follow-up for survival as described in the previous paragraph.

Study Population:

Inclusion Criteria for Phase 1 and Phase 3

Patients enrolling into either Phase 1 or Phase 3 of the study must meet ***all*** of the following inclusion criteria:

1) Disease that is unresectable, locally advanced, or metastatic
2) Understand and sign an Institutional Review Board (IRB)/Independent Ethics Committee (IEC)-approved informed consent form (ICF) prior to any study-specific evaluation
3) Life expectancy of at least 3 months in the opinion of the Investigator
4) Eastern Cooperative Oncology Group (ECOG) performance status of 0 to 1
5) Age ≥18 years at the time the ICF is signed
6) Negative serum β-human chorionic gonadotropin (β-hCG) pregnancy test ≤ 72 hours prior to treatment (women of childbearing potential only)

Study Population:

7) In sexually active patients (women of child bearing potential and males), willingness to use 2 effective methods of contraception, of which 1 must be a physical barrier method (condom, diaphragm, or cervical/vault cap) until 6 months after the last dose of FPA144. Other effective forms of contraception include:
   - Permanent sterilization (hysterectomy and/or bilateral oophorectomy, or bilateral tubal ligation with surgery, or vasectomy) at least 6 months prior to Screening
   - Women of childbearing potential who are on stable oral contraceptive therapy or intrauterine or implant device for at least 90 days prior to the study, or abstain from sexual intercourse as a way of living

8) Adequate hematological and biological function, confirmed by the following laboratory values within 96 hours of Cycle 1 Day 1.

   Bone Marrow Function
   - Absolute neutrophil count (ANC) ≥ 1.5 × 10^9/L
   - Platelets ≥ 100 × 10^9/L
   - Hemoglobin ≥ 9 g/dL

   Hepatic Function
   - Aspartate aminotransferase (AST) and alanine aminotransferase (ALT) < 3 × upper limit of normal (ULN); if liver metastases, then < 5 × ULN
   - Bilirubin < 1.5 × ULN

   Renal Function
   - Calculated creatinine clearance using Cockroft Gault formula ≥ 50 mL/min (see Appendix 1)

9) Patients on full-dose anticoagulants must be on a stable dose of warfarin and have an international normalised ratio (INR) within the therapeutic range for the patient's condition or be on a stable dose of low molecular weight heparin

10) Measurable or non-measurable disease

11) Tumor tissue for determination of FGFR2 status

Study Population (Continued):

Patients enrolling into **Phase 1** of the study must also meet the following inclusion criteria:

12) Histologically or cytologically confirmed GI malignancy for which mFOLFOX6 is considered an appropriate treatment (e.g., GC, colorectal carcinoma, pancreatic adenocarcinoma)
13) Patient must be a candidate to receive at least 2 doses of mFOLFOX6 chemotherapy

Patients enrolling into **Phase 3** of the study must also meet the following inclusion criteria:

14) Histologically documented gastric or gastroesophageal junction adenocarcinoma
15) Radiographic imaging of the chest, abdomen and pelvis (computed tomography (CT) preferred, magnetic resonance imaging (MRI) acceptable) performed within 28 days of enrollment
16) FGFR2 overexpression as determined by a centrally performed IHC test and/or *FGFR2* amplification as determined by a centrally performed ctDNA blood assay
17) Patient must be a candidate for mFOLFOX6 chemotherapy
18) No prior chemotherapy for metastatic or unresectable disease (except a maximum of 1 dose of a platinum and fluoropyrimidine combination may have been administered while awaiting results of FGFR2 testing during the pre-screening period)
19) No prior platinum-based chemotherapy (except as noted in the Inclusion Criterion #18)
20) If prior adjuvant or neo-adjuvant therapy (chemotherapy and/or chemoradiation) has been received, more than 6 months must have elapsed between the end of adjuvant therapy and enrollment

Exclusion Criteria for Phase 1 and Phase 3

Patients enrolling into either Phase 1 or Phase 3 will be excluded if *any* of the following criteria apply:

- Untreated or symptomatic central nervous system (CNS) metastases (CNS imaging not required). Patients with asymptomatic CNS metastases are eligible provided they have been clinically stable for at least 4 weeks and do not require intervention such as surgery, radiation, or any corticosteroid therapy for management of symptoms related to CNS disease

Study Population (Continued):

- Impaired cardiac function or clinically significant cardiac disease, including any of the following:
  - Unstable angina pectoris ≤6 months prior to enrollment
  - Acute myocardial infarction ≤6 months prior to enrollment
  - New York Heart Association class II-IV congestive heart failure
  - Uncontrolled hypertension (as defined as ≥ 160/90 despite optimal medical management)
  - Uncontrolled cardiac arrhythmias requiring anti-arrhythmic therapy other than beta blockers or digoxin
  - Active coronary artery disease
  - QTcF ≥ 480
- Peripheral sensory neuropathy ≥ Common Terminology Criteria for Adverse Events (CTCAE) Grade 2
- Active infection requiring systemic treatment or any uncontrolled infection ≤ 14 days prior to enrollment
- Known human immunodeficiency virus (HIV) or acquired immunodeficiency syndrome (AIDS)-related illness, or known active or chronic hepatitis B or C infection
- History of interstitial lung disease (e.g., pneumonitis or pulmonary fibrosis)
- Evidence or history of bleeding diathesis or coagulopathy
- Radiotherapy ≤ 28 days of enrollment. Patients must be recovered from all acute radiotherapy-related toxicities. No radiopharmaceuticals (strontium, samarium) within 8 weeks of enrollment
- Prior treatment with any selective inhibitor (e.g., AZD4547, BGJ398, JNJ-42756493, BAY1179470) of the FGF-FGFR pathway
- Ongoing adverse effects from prior treatment > NCI CTCAE Grade 1 (with the exception of Grade 2 alopecia)
- Participation in another therapeutic clinical study or receiving any investigational agent within 28 days of enrollment or during this clinical study
- Corneal defects, corneal ulcerations, keratitis, keratoconus, history of corneal transplant, or other known abnormalities of the cornea that may pose an increased risk of developing an ulcer
- Positive HER2 status (as defined by a positive IHC test of 3+ or IHC of 2+ with positive FISH).
- Major surgical procedures are not allowed ≤28 days prior to

Study Population (Continued):

enrollment. Surgery requiring local/epidural anesthesia must be completed at least 72 hours before enrollment. In all cases the patient must be sufficiently recovered and stable before treatment administration

- Women who are pregnant or breastfeeding (unless the patient is willing to interrupt breastfeeding during study treatment administration and then resume 6 months after study discontinuation); women of childbearing potential must not consider getting pregnant during the study
- Presence of any serious or unstable concomitant systemic disorder incompatible with the clinical study (e.g., substance abuse, psychiatric disturbance, or uncontrolled intercurrent illness including arterial thrombosis, and symptomatic pulmonary embolism)
- Presence of any other condition that may increase the risk associated with study participation, or may interfere with the interpretation of study results, and, in the opinion of the Investigator, would make the patient inappropriate for entry into the study
- Known allergy or hypersensitivity to components of the FPA144 formulation including polysorbate or to platinum-containing medications, 5-FU, or leucovorin
- History of prior malignancy, except:
  - Curatively treated non-melanoma skin malignancy
  - Cervical cancer *in situ*
  - Curatively treated ductal or lobular breast carcinoma in situ and not currently receiving any systemic therapy
  - Solid tumor treated curatively more than five years previously without evidence of recurrence

No waivers of these inclusion or exclusion criteria will be granted.

Study Treatment:

In Phase 1, FPA144 will be supplied in a sterile vial for dilution into an intravenous (IV) bag for administration by the study site over approximately 30 minutes (± 5 minutes) every 14 days (±3 days) until Investigator-assessed radiographic or clinical disease progression, unacceptable toxicity, or the patient meets any of the other protocol-specified withdrawal criteria. The IV administration set for FP144 infusion must contain a 0.22 µm in-line filter or a 0.22 µm syringe filter.

In Phase 3, blinded IP (FPA144 / placebo) will be prepared and administered in a similar fashion to open-label FPA144 in Phase 1. The site pharmacist will be unblinded to prepare IP [FPA144 or placebo (saline bag)] according to guidelines provided in a separate Pharmacy Manual.

**Study Treatment (Continued):**	Oxaliplatin, 5-FU, and leucovorin will be administered by each site as per routine institutional practice. The mFOLFOX6 regimen will be administered every 14 days (± 3 days) until Investigator-assessed radiographic (Phase 1 and Phase 3) or clinical (Phase 1 only) disease progression, unacceptable toxicity, or the patient meets any of the other protocol-specified withdrawal criteria.<br><br>Refer to the most current package insert for preparation and complete prescribing information.
**Pharmacokinetic Assessments:**	Blood samples will be collected at the timepoints outlined in Appendix 6 and Appendix 7 to measure serum levels of FPA144 in all enrolled patients in Phase 1 and Phase 3, respectively.
**Immunogenicity:**	For all enrolled patients in Phase 1 and Phase 3, blood samples will be collected for anti-FPA144 antibodies at the timepoints specified in Appendix 6 and Appendix 7, respectively.
**Efficacy Assessments:**	During Phase 3, tumor response assessment will be performed by the Investigator per RECIST v.1.1 guidelines. All radiology images will be collected, stored, and available to be analyzed by BICR. Full details around independent review by a BICR will be outlined in an Independent Imaging Review Charter.<br><br>Efficacy measures will include tumor assessments consisting of clinical examination and appropriate imaging techniques, preferably CT scans of the chest, abdomen, and pelvis with appropriate slice thickness per RECIST v1.1 guidelines, but MRI acceptable. Scans will be done at Screening (within 28 days of Cycle 1 Day 1 of enrollment/randomization see Section 6.8), then every 8 weeks (± 7 days) from Screening thereafter.
**Safety Assessments:**	Safety measures will include AEs, hematology, clinical chemistry, urinalysis, vital signs, body weight, concomitant medications/procedures, ECOG performance status, targeted physical examinations, ECGs, and ophthalmology examinations in both Phase 1 and Phase 3.<br><br>An independent Data Monitoring Committee (DMC) will evaluate safety study data (AE and SAEs) on a regular basis throughout the entire treatment phase (as prescribed in the DMC Charter) in Phase 3.

**Pharmacodynamic Assessments:**	*Phase 1*<br>PD assessments will be collected at the timepoints specified in Appendix 6. Tumor tissue submitted for evaluation of FGFR2 status, if available, will be retrospectively analysed for FGFR2b overexpression using IHC.<br>Blood samples submitted for evaluation of FGFR2 status will be collected prior to the first dose of study treatment and analysed retrospectively for *FGFR2* amplification using a ctDNA blood assay.<br>Blood samples for exploratory biomarker analysis of the FGFR pathway will be collected longitudinally.<br><br>*Phase 3*<br>PD assessments will be collected at the timepoints specified in Appendix 6. Tumor tissue will be submitted for evaluation of FGFR2 status and will be prospectively analysed for FGFR2b overexpression using IHC.<br>Blood samples will be submitted for evaluation of FGFR2 status and will be prospectively analysed for *FGFR2* amplification using a ctDNA blood assay.<br>Positive results from either tissue or blood, but not both, must be available prior to enrolment.
**Statistical Procedures:**	The total enrollment planned for this study is approximately 372 patients. Approximately 9-12 patients evaluable for any dose limiting toxicity, using a modified 3+3 design, will be enrolled into Phase 1. For Phase 3, efficacy and tolerability will be evaluated by enrollment of approximately 360 patients with FGFR2-selected GC, randomized 1:1 to receive FPA144 in combination with mFOLFOX6 or placebo in combination with mFOLFOX6. Eligible patients will be stratified by geographic region (US and EU vs Asia vs Rest of World), prior treatment status (*de novo* vs adjuvant/neo-adjuvant), and administration of a single dose of platinum and fluoropyrimadine prior to enrollment (yes or no).<br><br>In Phase 1, all analyses will be descriptive and will be presented by dose group and overall as appropriate. Descriptive statistics will include number of observations, mean, standard deviation, median, range, and inter-quartile range for continuous variables, and the number and percent for categorical variables; 95% confidence intervals will be presented where appropriate.

Statistical Procedures (Continued):

In Phase 3, the primary efficacy analysis is the comparison of PFS between patients treated with FPA144 in combination with mFOLFOX6 and those treated with placebo and mFOLFOX6.

The primary endpoint, PFS, is defined as time from randomization until the date of radiological disease progression based on Investigator assessment (per RECIST v.1.1) or death from any cause, whichever comes first. The secondary efficacy endpoints include OS and ORR, whereas OS is defined as time from randomization until death from any cause, and the ORR is defined as the proportion of patients with baseline measurable disease and a partial or complete response as determined by the Investigator per RECIST v.1.1.

There will be an interim analysis and primary analysis for PFS and both are event-based analyses. A futility test of PFS will be conducted at the interim analysis after 48 events (50% of target 96 PFS events for primary analysis of PFS) have been observed in the enrolled patients to exclude a hazard ratio HR>0.806 for the combination of FPA144 and mFOLFOX6 compared with placebo and mFOLFOX6. It is estimated that the interim analysis will occur approximately 20 months from the first patient enrolled.

The primary analysis of PFS will be conducted when at least 96 PFS events have been observed in the first 156 enrolled patients, and will be performed using the intent-to-treat (ITT) population.

The primary analysis will include only radiographic progression events as determined by the Investigator per RECIST v.1.1 and deaths from any cause.

The primary analysis of PFS will be conducted using a stratified log-rank 2-sided test with a 0.05 level of significance. The stratification factors will be the same used to stratify the randomization schedule as documented in the interactive voice and Web response system (IXRS).

If the p-value for the stratified log-rank test is statistically significant (< 0.05 two-sided) and the HR is < 1, the null hypothesis of no difference in PFS will be rejected and it will be inferred that PFS is statistically prolonged in the group receiving FPA144 in combination with mFOLFOX6 compared with the group receiving placebo and mFOLFOX6.

Statistical Procedures (Continued):

The median PFS and the associated 95% confidence interval for each treatment arm will be estimated using the Kaplan-Meier method. The hazard ratio (HR= $\lambda_{FPA144+\ mFOLFOX6}/\ \lambda_{mFOLFOX6}$) will be estimated using a Cox regression model with treatment group as the only main effect and stratifying by the same stratification factors as were used for the log-rank test. An unstratified HR will also be presented.

Analyses of secondary endpoints including OS, and ORR, will be conducted when the hypothesis of primary endpoint, PFS, is tested statistically significant, and formal hypotheses of OS and ORR will be tested hierarchically at a level of 0.05. The OS will be tested first and if it is significant, the ORR will be tested next. The type I error rate of testing primary and secondary endpoints will be in control by employing this gatekeeping testing procedure at a level of 0.05.

There will be a planned interim and final analysis for OS. The interim analysis of OS will be conducted at the time of primary analysis of PFS. Should OS be analyzed, the interim analysis of OS at the primary analysis of PFS (i.e., when at least 96 PFS events have been observed in the first 156 patients), and the final analysis of OS (i.e., when 249 deaths have been observed) will be performed based on the ITT population (all randomized patients).

The hypothesis testing of OS will be conducted using a stratified log-rank 2-sided test with a 0.05 level of significance. The group sequential method will be used to allocate type I error rate based on O'Brien-Fleming boundary and type II error rate based on the Gamma family with parameter -4 at the interim and final analysis of OS. The stratification factors will be the same used to stratify the randomization schedule as documented in the IXRS.

The median OS and the associated 95% confidence interval for each treatment arm will be estimated using the Kaplan-Meier method. The HR will be estimated using a Cox regression model with treatment group as the only main effect and stratifying by the same stratification factors used for the log-rank test. An unstratified HR will also be presented.

The analysis of ORR will be performed among the patients with baseline measurable disease. In the analysis of ORR, patients who do not have any post-baseline adequate tumor assessments will be counted as non-responders. Formal hypothesis testing of ORR will be performed using the stratified Cochran-Mantel-Haenszel test at a level of 0.05(2-sided). The stratification factors will be the same used to stratify the randomization schedule as documented in the IXRS.

**Statistical Procedures (Continued):**	**Power and Sample Size:** This study is designed to provide adequate power for a primary analysis of PFS.<br><br>Based on a median PFS (mPFS) of 6 months for patients receiving placebo and mFOLFOX6, approximately 156 patients (randomized 1:1) with a target of 96 PFS events are required to detect a hazard ratio (HR) of 0.5 for PFS with a power of 90% (2-sided α =0.05) for the combination of FPA144 and mFOLFOX6 compared with placebo and mFOLFOX6 after 24 months of accrual and 6 months of follow up. There is an interim analysis for futility, in which the futility boundary is non-binding.<br><br>Assuming an exponential distribution of PFS, the clinical hypothesis of HR = 0.5 corresponds to an increase in mPFS from 6 months to 12 months. In the current design, the minimum observed treatment effect that would result in statistical significance for PFS is a 50% improvement (HR = 0.67) from 6 to 9 months.<br><br>This study is also powered for analysis of OS.
**Statistical Procedures (Continued):**	Based on a median OS (mOS) for patients receiving placebo and mFOLFOX6 of 10 months, enrollment of the study will continue to approximately 360 patients with a target of 249 death events to demonstrate an HR of 0.7 for OS with a power of 80% at the overall type I error level of 0.05 for the combination of FPA144 and mFOLFOX6 compared to placebo and mFOLFOX6 after 36 months of accrual and 10 months of follow-up after enrollment of the last patient.<br><br>Assuming an exponential distribution of OS, the clinical hypothesis of HR=0.7 corresponds to an increase of 43% in median OS from 10 months to 14.3 months. In the current design, the minimum observed treatment effect that would result in statistical significance for OS at the final analysis is a 28% improvement (HR = 0.78) from 10 to 12.8 months.

**Statistical Procedures (Continued):**	<u>**Safety Analysis:**</u> All AEs will be coded using the Medical Dictionary for Regulatory Activities (MedDRA). The Investigator will classify the severity of AEs using the CTCAE v 4.03. A treatment emergent adverse event (TEAE) is defined as any event with an onset date on or after date of first dose of study treatment, or any event present before treatment that worsens after treatment. Only TEAEs with an onset date prior to date of last dose + 28 days will be tabulated in summary tables.<br><br>Clinical laboratory data will be summarized by the type of laboratory test. The number and percentage of patients who experience abnormal (i.e., outside of reference ranges) and/or clinically significant abnormalities after study treatment administration will be presented for each clinical laboratory measurement. For each clinical laboratory measurement, descriptive statistics will be provided for baseline and all subsequent post-treatment scheduled visits. Changes from baseline to the post-treatment visits will also be provided. Descriptive statistics of vital signs will also be provided in a similar manner. In addition, shift from baseline in CTCAE grade (where applicable) and by high/low flags (where CTCAE grades are not defined) will be presented in a similar manner.
**Statistical Procedures (Continued):**	**Safety Analyses in Phase 1**<br><br>Safety analyses will be performed for patients included in the safety population. The incidence of DLTs, incidence of TEAEs, clinical laboratory abnormalities (e.g. shift table), vital signs, corneal and retinal findings, and ECGs will be tabulated and summarized by dose level.<br><br>**Safety Analyses in Phase 3**<br><br>The analyses of safety will include all patients who receive any study treatment (FPA144 in combination with mFOLFOX6, or placebo and mFOLFOX6) throughout the study and will provide any post-treatment safety information. The incidence of TEAEs, clinical laboratory abnormalities, vital signs, corneal and retinal findings, and ECGs will be tabulated and summarized by treatment group.<br><br>No formal comparisons of safety endpoints are planned.<br><br><u>**PK Analysis:**</u> PK parameters will be characterized using non-compartmental analysis, though compartment analysis may be employed if appropriate.

List of Key Study Personnel

Sponsor:	Five Prime Therapeutics, Inc.<br>[redacted]
Responsible Medical Officer:	[redacted]
Contract Research Organization:	ICON Clinical Research Limited<br>[redacted]
Serious Adverse Event Reporting:	ICON Medical and Safety Services<br>[redacted]

TABLE OF CONTENTS

Protocol Approval Signature Page 2
Declaration of the Investigator 3
Protocol Synopsis 4
List of Key Study Personnel 23
Table of Contents 24
List of Tables 30
List of Figures 30
List of Appendices 31
List of Abbreviations and Definitions 32
1 Introduction 35
1.1 General Gastric Cancer 35
1.2 Targeted Agents 35
1.3 FPA144 36
1.3.1 Background 36
1.3.2 FPA144, an FGFR2b-specific Antibody 36
1.3.3 Nonclinical Studies with FPA144 37
1.3.3.1 In Vivo Pharmacology 37
1.3.3.2 Analysis of Immune Effector Functions of FPA144 39
1.3.3.2.1 In vivo ADCC Activity of FPA144 40
1.3.3.3 FPA144 Exposure Efficacy Relationships 41
1.3.4 Toxicology 41
1.3.5 Clinical Experience with FPA144 43
1.4 Rationale for mFOLFOX6 45
1.5 Rationale for Combination Therapy: FPA144 and mFOLFOX6 46
1.6 FPA144 and mFOLFOX6 Starting Dose Justification 47
1.7 Risk-Benefit Assessment of FPA144 and mFOLFOX6 48
1.8 Rationale for Phase 3 Pre-screening 50
1.9 Rationale for Tumor Tissue and Blood Assessments 51

2 Study Objectives and Endpoints 52
2.1 Phase 1: Primary Objectives 52
2.2 Phase 1: Secondary Objectives 52
2.3 Phase 1: Exploratory Objectives 52
2.4 Phase 3: Primary Objectives 52
2.5 Phase 3: Secondary Objectives 52
2.6 Phase 3: Exploratory Objectives 52
2.7 Phase 1: Primary Study Endpoints 53
2.8 Phase 1: Secondary Endpoints 53
2.9 Phase 1: Exploratory Endpoints 53
2.10 Phase 3: Primary Endpoint 53
2.11 Phase 3: Secondary Endpoints 53
2.12 Phase 3: Exploratory Endpoints 54
3 Overall Design and Plan of the Study 54
3.1 Study Overview 54
3.1.1 Phase 1 54
3.1.2 Phase 3 55
3.2 Study Screening (Phase 1 and Phase 3) and Study Prescreening (Phase 3) 55
3.2.1 Phase 1 55
3.2.2 Phase 3 56
3.3 Study Enrollment 56
3.3.1 Phase 1 56
3.3.2 Phase 3 56
3.4 Phase 1 Study Treatment Period 56
3.5 Phase 3 Study Treatment Period 59
3.6 Procedures 61
3.7 Study Schema 62
3.8 Rationale for the Study Design 65
4 Study Eligibility and Withdrawal Criteria 66

4.1 Planned Number of Patients and Study Centers 66
4.2 Inclusion Criteria for Phase 1 and Phase 3 66
4.3 Exclusion Criteria for Phase 1 and Phase 3 68
4.4 Patient Identification and Enrollment 69
4.5 Patient Withdrawal and Replacement 70
5 Study Treatment 71
5.1 FPA144 Identity 71
5.2 Administration 71
5.2.1 mFOLFOX6 71
5.2.2 Open-label FPA144 (Phase 1) and Blinded Investigational Product (IP) (Phase 3) 72
5.3 Packaging, Labeling, and Storage 72
5.4 Starting Dose and Dose Modifications 73
5.4.1 Dose Escalation within a Cohort 73
5.4.2 Dose-Limiting Toxicity 73
5.4.3 Toxicity at Lowest Dose Level 73
5.4.4 Dose Modification Criteria - Open-label FPA144 (Phase 1) and Blinded IP (Phase 3) 74
5.4.5 Dose Interruptions During Study Treatment Infusion 75
5.4.5.1 Open-label FPA144 (Phase 1) and Blinded Investigational Product (Phase 3) 75
5.4.5.2 mFOLFOX6 76
5.5 Blinding and Breaking the Blind 78
Emergency Unblinding of Treatment Assignment 79
5.6 Drug Accountability 79
5.7 Investigational Product Compliance 79
5.8 Concomitant Medication and Treatment 80
6 Parameters and Methods of Assessment 80
6.1 Safety Parameters 80
Adverse Events 80
6.1.1.1 Collection of Adverse Events 80
6.1.1.2 Definitions 81

6.1.1.3 Assessment of Adverse Events 81
6.1.1.3.1 Seriousness 82
6.1.1.3.2 Intensity (Severity) 82
6.1.1.3.3 Causality 83
6.1.1.3.4 Outcome and Action Taken 84
6.1.1.4 Reporting Serious Adverse Events 85
6.1.1.5 Follow-up of Adverse Events 86
6.1.1.6 Pregnancy 86
6.2 Laboratory Parameters 87
6.3 Vital Signs 87
6.4 Electrocardiograms 87
6.5 Physical and Ophthalmologic Examinations 88
6.6 Immunogenicity 88
6.7 Tumor Analysis for Patient Selection 89
6.7.1 Phase 1 89
6.7.2 Phase 3 89
6.8 Tumor Assessments 89
6.9 ctDNA Blood Assay 90
6.10 Pharmacodynamic Biomarker Analysis Using Blood 90
6.11 Quality of Life Scales 90
6.12 ECOG Performance Status 91
6.13 Pharmacokinetic Assessment 91
7 Study Conduct 91
7.1 Overview of Patient Assessments 91
7.2 Study Assessments and Procedures by Visit - Phase 1 91
7.2.1 Screening Period (Day –28 to Day 0) (Phase 1) 91
7.2.2 Treatment Period (Phase 1) 92
7.2.2.1 Cycle 1, Day 1 (Phase 1) 92
7.2.2.2 Cycle 1, Day 3 (Phase 1) 93
7.2.2.3 Cycle 1, Day 8 (Phase 1) 93
7.2.2.4 Cycle 2 Day 1 (Phase 1) 93
7.2.2.5 Cycle 2 Day 3 (Phase 1) 94

7.2.2.6 Cycle 3 Day 1 and Day 1 of Subsequent Odd Numbered Cycles (Except as Noted) (Phase 1) 94
7.2.2.7 Cycle 4 Day 1 and Day 1 of Subsequent Even Numbered Cycles (Phase 1) 95
7.2.2.8 Cycle 15 Day 1 and Day 1 of Every Subsequent 8 Cycles (Phase 1) 96
7.2.3 End-of-Treatment Visit or Early Termination (Phase 1) 96
7.3 Study Assessments and Procedures by Visit – Phase 3 97
7.3.1 Pre-screening Period (Phase 3) 97
7.3.2 Screening Period (Day –21 to Day 0) (Phase 3) 97
7.3.3 Treatment Period (Phase 3) 98
7.3.3.1 Cycle 1, Day 1 (Phase 3) 98
7.3.3.2 Cycle 1, Day 8 (Phase 3) 98
7.3.3.3 Cycle 2 Day 1 (Phase 3) 99
7.3.3.4 Cycle 3 Day 1 and Day 1 of Subsequent Odd Numbered Cycles (Except as Noted) (Phase 3) 99
7.3.3.5 Cycle 4 Day 1 and Day 1 of Subsequent Even Numbered Cycles (Phase 3) 100
7.3.3.6 Imaging Evaluation 100
7.3.3.7 Slit Lamp Examinations 101
7.3.4 End-of-Treatment Visit or Early Termination (Phase 3) 101
7.3.5 Additional Follow-up for Patients Without Progression at End-of-Treatment Visit (Phase 3) (Scan Follow-up) 101
7.3.6 Long-term Follow-Up (Phase 3) 102
8 Statistical Methods 102
8.1 Analysis Populations 102
8.2 General Considerations 103
8.3 Efficacy Analyses 104
8.3.1 Analysis of Primary Efficacy Endpoint 104
8.3.2 Analysis of Secondary Efficacy Endpoints 105
8.3.3 Analysis of Exploratory Efficacy Endpoints 106
8.4 Safety Analyses 106
8.4.1 Safety Analyses in Phase 1 107
8.4.2 Safety Analyses in Phase 3 107
8.5 Pharmacokinetic Analyses 107

8.6 Interim Analyses 107

8.7 Changes in the Planned Analyses 108

9 Ethical, Legal, and Administrative Aspects 108

9.1 Data Quality Assurance 108

9.2 Electronic Case Report Forms and Source Documentation............................108

9.3 Access to Source Data 108

9.4 Data Processing 109

9.5 Archiving Study Records..109

9.6 Good Clinical Practice 110

9.7 Informed Consent..110

9.8 Protocol Approval and Amendment...110

9.9 Study Committee and Central Review 111

9.9.1 Cohort Review Committee (CRC)...111

9.9.2 Data Monitoring Committee 111

9.9.3 Blinded Independent Central Review (BICR) 111

9.10 Long-term Follow-up 112

9.11 Premature Termination of the Study 112

9.12 Confidentiality 113

9.13 Other Ethical and Regulatory Issues...113

10 References.. 114

11 Appendices... 120

LIST OF TABLES

Table 1: Dose Escalation....58
Table 2: Eligibility Based on FGFR2 Status....70
Table 3: Dose Modification Guidelines for FPA144 (Any Non-corneal, Non-infusion Toxicity[a]) 74
Table 4. Dose Modification Guidelines for FPA144 (Any Related Corneal Toxicity[a])....75
Table 5: Dose Reductions and Delays for mFOLFOX6 Chemotherapy....77

LIST OF FIGURES

Figure 1: Tumor Growth Inhibition in OCUM-2 GC Cell Line....38
Figure 2: In Vitro ADCC Activity of FPA144....40
Figure 3: FPA144 but not an ADCC-Deficient FGFR2b Antibody Leads to Tumor Suppression in a Syngeneic Tumor Model with Modest FGFR2b Expression....41
Figure 4: Phase 1 Dose Escalation Safety Run-in....63
Figure 5: Phase 3 Randomized, Double-Blind, Placebo-Controlled Portion....64

LIST OF APPENDICES

Appendix 1: Formula to Calculate Creatinine Clearance 121
Appendix 2: Schedule of Assessments – Dose-Escalation Safety Run-in (Phase 1)...............122
Appendix 3: Schedule of Assessments –Randomized, Placebo-controlled Portion (Phase 3).125
Appendix 4: Laboratory Evaluations 129
Appendix 5: Study Flowchart for Pharmacokinetic, Immunogenicity, and Pharmacodynamic Blood Sample Collections for Phase 1 130
Appendix 6: Study Flowchart for Pharmacokinetic and Immunogenicity Blood Sample Collections for Phase 3 131
Appendix 7: ECOG Performance Status 132
Appendix 8: Fluorescein Stain Grading Systems 133

List of Abbreviations and Definitions

Abbreviation	Definition
ADCC	antibody-dependent cell-mediated cytotoxicity
AE	adverse event
AIDS	acquired immunodeficiency syndrome
ALT	alanine aminotransferase
ANC	absolute neutrophil count
AST	aspartate aminotransferase
AUC	area under serum concentration-time curve
AUC_{τ}	AUC over the dose interval τ
β-hCG	β-human chorionic gonadotropin
BICR	blinded independent central review
CDC	complement-dependent cytotoxicity
CHO	Chinese hamster ovary
CI	confidence interval
CL	total clearance
C_{max}	maximum observed serum concentration
CNS	central nervous system
CRC	Cohort Review Committee
CRO	contract research organization
CT	computed tomography
CTCAE	Common Terminology Criteria for Adverse Events
ctDNA	circulating tumor DNA
C_{trough}	minimum trough serum concentration
$C_{trough\ ss}$	trough concentration at steady state
DLT	dose-limiting toxicity
DMC	Data Monitoring Committee
DOR	duration of response
DPD	dipyrimidine dehydrogenase
EAP	etoposide/doxorubicin/cisplatin
ECF	epirubicin/cisplatin/5-FU
ECG	electrocardiogram
ECOG	Eastern Cooperative Oncology Group
eCRF	electronic case report forms
ELF	etoposide/leucovorin/5-FU

Abbreviation	Definition
ELISA	enzyme linked immunosorbent assay
EORTC	European Organisation for Research and Treatment of Cancer
EOS	end of study
EOT	end of treatment
FAM	5-FU, doxorubicin, and mitomycin C
FAMTX	5-FU/doxorubicin [Adriamycin]/methotrexate)
FGF	fibroblast growth factor
FGFR	fibroblast growth factor receptor
FISH	fluorescent in situ hybridization
FP	5-FU/cisplatin
FRS2	FGF receptor substrate-2
GC	gastric or gastroesophageal cancer
GCP	Good Clinical Practices
G-CSF	granulocyte-colony stimulating factor
GEJ	gastroesophageal junction
GI	gastrointestinal
GLP	GLP Good Laboratory Practices
HIV	human immunodeficiency virus
HNSTD	highest, non-severely toxic dose
HR	hazard ratio
IB	Investigator's Brochure
ICF	informed consent form
ICH	International Conference on Harmonization
ID	identification
IEC	Independent Ethics Committee
IHC	immunohistochemistry
IND	investigational new drug (application)
INR	international normalised ratio
IOP	intra-ocular pressure
IP	investigational product (FPA144 or placebo)
IRB	Institutional Review Board
IRC	Independent Review Committee
ITT	intent-to-treat
IV	intravenous

Abbreviation	Definition
IXRS	interactive voice and Web response system
LLOQ	lower limit of quantitation
mFOLFOX6	modified FOLFOX (infusional 5-FU, leucovorin, and oxaliplatin)
mOS	median OS
mPFS	median progression free survival
MRI	magnetic resonance imaging
MTD	maximum tolerated dose
NCI	National Cancer Institute
OCT	ocular coherence tomography
ORR	objective response rate
OS	overall survival
PD	pharmacodynamic(s)
PFS	progression-free survival
PK	pharmacokinetic(s)
PRO	patient reported outcomes
QLQ	quality of life questionnaire
QOL	quality of life
RD	recommended dose
RECIST	Response Evaluation Criteria in Solid Tumors
RPE	retinal pigment epithelium
SAE	serious adverse event
SAP	statistical analysis plan
SD	standard deviation
$t_{1/2}$	terminal half-life
TEAE	treatment emergent adverse event
TKI	tyrosine kinase inhibitor
TS	thymidylate synthase
ULN	upper limit of normal
VAS	visual analogue scale

1 Introduction

1.1 General Gastric Cancer

Gastric or gastroesophageal cancer (GC), including gastroesophageal junction (GEJ) cancer, represents the fourth most common cancer worldwide (Kamangar 2006) and is a highly lethal disease, with 5-year overall survival (OS) rates below 30% in the United States (US) regardless of stage (National Cancer Institute 2015). Though intensive multimodal therapy for locoregional disease improves survival (Waddell 2014, The GASTRIC Group 2010) it does not cure most patients and standard chemotherapy for metastatic disease provides only short-term benefits (Waddell 2014, Wagner 2006). First-line chemotherapy used in patients with metastatic or recurrent disease consists of a fluoropyrimidine (5-FU or capecitabine) with a platinum agent (usually oxaliplatin or cisplatin) (Kang 2009, Al-Batran 2008). This treatment prolongs survival by 6 months compared to best supportive care (Wagner 2006) but the benefits are only short-term with a median OS of 9 to10 months and a PFS of 5 to 5.6 months (Kang 2009, Waddell 2014). A recent study reported that patients with recurrent or metastatic disease who received first-line treatment with capecitabine and oxaliplatin had a median overall survival (mOS) of 10.4 months (Hecht 2016).

1.2 Targeted Agents

It is important to identify new treatments with acceptable toxicities for this patient population. Recent studies have identified important pathways involved in GC development. The availability of targeted agents has led to the development of strategies incorporating these agents into the therapy for patients with such a poor prognosis. Recently ramucirumab (a monoclonal antibody targeting the VEGF pathway) was approved for treatment in patients with GC who progressed following first line treatment (Wilke 2014). Overall survival for patients treated with ramucirumab with paclitaxel was 9.6 months compared to 7.4 months with paclitaxel and placebo.

One well established pathway is the human epithelial growth factor receptor 2 (HER-2 also known as ERBB2) (Gravalos 2008, Hofmann 2008). HER-2 overexpression has been identified in 9-38% of GCs depending on histology and tumor location (Gravalos 2008). The availability of trastuzumab, a monoclonal antibody targeting HER-2, led to the development of a randomized trial in newly diagnosed GC patients whose tumors overexpressed HER-2. About 22% of screened patients were HER-2 positive and the combination of standard chemotherapy with trastuzumab resulted in median overall survival of 13.8 months compared to 11.1 months for patients treated with standard chemotherapy (Bang 2010). Based on these results, this has become standard care for patients overexpressing HER-2.

In spite of these improvements, the majority of GC patients succumb to their disease, and there are few treatment options following progression after first-line chemotherapy. There are no

agents approved for treatment of patients beyond second line and best supportive care in second-line has a mOS of 2.4 to 3.8 months and a median progression free survival (mPFS) of only 1.3 to 1.8 months (Fuchs 2014, Thuss-Patience 2011, Ford 2014). Therefore, continued evaluation of agents that can target the mutations present in GC seems imperative.

Amplification of FGFR2 has recently been identified as having prognostic importance in patients with GC (Su 2014, Jung 2012, Seo 2016, Matsumoto 2012). Patients with *FGFR2* amplification appear to have a worse prognosis (Su 2014, Seo 2016), suggesting that inhibition of FGFR2 may be an important target (Jung 2012, Matsumoto 2012). FPA144 is a humanized monoclonal antibody specific to the human FGFR2b receptor that blocks FGF binding to the receptor. Evaluation of this agent in patients with GCs whose tumors have alterations of FGFR2 would be an important strategy to improve the prognosis for these patients.

1.3 FPA144

1.3.1 Background

The role of the fibroblast growth factor (FGF) receptor (FGFR) pathway in cancer is well known. FGFs can stimulate the transformation and proliferation of tumor cells and stimulate angiogenesis. There are 22 known human FGFs with the expression of individual FGFs generally restricted to specific tissues, cell types, and/or developmental stage. FGF signaling is mediated by a family of transmembrane tyrosine kinase receptors encoded by four distinct genes producing FGF receptor subtypes termed FGFR1–4 (Turner 2010).

The FGFR2 has 2 splicing variants, b and c. In general, FGFR2b is expressed in tissues of epithelial origin (e.g., stomach, skin) (Miki 1992). The major ligands signaling through FGFR2b are FGF7, FGF10 and FGF22. Alteration in signaling in the FGF/FGFR2 pathway (e.g., overexpression of FGFR2 protein or amplification of *FGFR2* gene) has been associated with gastric, breast, and other cancers, and appears to portend a worse prognosis (Wu 2013, Turner 2010). As early as 1990, subsets of patients with GC (approximately 3 to 9%) and breast cancer (1 to 2%) were noted to have amplification of the *FGFR2* gene, which resides on chromosome 10q26 (Hattori 1990, Turner 2010). In GC, *FGFR2* amplification leads to high-level expression of the FGFR2b receptor on the surface of the cells.

1.3.2 FPA144, an FGFR2b-specific Antibody

FPA144 is a humanized monoclonal antibody (IgG1 isotype) specific to the human FGFR2b receptor (NCBI reference sequence ID NP_001138385.1) that blocks FGF ligand binding to the receptor. FPA144 is directed against the third Ig region of the FGFR2b receptor isoform, the region that is alternatively spliced and regulates ligand specificity. This antibody is glycosylated, but is produced in a Chinese hamster ovary (CHO) cell line that lacks the *FUT8* gene (α1,6-Fucosyltransferase) and therefore lacks a core fucose in the polysaccharide portion of the antibody. The absence of the core fucose results in higher affinity for the Fc receptor FcγRIIIa compared to the fucosylated molecule and potentially enhances immune cell-mediated tumor cell

killing (Shinkawa 2003). The antibody has thus been glycoengineered for enhanced antibody-dependent cell-mediated cytotoxicity (ADCC) (Gemo 2014). FPA144 inhibits FGF ligand-stimulated FGFR2b phosphorylation and cell proliferation in cell culture in FGFR2b overexpressing gastric and breast cancer cell lines. FPA144 also inhibits tumor growth in FGFR2b overexpressing gastric and breast xenograft models. The 3 potential mechanisms of action of FPA144 thus include blocking ligand binding and downstream signaling, decreasing expression of the FGFR2b driver protein, and enhancing ADCC.

FPA144 can produce complete and durable tumor growth inhibition in FGFR2b-overexpressing and *FGFR2* gene-amplified GC xenografts in immune-compromised mice where FGFR2b is considered a driver of tumor growth (Gemo 2014). In addition, FPA144 demonstrates recruitment of NK cells and concomitant tumor growth inhibition in the 4T1 syngeneic tumor model with modest expression of FGFR2b (Powers 2016). These data suggest that ADCC may be efficacious in patients without *FGFR2* gene amplification with moderate FGFR2b overexpression, and that ADCC activity may be a major contributor to the mechanism of action in these patients.

Additionally, since FPA144 is specific for the FGFR2b receptor, it does not interfere with signaling of the other FGFs/ FGFRs, including FGFR2c. In contrast to the FGFR tyrosine kinase inhibitors (TKIs), FPA144 does not inhibit FGF23 signaling. FGF23 is a ligand involved in calcium/phosphate metabolism. Thus, treatment with FPA144 is not expected to cause the dose-limiting hyperphosphatemia associated with the FGFR TKIs (Andre 2013, Brown 2005, Dienstmann 2014, Sequist 2014).

As FPA144 is a targeted biologic, the clinical development of FPA144 will ultimately be in selected patients with alterations in the FGFR2 pathway that are most likely to respond to this novel agent. The tumor types most relevant to date include gastric, bladder, and possibly cholangiocarcinoma. Each of these cancers needs new therapeutic options. The present study is designed to evaluate the efficacy, safety, and PK of FPA144 in combination with standard mFOLFOX6 chemotherapy treatment. Patients with gastrointestinal (GI) tumors will be enrolled in a Phase 1 safety run in, while the Phase 3 will enroll GC patients specifically selected for FGFR2 expression and/or *FGFR2* amplification (FGFR2 selected) who are eligible for first-line mFOLFOX6 chemotherapy.

1.3.3 Nonclinical Studies with FPA144

The 3 mechanisms of action of FPA144 described above have been explored both *in vitro* and *in vivo*.

1.3.3.1 *In Vivo* Pharmacology

FPA144 has been studied in a series of mouse xenograft models using human gastric and breast tumor cell lines that contain the *FGFR2* amplicon. These *FGFR2* amplified lines all express high levels of the FGFR2b protein and respond to FPA144 in a dose-dependent fashion. A dose

response study (twice weekly dosing) was performed with the most sensitive model, a GC line, OCUM-2 (Figure 1). Mice were treated at the indicated concentrations of FPA144, and the tumor growth was compared to mice treated with albumin alone. Statistically significant tumor growth inhibition was seen at 0.3 mg/kg, but not at 0.1 mg/kg, and tumor regression was seen at 1 mg/kg with complete tumor regression starting at doses of 1.5 mg/kg (2/15 animals), 2 mg/kg (1/15 animals), 3 mg/kg (5/15 animals), and 5 mg/kg (8/15 animals). In the SNU-16 GC model, tumor growth inhibition was seen at 1 mg/kg, while in the MFM-223 tumor-bearing mice, 5 mg/kg led to tumor stasis.

Figure 1: Tumor Growth Inhibition in OCUM-2 GC Cell Line

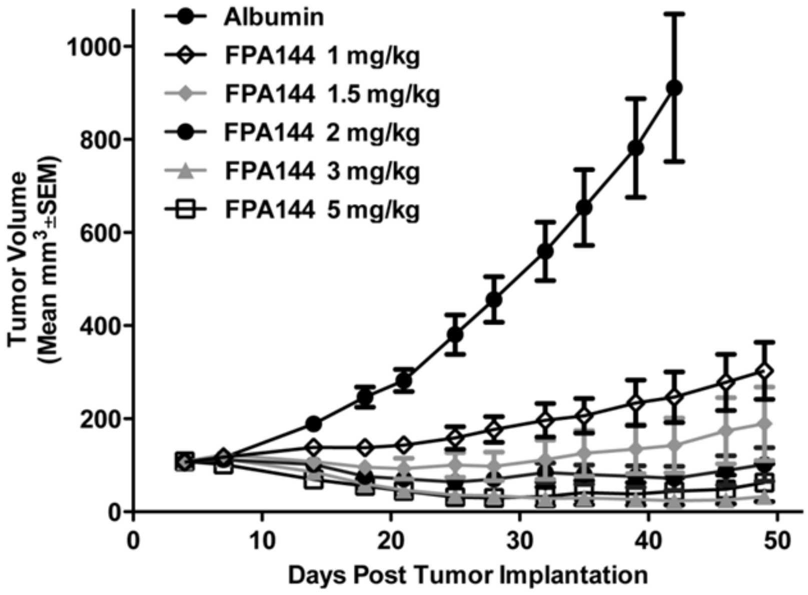

These tumor models require immunodeficient mice for tumor engraftment. Because these mice lack a fully functioning immune system, and because the mouse Fcγ receptor (the receptor on immune cells required for ADCC) has lower affinity for human antibodies than the human Fcγ receptor, ADCC is impaired in these models of FPA144 mediated tumor growth inhibition. Thus, in patients with FGFR2 overexpressing tumors, ADCC may further contribute to anti-tumor activity in the clinical setting. To understand the contribution of Fc receptor engagement and ADCC on FPA144 anti-tumor efficacy, a mutant antibody was engineered that cannot bind Fc receptors, thereby rendering it incapable of promoting ADCC. The syngeneic 4T1 model of breast cancer was employed in immune competent mice that express FGFR2b but to a lesser degree than amplified or overexpressed FGFR2b xenografts, such as OCUM-2M or SNU-16 xenograft tumors. 4T1 tumors were grown to a volume of approximately 100 mm^3, sorted into groups of equivalent tumor volume, then treated bi-weekly with FPA144, the ADCC-deficient FGFR2b antibody, or the Fc fragment of human IgG1 (hFc-G1) as control. FPA144 decreased tumor burden by 30% vs hFc-G1 control (p=0.001) while the mutant antibody showed no effect. These data support a role for ADCC activity through Fc receptor binding as a required mechanism for FPA144 efficacy in a model that expresses modest amounts of FGFR2b protein.

Mechanistically, FPA144 blocks FGFR2b phosphorylation, downregulates the receptor and inhibits downstream signaling. The effect on downstream signaling was measured by examing phosphorylation of a protein that is directly phosphorylated by the FGFR2 protein, FGF receptor substrate-2 (FRS2). This has been demonstrated in the SNU-16 *FGFR2*-amplified GC xenograft model. In this experiment, mice were treated twice weekly with 10 mg/kg FPA144. When tumors had reached approximately 500 mm^3, the animals were sacrificed and protein levels in tumors were measured via Western Blotting. FPA144 treatment resulted in decreased FGFR phosphorylation, total receptor expression, and phosphorylation of the downstream signal transduction molecule, FRS2.

In contrast to the results with *FGFR2*-amplified GC models, FPA144 has minimal impact on xenograft models that are not *FGFR2* amplified or do not express the FGFR2b protein. Mice bearing NCI-87 gastric tumors, which do not express FGFR2b, were dosed intraperitoneally twice a week with FPA144 once the tumors reached approximately 100 mm^3. The tumor growth rate was indistinguishable between animals treated with either FPA144 (5 mg/kg) or control animals administered albumin.

1.3.3.2 Analysis of Immune Effector Functions of FPA144

Some therapeutic antibodies containing IgG1 Fc are capable of recruiting immune effector function, specifically ADCC and complement-dependent cellular toxicity. Once antibodies of the IgG1 isotype bind to their target on tumor cells, immune cells which express the Fc gamma receptor IIIa (FcγRIIIa), especially NK cells and macrophages, are recruited to the tumor cells and promote cell death in a process known as ADCC. FPA144 is specifically engineered for enhanced ADCC. This antibody lacks a core fucose in the polysaccharide portion of the antibody, and the lack of fucose results in higher affinity of FPA144 for FcγRIIIa compared to the fucosylated molecule and potentially enhanced immune cell mediated tumor cell killing. In some *in vitro* studies, including ADCC assays, and *in vivo* studies, including toxicology studies, Five Prime compared the fucosylated form of FPA144 (FPA144-F) to the afucosylated form (FPA144).

FPA144 was compared to FPA144-F for *in vitro* ADCC activity. The target cell in the assay was an engineered cell line that expresses the full-length human FGFR2b described as Ba/F3 FGFR2b, and the effector cells were peripheral blood mononuclear cells (PBMCs) obtained fresh from individual human donors. As a negative control, FPA144 was also tested using a target cell line that was engineered to express the FGFR2c variant of the receptor (Ba/F3 FGFR2c cells), to which FPA144 does not bind. The data are shown in Figure 2. FPA144 and FPA144-F both showed ADCC activity, but the degree to which FPA144 killed the target cells was substantially greater than what was measured for FPA144-F. As expected, FPA144 showed no ADCC activity in the negative control.

Figure 2: *In Vitro* ADCC Activity of FPA144

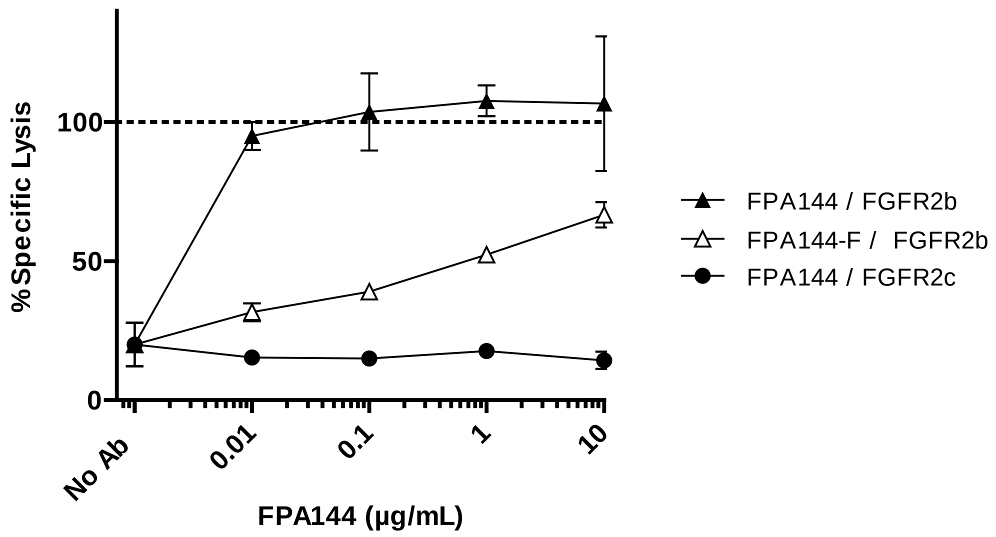

The ability of FPA144 to mediate complement-dependent cytotoxicity (CDC) of 4 gastric cell lines with high FGFR2b was tested using previously published methods (Li 2009, Zhao 2010). No CDC was observed under any conditions tested, although positive controls (rituximab tested with RAMOS and RAJI cells) did induce CDC.

1.3.3.2.1 *In vivo* ADCC Activity of FPA144

To understand the contribution of ADCC on FPA144 anti-tumor efficacy, a mutant antibody, FPA144 N297Q, which cannot bind Fc receptors, was compared to FPA144 in the syngeneic 4T1 model that expresses FGFR2b but to a lesser degree than amplified or overexpressed FGFR2b xenografts, such as OCUM-2M or SNU-16 xenograft tumors. 4T1 tumors were grown to a volume of approximately 100 mm3, then treated with FPA144, FPA144 N297Q, or the Fc fragment of human IgG1 (hFc-IgG1) as control. FPA144 decreased tumor burden vs hFc-IgG1 control while FPA144 N297Q showed no effect (Figure 3). These data support a role for ADCC activity through Fc receptor binding as a required mechanism for FPA144 efficacy in a model that expresses modest amounts of FGFR2b protein.

Figure 3: FPA144 but not an ADCC-Deficient FGFR2b Antibody Leads to Tumor Suppression in a Syngeneic Tumor Model with Modest FGFR2b Expression

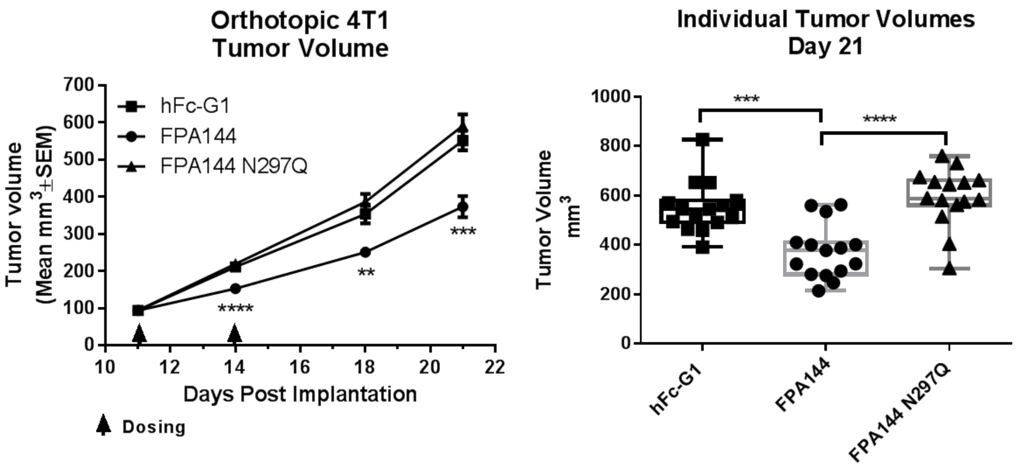

1.3.3.3 FPA144 Exposure Efficacy Relationships

To translate the efficacy results in animal models to cancer patients, the relationship between FPA144 trough concentrations and efficacy in animal models was examined. Intraperitoneal FPA144 doses of 1 mg/kg twice weekly were associated with tumor growth inhibition while greater efficacy depicted by tumor regression was noted at doses ≥ 3 mg/kg. A dose of 1 mg/kg in the mouse xenograft model led to steady state trough plasma concentrations of about 1 μg/ml, while 3 mg/kg resulted in significant tumor regression and trough plasma concentrations of 67 to 109 μg/mL.

1.3.4 Toxicology

Toxicology studies with FPA144 have been performed in rat and cynomolgus monkey. The studies performed have included pilot single dose PK/tolerability and repeat-dose studies as well as Good Laboratory Practices (GLP) repeat-dose studies. The longest of these studies involved intravenous (IV) administration of 13 weekly doses in rats and monkeys.

In pilot repeat-dose toxicology studies, rats and cynomolgus monkeys received 4 weekly IV doses of FPA144 up to 150 mg/kg. There were no changes in clinical signs and symptoms or clinical chemistry. The most significant findings from these repeat-dose pilot studies were microscopic findings in corneal epithelium. FPA144-treated animals displayed a dose-dependent thinning that represents both attenuation and reduction in the number of cells present in the corneal epithelium. In addition, microscopic changes in the retinal pigment epithelium (RPE) in rat were noted that included RPE atrophy in one high-dose animal that received 4 weekly 150 mg/kg doses. Retinal changes were not observed in the 13-week GLP toxicology studies with a high dose of 100 mg/kg.

In the 13-week repeat-dose GLP toxicology studies, FPA144 was administered by IV at dose levels of 1, 5, or 100 mg/kg/dose to both rats and monkeys for 13 weekly doses.

In the rat, FPA144 resulted in adverse findings including: tooth (incisor) abnormalities (clinical, macroscopic, and microscopic findings) and body weight loss/lack of weight gain, which were most likely secondary to the tooth findings that necessitated early euthanasia at the 100 mg/kg/dose, ocular findings (ophthalmic and microscopic findings), and macroscopic and/or microscopic findings in the Harderian gland (not present in humans) and oral mucosa (hard palate) at 5 and 100 mg/kg/dose, and macroscopic and/or microscopic findings in the tongue at all dose levels. FGFR2 pathway signaling is known to play a critical role in maintaining the health of rat incisors but has not been found to be relevant in human dentition. FPA144-related, but non-adverse microscopic findings, were also noted in the mammary gland of animals at all dose levels. Administration of FPA144 also resulted in exacerbation of background microscopic findings in the prostate gland of males given 1, 5, and 100 mg/kg, the non-glandular stomach of animals given 5 and 100 mg/kg/dose, and the lung of animals given 100 mg/kg/dose. With the exception of FPA144-related effects on incisor teeth, some degree of recovery up to total recovery was evident for all findings at the end of recovery. The absence of FPA144-related findings in the eye (ophthalmic or microscopic findings), Harderian gland, mammary gland, and prostate gland at the end of the recovery period indicated complete reversibility of the findings in these tissues. Since all findings in the 1 mg/kg/dose group were minimal, without clinical consequences, and recoverable, the highest, non-severely toxic dose (HNSTD) in rats was determined to be 1 mg/kg/dose when given weekly for 13 weeks. The lowest dose of 1 mg/kg/dose level was associated with mean C_{max} and AUC_τ (τ=168 hours) of 27.7 µg/mL and 789 h*µg/mL, respectively, for combined sexes on Day 85 of the dosing phase.

In the 13-week repeat-dose GLP toxicology study performed in cynomolgus monkeys, FPA144 was generally well tolerated. FPA144-related effects were limited to microscopic findings of corneal atrophy (slight to moderate) in animals given 5 and 100 mg/kg/dose and mammary gland atrophy (moderate to marked severity) in females from all dose groups. These findings in the cornea and mammary gland were not associated with clinical sequelae and were not observed at the end of the recovery phase, indicating complete recovery. Therefore, based on the lack of correlative clinical findings or changes (e.g., ophthalmic findings or clinical observations) and the demonstrated reversal during a recovery period, neither finding was considered adverse. The 100-mg/kg/dose level is considered below the severely toxic dose level in monkeys for the study. This represents a > 300-fold safety factor over the proposed starting dose of 0.3 mg/kg. The highest dose of 100 mg/kg was associated with mean C_{max} and AUC_τ (τ=168 hours) values of 3,266 µg/mL and 252,787 h*µg/mL, respectively, for combined sexes on Day 85 of the dosing phase.

In addition to *in vivo* toxicology studies, a GLP-compliant tissue cross reactivity study has been performed to compare the binding of FPA144 to a panel of 36 tissues from rat, cynomolgus monkey, and human. In general, the binding pattern of FPA144 was similar among the

three species and agreed with literature reports on the expression of FGFR2b being epithelial-based.

Further details of the nonclinical program for FPA144 can be found in the Investigator's Brochure (IB), which contains comprehensive information on the investigational product.

1.3.5 Clinical Experience with FPA144

The Phase 1, first-in-human study, FPA144-001, entitled "A Phase 1 Open-Label, Dose-Finding Study Evaluating Safety and Pharmacokinetics of FPA144 in Patients with Advanced Solid Tumors" started in December 2014 and is ongoing in the US, South Korea, and Taiwan. This open-label study has been assessing the safety, PK, pharmacodynamics (PD), and preliminary efficacy of FPA144 monotherapy in patients with solid tumors. The study is comprised of three parts: a dose escalation portion in unselected solid tumor patients (Part 1a), a dose escalation portion in GC patients (Part 1b), and a dose expansion portion for patients with FGFR2b-selected tumors including FGFR2b-selected GC, and FGFR2b-selected bladder cancer (Part 2).

Two parallel dose escalations were performed: Part 1a enrolled patients with solid tumors (19 patients), and Part 1b enrolled patients with GC (8 patients) to evaluate early evidence of efficacy and PKs to support the recommended dose for the dose expansion cohort. FPA144 was well tolerated in doses up to 15 mg/kg in patients with advanced solid tumors. There were no DLTs observed during dose-escalation and an MTD was not reached. Based on an assessment of safety, tolerability, and PK, an RD of 15 mg/kg was selected.

As of 20 March 2017, a total of 64 patients across the dose escalation (parts 1a and 1b: 27 patients) and the dose expansion (part 2; 37 patients) have enrolled in the study and 60 have received at least 1 dose of FPA144. Of these 64 patients, 41 patients had GC and 21 of those were identified as having GC with strong FGFR2b overexpression (or FGFR2b^{+} high, defined as IHC 3+ intensity in ≥ 10% of tumor cells). Of those 21 patients, 6 patients were enrolled in the Part 1b dose escalation and 15 patients in (Part 2) the dose expansion (Cohort A). In addition, 4 GC patients with low FGFR2b overexpression (or FGFR2b^{+} low, defined as IHC 2+ intensity in < 10% of tumor cells or any IHC 1+ staining) have been enrolled into Cohort E and 10 GC patients (IHC 0 – 2) have been enrolled into Cohort C in Part 2 (dose expansion).

Safety and tolerability of FPA144 is supported by a total of 64 patients from the Phase 1 study (FPA144-001) who have received at least 1 dose of FPA144.

Safety data from 64 patients enrolled in FPA144-001 including the 15-mg/kg expansion dose are described here. AEs have been reported in 58 of 64 patients (90.6%). Thirty-two of the 64 patients reported an AE that was deemed by the investigator to be drug related. Of the drug-related AEs, none were Grade 4 or 5, There were 6 Grade 3 events: an infusion reaction in 1 patient, an aspartate transaminase (AST) elevation and alkaline phosphatase increase in 1 patient, nausea in 2 patients and a transient decrease in neutrophil count (which resolved without dose interruption or modification) in 1 patient. Two patients discontinued treatment due

to an AE: one for E. coli sepsis and a second for cancer pain both considered unrelated to study treatment. A third patient (post data cut) with limbic stem cell deficiency considered related to study treatment also discontinued treatment. All other patients discontinued treatment as a result of disease progression. Treatment-related serious adverse events (SAEs) were reported in 3 patients; 1 patient (15 mg/kg) had a Grade 3 infusion reaction, one patient (15 mg/kg) had a Grade 2 corneal ulcer (also described earlier), and a third patient (10 mg/kg) had Grade 2 limbic stem cell deficiency. The patient with the infusion reaction resumed drug administration after premedication.

As discussed in Section 1.3.4, the preclinical animal toxicity studies supported a need for comprehensive ophthalmologic examinations. The comprehensive ophthalmologic examinations in the ongoing Phase 1 study (FPA144-001) include fundoscopic and slit lamp exam, ocular coherence tomography (OCT), visual acuity, and review of ocular and visual symptoms at Screening, prior to Cycle 2 Day 1, and at the end-of-treatment (EOT) visit. Slit lamp examinations (with completion of fluorescein staining score form), should be conducted for all patients every 8 weeks from Cycle 3 Day 1 through study completion. The comprehensive ophthalmologic examination is repeated at any time the patient develops new visual or ocular symptoms or reports changes in visual acuity.

As of 20 March 2017, there have been no reported adverse events (AEs) of any grade related to the retina. Eye disorders as an AE were reported in 15 patients. There was 1 reported SAE of Grade 2 corneal ulcer which was symptomatic and resolved with topical antibiotic treatment and FPA144 dosing interruption. The patient missed 1 dose of FPA144 and was subsequently able to resume dosing. Other treatment-emergent Grade 2 AEs that fall under the System/Organ/Class of eye disorders were 2 patients with dry eye, and one patient with increased lacrimation, cataract and blepharitis. The remaining eye disorder AEs were all Grade 1. After 20 March 2017, an event of Grade 2 limbic stem cell deficiency was reported by the investigator as related to FPA144. Study treatment administration was discontinued in this patient and during follow-up there was symptomatic improvement (downgraded to Grade 1).

Evidence of early efficacy in the $FGFR2b^{+}$ high gastric and GEJ cancer patient population is supported by a confirmed response rate (per Response Evaluation Criteria in Solid Tumors [RECIST] 1.1) of 19.0% [5.4-41.9%]) in 21 patients in the target patient population with a duration of response of 12.6 weeks (95% CI 9.1, 19.1 weeks) and a median progression-free survival (PFS) of 11 weeks (95% CI 5.7, 20.6 weeks).

FPA144 serum concentration was measured by a validated enzyme linked immunosorbent assay (ELISA) and the serum concentration versus time data (group mean ± SD) from Cycle 1 Dose 1 for patients in Phase 1 (total of 24 patients) (see the FPA144 Investigator Brochure [IB]). FPA144 demonstrated nonlinear clearance from 0.3 mg/kg to 1 mg/kg and close to linear clearance from 1 mg/kg to 15 mg/kg in patients with solid tumors including GCs tested in Parts 1A and 1B, suggesting target-mediated clearance. The estimated half-life ranged from 6.01 to 11.7 days across cohorts, which supports every 2-week dosing.

As derived from the mouse efficacy study using the OCUM2 *FGFR2*-amplified GC xenograft model, 60 µg/ml was selected as target trough serum concentration at steady state ($C_{trough\ ss}$). Based on the PK data in combination with safety data and evidence of efficacious activity from Part 1 of the clinical study, the 15-mg/kg every 2-week dose was selected to test in the Part 2 expansion cohorts as this dose level was expected to achieve target trough concentration at steady state ($C_{trough\ ss}$) of ≥ 60 µg/mL. Limited PK data in Part 2 from a total of 18 GC patients dosed at 15 mg/kg every 2 weeks, including 8 patients with high FGFR2b overexpression (Cohort A) and 10 patients without FGFR2b overexpression defined as IHC 0 (Cohort C), suggest that a recommended dose of 15 mg/kg every 2 weeks will achieve $C_{trough\ ss}$ target of ≥ 60 µg/mL (see the FPA144 IB).

1.4 Rationale for mFOLFOX6

Chemotherapy for advanced GC is widely used since it prolongs survival and improves symptoms (Okines 2009, Wagner 2006, Waddell 2014, Kang 2009, Al-Batran 2008). The combination of a platinum agent with a fluoropyrimidine has become the most frequently used combination (Kang 2009) and in a recent meta-analysis has been identified as superior to single agent treatment and best supportive care (Wagner 2006). Generally, patients with advanced GC receive either 5-fluorouracil (5-FU) or capecitabine as the main chemotherapeutic agent combined with other therapies (most frequently a platinum agent) based on improved clinical outcomes with 5-FU combination chemotherapies (Keam 2008, Kang 2009, Okines 2009, Al-Batran 2008, Wagner 2006).

The antitumor treatment effect of 5-FU is believed to result from inhibiting the enzyme thymidylate synthase (TS). Polymorphisms in TS are theorized to have a role in TS mRNA transcription, stability, or protein expression (Keam 2008) and certain polymorphisms in the enhancer region have been reported to have an effect on the effectiveness and toxicity of 5-FU (Ulrich 2000, Pullarkat 2001, Chen 2003). Leucovorin, also known as folinic acid, is known to cause a biochemical modulation of 5-FU and has been proven in several studies to enhance the treatment effect of 5-FU in patients with GC (Kim 2003).

Oxaliplatin is a cisplatin analog that functions through its ability to form DACH-platinum adducts and block deoxyribonucleic acid (DNA) replications. Oxaliplatin exhibits additive or synergistic properties when combined with 5-FU and has proven to be effective even when treating 5-FU resistant cell lines or cell lines resistant to cisplatin (Kim 2003, Keam 2008). Oxaliplatin also has been shown to have fewer toxicities and be better tolerated than cisplatin (Al-Batran 2008).

Since at least 1980, researchers have observed improved response rates and survival from using 5-FU in combination with other chemotherapeutic agents (Koizumi 2007). A number of chemotherapy combinations have been proposed for the treatment of advanced GC, including FAM (5-FU, 3 doxorubicin, and mitomycin C) (MacDonald 1980), FAMTX (5-FU/ doxorubicin [Adriamycin]/methotrexate [MTX]) (Wils 1991), EAP (etoposide/doxorubicin/ cisplatin) (Kelsen

1992), FP (5-FU/cisplatin) (Kim 1993), ECF (epirubicin/cisplatin/5-FU) (Webb 1997), and ELF (etoposide/leucovorin [LV]/5-FU) (Vanhoefer 2000).

In one trial, a combination of 5-FU with cisplatin was determined to be significantly more effective than 5-FU alone but with significant toxicity (Wohrer 2004, Kim 1993). Phase 2 trials have successfully substituted oxaliplatin for cisplatin with improved tolerability (Louvet 2002, Al-Batran 2004, Chao 2004, De Vita 2005, Lordick 2005). Infusional 5-FU, leucovorin, and oxaliplatin (mFOLFOX6) is an approved chemotherapy regimen and is one of the treatment regimens accepted for first-line treatment of metastatic GC (see Section 1.4). A randomized Phase 3 trial comparing mFOLFOX6 with 5-FU/LV/cisplatin (FLP) in the treatment of 220 patients with GC reported a statistically insignificant improved time-to-progression; however, mFOLFOX6 was associated with meaningful reductions in Grade 3/4 AEs, including neutropenia, anemia, and peripheral neuropathy (Al-Batran 2008). Subsequent studies have confirmed the safety and efficacy of mFOLFOX6 as the first line of treatment for advanced GC (Keam 2008).

mFOLFOX6 is used as standard in advanced/metastatic GC patients in the US, EU and Asia.

1.5 Rationale for Combination Therapy: FPA144 and mFOLFOX6

Since advanced GC cannot be cured with the currently available chemotherapy regimens, there is a continued need to provide improvement to available treatments. The FGFR pathway has been shown to play an important role in the transformation and proliferation of tumor cells, and inhibiting FGF ligand-stimulated FGFR2b phosphorylation and cell proliferation has been shown to reduce tumor growth in both FGFR2b overexpressing gastric and breast cell lines and xenograft models. Preliminary data from the first-in-human study of FPA144 indicate that FPA144 monotherapy has activity in patients with FGFR2b-selected GC. The safety profile has been tolerable, with no dose-limiting toxicities encountered to date.

FPA144 as monotherapy has demonstrated objective tumor responses in preclinical studies, blocking ligand binding and downstream signaling, decreasing expression of the FGFR2b driver protein and enhancing ADCC. The combination of FPA144 and paclitaxel demonstrated enhanced activity in both the OCUM-2M and HSC-39 xenograft models of GC compared to monotherapy of either agent at the doses tested. Using what is considered a more aggressive chemotherapy regimen in these same models, such as cisplatin and 5-FU or oxaliplatin and 5-FU chemotherapy, provided near complete growth suppression in the HSC-39 model and therefore no additional benefit was observed with the addition of FPA144. Furthermore, the OCUM-2M *FGFR2*-amplified model, demonstrated near complete tumor suppression with FPA144 at the doses tested, and therefore no additional benefit was observed in combination with cisplatin + 5-FU or FOLFOX chemotherapy. Importantly, the addition of FPA144 to any of these chemotherapy regimens did not increase toxicity associated with chemotherapy as measured by weight loss in mice.

Advanced stage GC has been demonstrated to be heterogenous, and the development of additional heterogeneity is hypothesized to be induced by standard front line chemotherapy (Smyth 2016). Specifically, GC metastases are more likely to overexpress FGFR2b compared to the primary tumor, suggesting that emergence of an FGFR2b overexpressing tumor is a later stage event (Ahn 2016), and may even be induced by prior systemic chemotherapy. Sequential biopsies and ctDNA testing have demonstrated that subclones of tumors with different phenotype emerge after targeted therapy treatment (Catenacci 2017, Smyth 2016). The hypothesis that chemotherapy and FPA144 target different subclones of GC is supported by the ability of FPA144 or mFOLFOX6 to drive complete growth suppression of GC tumors in distinct xenograft models supports (data on file). mFOLFOX6 and FPA144 are not anticipated to have overlapping toxicities and the combination is hypothesized to provide additional clinical benefit to patients without increasing toxicity in those patients whose tumors overexpress FGFR2b.

Further, the hypothesis that adding a targeted biologic agent to standard chemotherapy in GC may be beneficial in selected patients is further supported by the demonstration of the clinical benefit (PFS and OS) of adding trastuzumab to chemotherapy in patients with GC whose tumors overexpress HER2 (Bang 2010) and the addition of ramucirumab to paclitaxel in patients who progressed after front-line therapy (Wilke 2014).

1.6 FPA144 and mFOLFOX6 Starting Dose Justification

In study FPA144-001, no DLTs were reported at doses tested from 0.3 mg/kg to 15 mg/kg and no maximum tolerated dose (MTD) of FPA144 was identified. Therefore, the recommended dose for expansion was based on the observation of clinical efficacy and tolerability in the human FPA144001 Phase 1 study, combined with preclinical data from a OCUM2 *FGFR2*-amplified GC xenograft mouse model, which identified 60 µg/mL as the target trough serum concentration to achieve maximum efficacy. Supporting the hypothesis that 60 µg/mL should be the target C_{trough}, all patients who demonstrated a partial response in the ongoing FPA144-001 study achieved the target $C_{trough\ ss}$ of ≥ 60 µg/mL. Patients receiving a lower dose of 10 mg/kg every 2 weeks also eventually achieved a target trough of > 60 µg/mL, but not until the point of steady state, which did not occur until approximately 3 months after the initiation of FPA144. At the earlier time point of 28 days, only 3 of 6 patients dosed at the 10 mg/kg dose achieved the target C_{trough} of ≥ 60 µg/mL compared to 21/29 dosed at 15mg/kg every 2 weeks based on data cut on 20 March 2017. Due to the rapid progression and aggressiveness of gastric cancer and the lack of toxicity observed at the higher 15 mg/kg dose, the recommendation is to use the dose which achieves target trough levels as soon as possible.

No drug-drug interactions are expected between FPA144 and mFOLFOX6 based on known mechanisms of clearance. Additionally, no overlapping clinical toxicities are expected. Therefore, the expected dose of FPA144 when combined with mFOLFOX6 chemotherapy is the same as the monotherapy dose of 15 mg/kg administered every 2 weeks. The Phase 1 part of this study will initiate FPA144 combined with mFOLFOX6 at a dose 33% lower than the predicted Phase 3 dose; 10 mg/kg administered every 2 weeks. Assuming the dose of 10 mg/kg is

demonstrated to be safe, tolerable and without clinical or pharmacologic evidence of a drug interaction, the second and final dose to be tested will be 15 mg/kg every 2 weeks. At least 6 patients will be evaluated for safety, tolerability and PK at the final recommended dose prior to initiating the Phase 3 portion of the study.

1.7 Risk-Benefit Assessment of FPA144 and mFOLFOX6

This overview is not intended to replace the complete information presented in the FPA144 Investigator Brochure (IB). Please consult the IB for more detailed information.

GC is a highly lethal disease, the treatment of which depends significantly on the stage of the disease. Intensive multimodal therapy for locoregional disease fails to cure most patients and standard chemotherapy for metastatic disease provides only short-term benefits. First-line chemotherapy used in metastatic or recurrent disease generally consists of a fluoropyrimidine (5-FU or capecitabine) with a platinum agent (usually oxaliplatin or cisplatin) and the median OS is 9-10 months with a median PFS of 5-5.6 months (Kang 2009). In HER-2 positive disease, the combination of trastuzumab with standard chemotherapy (cisplatin/capecitabine or 5-FU/cisplatin) results in a median overall survival of 13.8 months and median PFS of 6.7 months compared with an overall survival 11.1 months and median PFS of 5.5 months for patients treated with chemotherapy alone (Bang 2010).

Since patients with GC have a poor prognosis, only about 50% of patients with metastatic GC worldwide receive second-line therapy (Kanagavel 2015). Second-line chemotherapy options (Waddell 2014, Hironaka 2013) include the combination of paclitaxel and ramucirumab (Wilke 2014), or single agents such as ramucirumab, irinotecan, docetaxel or paclitaxel. Median OS for second line therapy ranges between 2.4 and 4.7 months (Thuss-Patience 2011, Ford 2014, Li 2016). Recently, checkpoint inhibitors have been reported to have objective responses ranging from 6 to 26% and median OS ranging between 4.8 and 12.7 months (Goode 2016). Patients with GC and *FGFR2* amplification (Seo 2016) or FGFR2 overexpression (Ahn 2016) have a worse prognosis (Hattori 1996, Gemo 2014) thus warranting evaluation of a new targeted agent.

FOLFOX chemotherapy is associated with myelosuppression, most frequently Grade 3-4 neutropenia reported in about 40-50% of patients, peripheral neuropathy (> Grade 2) reported in about 70% of patients, diarrhea (> Grade 2) reported in about 25% of patients and nausea/vomiting reported in about 3% of patients each (Tournigand 2004, van Hazel 2016). These toxicities would not overlap with FPA144.

The individual agents used in the FOLFOX regimen (5-FU, oxaliplatin and leucovorin) are also associated with risks. Refer to local prescribing information for complete details.

The risks associated with fluororuacil include: diarrhea, nausea and vomiting, mouth sores, poor appetite, watery eyes, taste changes, discoloration of the vein and low blood counts, skin reactions, hair thinning, nail changes, hand foot syndrome, chest pain and electrocardiographic changes. Due to a potential drug-drug interaction between 5-FU and warfarin, there is a risk of

an elevated INR for patients on warfarin who also receive 5-FU (Teva Pharmaceuticals USA Inc 2016).

The risks associated with oxaliplatin include peripheral neuropathy, nausea/vomiting, diarrhea, mouth sores, low blood counts, fatigue and loss of appetite, constipation, fever, generalized pain, headache, cough, increased liver functions tests and allergic reactions.

As of April 2017, no drug has been approved in the US, EU or Asia specifically for the subset of patients with FGFR2-selected GC. Based on the emerging data from the Phase 1 FPA144-001 trial, FPA144 may provide a meaningful clinical benefit with an acceptable tolerability profile in heavily pre-treated patients with GC whose tumors overexpressed FGFR2. FGF signaling pathways appear to be a valid target for clinical investigation in human cancer based on preclinical models as well as ongoing clinical trials with other molecules that broadly target FGF signaling (Hall 2016, Taiho Oncology Inc 2015, GlaxoSmithKline 2017).

As detailed in Section 1.5, the addition of FPA144 to current standard of care first-line treatment for advanced GC with mFOLFOX6 is anticipated to improve PFS compared to chemotherapy alone. Preliminary data from the first-in-human study of FPA144 indicate that FPA144 monotherapy has activity in patients with FGFR2b-selected gastric and bladder cancer. The safety profile has been tolerable, with no dose-limiting toxicities reported.

In addition to the study design (dose escalation) and eligibility criteria that exclude patients with significant organ dysfunction, the following precautions will be taken:

- Based on non-clinical toxicology and data from the ongoing Phase 1 study, FPA144 has an expected on-target effect leading to corneal thinning which may increase the risk of developing a corneal ulcer or a secondary corneal infection. Accordingly, patients who at baseline have a history of corneal disease such as keratitis or corneal transplant will be excluded. Patients found to have certain ocular abnormalities during screening will also be excluded (corneal defects, corneal ulcerations, keratoconus, or other abnormalities that can pose an increased risk of developing an ulcer). Ophthalmology examinations will be performed at baseline and at regular protocol required intervals during the study to monitor potential ocular effects (described in Section 6.5).
- Based on non-clinical data, FPA144 may have on-target effects on the epithelium of the oropharynx. Fluoropyrimidines have a known toxicity of mucositis (Teva Parenteral Medicines Inc 2016). Patients will undergo physical examination approximately every 14 days, including examination of the oropharynx. To date, one event of Grade 1 dry mouth has been reported in a patient during cycle 6 of FPA144 given at 3 mg/kg.

- Patients will be closely monitored for infusion-related reactions which are known potential toxicities of both oxaliplatin and FPA144. The FPA144 infusion rate may be reduced at the Investigator’s discretion based on occurrence of infusion-related reactions, such as changes in vital signs, nausea, vomiting, or other constitutional symptoms or allergic reactions occurring during infusion or up to 2 hours after cessation of the infusion.
    - Routine premedication is not generally administered for the initial FPA144 dose; patients who develop infusion-related AEs may be pre-medicated prior to subsequent infusions of FPA144 at the discretion of the Investigator. In this study, since patients will also be receiving mFOLFOX6, pre-medication should be at the discretion of the Investigator. Pre-medication should be administered according to the institution’s standard practice, and should be captured on the patient’s eCRF.
    - Epinephrine for subcutaneous injection, diphenhydramine for IV injection, and any other medications and resuscitation equipment for emergency management of anaphylactic reactions must be available in the room where the infusions are being performed.

1.8 Rationale for Phase 3 Pre-screening

FPA144 is an antibody designed to recognize the FGFR2 receptor when expressed on gastric tumors. The current hypothesis is that the presence of FGFR2 will be an important predictor of how patients with FGFR2-selected gastric or gastroesophageal cancer will respond to treatment with FPA144. This is based on the preclinical observation that only tumors that overexpressed FGFR2b responded to FPA144 treatment in xenograft studies, as well as early results from the ongoing first-in-human study demonstrating objective responses in patients with FGFR2b overexpression.

Eligibility for enrollment in the Phase 3 portion is based on FGFR2 overexpression **and/or** *FGFR2* amplification as determined by a centrally performed, validated IHC or ctDNA blood assay. Patients who are positive for FGFR2 overexpression and/or*FGFR2* amplification are referred to in this protocol as FGFR2-selected. Positivity based on only one assay is adequate to meet eligibility requirements (e.g., positive by ctDNA blood assay, but negative by IHC) (see Table 2).

Patients must sign a Pre-screening ICF for collection of blood and tissue to determine FGFR2 positivity using ctDNA and IHC assays. Only patients determined to be FGFR2-selected are eligible to sign the Screening ICF and enter the Screening Period. Enrollment in the study requires achieving all other eligibility criteria (see Section 4.2).

Eligible patients for the Phase 3 portion must be naïve to prior chemotherapy for metastatic or unresectable disease, with the exception that patients may have received prior adjuvant or neo-adjuvant therapy (chemotherapy and/or chemoradiation) greater than 6 months prior to enrollment.

Since the IHC and ctDNA blood results may require approximately 2 weeks to complete, patients eligible for entering Phase 3 of this study are allowed to receive one dose of a local standard of care platinum and 5-FU combination during this interim time period (Pre-Screening Period) at the discretion of the Investigator. This one dose of chemotherapy is not a requirement of the study and is not considered part of this clinical study.

1.9 Rationale for Tumor Tissue and Blood Assessments

Patients in Phase 3 of this trial are required to submit both a tissue sample and a blood sample to test for FGFR2 and patients unable to provide both samples will not be eligible for this trial. Patients who do not demonstrate either FGFR2 overexpression using IHC or *FGFR2* amplification using a ctDNA blood assay will not be eligible for enrollment. Positivity based on either one or both assays is adequate to meet the eligibility requirements (e.g., positive by ctDNA blood assay, but negative by IHC). The blood test will reveal DNA amplification of *FGFR2*, while the IHC test will show the extent of protein expression. Five Prime has developed an anti-FGFR2 antibody for nonclinical use, whose sensitivity and specificity to detect FGFR2 by IHC has been optimized (Deshpande 2014).

In studies evaluating GC samples, *FGFR2* amplification has been uniformly associated with significant FGFR2b surface expression, as detected by IHC (Gemo 2014). The antitumor effect of FPA144 that was observed in preclinical testing was predicated upon the overexpression of FGFR2b in the tumor cell lines. Patients without overexpression of FGFR2 are unlikely to see a significant benefit from treatment with FPA144 in combination with mFOLFOX6.

The selection of patients with FGFR2-positive tumors for treatment with FPA144 is supported by data from the ongoing Phase 1, first-in-human study of FPA144-001. This open-label study is assessing the safety, PK, PD, and preliminary efficacy of FPA144 in patients with solid tumors. The study is comprised of 3 parts: dose escalation in patients with unselected solid tumors (Part 1A), dose escalation in patients with GC (Part 1B), and dose expansion in patients with GC and other FGFR2b-selected solid tumors (Part 2).

As of 20 March 2017, a total of 64 patients across Part 1 and Part 2 had enrolled in the study and received at least 1 dose of FPA144. Of the 64 patients, 41 patients had GC and 21 patients were identified as having GC with strong FGFR2b overexpression, defined in the study as IHC 3+ ≥ 10% tumor membrane staining. Of those 21 patients, 6 patients were enrolled in the Part 1B dose escalation and 15 patients were enrolled in Cohort A of the Part 2 dose expansion. Early evidence of efficacy in patients with strong FGFR2b expression is supported by a confirmed response rate (per Response Evaluation Criteria in Solid Tumors [RECIST] 1.1) of 19.0% [5.4-41.9%]) in 21 patients in the target patient population with a duration of response of 12.6 weeks (95% CI 9.1, 19.1 weeks) and a median PFS of 11 weeks (95% CI 5.7, 20.6 weeks). The observation of activity seen in patients having tumors with FGFR2b overexpression validates the strategy of selecting these patients for treatment with FPA144.

2 Study Objectives and Endpoints

2.1 Phase 1: Primary Objectives

- To determine the recommended dose (RD) of FPA144 in combination with a fixed dose of 5-FU, leucovorin, and oxaliplatin (mFOLFOX6) in patients with advanced GI tumors

2.2 Phase 1: Secondary Objectives

- To evaluate the safety and tolerability of FPA144 in combination with mFOLFOX6 in patients with GI tumors
- To characterize the pharmacokinetic (PK) profile of FPA144 in combination with mFOLFOX6 in patients with GI tumors
- To characterize the immunogenicity of FPA144

2.3 Phase 1: Exploratory Objectives

- To characterize the pharmacodynamic (PD) profile of FPA144 in combination with mFOLFOX6, through evaluation of exploratory biomarkers in blood samples from patients with GI tumors

2.4 Phase 3: Primary Objectives

- To compare Investigator-assessed progression-free survival (PFS) in patients with FGFR2-selected GC treated with FPA144 in combination with mFOLFOX6 to those treated with placebo and mFOLFOX6

2.5 Phase 3: Secondary Objectives

- To compare overall survival (OS) in patients with FGFR2-selected GC treated with FPA144 in combination with mFOLFOX6 to those treated with placebo and mFOLFOX6
- To compare objective response rate (ORR) in patients with FGFR2-selected GC and measurable disease treated with FPA144 in combination with mFOLFOX6 to those treated with placebo and mFOLFOX6
- To evaluate the safety and tolerability of FPA144 in combination with mFOLFOX6 compared to placebo and mFOLFOX6 in patients with FGFR2-selected GC
- To characterize PK of FPA144 in combination with mFOLFOX6 in patients with FGFR2-selected GC
- To characterize the immunogenicity of FPA144

2.6 Phase 3: Exploratory Objectives

- To compare blinded independent central review (BICR)-assessed PFS in patients with FGFR2- selected GC treated with FPA144 in combination with mFOLFOX6 to those treated with placebo and mFOLFOX6
- To compare one year OS of patients with FGFR2-selected GC treated with FPA144 in combination with mFOLFOX6 to those treated with placebo and mFOLFOX6

- To compare duration of response (DOR) in patients with FGFR2-selected GC treated with FPA144 in combination with mFOLFOX6 to those treated with placebo and mFOLFOX6
- To assess patient reported outcomes (PROs) and quality of life (QOL) outcomes in patients with FGFR2-selected GC receiving FPA144 in combination with mFOLFOX6 compared to placebo and mFOLFOX6
- To explore the association between FGFR2 status (in tumor tissue and/or blood) with clinical outcome
- To explore the concordance between FGFR2 status in tumor tissue and *FGFR2* amplification in blood

2.7 Phase 1: Primary Study Endpoints

- The incidence of Grade 2 or higher AEs assessed as related to FPA144 by the Investigator and the incidence of clinical laboratory abnormalities defined as dose-limiting toxicities (DLTs)

2.8 Phase 1: Secondary Endpoints

- The incidence of AEs, clinical laboratory abnormalities, corneal and retinal findings, and electrocardiogram (ECG) abnormalities
- PK parameters of FPA144, such as area under serum concentration-time curve (AUC), maximum serum concentration (C_{max}), trough serum concentration (C_{trough}), clearance (CL), terminal half-life ($t_{1/2}$), volume of distribution, and accumulation ratio, to be derived from the serum concentration-time profiles when applicable
- The incidence of treatment emergent anti-FPA144 antibody response

2.9 Phase 1: Exploratory Endpoints

- PD parameters, including exploratory biomarker analysis of the FGFR pathway, in blood

2.10 Phase 3: Primary Endpoint

- PFS, defined as time from randomization until the date of radiological disease progression based on Investigator assessment (per RECIST v.1.1) or death from any cause, whichever comes first

2.11 Phase 3: Secondary Endpoints

- Overall survival (OS), defined as time from randomization until death from any cause
- Overall response rate (ORR), defined as the proportion of patients with partial or complete response (in patients with baseline measurable disease) based on Investigator assessment of tumor lesions per RECIST v1.1 (Appendix 1).
- Incidence of AEs, clinical laboratory abnormalities, corneal and retinal findings, and ECG abnormalities
- C_{max} and C_{trough} of FPA144 in combination with mFOLFOX6 when applicable

- The incidence of treatment emergent anti-FPA144 antibody response

2.12 Phase 3: Exploratory Endpoints

- PFS, defined as time from randomization until the date of radiological disease progression based on BICR assessment (per RECIST v.1.1) or death from any cause, whichever comes first
- 1-year OS, defined as the proportion of patients who are alive one year since randomization
- DOR, limited to patients with measurable disease who are responders to treatment determined by the Investigator per RECIST v1.1 and defined as the time of first response to progression or death from any cause, whichever comes first
- Functional outcomes as measured by EQ-5D-5L and the EORTC quality of life questionnaire (QLQ)-C30
- The correlation between identified FGFR2 status in tumor tissue and/or ctDNA blood assay, as determined by IHC and blood-based molecular diagnostic assay, with PFS, OS, and objective response per RECIST v1.1
- The correlation between identified FGFR2 status in tumor tissue by IHC and FGFR2 amplification as determined by ctDNA blood assay

3 Overall Design and Plan of the Study

3.1 Study Overview

This is a multicenter study to evaluate the safety, tolerability, efficacy, PK, and PD of FPA144 in combination with mFOLFOX6. The study will include an open-label, Phase 1 safety run-in in patients with GI tumors (not FGFR2 selected) followed by a randomized, double-blind, placebo-controlled, Phase 3 portion in patients with FGFR2-selected GC (as determined by prospective IHC analysis of FGFR2b overexpression and/or a ctDNA blood assay demonstrating *FGFR2* amplification). After an initial screening period, patients will be treated with mFOLFOX6 in combination with FPA144 or placebo (IP).

3.1.1 Phase 1

Phase 1 consists of a minimum of 2 planned dosing cohorts of FPA144 in combination with mFOLFOX6 in eligible patients with advanced GI tumors to determine the RD of FPA144 in combination with mFOLFOX6.

Each patient enrolled into Phase 1 will be observed for 28 days starting on the first day of treatment with FPA144, for safety assessments, PK, and occurrence of dose-limiting toxicities (DLT Period). Upon completion of the DLT Period, patients may continue to receive FPA144 in combination with mFOLFOX6 at the Investigator's discretion. Additional treatments may be administered every 14 days (1 cycle) until Investigator-assessed radiographic or clinical disease progression, unacceptable toxicity, or until the patient meets any of the other protocol-specified withdrawal criteria. There is no maximum number of doses of FPA144 or mFOLFOX6. Ongoing

administration of the mFOLFOX6 regimen beyond the DLT Period will be according to local standard of care.

3.1.2 Phase 3

Enrollment into Phase 3 will begin when an RD for FPA144, which will not exceed 15 mg/kg, has been identified by the CRC in Phase 1. Opening the Phase 3 portion of the study for enrollment will be at the discretion of the Sponsor.

Phase 3 is randomized, double-blind, and placebo-controlled. Approximately 360 FGFR2-selected GC patients will be randomized 1:1 to be treated with the RD of FPA144 in combination with mFOLFOX6 or placebo in combination with mFOLFOX6 once every 14 days. Patients will be enrolled into either Phase 1 or Phase 3 of the study, but not both.

Eligibility for enrollment requires patients to have unresectable locally advanced or metastatic GC, be candidates for mFOLFOX6 chemotherapy as standard first line therapy, and have a tumor that is FGFR2 positive by either a centrally performed IHC test and/or a centrally performed ctDNA blood assay. A Pre-Screening ICF must be signed by the patients prior to submission of tissue (archival or fresh) and a blood sample. As receiving results of the centralized FGFR2 testing may take approximately 2 weeks, patients are allowed to receive one dose of a local standard of care platinum and 5-FU combination during this interim time period (Pre-Screening Period) at the discretion of the Investigator. This one dose of chemotherapy is not a requirement of the study and is not considered part of this clinical study.

Patients whose tumors test positive for FGFR2b by IHC and/or positive for *FGFR2* amplification by blood may consent to full study participation and subsequently undergo screening procedures, including a full ophthalmologic examination, to ensure the eligibility criteria are met. Baseline radiographic imaging is also a study requirement. Imaging performed as standard of care if it includes imaging of chest, abdomen and pelvis may be used if it has been performed within 28 days of treatment. The period of time between signing the full study ICF and Enrollment into the study is considered the Screening Period (up to 21 days).

3.2 Study Screening (Phase 1 and Phase 3) and Study Prescreening (Phase 3)

3.2.1 Phase 1

The Screening Period for the Phase 1 portion of the study (up to 28 days) begins when patients sign the ICF. All patients will undergo screening assessments within 28 days prior to the first dose of FPA144. Refer to the Schedule of Assessments (Appendix 2) for specifics of the screening assessments required prior to enrollment. Patients may have initiated or received mFOLFOX6 chemotherapy prior to enrollment into Phase 1, but eligibility requires that the patient be a candidate to receive at least 2 additional doses of mFOLFOX6 chemotherapy (there is no upper limit on the number of previous mFOLFOX6 doses that patients may have received, nor is there a requirement for prior treatment with mFOLFOX6 in Phase 1).

3.2.2 Phase 3

The description of testing for FGFR2 positivity are described in Section 1.8. Eligibility for Phase 3 will be evaluated in 2 steps:

Step 1: Patients will sign a Pre-Screening ICF to allow testing for FGFR2 status by archival or fresh tissue with IHC and a blood sample for ctDNA. The period of time between signing the Pre-Screening ICF and site notification of the result of the test is considered the Pre-Screening Period (also see Section 1.8).

Step 2: Once the site is notified of the results of the tissue and/or the blood, only patients who are positive for FGFR2 testing may be given the opportunity to consent to full study participation and enter the Screening Period. The time between signing the full study ICF and enrollment is considered the Screening Period. Patients who test negative for FGFR2b will be considered Pre-Screen-Failed. Patients who test positive and enter the Screening Period, but do not enroll, will be considered Screen-Failed.

3.3 Study Enrollment

3.3.1 Phase 1

Phase 1 is an open-label dose-escalation safety run-in. There is no randomization in this phase. Patients who are determined to be eligible will be enrolled sequentially. For patients participating in Phase 1, the date of enrollment is considered to be the date of first administration of study treatment.

3.3.2 Phase 3

Patients who meet eligibility will be randomized 1:1 to FPA144 in combination with mFOLFOX6 or placebo in combination with mFOLFOX6. For patients participating in Phase 3, the date of randomization is considered to be the date of enrollment. Patients must receive first administration of study treatment within 3 days of randomization. The date of first administration of study treatment is Cycle 1, Day 1 or Study Day 1.

3.4 Phase 1 Study Treatment Period

Patients enrolled into Phase 1 will be treated with escalating doses of FPA144 in combination with a fixed-dose chemotherapy regimen of mFOLFOX6 in 14-day cycles, as follows:

FPA144 Administration:

FPA144 IV is administered every 14 days on Day 1 of each cycle (1 dose = 1 cycle) prior to administration of mFOLFOX6 chemotherapy. FPA144 will be administered as an approximately 30-minute IV infusion via a peripheral vein or central venous catheter. The IV administration set for FPA144 infusion must contain a 0.22 µm in-line filter or a 0.22 µm syringe filter.

mFOLFOX6 Administration:

Administration of mFOLFOX6 chemotherapy will also commence on Cycle 1 Day 1 (Study Day 1) of each treatment cycle 30 minutes after the end of the infusion of FPA144. mFOLFOX6 is administered every 14 days as follows:

- Day 1: Oxaliplatin 85 mg/m^2 IV infusion over 120 minutes,
- Day 1: Leucovorin 400 mg/m^2 IV infusion over 120 minutes, can be administered concurrently with oxaliplatin if use a Y connector,
- Day 1: Immediately after oxaliplatin and leucovorin, 5 FU 400 mg/m^2 bolus over approximately 5 minutes

Day 1: Immediately after the 5-FU bolus, 5-FU 2400 mg/m^2 as a continuous IV infusion over 46 hours. After the first dose, patients may receive dose reductions, delay, or discontinuation based on toxicity as per guidelines in Section 5.4.4. These guidelines may be superceded by local standard of care.

Premedication with anti-emetics may be used at the discretion of the Investigator per local standard of care.

Dose Levels (Phase 1)

In Phase 1, the first dose cohort of FPA144 to be tested is at 10 mg/kg in a modified 3+3 dose escalation design. The anticipated dose levels are:

Dose Level 1	10 mg/kg FPA144
Dose Level 2	15 mg/kg FPA144
Dose Level -1	*6 mg/kg FPA144*<br>*(only if dose reduction is required from Dose Level 1)*

If the first cohort at 10 mg/kg clears the 28-day DLT period, the second dose cohort at 15 mg/kg will open to enroll 6 subjects. Dose escalation decisions will be based on an assessment of DLTs, overall safety, and tolerability, and will be made after the last patient enrolled in each cohort has completed the 28-day DLT Period (completion of 2 treatment cycles of FPA144 and mFOLFOX6). Dose escalation decisions will be agreed upon by the Cohort Review Committee (CRC), consisting of the Sponsor and Investigators. Review of safety and PK parameters may

inform decisions to add cohorts with alternative dose levels to reach an optimal target exposure. Dose Level -1 will only be enrolled if ≥ 2 DLTs are observed at Dose Level 1. DLTs are defined in Section 5.4.2.

The algorithm shown in Table 1 will be used for Phase 1 dose escalation decisions:

Table 1: Dose Escalation

Number of Patients with DLTs	Action
0/3	Open next cohort
1/3	Enroll 3 more patients in same cohort
≥ 2/3	Stop enrollment. Enter 3 more patients at dose level below, if only 3 were previously entered
1/6	Open next cohort
≥ 2/6	Stop enrollment. Enter 3 more patients at dose level below if only 3 were previously entered to demonstrate that ⩽ 1 of 6 patients experience DLT

The RD of FPA144 for Phase 3 will be identified by the CRC based on an evaluation of the overall safety, tolerability, and PK and will not exceed 15 mg/kg administered IV every 14 days. The RD, therefore, may or may not be the same as the identified maximum tolerated dose (MTD). For example, if the MTD is not reached, or if data from subsequent cycles of treatment from Phase 1 provide additional insight on the safety profile, then the RD may be a different, though not higher, dose than the MTD.

The MTD is defined as the maximum dose at which < 33% of patients experience a DLT during the DLT Period. If a DLT is observed in 1 of 3 patients at a given dose level, then 3 additional patients will be enrolled at that same dose level. Dose escalation may continue until 2 of 3 to 6 patients treated at a dose level experience a DLT (dose level not to exceed 15 mg/kg). The next lower dose will then be considered the MTD.

Upon initiation of enrollment into the 15 mg/kg cohort, 6 patients will be enrolled to explore the safety and efficacy at this one dose level. The total enrollment for Phase 1 will be approximately 9 to 12 patients.

Any patient who does not receive 2 doses of FPA144 and 2 doses of mFOLFOX6 during the DLT Period due to a reason that is not a DLT or an AE related to FPA144 will be considered unevaluable and the patient will be replaced. The replaced patient may continue on study at the Investigator's discretion and after discussion with the Sponsor. No more than 2 doses of FPA144 or 2 doses of mFOLFOX6 should be administered during the 28-day DLT Period. The second dose of FPA144 and mFOLFOX6 do not need to be synchronized (as an example; if mFOLFOX6 is delayed due to an AE that is deemed related only to mFOLFOX6 and not to

FPA144, FPA144 might be administered on Study Day 1 and 15 and mFOLFOX6 may be administered on Study Day 1 and 21).

Upon completion of the DLT Period, patients may continue receiving FPA144 in combination with mFOLFOX6 at the Investigator's discretion. Additional treatments may be administered every 14 days (1 cycle) until Investigator-assessed radiographic or clinical disease progression, unacceptable toxicity, or until the patient meets any of the other protocol-specified withdrawal criteria.

There is no mandated maximum number of doses of FPA144 or mFOLFOX6. Ongoing administration of the mFOLFOX6 regimen beyond the DLT Period will be according to Section 3.4 or be superseded by local standard of care as applicable. Initiation of a new cycle of mFOLFOX6 following a dosing delay may be synchronized with administration of an FPA144 infusion, but is not a study requirement. In the Phase 1 portion of the study, if FPA144 is permanently discontinued for any reason the patient will undergo an end of treatment (EOT) follow-up visit approximately 28 days after the last dose of FPA144. For these patients, the end of FPA144 treatment is the end of study and no further follow-up will be conducted.

If mFOLFOX6 is discontinued for any reason other than Investigator-assessed radiographic or clinical disease progression, or any of the other protocol-specified withdrawal criteria, FPA144 may be continued as a single agent therapy at the Investigator's discretion, and administered every 14 days until Investigator-assessed radiographic or clinical disease progression, unacceptable toxicity, or until the patient meets any of the other protocol-specified withdrawal criteria.

In the event a cycle of mFOLFOX6 is delayed beyond 14 days due to chemotherapy-related toxicity during the first 3 doses (cycles), FPA144 should be administered every 14 days (±3 days). After the first 3 doses, FPA144 may be delayed up to ±7 days to be synchronized with administered mFOLFOX6.

3.5 Phase 3 Study Treatment Period

Patients will be enrolled into Phase 3 with the objective of evaluating the efficacy of FPA144 combined with mFOLFOX6 compared to placebo combined with mFOLFOX6 in patients with FGFR2-selected GC. Enrollment into Phase 3 will begin when an RD for FPA144, which will not exceed 15 mg/kg, has been identified by the CRC in Phase 1. Opening Phase 3 for enrollment will be at the discretion of the Sponsor.

As detailed above, Phase 3 is randomized, double-blind, placebo-controlled, and will enroll approximately 360 patients randomized 1:1 to be treated with the RD of FPA144 in combination with mFOLFOX6 or placebo in combination with mFOLFOX6 once every 14 days. An unblinded site pharmacist will supply FPA144 or saline as the placebo (details provided in a separate Pharmacy Manual).

Treatment arms consist of:

Arm 1: FPA144 at the RD and mFOLFOX6 administered every 14 days

Or

Arm 2: Placebo and mFOLFOX6 administered every 14 days

Opening of Phase 3 for enrollment will be at the discretion of the Sponsor.

Enrolled patients may continue treatment in 14-day cycles of mFOLFOX6 and either FPA144 or placebo (hereafter referred to as Investigational Product [IP]) until Investigator-assessed radiographic disease progression, unacceptable toxicity, or until the patient meets any of the other protocol-specified withdrawal criteria. There is no protocol mandated maximum number of doses of mFOLFOX6 or IP.

Discontinuation of one component of the study (mFOLFOX6, a component of mFOLFOX6, or IP) for any reason other than disease progression, does not mandate discontinuation of other components. The exception is the discontinuation of 5-FU for any reason requires discontinuation of oxaliplatin and leucovorin.

Ongoing administration of the mFOLFOX6 regimen is recommended to be according to Section 3.5 or local standard of care if applicable. All treatment decisions will be made by the Investigator using local assessments.

The first 3 doses (cycles) of IP should be administered every 14 days (±3 days) regardless of delays in mFOLFOX6 treatment. After the first 3 doses, IP may be delayed up to 7 days to be synchronized with administered mFOLFOX6. Synchronization, however is not a protocol requirement. If after 7 days, the patient is still unable to receive mFOLFOX6, IP should continue as monotherapy every 14 days.

Patients who discontinue all study treatment (all components of mFOLFOX6 and IP) for any reason other than consent withdrawal will undergo an EOT follow-up visit approximately 28 days after the last dose of the last administered component of treatment (oxaliplatin, leucovorin, 5-FU or IP). If the patient has not progressed at the time of the EOT follow-up visit, they will then continue to undergo tumor assessments according to the protocol schedule until radiographic progression or the initiation of additional anti-cancer therapy.

In addition, patients will undergo long-term follow-up for survival by clinic visit or by telephone approximately every 3 months (± 1 month) after the EOT visit until up to 24 months after the last patient is enrolled into the study, or until death, loss to follow-up, withdrawal of consent or study termination by the Sponsor (whichever occurs first).

3.6 Procedures

Patients will undergo safety evaluations that include evaluation for DLTs and other AEs, hematology, clinical chemistry, urinalysis, vital signs, body weight, concomitant medications/procedures, Eastern Cooperative Oncology Group (ECOG) performance status, targeted physical examinations, ECGs, and ophthalmology examinations.

Assessment of AEs will follow the guidelines provided in the National Cancer Institute (NCI) Common Toxicity Criteria for Adverse Events (CTCAE), version 4.03. AEs will be assessed as outlined in Section 6.1.1.3. Abnormal laboratory results that lead to a change in patient treatment management (e.g., dose delay, requirement for additional medication or monitoring) are considered clinically significant for the purposes of this study and will be recorded on the AE page of the eCRF. Events meeting SAE criteria must be reported as SAEs (Section 6.1.1.3.1). The Investigator's determination of relationship of the AE to drug therapy and counter measures undertaken will be documented and noted on the eCRF.

Tumor assessments (required in Phase 3 only) should consist of clinical examination and appropriate imaging techniques (preferably CT scans with appropriate slice thickness per RECIST 1.1 guidelines, MRI acceptable).

PK blood samples will be collected to measure the concentration of FPA144 in serum. Immunogenicity blood samples will be collected to measure the level of anti-FPA144 antibodies in serum. Samples for PK and immunogenicity assessment will be drawn from each patient at the time points outlined in Appendix 5 (for Phase 1) and Appendix 6 (for Phase 3). These samples will be collected and processed according to the instructions provided in a separate Laboratory Manual.

Blood samples for exploratory biomarker analysis of the FGFR pathway will be collected at time points outlined in Appendix 5 (for Phase 1).

Safety blood tests are listed in Appendix 4. Local hematology and blood chemistry test results must be obtained within 96 hours of dosing to confirm eligibility. On FPA144 dosing days, hematology and blood chemistry results must be obtained within 72 hours prior to study treatment administration. Coagulation samples need to be obtained at baseline, at Cycles 1 through 4, and whenever clinically indicated (e.g., patients on anticoagulant therapy requiring close monitoring).

A negative serum pregnancy test in female patients of childbearing potential obtained within 96 hours prior to Cycle 1 Day 1 is mandatory. On dosing days of every other cycle (odd cycles), a negative urine pregnancy test must be obtained within 72 hours prior to dosing.

A complete physical examination including height and weight will be performed at Screening. Limited physical examinations should be conducted per the Schedule of Assessments

(Appendix 2 and Appendix 3 for Phase 1 and Phase 3, respectively) and include examination of the oropharynx making certain there are no mouth sores or desquamation of the oral mucosa.

Comprehensive ophthalmologic examinations include fundoscopic and slit lamp exam, ocular coherence tomography (OCT), visual acuity, completion of fluorescein staining score form, determination of intraocular pressure, and review of ocular/visual symptoms.

All patients should return to the clinic 28 days (±3 days) after their last dose of study treatment (the last administered dose of FPA144 in Phase 1 or the last administered dose of IP or any component of mFOLFOX6 for Phase 3) for EOT follow-up assessments. For patients in Phase 3 who discontinue study treatment (the last administered dose of IP and/or mFOLFOX6) for reasons other than disease progression or withdrawal of consent, radiographic tumor assessments will continue according to the protocol until the patient initiates additional anti-cancer therapy or disease progression.

Patients in Phase 3 will undergo long-term follow-up for survival by clinic visit or by telephone approximately every 3 months ± 28 days after the End of Treatment (EOT) visit until up to 24 months after the last patient is enrolled into the study, or until death, loss to follow-up, withdrawal of consent or study termination by the Sponsor (whichever occurs first), which will be considered end of study. Subsequent cancer treatments will be collected as part of the survival assessment.

3.7 Study Schema

The study schema is shown in Figure 4 (Phase 1) and Figure 5 (Phase 3).

Figure 4: Phase 1 Dose Escalation Safety Run-in

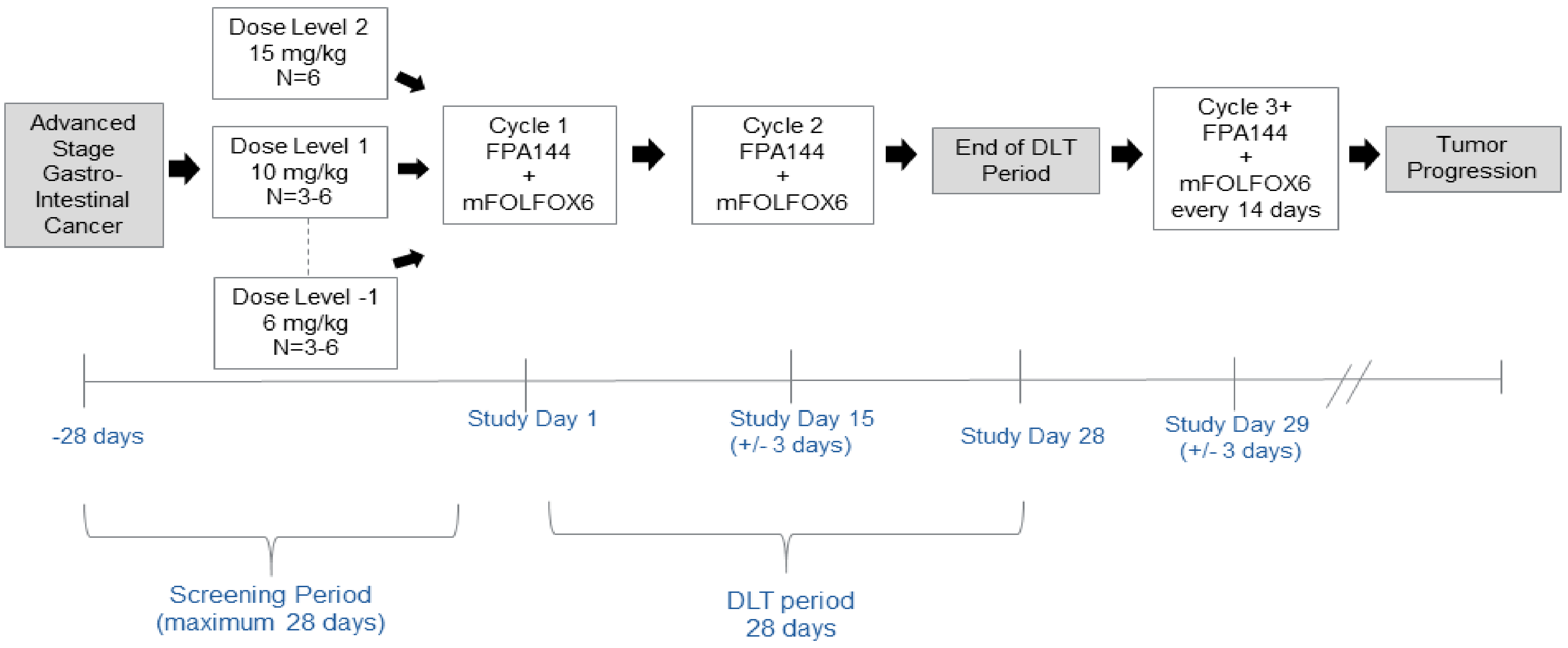

Figure 5: Phase 3 Randomized, Double-Blind, Placebo-Controlled Portion

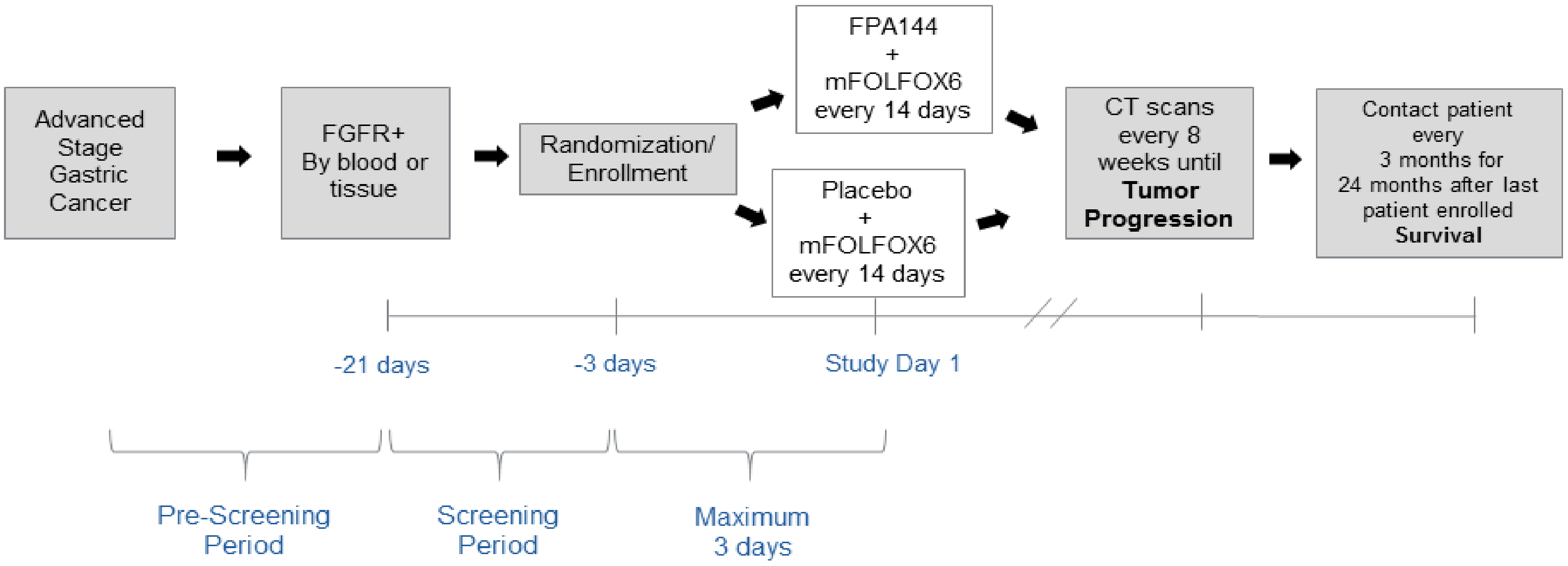

3.8 Rationale for the Study Design

Tolerability of FPA144 has been observed in the ongoing Phase 1 monotherapy study (FPA144-011). The Phase 1 open-label dose-escalation of this study (FPA144-004) will assess the safety, PK, and PD of FPA144 in combination with mFOLFOX6 in patients who have unselected GI cancer (with or without FGFR2 positive status) and will inform selection of the recommended dose of FPA144 for combination with mFOLFOX6 in the Phase 3 portion of this study.

In Phase 3, FGFR2-selected patients based on IHC and/or ctDNA blood assays will be randomized 1:1 to treat with FPA144 and mFOLFOX6 or placebo and mFOLFOX6 in 14-day cycles. The rationale for combining FPA144 and mFOLFOX6 is described in Section 1.5. The rationale for selecting patients with FGFR2 positive tumors is described in Section 1.8.

The study will measure clinically meaningful endpoints of PFS, OS and ORR that demonstrate benefit in gastric cancer patients. FPA144 will be combined with chemotherapy to potentially increase the benefit seen with FPA144 monotherapy in late line gastric cancer patients. Furthermore, the toxicities of FPA144 and FOLFOX therapy are not expected to overlap.

Selecting patients for *FGFR2 amplification* and overexpression targets patients most likely to obtain a clinical benefit from FPA144. It is hypothesized that the presence of FGFR2 will be an important predictor of how patients with FGFR2-selected gastric or gastroesophageal cancer will respond. This is based on the preclinical observation that only tumors that overexpressed FGFR2b responded to FPA144 treatment in xenograft studies, as well as early results from the ongoing first-in-human study (FPA144-001) demonstrating objective responses in patients with FGFR2b overexpression. To be eligible, patients must demonstrate either FGFR2 overexpression using IHC or *FGFR2* amplification using a ctDNA blood assay. Positivity based on either one or both assays is adequate to meet the eligibility requirements. The blood test will reveal DNA amplification of *FGFR2*, while the IHC test will show the extent of protein expression. In studies evaluating GC samples, *FGFR2* amplification has been uniformly associated with significant FGFR2b surface expression, as detected by IHC (Gemo 2014). Patients without overexpression of FGFR2 are unlikely to see a significant benefit from treatment with FPA144 and mFOLFOX6.

Including a placebo arm as an inactive control allows a better evaluation of the true overall effect of the active drug. The investigator and patient blind will be maintained by allowing the pharmacist to prepare the infusion bags of study treatment or placebo.

4 Study Eligibility and Withdrawal Criteria

4.1 Planned Number of Patients and Study Centers

In Phase 1, 2 dose cohorts of FPA144 are anticipated in a modified 3+3 dose escalation design, with a minimum of 3 patients enrolled into each cohort and a minimum of 6 enrolled at the RD. The total enrollment for Phase 1 will, therefore, be approximately 9 to 12 patients.

In Phase 3, up to 360 FGFR2-selected GC patients will be randomized 1:1 to be treated with FPA144 in combination with mFOLFOX6 or placebo in combination with mFOLFOX6 in 14-day cycles at an RD selected after assessment of data obtained in Phase 1. Opening of Phase 3 for enrollment will be at the discretion of the Sponsor.

The total enrollment planned for this study is approximately 372 patients.

The study will be conducted at up to 250 global study centers.

4.2 Inclusion Criteria for Phase 1 and Phase 3

Patients enrolling into either Phase 1 or Phase 3 of the study must meet ***all*** of the following inclusion criteria:

1) Disease that is unresectable, locally advanced, or metastatic
2) Understand and sign an Institutional Review Board (IRB)/Independent Ethics Committee (IEC)-approved informed consent form (ICF) prior to any study-specific evaluation
3) Life expectancy of at least 3 months in the opinion of the Investigator
4) Eastern Cooperative Oncology Group (ECOG) performance status of 0 to 1
5) Age ≥18 years at the time the ICF is signed
6) Negative serum β-human chorionic gonadotropin (β-hCG) pregnancy test ≤ 96 hours prior to treatment (women of childbearing potential only)
7) In sexually active patients (women of child bearing potential and males), willingness to use 2 effective methods of contraception, of which 1 must be a physical barrier method (condom, diaphragm, or cervical/vault cap) until 6 months after the last dose of FPA144. Other effective forms of contraception include:
    - Permanent sterilization (hysterectomy and/or bilateral oophorectomy, or bilateral tubal ligation with surgery, or vasectomy) at least 6 months prior to Screening
    - Women of childbearing potential who are on stable oral contraceptive therapy or intrauterine or implant device for at least 90 days prior to the study, or abstain from sexual intercourse as a way of living

8) Adequate hematological and biological function, confirmed by the following laboratory values within 96 hours of treatment (Cycle 1 Day 1).
   - Bone Marrow Function
     - Absolute neutrophil count (ANC) ≥ 1.5 × 10^9/L
     - Platelets ≥ 100 × 10^9/L
     - Hemoglobin ≥ 9 g/dL
   - Hepatic Function
     - Aspartate aminotransferase (AST) and alanine aminotransferase (ALT) < 3 × upper limit of normal (ULN); if liver metastases, then < 5 × ULN
     - Bilirubin < 1.5 × ULN
   - Renal Function
     - Calculated creatinine clearance ≥ 50 mL/min (see Appendix 1)
9) Patients on full-dose anticoagulants must be on a stable dose of warfarin and have an international normalised ratio (INR) within the therapeutic range for the patient's condition or be on a stable dose of low molecular weight heparin
10) Measurable or non-measurable disease
11) Tumor tissue for determination of FGFR2 status

Patients enrolling into **Phase 1** of the study must also meet the following inclusion criteria:

12) Histologically or cytologically confirmed GI malignancy for which mFOLFOX6 is considered an appropriate treatment (e.g., GC, colorectal carcinoma, pancreatic adenocarcinoma)
13) Patient must be a candidate for at least 2 doses of mFOLFOX6 chemotherapy

Patients enrolling into **Phase 3** of the study must also meet the following inclusion criteria:

14) Histologically documented gastric or gastroesophageal junction (defined as 5 cm proximal and distal to the GEJ) adenocarcinoma
15) Radiographic imaging of the chest, abdomen and pelvis (CT preferred, MRI acceptable) performed within 28 days of treatment (Cycle 1, Day 1)
16) FGFR2 overexpression as determined by a centrally performed IHC test and/or *FGFR2* amplification as determined by a centrally performed ctDNA blood assay
17) Patient must be a candidate for mFOLFOX6 chemotherapy

18) No prior chemotherapy for metastatic or unresectable disease (except a maximum of 1 dose of a platinum and fluoropyrimidine combination may have been administered while awaiting results of FGFR2 testing)
19) No prior platinum-based chemotherapy (except as noted in the Inclusion Criterion #18)
20) If prior adjuvant or neo-adjuvant therapy (chemotherapy and/or chemoradiation) has been received, more than 6 months must have elapsed between the end of adjuvant therapy and enrollment

4.3 Exclusion Criteria for Phase 1 and Phase 3

Patients enrolling into either Phase 1 or Phase 3 will be excluded if *any* of the following criteria apply:

1) Untreated or symptomatic central nervous system (CNS) metastases. Patients with asymptomatic CNS metastases are eligible provided they have been clinically stable for at least 4 weeks and do not require intervention such as surgery, radiation, or any corticosteroid therapy for management of symptoms related to CNS disease
2) Impaired cardiac function or clinically significant cardiac disease, including any of the following:
   - Unstable angina pectoris ≤ 6 months prior to enrollment
   - Acute myocardial infarction ≤ 6 months prior to enrollment
   - New York Heart Association class II-IV congestive heart failure
   - Uncontrolled hypertension (as defined as ≥ 160/90 despite optimal medical management)
   - Unstable cardiac arrhythmia
   - Active coronary artery disease
3) QTcF ≥ 480
4) Peripheral sensory neuropathy ≥ Common Terminology Criteria for Adverse Events (CTCAE) Grade 2
5) Active infection requiring systemic treatment or any uncontrolled infection ≤ 14 days prior to enrollment
6) Known human immunodeficiency virus (HIV) or acquired immunodeficiency syndrome (AIDS)-related illness, or known active or chronic hepatitis B or C infection
7) History of interstitial lung disease (e.g., pneumonitis or pulmonary fibrosis)
8) Evidence or history of bleeding diathesis or coagulopathy

9) Radiotherapy ≤ 28 days of enrollment. Patients must be recovered from all radiotherapy-related toxicities. No radiopharmaceuticals (strontium, samarium) within 8 weeks of enrollment

10) Prior treatment with any selective inhibitor (e.g., AZD4547, BGJ398, JNJ-42756493, BAY1179470) of the FGF-FGFR pathway

11) Ongoing adverse effects from prior treatment > NCI CTCAE Grade 1 (with the exception of Grade 2 alopecia)

12) Participation in another therapeutic clinical study or receiving any investigational agent within 28 days of enrollment or during this clinical study

13) Corneal defects, corneal ulcerations, keratitis, keratoconus, history of corneal transplant, or other known abnormalities of the cornea that may pose a risk with FPA144 treatment

14) Positive HER2 status (as defined by a positive IHC test of 3+ or IHC of 2+ with positive FISH).

15) Major surgical procedures are not allowed ≤ 28 days prior to enrollment. Surgery requiring local/epidural anesthesia must be completed at least 72 hours before enrollment. In all cases the patient must be sufficiently recovered and stable before treatment administration

16) Women who are pregnant or breastfeeding (unless the patient is willing to interrupt breastfeeding during study treatment administration and then resume 6 months after study discontinuation); women of childbearing potential must not consider getting pregnant during the study

17) Presence of any serious or unstable concomitant systemic disorder incompatible with the clinical study (e.g., substance abuse, psychiatric disturbance, or uncontrolled intercurrent illness including arterial thrombosis, and symptomatic pulmonary embolism)

18) Presence of any other condition that may increase the risk associated with study participation or may interfere with the interpretation of study results, and, in the opinion of the Investigator, would make the patient inappropriate for entry into the study

19) Known allergy or hypersensitivity to components of the FPA144 formulation, including polysorbate, or to platinum-containing medications, 5-FU, or leucovorin

20) History of prior malignancy except another malignancy that in the Investigator's opinion would not affect the determination of study treatment effect

No waivers of these inclusion or exclusion criteria will be granted.

4.4 Patient Identification and Enrollment

Patients must be able to provide written informed consent and meet all eligibility criteria prior to enrollment. No waivers of inclusion or exclusion criteria will be granted by the Investigator and Sponsor or its designee for any patient enrolled in the study. Patients who qualify for Phase 1 of

the study will be enrolled into the first available cohort. A patient may be enrolled into either Phase 1 or Phase 3 of the study, but not both.

In Phase 3, patients first undergo Pre-screening in which both a ctDNA blood assay and a tissue test are required. Tissue may be archival or fresh. Patients who are determined to be FGFR2 positive by either test may immediately enter the Screening Period and be eligible for the Phase 3 study (see Table 2).

Table 2: Eligibility Based on FGFR2 Status

**FGFR2 Amplificationa (using a ctDNA blood assay)b**	**IHC FGFR2 Overexpressiona (using a tissue-based IHC assay for FGFR2b protein)c**	**Eligibility**
Blood (+)	IHC (+)	Eligible
Blood (-)	IHC (+)	Eligible
Blood (+)	IHC (-)	Eligible
Blood (-)	IHC (-)	Ineligible

a: Both tests will be carried out at central laboratories.
b: Requires 2 x 10 mL
c: IHC: Minimum of 5 slides required. A sample is scored as positive in the presence of any membranous staining of 2+ or 3+ intensity.

In both Phase 1 and 3, the Investigator may repeat qualifying laboratory tests and vital signs/ECGs prior to enrollment if a non-qualifying finding is considered an error or an acute finding is likely to meet eligibility criteria on repeat testing. Local hematology and blood chemistry test results must be obtained within 96 hours of dosing to confirm eligibility.

4.5 Patient Withdrawal and Replacement

A patient must be discontinued from protocol-prescribed therapy if any of the following apply:

- Consent withdrawal at the request of the patient or their legally authorized representative
- Progression of patient's disease as assessed by the Investigator
- Any event that would pose an unacceptable safety risk to the patient
- A concurrent illness that would affect assessments of the clinical status to a significant degree
- A positive pregnancy test at any time during the study
- At the specific request of the Sponsor or its authorized representative (e.g., if the study is terminated for reasons of patient safety)

Patient replacement will be as follows:

- Patients in the Phase 1 Safety Run-in will be replaced if they are unevaluable for DLT
- Patients in the Phase 3 will not be replaced

5 Study Treatment

5.1 FPA144 Identity

FPA144 drug product is supplied for IV administration as a sterile, aqueous, colorless to slightly yellowish, pyrogen-free solution supplied in single-use glass vials. The composition of the drug product contains 20 mg/mL active ingredient, 20 mM L-histidine, 270 mM sucrose, 0.01% polysorbate 20 at pH 6.0. The container-closure system consists of a 20 mL Type I glass vial, sealed with a bromobutyl rubber stopper, and a flip-off cap. The final drug product will be provided as 2° to 8°C refrigerated liquid protected from light which is diluted for administration per instructions provided in a separate Pharmacy Manual.

FPA144 will be supplied in a sterile vial for dilution into an IV bag for administration by the study center.

5.2 Administration

5.2.1 mFOLFOX6

Oxaliplatin, 5-FU, and leucovorin will be administered by each site as per routine institutional practice. The mFOLFOX6 regimen will be administered every 14 days (± 3 days) until Investigator-assessed radiographic disease progression, clinical disease progression (phase 1 only), unacceptable toxicity, or the patient meets any of the other protocol-specified withdrawal criteria. Refer to the local/country specific prescribing information for preparation and complete prescribing information.

The starting dose for mFOLFOX6 includes 85 mg/m^2 of oxaliplatin, 400 mg/m^2 of calcium folinate (folinic acid), a 400 mg/m^2 bolus dose of 5-FU, and a 2400 mg/m^2 continuous infusion dose of 5-FU over 46 hours (Hochster 2008, Cheeseman 2002).

- Day 1: Oxaliplatin 85 mg/m^2 IV infusion over 120 minutes
- Day 1: Leucovorin 400 mg/m^2 IV infusion over 120 minutes, can be administered concurrently with oxaliplatin if using a Y connector
- Day 1: Immediately after oxaliplatin and leucovorin, 5 FU 400 mg/m^2 bolus over approximately 5 minutes
- Day 1, Immediately after the 5-FU bolus, 5-FU 2400 mg/m^2 as a continuous IV infusion over 46 hours

Recommended dose adjustments and delays due to toxicity are outlined in Section 5.4.4.2. Doses should be adjusted if there is a change in weight by > 10% compared to the weight at Cycle 1, Day 1.

5.2.2 Open-label FPA144 (Phase 1) and Blinded Investigational Product (IP) (Phase 3)

FPA144/IP will be administered only to patients in this study using procedures described in this protocol. The dose of FPA144/IP is based on body weight at Cycle 1 Day 1 and adjusted if the patient's weight changes > 10% from Cycle 1 Day 1.

A pharmacist (or other responsible person) will prepare FPA144/IP for administration. After calculating the number of vials based on the patient's weight, the FPA144/IP will be diluted in a 0.9% sodium chloride solution. Prepared FPA144/IP should be administered ≤8 hours after preparation (ambient temperature). FPA144/IP will be administered under medical supervision over approximately 30-minute (± 5 minutes) IV infusion via a peripheral vein or central venous catheter. The IV administration set for FPA144/IP infusion must contain a 0.22 µm in-line filter or a 0.22 µm syringe filter. For Phase 3, the pharmacist, who will be unblinded to treatment assignment, will supply FPA144 and placebo (saline bag) according to guidelines provided in a separate Pharmacy Manual.

Infusion of FPA144/IP must be stopped, reduced, interrupted, or discontinued per Sections 5.4.2 and 5.4.4. If a patient experiences an infusion reaction, the patient's vital signs (temperature, blood pressure, pulse, and respiration rate) should be monitored during the infusion as well as every 30 minutes after the infusion for a minimum of 2 hours and until resolution of the infusion reaction.

Further instructions on drug preparation and administration are in the Pharmacy Manual.

Patients may continue receiving FPA144/IP administered in 14-day cycles until Investigator-assessed radiographic disease progression (or, in Phase 1 only, until clinical progression), unacceptable toxicity, or until the patient meets any of the other protocol-specified withdrawal criteria. There is no maximum number of doses of FPA144/IP.

5.3 Packaging, Labeling, and Storage

Refer to the most current package insert for packaging, labeling, and storage information of mFOLFOX6.

FPA144 will be packaged and labeled as a 20 mL fill in ISO 20R vials by the Sponsor (or designee) per applicable local regulatory requirements.

All FPA144 vials must be stored refrigerated at 2° to 8°C in accordance with the manufacturer's instructions as provided in the Pharmacy Manual. Until dispensed to patients, FPA144 will be stored in a securely locked area, accessible to authorized personnel only.

5.4 Starting Dose and Dose Modifications

In Phase 1 the starting dose level of FPA144 in combination with a fixed dose chemotherapy regimen of mFOLFOX6 is 10 mg/kg. Subsequent dose escalations between cohorts in Phase 1 are described in Section 3.4. The dose of IP in combination with a fixed dose chemotherapy regimen of mFOLFOX6 in Phase 3 will be determined by evaluation of the data from Phase 1 of the study as described in Section 3.5.

5.4.1 Dose Escalation within a Cohort

In Phase 1, intra-patient dose escalation will not be permitted.

In Phase 3, patients will be treated at the RD as determined from Phase 1, and dose escalation will not be allowed.

5.4.2 Dose-Limiting Toxicity

DLTs are defined as any of the following events that occur during the first 28 days of treatment and are assessed by the CRC as related to FPA144. As applicable, events will be classified according to the NCI CTCAE (Version 4.03).

- ANC < 0.5 x 10^9/L > 5 days duration or febrile neutropenia (i.e., ANC < 1.0 x 10^9/L with a single temperature of >38.3°C, or fever $\geq$38 °C for more than 1 hour). Use of G-CSF is permitted per institutional standards
- Platelets <25 x 10^9/L or platelets <50 x 10^9/L with bleeding requiring medical intervention
- Prolonged (>7 days) Grade 3 thrombocytopenia
- Grade 4 anemia (i.e., life-threatening consequences; urgent intervention indicated)
- Any Grade 2 or greater ophthalmologic AE that does not resolve within 7 days
- AST/ALT >3 x ULN and concurrent total bilirubin >2 x ULN not related to liver involvement with cancer
- Any non-hematological AE Grade 3 or greater (except nausea, vomiting, and diarrhea if well controlled by systemic medication). Grade 3 or 4 laboratory values that are not of clinical significance per Investigator and Sponsor agreement will not be considered DLTs.
- Any FPA144 related AE which results in a dose reduction or delay by at least 96 hours of any component of mFOLFOX6

5.4.3 Toxicity at Lowest Dose Level

If the MTD is unexpectedly exceeded at the first dose level of FPA144 (10 mg/kg every 14 days), the dose will be reduced to 6 mg/kg every 14 days. Decisions on how to next proceed will be based on safety, tolerability, and PK data, and will be determined by the Cohort Review Committee (CRC).

Dose Level -1 (FPA144 6 mg/kg every 14 days; 3 to 6 subjects) will only be enrolled if ≥ 2 DLTs are observed at Dose Level 1.

5.4.4 Dose Modification Criteria - Open-label FPA144 (Phase 1) and Blinded IP (Phase 3)

Dose reductions for FPA144/IP may be permitted for patients on treatment beyond the DLT Period in Phase 1 or any patient in Phase 3 and can be assessed using local laboratories. If a patient in Phase 1 requires a dose reduction of FPA144 during the DLT period, they will be considered a DLT and be permanently discontinued from FPA144. Dose reductions for FPA144/IP-related AEs should follow the guidelines outlined in Table 3 (non-corneal toxicities) and Table 4 (corneal toxicities). Any patient with FPA144/IP-related retinal toxicity should permanently discontinue FPA144/IP. If dose reductions or interruptions that do not fall within these guidelines are being considered by the Investigator, these will require discussion with and approval by the Sponsor or designee. Patients may resume the FPA144/IP if the event returns to baseline or ≤ Grade 1 in accordance with the guidelines outlined in Table 3 and Table 4.

Table 3: Dose Modification Guidelines for FPA144 (Any Non-corneal, Non-infusion Toxicity[a])

FPA144/IP-related Toxicity Grade	Dose Schedule	New FPA144 Dose
Grade 1 or 2	No delay or missed dose required	100% of dose
Grade 3<br>(first occurrence)	Delay or miss dose until recovery to baseline or Grade 1	If recovery to baseline or Grade 1 within 28 days, may resume at 100% of starting dose or one dose lower[b]
Grade 3<br>(second occurrence)	Delay or miss dose until recovery to baseline or Grade 1	If recovery to baseline or Grade 1 within 28 days, may resume at one dose level lower[b] than previous dose or discontinue
Grade 3 (third occurrence)<br>Grade 3 which does not recover to baseline or Grade 1 within 28 day<br>Any Grade 4	Permanently Discontinue	N/A

[a] FOLFOX6 dosing may continue regardless of FPA144 dose modifications.

[b] One dose level lower for patients treated at 15 mg/kg is 10 mg/kg; one dose level lower for patients treated at 10 mg/kg is 6 mg/kg. One dose lower than 6 mg/kg requires permanent discontinuation.

Any patient who reports pain or irritation of the eye or change in vision should be evaluated by an ophthalmologist.

Table 4. Dose Modification Guidelines for FPA144 (Any Related Corneal Toxicity[a])

FPA144/IP-related Toxicity Grade	Dose Schedule	New FPA144 Dose
Grade 1	No Delay	100%
Grade 2 and Grade 3	Delay dosing, see ophthalmologist and treat with topical (ophthalmologic) antibiotics	If recovery to baseline or Grade 1 within 28 days, may resume at 100% dose
Grade 2 or Grade 3 which does not return to baseline within 28 days<br>Any Grade 4	Permanently discontinue dosing of FPA144	N/A

[a] mFOLFOX6 dosing may continue regardless of FPA144 dose modifications.

There is a ± 3-day window for the first 3 scheduled IP dosing visits. After 3 doses, FPA144/IP can be delayed up to a maximum of 7 days to align with mFOLFOX6 chemotherapy infusion, however alignment of FPA144/IP with chemotherapy is not required. Patients should not have 2 consecutive doses of FPA144 within 7 days. The first dose of each cycle is considered Day 1 of each cycle. Cycles will repeat every 14 days unless there is a treatment delay. Intra-patient dose escalation above the starting dose for each patient will not be permitted. Any patient whose dose of FPA144/IP is decreased cannot be subsequently increased.

5.4.5 Dose Interruptions During Study Treatment Infusion

5.4.5.1 Open-label FPA144 (Phase 1) and Blinded Investigational Product (Phase 3)

Infusion of FPA144/IP must be stopped if any AE ≥ Grade 3 occurs during the infusion. If bronchospasm or dyspnea occurs in a patient during the infusion, the infusion must be stopped.

In addition, at the Investigator's discretion, the infusion rate for FPA144/IP may be reduced or stopped if a less severe AE (Grade 1 or 2) occurs during the infusion. If a Grade 3 or less severe AE resolves within 4 hours, the infusion may be restarted at half the previous rate and promptly managed according to the discretion of the Investigator. If the same AE appears again with the same severity at any time during the restarted infusion, the infusion should be discontinued, and no further dosing of FPA144/IP will occur without consultation with the Sponsor or Sponsor's designee. It is up to Investigator discretion whether or not to treat with mFOLFOX6 in the event of infusion reaction with FPA144/IP.

If a patient experiences an infusion reaction prior to completion of the infusion, the infusion must be stopped, and the patient should be promptly managed and monitored according to signs and symptoms, and local clinical protocol until there is a complete resolution of the event. Symptoms of infusion reactions may include: fever, chills, rigors, urticaria, hypotension and hypertension with headache, wheeze, breathlessness, hypoxia, and pulmonary infiltrates. For patients whose

infusion-associated events were either Grade 1 or 2, and completely resolved on the day of the infusion, the infusion may be resumed at the discretion of the Investigator at a slower rate with premedication. All subsequent infusions for that patient should then be administered at the reduced rate of infusion with pre-medications. Pre-medications may include medications such as corticosteroids, diphenhydramine, acetaminophen and/or bronchodilators as indicated. FPA144/IP will be permanently discontinued for patients who have experienced Grade 3 or above infusion-associated AEs, and for patients who have recurrent infusion-associated reactions after restarting the infusion despite pre-medications and slower infusion.

If a patient experiences an infusion reaction, the patient’s vital signs (temperature, blood pressure, pulse, and respiration rate) should be monitored during the infusion, as well as every 30 minutes after the infusion for a minimum of 2 hours and until resolution of the infusion reaction.

5.4.5.2 mFOLFOX6

Patients should be closely monitored for mFOLFOX6 toxicity. In Phase 1, dose adjustments for 5-FU, leucovorin, and oxaliplatin are permitted, but patients who require dose adjustments or delay of any component of mFOLFOX6 during the DLT period will not be considered evaluable unless the dose adjustment or delay is due to an AE deemed related to FPA144, in which case the AE will be considered a DLT (see Section 5.4.2 for DLT definition). Beyond the DLT Period in Phase 1, or at any time in Phase 3, dose adjustments for any component of mFOLFOX6 are permitted per the guidelines outlined below or per local standard of care if applicable.

If there is a change in body weight of at least 10%, doses should be recalculated.

Counsel patients to avoid exposure to cold during and for approximately 72 hours after each infusion.

Correct hypokalemia and hypomagnesemia prior to initiating oxaliplatin.

Severe diarrhea, mucositis, and myelosuppression after 5-FU should prompt evaluation for dihydropyrimidine dehydrogenase deficiency.

Leucovorin dose is given for d,l-racemic mixture. Use half the dose for LEVO-leucovorin (l-leucovorin)

In the event that oxaliplatin administration is discontinued for any reason prior to disease progression, 5-FU/leucovorin therapy may continue on an every-14-day schedule until disease progression, unacceptable toxicity, or other cause for study withdrawal. In the case 5-FU/leucovorin therapy is discontinued then oxaliplatin must be discontinued.

Recommended dose adjustments for mFOLFOX6 toxicity are shown in Table 5. The dose adjustments are guidelines, not required, therefore if local guidelines are different, they may be substituted.

Table 5: Dose Reductions and Delays for mFOLFOX6 Chemotherapy

Toxicity	Grade	Oxaliplatin	5-FU/Leucovorin
Neurotoxicity	Persistent (≥ 1 cycle) Grade 2 Neurotoxicity	Decrease from 85 mg/m2 to 65 mg/m2*	No change
	Transient (> 7 days and ≤14 days) Grade 3 Neurotoxicity	Decrease from 85 mg/m2 to 65 mg/m2*	No change
	Persistent (> 1 cycle) ≥ Grade 3 Neurotoxicity or any Grade 4 Neurotoxicity	Discontinue	No change
Gastrointestinal	≥ Grade 3 (after prophylaxis)	Hold until toxicity is ≤ Grade 1, decrease from 85 mg/m2 to 65 mg/m2*	Hold until toxicity is ≤ Grade 1, decrease by 20%*
Hematologic	≥ Grade 3 platelets	Hold until platelets are ≥ 75,000 days, then decrease from 85 mg/m2 to 65 mg/m2*	Reduce by 20%*
	≥ Grade 3 neutropenia	Hold until ANC is ≥ 1500, then decrease from 85 mg/m2 to 65 mg/m2*	Reduce by 20%*
Skin	≥ Grade 3 Hand/foot syndrome	Hold until 5-FU resumes, then no change	Hold until ≤ Grade 1, then decrease by 20%*
Other	≥ Grade 3	Hold until ≤ Grade 1, then decrease from 85 mg/m2 to 65 mg/m2*	Hold until ≤ Grade 1, then reduce by 20%*
Pharyngolaryngeal dysesthesia	Any	Stop infusion, then consider increase duration of infusion up to 6 hours	No change

Pneumonitis	Any	Hold, investigate; discontinue permanently if confirmed	
Hepatic Impairment	Bilirubin 1-2 X ULN	No change	No change, consider decrease by 20%*
	Bilirubin > 2 - 4 X ULN and/or AST/ALT is 2-4 X ULN	No change	No change, consider decrease by 20%
	Bilirubin > 4 X ULN and/or AST/ALT is >4 x ULN	Discontinue	Discontinue
Renal Impairment (Creatinine Clearance)	> 50 mL/min	No change	No change
	30 to < 50 mL/min	No change, consider decrease to 65 mg/m2*	No change
	< 30 mL/min	Discontinue	Decrease dose by 20%*

*If toxicity recurs at the same grade level after dose reduction; consider permanent discontinuation. Note that if 5-FU is permanently discontinued, oxaliplatin and leucovorin should be permanently discontinued.

Adapted from: (Cheeseman 2002, Hochster 2008, Teva Parenteral Medicines Inc 2016, Teva Parenteral Medicines Inc 2014, Teva Pharmaceuticals USA 2012)

5.5 Blinding and Breaking the Blind

Phase 1: Blinding and breaking the blind are not applicable as this portion of the study is open label.

Phase 3: The Investigator, study coordinator(s), patients, and Sponsor study team will be blinded to the identity of treatment assignment from the time of randomization until database lock (upon completion of Phase 3). The site pharmacist (who prepares infusion bags at each study center) will be unblinded to drug assignment.

Unblinding will only occur in the case of patient emergencies and at the conclusion of the study.

Emergency Unblinding of Treatment Assignment

Blinding of Investigational Product is critical to the integrity of this clinical trial. Therefore, if a patient's treatment with IP is disclosed to the study site, the patient will discontinue IP. The patient will be removed from study treatment but continue chemotherapy with mFOLFOX6 and be followed for disease progression and survival according to sections 7.3.5 and 7.3.6.

Unblinding by study site personnel for AEs should only be performed in emergencies when knowledge of the patient's treatment assignment is essential for further management of the patient's medical care. For the emergency unblinding, the treating physician may access the treatment information for this subject through the interactive voice and Web response (IXRS) system. Unblinding a patient's treatment assignment under any other circumstances will be considered a protocol violation. The Investigator should notify the Sponsor's Medical Monitor within 24 hours of unblinding any subject's treatment assignment. Although the Medical Monitor should be contacted, the subject's treatment code should not be communicated to the Medical Monitor. An unblinded notification, including the patient ID, treatment group, and date of unblinding will be provided to the investigator and to the chair of the Independent Data Monitoring Committee (IDMC). A blinded notification that includes only the patient ID and the date of unblinding will be provided to the responsible medical monitor and the Sponsor's Head of Drug Safety (or designee).

5.6 Drug Accountability

The Investigator or appropriately qualified staff is responsible for maintaining accurate study treatment accountability records throughout the study.

The Investigator is responsible for returning all unused study treatment to the Sponsor (or designee), and must verify that no remaining supplies are in the Investigator's possession. The study site is permitted to destroy used or partially used study treatment vials according to the site policy once Sponsor approval of their documented destruction procedure has been obtained. On completion of the study, the number of FPA144/IP vials shipped, destroyed, and returned must be reconciled.

5.7 Investigational Product Compliance

Only qualified trained study center personnel may administer FPA144/IP. Pharmacy personnel trained in the study requirements will monitor compliance with the treatment assignments. Records of study medication administered (date, time, and dose administered relative to time of preparation) will be recorded on the patient's electronic case report form (eCRF).

5.8 Concomitant Medication and Treatment

Supportive care (e.g., anti-emetics, analgesics for pain control) may be used at the Investigator's discretion and in accordance with institutional procedures. Hematopoietic stimulating agents may be used if indicated. Concomitant anti-cancer therapies of any kind are not permitted.

Patients receiving oxaliplatin should not receive oral cryotherapy as this may exacerbate laryngopharyngeal dysesthesia caused by oxaliplatin. For patients on anticoagulant therapy, close monitoring of coagulation parameters is recommended.

6 Parameters and Methods of Assessment

6.1 Safety Parameters

Safety measures will include AEs, hematology, clinical chemistry, urinalysis, vital signs, body weight, concomitant medications/procedures, ECOG performance status, targeted physical examinations, ECGs, and ophthalmology examinations in both Phase 1 and Phase 3.

An independent DMC will evaluate safety study data (AEs and SAEs) on a regular basis throughout the entire treatment phase (as prescribed in the DMC Charter) in Phase 3.

Adverse Events

6.1.1.1 Collection of Adverse Events

In Phase 1, any new symptoms, injury or worsening of symptoms that occur during the Screening Period, i.e., following signing of the ICF but prior to first infusion (Cycle 1 Day 1), will be considered pretreatment events and reported on the Medical History page of the eCRF, unless they directly correlate to a study-related procedure in which case they will be reported on the AE eCRF page. Otherwise, AE reporting will begin at the time of infusion of Cycle 1, Day 1 (day of first infusion) and continue until completion of the End-of-Treatment visit or until 4 weeks (28 days) after the last dose of study treatment.

In Phase 3, AEs should not be reported during the Pre-Screening Period unless they are related to a study procedure, as patients are not yet enrolled on the study at that time. AEs occurring during the Screening Period should be reported as described above for the Phase 1 Screening Period. AE reporting will continue until the End of Treatment Visit.

Since the IHC and ctDNA blood results may require up to two weeks to complete, patients eligible for entering Phase 3 of this study may not be able to delay start of standard chemotherapy while waiting to receive the IHC or ctDNA blood result. Therefore, patients are allowed to receive 1 dose of a platinum and fluoropyrimidine combination prior to enrolling in this clinical trial, while waiting for FGFR2 status to be confirmed. Adverse events due to this single dose of therapy that are ongoing on Study Day 1 will be captured on the medical history eCRF.

Patients in Phase 3 will undergo long-term follow-up for survival by clinic visit or by telephone approximately every 3 months ± 28 days after the EOT visit until up to 24 months after the last patient is enrolled into the study.

SAEs occurring after the EOT visit should be reported to the Sponsor by the Investigator only if the Investigator considers a causal relationship with FPA144/IP or mFOLFOX6. SAEs should always be recorded on the AE eCRF.

6.1.1.2 Definitions

An AE is any untoward medical occurrence that occurs in a patient administered a pharmaceutical product, and which does not necessarily have to have a causal relationship with this treatment. An AE can therefore be any unfavorable and unintended sign (including abnormal laboratory finding), symptom, or disease temporally associated with the use of an investigational product, whether or not considered related to the product. Abnormal laboratory findings that are not considered clinically significant will be recorded only on the laboratory eCRF pages and not on the AE pages. Abnormal laboratory results that are considered clinically significant in the Investigator’s opinion are also to be recorded on the AE eCRF. Relationship (reasonable causal relationship) to drug therapy and counter measures undertaken will be noted on the eCRF.

All AEs including intercurrent illnesses that occur during the study, from the time of administration of study treatment, will be documented on the AE eCRF. Concomitant illnesses, which existed prior to the day of the first study infusion, will not be considered AEs unless they worsen by at least 1 grade during the treatment period. Intensity (severity) grade will be defined according to the NCI-CTCAE, version 4.03. Pre-existing conditions will be recorded on the Medical History eCRF.

A treatment-emergent AE will be defined as an AE that begins or worsens in severity after at least 1 dose of study treatment (FPA144/IP and/or mFOLFOX6) has been administered.

Diagnostic and therapeutic non-invasive and invasive procedures, such as surgery, will not be reported as an AE, but the procedure and/or therapeutic treatment should be recorded on the appropriate eCRF. The medical condition for which the procedure was performed must be reported as an AE (or as part of the patient’s medical history, if the procedure precedes the initiation of study-prescribed treatment). Signs and symptoms associated with disease progression itself should not be reported as an AE or SAE if the diagnosis is available. Disease progression itself is an endpoint and not an AE or SAE.

6.1.1.3 Assessment of Adverse Events

Each AE will be assessed by the Investigator with regard to seriousness, intensity (severity), causality and the outcome and action taken. All AEs, regardless of the relationship to study treatment, will be recorded on the AE eCRF. This includes potential end-organ toxicity, e.g., renal (proteinuria), hepatic, and cardiovascular (increased blood pressure) effects, and effects on

wound healing. All AE reports should contain a brief description of the event, date of onset, ongoing or date of resolution, intensity, treatment required, relationship to study treatment, action taken with the study treatment, outcome, and whether the event is classified as serious as described below.

6.1.1.3.1 Seriousness

An SAE is defined as any untoward medical occurrence that at any dose:

- Results in death. Death may occur as a result of the underlying disease process. All events other than progression of underlying disease that result in death during the reporting period up to 28 days following the last dose of study treatment must be treated as an SAE and reported as such
- Is life-threatening (patient is at immediate risk of death from the event as it occurred)
- Requires inpatient hospitalization (formal admission to a hospital for medical reasons) or prolongation of existing hospitalization
- Results in persistent or significant disability or incapacity
- Is a congenital anomaly or birth defect

Medically significant events that may not result in death, are not life-threatening, or do not require hospitalization may be considered SAEs when, based on appropriate medical judgment, they may jeopardize the patient and may require medical or surgical intervention to prevent one of the outcomes listed in this definition. Examples of such events are intensive treatment in an emergency room or at home for allergic bronchospasm, blood dyscrasias or convulsions that do not result in hospitalization, or development of drug dependency or drug abuse.

Medical and scientific judgment should be exercised in deciding whether a case is considered medically significant or serious and whether expedited reporting is appropriate.

Hospitalization for an event solely related to disease progression is not considered an SAE. Hospitalization for an elective or planned procedure to treat a pre-existing condition is not considered an SAE unless it results in one of the outcomes listed above.

6.1.1.3.2 Intensity (Severity)

Investigators need to assess the severity of AEs according to the guidelines provided in NCI-CTCAE, version 4.03.

CTCAE v 4.03 Severity Grades are:

- Grade 1: Mild; asymptomatic or mild symptoms; clinical or diagnostic observations only; intervention not indicated; mild AE

- Grade 2: Moderate; minimal, local or non-invasive intervention indicated; limiting age-appropriate instrumental activities of daily living; moderate AE
- Grade 3: Severe or medically significant but non-immediately life-threatening; hospitalization or prolongation of hospitalization indicated; disabling; limiting self-care activities of daily living; severe AE
- Grade 4: Life-threatening consequences; urgent intervention indicated
- Grade 5: Fatal AE

If the AE is not specified in the CTCAE or the study protocol, the grading of severity will be assessed as mild (Grade 1), moderate (Grade 2), severe (Grade 3), life-threatening (Grade 4), or death due to the AE (Grade 5) using the following definitions:

- Mild: The patient is aware of the event or symptom, but the event or symptom is easily tolerated.
- Moderate: The patient experiences sufficient discomfort to interfere with or reduce his or her usual level of activity.
- Severe: Significant impairment of functioning: the patient is unable to carry out usual activities.
- Very severe (life-threatening): The patient’s life is at risk from the event.

6.1.1.3.3 Causality

The Investigator will assess the causality/relationship between the study treatment and the AE and record that assessment on the eCRF.

The most likely cause of an SAE (e.g., disease under treatment, concomitant disease, concomitant medication, other) will be indicated on the eCRF with details of the concomitant disease or medication or other cause.

The causal relationship of the AE to study treatment will be assessed by means of a question: ‘Is there a reasonable possibility that the AE may have been caused by the study treatment?’ Answer Yes or No.

The description below provides guiding principles for the investigator to make casualty assessments.

- Yes, there is a reasonable possibility that the AE may have been caused by the study treatment:
    - Follows a reasonable temporal sequence from administration of the study treatment

    - Could not be reasonably explained by the patient's clinical state, environmental or toxic factors, or other therapies administered to the patient
    - Disappears or decreases on cessation or reduction in dose of the study treatment
    - Follows a known pattern of response to the study treatment
    - Reappears or worsens on re-challenge
- No, there is no reasonable possibility that the AE may have been caused by the study treatment:
    - Does not follow a reasonable temporal sequence from administration of the study treatment
    - Could be reasonably explained by the patient's clinical state, environmental or toxic factors, or other therapies administered to the patient
    - Does not follow a known pattern of response to the study treatment
    - Does not reappear or worsen on re-challenge

The relatedness for SAEs will also be assessed and documented on the AE eCRF.

If the causality of the AE requiring discontinuation is confirmed to be due to one of the study treatments in the combination therapy, the other drug may be continued per protocol schedule under the following scenarios:

- Timely resolution of the AE based on the treatment modification table
- Clinical benefit is shown by the patient based on Investigator assessment

6.1.1.3.4 Outcome and Action Taken

The Investigator will record the action taken and outcome for each AE according to the following criteria:

- Action Taken
    - Dose Not Changed
    - Drug Interrupted
    - Drug Withdrawn
    - Not Applicable
    - Unknown
- Outcome
    - Fatal
    - Not Recovered/Not Resolved

- Recovered/Resolved
- Recovered/Resolved with Sequelae
- Recovering/Resolving

6.1.1.4 Reporting Serious Adverse Events

Any SAEs, whether or not considered related to treatment with FPA144/IP or mFOLFOX6, must be reported by the Investigator to the Sponsor or Sponsor's designee within 24 hours of the Investigator becoming aware of the event and must be recorded on both the SAE form and AE eCRF. Additional SAE information including medications or other therapeutic measures used to treat the event, action taken with the study treatment due to the event, and outcome/resolution of the event will be recorded on the SAE form. Forms for reporting SAEs will also be provided to the study centers.

A copy of the SAE forms must be faxed **within 24 hours** to the attention of the ICON Pharmacovigilance Safety Specialist:

ICON Medical and Safety Services
[redacted]

The Investigator should not wait to receive additional information to fully document the event before notification of a SAE, though additional information may be requested. The minimum information that is required for an initial SAE report is as follows:

- Patient number
- Investigator name and study center number
- Event term
- Event onset date
- Serious criteria
- Relationship to study treatment(s)

As applicable, information from relevant laboratory results, hospital case records, and autopsy reports should be obtained.

Serious AEs occurring after the EOT visit should be reported to the Sponsor by the Investigator if the Investigator considers there is a causal relationship with the study treatment.

The Investigator and Sponsor will review each SAE report and evaluate the seriousness and causal relationship of the event to study treatment. In the event of a disagreement about

causality, the most conservative assessment will be used. In addition, the Sponsor will evaluate the expectedness according to the FPA144 Investigator's Brochure. Based on the Investigator and Sponsor's assessment of the event, a decision will be made concerning the need for further action.

The Sponsor or its designee is responsible for submitting reports of AEs associated with the use of the drug that are both serious and unexpected to FDA, per 21 Code of Federal Regulations (CFR) 312.32, and to other regulatory authorities, according to national law and/or local regulations. All Investigators participating in ongoing clinical studies with the study medication will receive copies of these reports for prompt submission to their Institutional Review Board (IRB) or Independent Ethics Committee (IEC).

The Sponsor or its designee will submit all safety updates and periodic reports to the regulatory authorities as required by applicable regulatory requirements.

6.1.1.5 Follow-up of Adverse Events

All treatment-related SAEs experienced by a study patient, will be monitored until the event has resolved or stabilized, any abnormal laboratory values have returned to baseline or stabilized at a level acceptable to the Investigator and Sponsor, there is a satisfactory explanation for the changes observed, or the patient is lost to follow-up.

All unresolved AEs should be followed by the investigator until the events are resolved, the subject is lost to follow-up, or the AE is otherwise explained. The investigator should notify the study sponsor of any death or AE occurring at any time after a subject has discontinued or terminated study participation that may reasonably be related to this study. The sponsor should also be notified if the investigator should become aware of the development of cancer or of a congenital anomaly in a subsequently conceived offspring of a subject that has participated in this study.

6.1.1.6 Pregnancy

Pregnancy is an exclusion criterion and women of childbearing potential must not be considering getting pregnant during the study. Pregnancy tests should be performed for any woman of childbearing potential, as noted in accordance with the Schedule of Assessments (Appendix 2, Appendix 3). Serum ß-hCG (evaluated by local laboratories) and urine pregnancy tests will be performed only on women of childbearing potential.

In the event of suspected pregnancy, a serum pregnancy test should be repeated. Patients who become pregnant during the study must discontinue study treatment immediately.

The Sponsor must be notified of any patient who becomes pregnant while participating in this study. Although pregnancy is not an AE, all pregnancies must be followed to conclusion to determine their outcome. It is the responsibility of the Investigator or designee to report any

pregnancy in a patient that occurs during the study by completing the Pregnancy Reporting Form. Please contact the study monitor to receive the Pregnancy Reporting Form on learning of a pregnancy.

Notification of the pregnancy including the anticipated date of birth should be submitted on a Pregnancy Reporting Form within 24 hours of awareness and reported using the same procedure as described for reporting SAEs (Section 6.1.1.4). If the pregnancy is to be terminated, the anticipated date of termination should be provided.

The patient will be asked to provide information on the outcome of the pregnancy, including premature termination should the case arise. Spontaneous miscarriages, premature termination of the pregnancy, and congenital abnormalities will be reported as SAEs. Information on the status of the mother and child will be forwarded to the Sponsor. Generally, follow-up will be in accordance with regulatory guidance and at least 6 to 8 weeks after the estimated delivery date.

Pregnancies that occur during the first 6 months of the Follow-up period should be reported to the Sponsor, and followed as described above.

6.2 Laboratory Parameters

Laboratory assessments (listed in Appendix 4) will be performed locally at each study center's laboratory by means of their established methods. Before starting the study, the Investigator will provide the Sponsor (or designee) with a list of the normal ranges and units of measurement.

Blood samples should be taken using standard venipuncture techniques. Laboratory assessments will be performed in accordance with the Schedule of Assessments (Appendix 2, Appendix 3). Abnormal laboratory results that lead to a change in patient treatment management (e.g., dose delay, requirement for additional medication or monitoring) are considered clinically significant for the purposes of this study and will be recorded on the AE page of the eCRF. Values meeting SAE criteria must be reported as SAEs. Refer to Section 6.1.1.2 for details around the reporting of abnormal laboratory findings as AEs.

6.3 Vital Signs

Vital signs (blood pressure, pulse, respiration, and temperature) are to be measured in accordance with the Schedule of Assessments (Appendix 2, Appendix 3).

6.4 Electrocardiograms

Twelve-lead ECGs will be performed in accordance with the Schedule of Assessments (Appendix 2, Appendix 3). The Investigator must review the ECG, document this review in the source documents, and record any clinically significant changes that occur during the study as an AE in the eCRF.

6.5 Physical and Ophthalmologic Examinations

A complete physical examination including height and weight will be performed at Screening. Limited physical examinations should be conducted per the Schedule of Assessments (Appendix 2, Appendix 3) and include examination of the oropharynx. After Cycle 1, the FPA144/IP dose will be recalculated at each infusion visit only if weight has changed >10% from Cycle 1, Day 1.

Comprehensive ophthalmologic examinations (conducted at Screening, prior to Cycle 3 Day 1, and at the EOT visit) include fundoscopic and slit lamp exam, ocular coherence tomography (OCT), visual acuity, completion of fluorescein staining score form (Appendix 8), determination of intraocular pressure, and review of ocular/visual symptoms.

When performing the ocular examination by the ophthalmologist, the following should be noted:

- Intraocular pressure (IOP) can be measured by tonometer or applanation, but should be done before dilation
- Confrontation VF is adequate
- OCT should include the macula
- Corneal staining and scoring should be done before dilation and before the IOP check as both may disrupt corneal integrity

The comprehensive ophthalmologic examination will be repeated at any point if changes in visual acuity or visual symptoms are reported by patients.

Slit lamp examinations, (with completion of fluorescein staining score form), should be conducted for all patients every 8 weeks from Cycle 3 Day 1 through study completion.

6.6 Immunogenicity

Immunogenicity, defined as an immune response to FPA144, will be assessed by measurement of total anti-FPA144 antibodies from all patients. Immunogenicity testing will consist of screening, confirmation, and titration. Additional characterization of a confirmed anti-FPA144 antibody response may be considered.

Samples for immunogenicity assessment will be drawn from each patient at the time points outlined in Appendix 5 and Appendix 6. Samples for immunogenicity testing will be collected and processed according to the instruction provided in the Laboratory Manual.

6.7 Tumor Analysis for Patient Selection

6.7.1 Phase 1

Patients eligible for Phase 1 have unselected GI cancer (with or without FGFR2 positive status) with unresectable, locally advanced, or metastatic disease, and are candidates to receive both FPA144 and mFOLFOX6 chemotherapy. FGFR2 status will be determined retrospectively by IHC and ctDNA blood assay.

6.7.2 Phase 3

Patients in Phase 3 of this study must consent to tumor tissue analysis and blood sample analysis. Patients will be selected for enrollment based on FGFR2b overexpression and/or *FGFR2* amplification, as determined by a validated IHC or ctDNA blood assay, respectively. Patients who do not demonstrate either FGFR2b overexpression using IHC or *FGFR2* amplification using a ctDNA blood assay will not be eligible for enrollment; however, positivity based on one or both of the assays is adequate to meet eligibility requirements (e.g., positive by ctDNA blood assay, but negative by IHC). It is the responsibility of each Investigator to obtain an adequate tumor specimen for analysis of FGFR2 positivity for enrollment. A minimum of 5 slides are required. Tumor slide or tumor block specimen processing, labeling, and shipping instructions are detailed in the Laboratory Manual that will be distributed with the specimen collection kit.

Central laboratories will perform the FGFR2b overexpression and *FGFR2* amplification analysis using a validated IHC test and ctDNA blood assay.

For Phase 3, once tumor and blood specimens are received, analysis will be performed as efficiently as possible, and results will be communicated back to the Investigator or designee.

6.8 Tumor Assessments

Tumor assessments will be performed in Phase 3 only. Tumor assessments should consist of clinical examination and appropriate imaging techniques (preferably CT scans with appropriate slice thickness per RECIST v1.1, MRI acceptable). The same methods used to detect lesions at baseline are to be used to follow the same lesions throughout the clinical study.

Tumor response assessment will be performed by the Investigator per RECIST 1.1 guidelines. The BICR will collect, hold and be ready to analyze a proportion of patient radiographic images. The independent review by BICR will be conducted upon Sponsor’s agreement. Full details around independent review by a BICR will be listed in an Independent Imaging Review Charter.

Tumor scans will be performed at Screening (within 28 days of Cycle 1 Day 1 allowed) and then every 8 weeks (± 7 days) from Enrollment (Cycle 1 Day 1) thereafter.

After discontinuation of study treatment for reasons other than disease progression, or withdrawal of consent or initiation of additional anti-cancer therapy, tumor assessments will continue every 8 weeks.

6.9 ctDNA Blood Assay

In Phase 1, samples for ctDNA blood assay will be collected prior to the first dose of study treatment (Cycle 1 Day 1) and analyzed for *FGFR2* amplification. Results do not need to be available prior to enrollment and dosing.

In Phase 3, a ctDNA blood assay will be done during Pre-screening. This sample will be analysed prospectively for *FGFR2* amplification.

6.10 Pharmacodynamic Biomarker Analysis Using Blood

Blood samples for exploratory biomarker analysis of the FGFR pathway (for example: FGF7, FGF10) will be collected from patients enrolled in the Phase 1 portion of the study at the timepoints specified in Appendix 5.

6.11 Quality of Life Scales

The EQ-5D-5L quality of life (QOL) questionnaire and the EORTC QLQ-C30 will be administered on multiple occasions (see Appendix 3 for time points) in Phase 3.

The EQ-5D-5L questionnaire was developed by the EuroQol Group, which is a standardized measure to provide utilities for clinical and economic appraisal. It uses a descriptive system and a visual analogue scale (VAS). The descriptive system has 5 dimensions: mobility, self-care, usual activities, pain/discomfort and anxiety/depression, and each dimension has 5 levels: no problems, slight problems, moderate problems, severe problems, and extreme problems.

Respondents are asked to indicate their health state by marking the box against the most appropriate statement in each of the 5 dimensions. The digits for the 5 dimensions can be combined in a 5-digit number describing the respondent's health state. Health states defined by the EQ-5D-5L descriptive system are converted into a single index value to calculate utilities (Herdman 2011). The VAS portrays the respondent's self-rated health on a 20-cm vertical VAS, with endpoints labeled "the best health you can imagine" and "the worst health you can imagine".

The European Organisation for Research and Treatment of Cancer (EORTC) quality of life questionnaire (QLQ) is an integrated system for assessing the health-related quality of life of cancer patients participating in international clinical trials. The EORTC uses a modular approach to QOL assessment, consisting of a core questionnaire (EORTC QLQ-C30) to be administered, if necessary with a module specific to tumor site, treatment modality or a QOL dimension (e.g., GC-specific module is QLQ-STO22).

The patient provides answers for five functional scales (physical, role, emotional, social, and cognitive), three symptom scales (fatigue, nausea and vomiting, and pain) and a global health status/QOL scale and six single items (dyspnea, insomnia, appetite loss, constipation, diarrhea, and financial difficulties).

6.12 ECOG Performance Status

ECOG performance status will be assessed in all patients at the time points outlined in Appendix 2 and Appendix 3.

The ECOG performance status is a scale used to assess how a patient's disease is progressing, assess how the disease affects the daily living abilities of the patient, and determine appropriate treatment and prognosis. The ECOG scale is shown in Appendix 7.

6.13 Pharmacokinetic Assessment

Blood samples to determine serum FPA144 concentration will be acquired from each patient as outlined in the Study Flowchart for Pharmacokinetic, Immunogenicity, and Pharmacodynamic Blood Sample Collections (Appendix 5 and Appendix 6). These samples will be collected and processed according to the instructions provided in a separate Laboratory Manual.

7 Study Conduct

7.1 Overview of Patient Assessments

The schedule of detailed patient assessments is shown in Appendix 2 (Phase 1) and Appendix 3 (Phase 3). The list of safety laboratory assessments is shown in Appendix 4. Instructions for the sampling and processing of PK, PD, and immunogenicity data are provided in a flowchart in Appendix 5 (Phase 1) and Appendix 6 (Phase 3).

7.2 Study Assessments and Procedures by Visit - Phase 1

7.2.1 Screening Period (Day –28 to Day 0) (Phase 1)

Written, signed informed consent must be collected prior to any study-specific procedures. Patients who have fully consented to participation in the study will undergo screening assessments within 28 days prior to administration of the first infusion of study treatment. The following procedures will be performed:

- Review/confirm eligibility criteria
- Medical/oncology and medication history
- Tumor tissue collection from archival or newly obtained material for analysis of FGFR2b overexpression by IHC

- Demographic and baseline characteristics
- Complete physical examination, including weight and height
- ECOG performance status evaluation
- Vital signs (blood pressure, pulse, respiration, and body temperature [°C] after 5 minutes of rest)
- 12-lead ECG after 5 minutes of rest prior to recording
- Comprehensive ophthalmologic examination
- Clinical safety laboratory sampling (see Appendix 4)
- Serum pregnancy test (beta-human chorionic gonadotropin [β-HCG]), ≤ 96 hours prior to dosing, for women of childbearing potential
- Urinalysis (includes dipstick for protein, glucose, blood, pH, and ketones)
- AE reporting, if applicable

7.2.2 Treatment Period (Phase 1)

7.2.2.1 Cycle 1, Day 1 (Phase 1)

The following procedures will be performed:

- Review/confirm eligibility criteria
- Update medical, disease and medication history to capture any changes from screening
- Limited physical examination, including examination of the oropharynx.
- Vital signs (blood pressure, pulse, respiration, and body temperature [°C]) pre-dose and at 0.5, 1, 2, and 4 hours from the start of FPA144 infusion
- Clinical safety laboratory sampling, with results obtained within 96 hours of Cycle 1 Day 1 to confirm eligibility (see Appendix 4). Safety laboratory results necessary to start treatment include an ANC $\geq 1.5 \times 10^9$/L, platelets $\geq 100 \times 10^9$/L, AST/ALT ≤ 3x ULN or ≤ 5x ULN if liver metastases, bilirubin < 1.5x ULN, and creatinine clearance ≥ 50 mL/min (see Appendix 1)
- Serum pregnancy test (β-HCG), ≤ 96 hours prior to dosing, for women of childbearing potential
- Urinalysis (includes dipstick for protein, glucose, blood, pH, and ketones)
- Blood sampling for PK, immunogenicity testing, and exploratory biomarker analysis of the FGFR pathway as outlined in Appendix 5
- Sample for ctDNA blood assay: a ctDNA sample will be collected prior to dosing on Cycle 1 Day 1 for retrospective analysis.
- AE reporting

- Review of concomitant medications

Study treatment administration:

- FPA144 administered by IV infusion over 30 minutes (± 5 minutes)
- mFOLFOX6 chemotherapy administered after 30 to 60 minutes of rest following FPA144 administration

7.2.2.2 Cycle 1, Day 3 (Phase 1)

The following procedures will be performed:

- Vital signs (blood pressure, pulse, respiration, and body temperature [°C] after 5 minutes of rest)
- Blood sampling for PK and exploratory biomarker analysis of the FGFR pathway as outlined in Appendix 5
- AE reporting
- Review of concomitant medications

7.2.2.3 Cycle 1, Day 8 (Phase 1)

The following procedures will be performed:

- Limited physical examination, including examination of the oropharynx
- Vital signs (blood pressure, pulse, respiration, and body temperature [°C] after 5 minutes of rest)
- Clinical safety laboratory sampling (see Appendix 4)
- Blood sampling for PK analysis as outlined in Appendix 5
- AE reporting
- Review of concomitant medications

7.2.2.4 Cycle 2 Day 1 (Phase 1)

The following procedures will be performed:

- Limited physical examination, including examination of the oropharynx
- Vital signs (blood pressure, pulse, respiration, and body temperature [°C]) measured pre-dose and at 0.5,1, and 2 hours from the start of FPA144 infusion

- Clinical safety laboratory sampling, with results obtained within 72 hours prior to dosing (see Appendix 4). Safety laboratory results necessary to start treatment include an ANC $\geq 1.5 \times 10^9$/L, platelets $\geq 100 \times 10^9$/L, AST/ALT ≤ 3x ULN or ≤ 5x ULN if liver metastases, bilirubin < 1.5x ULN, and creatinine clearance ≥ 50 mL/min (see Appendix 1)
- Blood sample for PK and immunogenicity testing as outlined in Appendix 5
- AE reporting
- Review of concomitant medications

Study treatment administration:

- FPA144 administered by IV infusion over 30 minutes (± 5 minutes)
- mFOLFOX6 chemotherapy administered after 30 to 60 minutes of rest following FPA144 administration

7.2.2.5 Cycle 2 Day 3 (Phase 1)

- Vital signs (blood pressure, pulse, respiration, and body temperature [°C]) after 5 minutes of rest
- Blood sampling for PK analysis as outlined in Appendix 5.
- AE reporting
- Review of concomitant medications

7.2.2.6 Cycle 3 Day 1 and Day 1 of Subsequent Odd Numbered Cycles (Except as Noted) (Phase 1)

On completion of the DLT Period, patients may continue receiving FPA144 and mFOLFOX6, administered in 14-day cycles until Investigator-assessed radiographic or clinical disease progression, unacceptable toxicity, or until the patient meets any of the other protocol-specified withdrawal criteria. There is no maximum number of doses of FPA144. Ongoing administration of the mFOLFOX6 regimen beyond the DLT Period will be according to local standard of care.

The following procedures will be performed:

- Limited physical examination, including examination of the oropharynx
- ECOG performance status evaluation
- Vital signs (blood pressure, pulse, respiration, and body temperature [°C]) performed pre-dose and at 0.5,1, and 2 hours from the start of FPA144 infusion
- Comprehensive ophthalmologic examination

- Clinical safety laboratory sampling, with results obtained within 72 hours prior to dosing (see Appendix 4). Safety laboratory results necessary to start treatment include an ANC $\geq 1.5 \times 10^9$/L, platelets $\geq 100 \times 10^9$/L, AST/ALT ≤ 3x ULN or ≤ 5x ULN if liver metastases, bilirubin < 1.5x ULN, and creatinine clearance ≥ 50 mL/min (see Appendix 1)
- Urine pregnancy test, ≤ 72 hours prior to dosing, for women of childbearing potential
- Urinalysis (includes dipstick for protein, glucose, blood, pH, and ketones)
- Blood sampling for PK, immunogenicity testing, and exploratory biomarker analysis of the FGFR pathway as outlined in Appendix 5.
- AE reporting
- Review of concomitant medications

Study treatment administration:

- FPA144 administered by IV infusion over 30 minutes (± 5 minutes)
- mFOLFOX6 chemotherapy administered after 30 to 60 minutes of rest following FPA144 administration

7.2.2.7 Cycle 4 Day 1 and Day 1 of Subsequent Even Numbered Cycles (Phase 1)

The following procedures will be performed:

- Limited physical examination, including examination of the oropharynx
- Vital signs (blood pressure, pulse, respiration, and body temperature [°C]) measured pre-dose and at 0.5,1, and 2 hours from the start of FPA144 infusion
- Clinical safety laboratory sampling, with results obtained within 72 hours prior to dosing (see Appendix 4). Safety laboratory results necessary to start treatment include an ANC $\geq 1.5 \text{ X } 10^9$/L, platelets $\geq 100 \times 10^9$/L, AST/ALT ≤ 3x ULN or ≤ 5x ULN if liver metastases, bilirubin < 1.5x ULN, and creatinine clearance ≥ 50 mL/min (see Appendix 1)
- AE reporting
- Review of concomitant medications

Study treatment administration:

- FPA144 administered by IV infusion over 30 minutes (± 5 minutes)
- mFOLFOX6 chemotherapy administered after 30 to 60 minutes of rest following FPA144 administration

In addition:

Cycle 10 Day 1 (Phase 1)

- Blood sampling for PK testing as outlined in Appendix 5.

Cycle 10 Day 3 (Phase 1)

- Blood sampling for PK and exploratory biomarker analysis of the FGFR pathway as outlined in Appendix 5.

Slit Lamp Examinations

- Slit lamp examinations, (with completion of fluorescein staining score form), should be conducted for all patients every 8 weeks from Cycle 3 Day 1 through study completion. OCT is not required at these time points.

7.2.2.8 Cycle 15 Day 1 and Day 1 of Every Subsequent 8 Cycles (Phase 1)

- Blood sampling for PK, immunogenicity testing, and exploratory biomarker analysis of the FGFR pathway as outlined in Appendix 5.

7.2.3 End-of-Treatment Visit or Early Termination (Phase 1)

Patients will return to the study center approximately 28 (± 3) days after the last study treatment administration, or in the event a patient discontinues prematurely from the study.

The following assessments will be performed at the End-of-Treatment visit:

- Limited physical examination, including examination of the oropharynx
- ECOG performance status evaluation
- Vital signs (sitting pulse, blood pressure, respiration, and body temperature [°C] after 5 minutes of rest)
- 12-lead ECG after 5 minutes of rest
- Comprehensive ophthalmologic examinations
- Clinical safety laboratory sampling (see Appendix 4)
- Serum pregnancy test, for women of childbearing potential
- Urinalysis (includes dipstick for protein, glucose, blood, pH, and ketones)
- Blood samples for PK, immunogenicity testing, and exploratory biomarker analysis of the FGFR pathway as outlined in Appendix 5.
- AE reporting
- Review of concomitant medications

7.3 Study Assessments and Procedures by Visit – Phase 3

7.3.1 Pre-screening Period (Phase 3)

Written, signed informed consent (Pre-screening ICF) must be collected prior to any study-specific procedures.

- Prospective IHC analysis of FGFR2b expression and a ctDNA blood assay demonstrating *FGFR2* amplification (see Section 6.3).

7.3.2 Screening Period (Day –21 to Day 0) (Phase 3)

Written, signed informed consent must be collected prior to any study-specific procedures. Patients who have fully consented to participation in the study will undergo screening assessments within 21 days prior to administration of the first infusion of IP. The following procedures will be performed:

- Review/confirm eligibility criteria
- Medical/oncology and medication history
- Demographic and baseline characteristics
- Complete physical examination, including weight and height
- ECOG performance status evaluation
- Patient reported outcomes (EQ-5D-5L and EORTC QLQ-C30)
- Vital signs (blood pressure, pulse, respiration, and body temperature [°C] after 5 minutes of rest)
- 12-lead ECG after 5 minutes of rest prior to recording
- Comprehensive ophthalmologic examination
- Clinical safety laboratory sampling (see Appendix 4)
- Serum pregnancy test (beta-human chorionic gonadotropin [β-HCG]), ≤ 96 hours prior to dosing, for women of childbearing potential
- Urinalysis (includes dipstick for protein, glucose, blood, pH, and ketones)
- Radiological/tumor scans: tumor assessments performed within 28 days prior to start of treatment and including clinical examination and appropriate imaging techniques (preferably CT scans with appropriate slice thickness per RECIST v1.1, MRI acceptable)
- Randomization
- AE reporting, if applicable

7.3.3 Treatment Period (Phase 3)

7.3.3.1 Cycle 1, Day 1 (Phase 3)

The following procedures will be performed:

- Review/confirm eligibility criteria
- Update medical, disease and medication history to capture any changes from screening
- Limited physical examination, including examination of the oropharynx
- Vital signs (blood pressure, pulse, respiration, and body temperature [°C]) pre-dose and at 0.5, 1, 2, and 4 hours from the start of IP infusion
- Clinical safety laboratory sampling, with results obtained within 96 hours of Cycle 1 Day 1 prior to dosing to confirm eligibility (see Appendix 4). All patients must meet the following criteria prior to every cycle of treatment: ANC $\geq 1.5 \times 10^9$/L, platelets $\geq 100 \times 10^9$/L, AST/ALT $\leq$ 3 X ULN of $\leq$ 5X ULN if liver metastases, bilirubin < 1.5 X ULN, and creatinine clearance $\geq$ 50 mL/min (see Appendix 1)
- Serum pregnancy test (β-HCG), ≤ 96 hours prior to dosing, for women of childbearing potential
- Urinalysis (includes dipstick for protein, glucose, blood, pH, and ketones)
- Blood sampling for PK and immunogenicity testing, as outlined in Appendix 6
- AE reporting
- Review of concomitant medications

Study treatment administration:

- IP administered by IV infusion over 30 minutes (± 5 minutes)
- mFOLFOX6 chemotherapy administered after 30 to 60 minutes of rest following IP administration

7.3.3.2 Cycle 1, Day 8 (Phase 3)

The following procedures will be performed:

- Limited physical examination, including examination of the oropharynx
- Vital signs (blood pressure, pulse, respiration, and body temperature [°C] after 5 minutes of rest)
- Clinical laboratory safety sampling (see Appendix 3).
- AE reporting
- Review of concomitant medications

7.3.3.3 Cycle 2 Day 1 (Phase 3)

The following procedures will be performed:

- Limited physical examination, including examination of the oropharynx
- Vital signs (blood pressure, pulse, respiration, and body temperature [°C]) measured pre-dose and at 0.5,1, and 2 hours from the start of IP infusion
- Clinical laboratory safety sampling, with results obtained within 72 hours prior to dosing (see Appendix 3). Patients must meet the following criteria prior to every cycle of treatment: ANC ≥ 1.5 × 10^9/L, platelets ≥ 100 × 10^9/L, AST/ALT ≤ 3 X ULN of ≤ 5X ULN if liver metastases, bilirubin < 1.5 X ULN, and creatinine clearance ≥ 50 mL/min (see Appendix 1)
- AE reporting
- Review of concomitant medications

Study treatment administration:

- IP administered by IV infusion over 30 minutes (± 5 minutes)
- mFOLFOX6 chemotherapy administered after 30 to 60 minutes of rest following IP administration

7.3.3.4 Cycle 3 Day 1 and Day 1 of Subsequent Odd Numbered Cycles (Except as Noted) (Phase 3)

Patients may continue receiving IP and mFOLFOX6 administered in 14-day cycles until Investigator-assessed radiographic disease progression, unacceptable toxicity, or until the patient meets any of the other protocol-specified withdrawal criteria. There is no maximum number of doses of IP. Ongoing administration of the mFOLFOX6 regimen will be according to local standard of care.

The following procedures will be performed:

- Limited physical examination, including examination of the oropharynx
- ECOG performance status evaluation
- Vital signs (blood pressure, pulse, respiration, and body temperature [°C]) performed pre-dose and at 0.5,1, and 2 hours from the start of IP infusion
- Comprehensive ophthalmologic examination
- Clinical safety laboratory testing, with results obtained within 72 hours prior to dosing (see Appendix 4). Patients must meet the following criteria prior to every cycle of treatment: ANC ≥ 1.5 × 10^9/L, platelets ≥ 100 × 10^9/L, AST/ALT ≤ 3 X ULN of ≤ 5 X ULN if liver metastases, bilirubin < 1.5 X ULN, and creatinine clearance ≥ 50 mL/min (see Appendix 1)

- Urine pregnancy test, ≤ 72 hours prior to dosing, for women of childbearing potential
- Urinalysis (includes dipstick for protein, glucose, blood, pH, and ketones)
- Blood sampling for PK and immunogenicity testing, as outlined in Appendix 6.
- AE reporting
- Review of concomitant medications

Study treatment administration:

- IP administered by IV infusion over 30 minutes (± 5 minutes)
- mFOLFOX6 chemotherapy administered after 30 to 60 minutes of rest following IP administration

7.3.3.5 Cycle 4 Day 1 and Day 1 of Subsequent Even Numbered Cycles (Phase 3)

The following procedures will be performed:

- Limited physical examination, including examination of the oropharynx
- Vital signs (blood pressure, pulse, respiration, and body temperature [°C]) measured pre-dose and at 0.5,1, and 2 hours from the start of IP infusion
- Clinical safety laboratory sampling, with results obtained within 72 hours prior to dosing (see Appendix 4). Patients must meet the following criteria prior to every cycle of treatment: ANC ≥ 1.5 × 10^9/L, platelets ≥ 100 × 10^9/L, AST/ALT ≤ 3 X ULN of ≤ 5 X ULN if liver metastases, bilirubin < 1.5 X ULN, and creatinine clearance ≥ 50 mL/min (see Appendix 1)
- AE reporting
- Review of concomitant medications

Study treatment administration:

- IP administered by IV infusion over 30 minutes (± 5 minutes)
- mFOLFOX6 chemotherapy administered after 30 to 60 minutes of rest following IP administration

7.3.3.6 Imaging Evaluation

Radiological/tumor scans: Tumor assessments performed every 8 weeks (± 7 days) from Enrollment (Cycle 1 Day 1). Assessments include clinical examination and appropriate imaging techniques (preferably CT scans with appropriate slice thickness per RECIST v1.1, MRI acceptable).

7.3.3.7 Slit Lamp Examinations

Slit lamp examinations, without OCT, every 8 weeks from Cycle 3 Day 1 through study completion.

7.3.4 End-of-Treatment Visit or Early Termination (Phase 3)

Patients will return to the study center approximately 28 (± 3) days after the last study treatment administration, or in the event a patient discontinues prematurely from the study.

The following assessments will be performed at the End-of-Treatment visit:

- Limited physical examination, including examination of the oropharynx
- ECOG performance status evaluation
- Patient reported outcomes (EQ-5D-5L and EORTC QLQ-C30)
- Vital signs (sitting pulse, blood pressure, respiration, and body temperature [°C] after 5 minutes of rest)
- 12-lead ECG after 5 minutes of rest
- Comprehensive ophthalmologic examinations
- Clinical safety laboratory sampling (see Appendix 4)
- Serum pregnancy test, for women of childbearing potential
- Urinalysis (includes dipstick for protein, glucose, blood, pH, and ketones)
- Tumor scan, which can be omitted if the last scan was performed < 8 weeks prior to EOT visit or if tumor progression was previously determined (window of ± 7 days)
- Blood samples for PK and immunogenicity testing, as outlined in Appendix 6
- AE reporting
- Review of concomitant medications

Note: After discontinuation of study treatment for reasons other than radiographical disease progression or withdrawal of consent, tumor assessments will continue until the patient initiates additional anti-cancer therapy or progresses.

7.3.5 Additional Follow-up for Patients Without Progression at End-of-Treatment Visit (Phase 3) (Scan Follow-up)

If a patient discontinues study treatment (IP and/or mFOLFOX6) for reasons other than disease progression or withdrawal of consent, they will have follow-up visits and continue to undergo tumor assessments until radiographic progression or the initiation of additional anti-cancer therapy. The following evaluations will be done every 8 weeks (± 7 days):

- Tumor assessments. Assessments include clinical examination and appropriate imaging techniques (preferably CT scans with appropriate slice thickness per RECIST v1.1, MRI acceptable).
- Limited physical examination, including examination of the oropharynx
- Vital signs (sitting pulse, blood pressure, respiration, and body temperature [°C] after 5 minutes of rest)
- Clinical safety laboratory sampling (see Appendix 4)
- Serum pregnancy test, for women of childbearing potential

7.3.6 Long-term Follow-Up (Phase 3)

- During the first 6 months of the Follow-up Period, any pregnancy that occurs should be reported to the Sponsor (see Section 6.1.1.6).
- Patients will undergo long-term follow-up for survival by clinic visit or by telephone approximately every 3 months ± 28 days after the EOT visit until up to 24 months after the last patient is enrolled into the study, or until death, loss to follow-up, withdrawal of consent or study termination by the Sponsor (whichever occurs first), which will be considered the end of study.
- During the Follow-up Period, if the patient undergoes anti-cancer therapy, this should be documented.
- Serious AEs occurring after the EOT visit should be reported to the Sponsor by the Investigator if the Investigator considers there is a causal relationship with the study treatment.

8 Statistical Methods

Before database lock, a statistical analysis plan (SAP) will be finalized, providing detailed methods for the analyses outlined below. Any deviations from the planned analyses will be described and justified in the final integrated study report.

8.1 Analysis Populations

The following analysis populations are defined for the study:

- Safety Population: all enrolled patients who have received any portion of at least one dose of study treatment (FPA144 + mFOLFOX6 or placebo + mFOLFOX6).
- DLT-evaluable Population: all patients enrolled into Phase 1 of the study who received at least 2 doses of FPA144 and mFOLFOX6 and completed Cycles 1 and 2 of treatment, or who experienced a DLT in Cycle 1 or Cycle 2.

- PK-evaluable Population: all patients who have received at least one dose of FPA144 and have had adequate PK assessments drawn for determination of the FPA144 concentration. Adequacy will be determined on a case-by-case basis and will be assessed prior to analysis of the blood samples.
- Intent-to-treat (ITT) Population: all randomized patients (Phase 3).
- Efficacy-evaluable Population: all patients who met eligibility criteria, received at least 1 dose of study treatment (FPA144 + mFOLFOX6 or placebo + mFOLFOX6), and have at least 1 post-baseline disease assessment and no major protocol deviation that could introduce bias in any efficacy assessment.

8.2 General Considerations

The total enrollment planned for this study is approximately 372 patients. Up to 12 patients evaluable for any DLT, per modified 3+3 design, will be enrolled into Phase 1.

For Phase 3, efficacy and tolerability will be examined by enrollment of approximately 360 patients with FGFR2-selected GC, randomized 1:1 to receive FPA144 and mFOLFOX6 or placebo and mFOLFOX6. Eligible patients will be stratified according to geographic region (US and EU vs Asia vs Rest of World), prior treatment status (*de novo* vs adjuvant/neo-adjuvant), and administration of a single platinum and fluoropyrimidine dose prior to enrollment (yes or no).

Power and Sample Size in Phase 3

This study is designed to provide adequate power for primary analysis of PFS.

Based on a mPFS for patients receiving placebo and mFOLFOX6 of 6 months, approximately 156 patients (randomized 1:1) with a target of 96 PFS events are required to detect a hazard ratio (HR) of 0.5 for PFS with a power of 90% (2-sided α =0.05) for the combination of FPA144 and mFOLFOX6 compared with placebo and mFOLFOX6 after 24 months of accrual and 6 months of follow up. There is an interim analysis for futility, in which futility boundary is non-binding.

Assuming an exponential distribution of PFS, this corresponds to an increase in median PFS from 6 months to 12 months. In the current design, the minimum observed effect that would result in statistical significance for PFS is a 50% improvement (HR = 0.67) from 6 to 9 months.

This study is also powered for primary analysis of OS.

Based on a mOS for patients receiving placebo and mFOLFOX6 of 10 months, enrollment of the study will continue to approximately 360 patients with a target of 249 death events to detect an HR of 0.7 for OS with a power of 80% at the overall type I error level of 0.05 for the combination of FPA144 and mFOLFOX6 compared to placebo and mFOLFOX6 after 36 months of accrual and 10 months of follow-up after enrollment of the last patient. The group sequential

method will be used to allocate type I error rate based on O'Brien-Fleming boundary and type II error rate based on the Gamma family with parameter -4 at the interim and final analysis of OS.

Assuming an exponential distribution of OS, this corresponds to an increase of 43% in median OS from 10 months to 14.3 months. In the current design, the minimum observed effect that would result in statistical significance for OS at the final analysis is a 28% improvement (HR = 0.78) from 10 to 12.8 months.

Power and sample size estimates were estimated using EAST®(V6.4).

8.3 Efficacy Analyses

In Phase 1, all analyses will be descriptive and will be presented by dose group and overall as appropriate. Descriptive statistics will include number of observations, mean, standard deviation, median, range, and inter-quartile range for continuous variables, and the number and percent for categorical variables; 95% confidence intervals will be presented where appropriate.

8.3.1 Analysis of Primary Efficacy Endpoint

In Phase 3, the primary efficacy analysis is the comparison of PFS in patients treated with FPA144 in combination with mFOLFOX6 or placebo and mFOLFOX6.

The primary endpoint, PFS, is defined as time from randomization until the date of radiologically confirmed progressive disease based on Investigator assessment (per RECIST v.1.1) or death from any cause, whichever comes first. The secondary efficacy endpoints include OS and ORR.

The hypothesis of PFS will be tested first. There will be an interim analysis and primary analysis for PFS and both are event-based analyses. Only a futility test of PFS will be conducted at the interim analysis after 48 events (50% of target 96 PFS events for primary analysis of PFS) have been observed in the enrolled patients to exclude HR>0.806 for the combination of FPA144 and mFOLFOX6 compared with placebo and mFOLFOX6. It is estimated that the interim analysis will occur approximately 20 months from the first patient enrolled.

The primary analysis of PFS will be conducted when at least 96 PFS events have been observed in the first 156 enrolled patients, and will be performed using the intent-to-treat (ITT) population.

The primary analysis of PFS will be conducted using a stratified log-rank 2-sided test with a 0.05 level of significance. The stratification factors will be the same used to stratify the randomization schedule as documented in the interactive voice and Web response system (IXRS).

If the p-value for the stratified log-rank test is statistically significant (< 0.05 two-sided) and the HR is < 1, the null hypothesis of no difference in PFS will be rejected and it will be inferred that

PFS is statistically prolonged in the group receiving FPA144 in combination with mFOLFOX6 compared with the group receiving placebo and mFOLFOX6.

The median PFS and the associated 95% confidence interval for each treatment arm will be estimated using the Kaplan-Meier method. The hazard ratio (HR= $\lambda_{FPA144+\ mFOLFOX6}/\ \lambda_{mFOLFOX6}$) will be estimated using a Cox regression model with treatment group as the only main effect and stratifying by the same stratification factors as were used for the log-rank test. An unstratified HR will also be presented.

Analyses of secondary endpoints OS and ORR will be conducted when the primary endpoint, PFS, is tested statistically significant, and formal hypotheses OS and ORR will be tested hierarchically at a level of 0.05. The OS will be tested first and if it is significant, the ORR will be tested next. The family-wise type I error rate of testing primary and secondary endpoints will be in a control by employing this gate-keeping testing procedure at a level of 0.05.

8.3.2 Analysis of Secondary Efficacy Endpoints

There will be a planned interim and final analysis for OS if the test for PFS is statistically significant. The interim analysis of OS will be conducted at the time of primary analysis of PFS. Should OS be analyzed, the interim analysis (i.e., when at least 96 PFS events have been observed), and the final analysis (i.e., when 249 deaths have been observed) will be performed based on the ITT population.

The hypothesis testing of OS will be conducted using a stratified log-rank 2-sided test with a 0.05 level of significance. The group sequential method will be used to allocate type I error rate based on O’Brien-Fleming boundary and type II error rate based on the Gamma family with parameter -4 at the interim and final analysis of OS. Those futility and efficacy boundaries for OS are non-binding. The stratification factors will be the same used to stratify the randomization schedule as documented in the IXRS.

The median OS and the associated 95% confidence interval for each treatment arm will be estimated using the Kaplan-Meier method. The HR will be estimated using a Cox regression model with treatment group as the only main effect and stratifying by the same stratification factors as were used for the log-rank test. An unstratified HR will also be presented.

The analysis of ORR will be performed among the patients with baseline measurable disease. In the analysis of ORR, patients who don’t have any post-baseline adequate tumor assessments will be counted as non-responders. Formal hypothesis testing of ORR will be performed using the stratified Cochran-Mantel-Haenszel test (2-sided) at a level of 0.05. The stratification factors will be the same used to stratify the randomization schedule as documented in the IXRS.

8.3.3 Analysis of Exploratory Efficacy Endpoints

The exploratory efficacy endpoints include BICR assessed PFS per RECIST 1.1, duration of response in responding patients, and 1-year OS rate.

BICR assessed PFS per RECIST 1.1, defined as time from randomization date until the date of radiologic disease progression based on BICR assessment (per RECIST v.1.1) or death from any cause, whichever comes first. The analysis of BICR assessed PFS will be conducted similarly as the analysis of primary endpoint, PFS, which is based on PIs' assessment. This analysis will be considered as a sensitivity analysis of PFS to assess the robustness of treatment effect based on the PI's assessment.

Duration of response is defined, for patients with an objective response, as the time from onset of radiographic documentation of objective response to disease progression by RECIST 1.1 or death due to any cause. Median duration of response and its associated 95% CI will be estimated, by treatment group, using Kaplan-Meier methods. The difference between treatment groups will be analyzed using a stratified log-rank test, using the same stratification that was used for randomization.

The 1-year OS rate, defined as proportion of patients alive at least 1 year, will be estimated during the analysis of overall survival using the Kaplan-Meier method. The variance of proportions will be estimated using Greenwood's formula. The overall comparison for the difference in 1-year survival between the two treatment groups, will be calculated using the z-statistic where t =1 year.

The summary statistics for change from baseline in functional outcomes as measured by EQ-5D-5L and the EORTC QLQ-C30 will be presented at each post baseline assessment and at the End of Treatment. Differences between treatment groups will be analyzed using repeated measures analysis methods if applicable.

8.4 Safety Analyses

All AEs will be coded using the Medical Dictionary for Regulatory Activities (MedDRA). The Investigator will classify the severity of AEs using the CTCAE v 4.03. A treatment emergent adverse event (TEAE) is defined as any event with an onset date on or after date of first dose of study treatment, or any event present before treatment that worsens after treatment. Only TEAEs with an onset date prior to date of last dose + 28 days will be tabulated in summary tables.

Clinical laboratory data will be summarized by the type of laboratory test. The number and percentage of patients who experience abnormal (ie, outside of reference ranges) and/or clinically significant abnormalities after study treatment administration will be presented for each clinical laboratory measurement. For each clinical laboratory measurement, descriptive statistics will be provided for baseline and all subsequent post-treatment scheduled visits. Changes from baseline to the post-treatment visits will also be provided. Descriptive statistics of

vital signs will also be provided in a similar manner. In addition, shift from baseline in CTCAE grade (where applicable) and by high/low flags (where CTCAE grades are not defined) will be presented in a similar manner.

8.4.1 Safety Analyses in Phase 1

Safety analyses will be performed for patients included in the safety population. The incidence of DLTs, incidence of TEAEs, clinical laboratory abnormality (e.g. shift table), vital signs, corneal and retinal findings, and ECGs will be tabulated and summarized by dose level.

8.4.2 Safety Analyses in Phase 3

The analyses of safety will include all patients who receive any study treatment (FPA144 in combination with mFOLFOX6, or placebo and mFOLFOX6) throughout the study and provide any post-treatment safety information. The incidence of TEAEs, clinical laboratory abnormality (e.g. shift table), vital signs, corneal and retinal findings, and ECGs will be tabulated and summarized by treatment group.

No formal comparisons of safety endpoints are planned.

8.5 Pharmacokinetic Analyses

Individual and mean (±SD) serum FPA144 concentration-time data will be tabulated and plotted by dose level. FPA144 PK parameters will be estimated from the serum FPA144/IP concentration-time data from Phase 1 using a non-compartmental analysis method with IV infusion input. Alternative methods may be considered. Estimated individual and mean (±SD) PK parameters will be tabulated and summarized by dose level. Other descriptive statistics might be reported for serum FPA144 concentration-time data and estimated PK parameters. Dose proportionality, FPA144/IP accumulation, and attainment of steady state will be evaluated as data allow.

The impact of immunogenicity on FPA144 exposure will be assessed.

8.6 Interim Analyses

There will be 2 interim analyses conducted in this trial. One will be an interim analysis for PFS, at which only a futility test of PFS will be conducted, after 48 events (50% of target 96 PFS events for primary analysis of PFS) observed in the first 156 enrolled patients, to exclude HR>0.806 in PFS for the combination of FPA144 and mFOLFOX6 compared with placebo and mFOLFOX6. The other will be an interim analysis for OS if the test for PFS is statistically significant. The interim analysis of OS will be conducted at the time of primary analysis of PFS. Should OS be analyzed, analysis of OS at the interim will be performed on the ITT population, and the type I error rate at the interim analysis is determined by implementing a Lan-DeMets

O'Brien-Fleming alpha spending function depending on the fraction of information (death events) at the time of analysis.

In addition, safety data will be reviewed on a routine basis by the Sponsor and CROs' Medical Monitors. During the dose escalation stage, the Medical Monitors and Investigator(s) will review safety data from each dose cohort prior to dose escalation or de-escalation. AE data from all cycles will be presented to the Medical Monitors when available.

8.7 Changes in the Planned Analyses

If discrepancies exist between the text of the statistical analysis as planned in the protocol and the final SAP, a protocol amendment will not be issued and the SAP will prevail.

9 Ethical, Legal, and Administrative Aspects

9.1 Data Quality Assurance

The Sponsor (or designee) may conduct a site visit prior to study initiation at a site to verify the qualifications of each Investigator, inspect the site facilities, and inform the Investigator of responsibilities and for ensuring study compliance and procedures for adequate and correct documentation.

The Investigator is required to prepare and maintain adequate and accurate case histories designed to record all observations and other pertinent data for each study patient. All information recorded on the eCRFs for this study must be consistent with the patients' source documentation (i.e., medical records).

9.2 Electronic Case Report Forms and Source Documentation

All data obtained during this study should be entered into the eCRFs promptly. All source documents from which eCRF entries are derived should be placed in the patient's medical records. eCRF fields for which source documents will typically be needed include laboratory assessments, physical examination reports, nursing notes, ECG recordings, hospital records, computed tomography (CT) scans, and/or magnetic resonance imaging (MRI) reports.

The eCRFs for each patient will be checked against source documents at the study site by the site monitor.

Instances of missing or uninterpretable data will be discussed with the Investigator for resolution.

9.3 Access to Source Data

During the study, a monitor will perform routine site visits to review protocol compliance, compare eCRFs and individual patient's medical records, assess drug accountability, and ensure that the study is being conducted according to pertinent regulatory requirements. eCRF entries

will be verified with source documentation. The review of medical records will be performed in a manner to ensure that patient confidentiality is maintained.

In accordance with ICH GCP guidelines, the Investigator must ensure provision of sufficient time, reasonable space, and adequate qualified personnel for the monitoring visits. The visits are for the purpose of verifying adherence to the study protocol and the completeness, consistency, and accuracy of data entered on the eCRF and other documents. Moreover, regulatory authorities, IRBs, IECs, and/or the Sponsor's Quality Assurance group may wish to carry out such source data checks and/or on-site audit inspections. Direct access to source data will be required for these inspections and audits; they will be carried out giving due consideration to data protection and medical confidentiality. The Investigator assures that the Sponsor and/or Sponsor's designee will receive the necessary support to complete these activities.

All participating centers should take particular care in ensuring that original imaging source data (CT images, MRI images, echo images, etc.) are maintained and accessible for monitoring, and that these original source data are then archived on a long-term basis in compliance with ICH GCP Section 8. These images must be stored in a secure location until the Sponsor or Sponsor's designee authorizes their destruction, and must be retrievable by study patient number in the event of an audit.

9.4 Data Processing

The Data Management Plan, to be developed during the initiation phase of the study, will include specifications for consistency and plausibility checks on data and will also include data-handling rules for obvious data errors. All processes for data processing and query handling will be described in the Data Management Plan.

9.5 Archiving Study Records

Each study site will maintain a study file, which should contain, at minimum, the Investigator's Brochure, the protocol and any amendments, the protocol for tissue sampling, drug accountability records, correspondence with the IEC/IRB and the Sponsor (or designee), and other study-related documents.

The Investigator agrees to keep records and those documents that include (but are not limited to) the identification of all participating patients, medical records, study-specific source documents, source worksheets, all original signed and dated ICFs, copies of all eCRFs, query responses, and detailed records of drug disposition to enable evaluations or audits from regulatory authorities and the Sponsor or its designees.

The Investigator shall retain records required to be maintained for a period of 5 years following the date a marketing application in an ICH region is approved for the drug for the indication for which it is being investigated or, if no application is to be filed or if the application is not approved for such indication, until at least 5 years after the investigation is discontinued.

However, these documents should be retained for a longer period if required by the applicable regulatory requirement(s) or if needed by the Sponsor. In addition, the Investigator must make provision for the patients' medical records to be kept for the same period of time.

No data should be destroyed without the agreement of the Sponsor. Should the Investigator wish to assign the study records to another party or move them to another location, the Sponsor must be notified in writing of the new responsible person and/or the new location.

Patients' medical records and other original data will be archived in accordance with the archiving regulations or facilities of the investigational site.

9.6 Good Clinical Practice

The procedures set out in this study protocol are designed to ensure that the Sponsor and Investigator abide by GCP guidelines of the ICH and the Declaration of Helsinki (1989). The study also will be carried out in compliance with local legal requirements.

9.7 Informed Consent

All information about the clinical study, including the patient information and the ICFs, is prepared and used for the protection of the human rights of the patient per ICH GCP guidelines and the Declaration of Helsinki.

The ICFs, prepared by the Investigator with the assistance of the Sponsor, must be approved along with the study protocol by the IEC/IRB and be acceptable to the Sponsor before each patient is enrolled on the study; written informed consent will be obtained according to the regulatory and legal requirements. Copies of the signed ICFs will be retained by the patient and the original will be filed in the Investigator's study center file, unless otherwise agreed. The Investigator will not undertake any investigation specifically required only for the clinical study until valid consent has been obtained. The terms of the consent and when it was obtained must be documented in the source documents and in the eCRF.

If a protocol amendment is required, the ICFs may need to be revised to reflect the changes to the protocol. If the consent form is revised, it must be reviewed and approved by the appropriate IRB/IEC and signed by all patients subsequently enrolled in the study as well as those currently enrolled in the study.

9.8 Protocol Approval and Amendment

Before the start of the study, the study protocol and/or other relevant documents will be approved by the IRB/IEC, in accordance with local legal requirements. The Sponsor, Sponsor's agents, and Investigator must ensure that all ethical and legal requirements have been met before the first patient is enrolled in the study.

This protocol is to be followed exactly. To alter the protocol, amendments must be written, receive approval from the appropriate personnel, and receive IRB/IEC approval prior to implementation (if appropriate). Following approval, the protocol amendment will be submitted to the investigational new drug (IND) application under which the study is being conducted.

All amendments will be distributed to all protocol recipients, with appropriate instructions. Administrative changes (not affecting the patient benefit/risk ratio) may be made without the need for a formal amendment. Administrative changes will be distributed to the Investigator and others as appropriate.

9.9 Study Committee and Central Review

9.9.1 Cohort Review Committee (CRC)

The phase I safety run-in for this study will have a CRC consisting of representatives from the Sponsor, the Clinical Research Organization (CRO) and one or more Investigators from actively participating sites. The CRC will assess the safety of the dose escalation (phase I) of the study on a regular basis. All dose escalation decisions will be based on assessment of DLTs, overall safety and tolerability and will be made after the last patient enrolled in each cohort has completed the first treatment cycle. Dose escalation decisions will be agreed upon by the CRC.

9.9.2 Data Monitoring Committee

The study will have a Data Monitoring Committee (DMC) that will operate independently from the Sponsor and the clinical investigators. The primary responsibilities of the DMC are to review the accumulating safety data from the phase 3 study on a regular and ad hoc basis, to review the results of an interim PFS efficacy analysis for futility, and to make recommendations to the Sponsor regarding the continued conduct of the study. Safety data from the phase 3 study will be provided at regular intervals to the DMC in the form of unblinded summary reports and/or data listings from an independent statistical center designated by the Sponsor.

The first meeting of the DMC is planned to occur after a minimum number of patients (ie, 50 patients) have had the opportunity to complete a cycle of study treatment. Subsequent meetings are planned to occur periodically (eg, quarterly) and ad hoc at the request of the DMC members or the Sponsor. Details regarding DMC membership, schedule and format of meetings, format for presentation of data, access to interim data, method and timing of providing interim reports to the DMC, and other issues relevant to committee operations will be described in the DMC charter.

9.9.3 Blinded Independent Central Review (BICR)

A Blinded Independent Central Review (BICR) committee will be established to evaluate the concordance between investigator assessed PFS and BICR assessed PFS. An IRC charter will be in place to guide and direct decisions based on BICR read(s).

The study will use investigator-assessed PFS as a primary outcome measure, and since the study will be executed globally at multiple institutions (at least 250), the use of BICR will assist in minimizing bias. The BICR will collect, hold and be ready to analyze a proportion of patient radiographic images. It is proposed to assess the differential discordance, defined as the difference in discordance rates between treatment arms (mechanism that induces bias). A large magnitude of differential discordance between the treatment arms can be associated with divergent estimates of treatment effect between the investigator's assessment and BICR. Since the study design uses a hazard ratio (HR) of 0.5, it is proposed that a proportion of patients' scans (eg, 30% of patients]) will be reviewed by BICR to assess the discordance between the Investigators and BICR prior to the primary analysis of PFS. Discordance will be defined in the charter with respect to degree of difference requiring a BICR analysis.

The degree of differential discordance identified will dictate the use of the BICR review. If the differential discordance is not large, the remaining scans will not be read by the BICR at the primary analysis of PFS. Alternatively, if a large magnitude of differential discordance is identified prior to the primary analysis of PFS, the BICR will review scans for 100% of the first 156 patients enrolled, and PFS will be evaluated both by investigator assessment and BICR. If the differential discordance is confirmed, the Sponsor will use BICR-assessed PFS as the primary endpoint and will revise the SAP accordingly before database lock.

9.10 Long-term Follow-up

Patients in Phase 3 will undergo long-term follow-up for survival by clinic visit or by telephone approximately every 3 months ± 28 days after the EOT follow-up visit until up to 24 months after the last patient is enrolled into the study. Patients should be followed until death, loss to follow-up, withdrawal of consent, or study termination by the Sponsor (whichever occurs first). During the Follow-up Period, if the patient undergoes anti-cancer therapy, this should be documented.

For the first 6 months of the Long-term Follow-up Period (for patients in Phase 3), and for the first 6 months after the EOT visit (for patients in Phase 1), any known pregnancy that occurs should be reported to the Sponsor and followed as described in Section 6.1.

9.11 Premature Termination of the Study

If the Investigator, Sponsor, or Medical Monitor becomes aware of conditions or events that suggest a possible hazard to patients if the study continues, the study may be terminated. The study may also be terminated early at the Sponsor's discretion in the absence of such a finding.

Conditions that may warrant termination include, but are not limited to:

- Discovery of an unexpected, significant, or unacceptable risk to the patients enrolled in the study

- Failure to enroll patients at an acceptable rate
- Decision on the part of the Sponsor to suspend or discontinue development of the drug

9.12 Confidentiality

All study findings and documents will be regarded as confidential. The Investigator and members of his/her research team must not disclose such information without prior written approval from the Sponsor.

The anonymity of participating patients must be maintained. Patients will be identified on eCRFs and other documents submitted to the Sponsor or Sponsor's designee by their patient number, initials, and/or birth date. Study patients are not to be identified by name, and any information sent to the Sponsor or Sponsor's designee should have patient identifiers redacted, and replaced with patient ID. Documents that include the name of the patient (e.g., the signed informed consent) must be maintained in confidence by the Investigator. The Investigator further agrees to take all reasonable precautions to prevent the disclosure by any employee or agent of the study center to any third party or otherwise into the public domain.

9.13 Other Ethical and Regulatory Issues

If a significant safety issue is identified, either from an individual case report or review of aggregate data, then the Sponsor will issue prompt notification to applicable regulatory authorities and Investigators. Investigators will then notify local IRB/IECs as deemed appropriate based on individual IRB/IEC policy.

A significant safety issue is one that has a significant impact on the course of the clinical trial or program (including the potential for suspension of the trial program or amendments to protocols) or warrants immediate update of informed consent.

10 References

Ahn, S. , J. Lee, M. Hong, *et al.* "FGFR2 in Gastric Cancer: Protein Overexpression Predicts Gene Amplification and High H-Index Predicts Poor Survival." *Modern Pathology* (2016): 1-9.

Al-Batran, S-E. , A. Atmaca, S. Hegewisch-Becker, *et al.* "Phase II Trial of Biweekly Infusional Flourouracil, Folinic Avid, and Oxaliplatin in Patients with Advanced Gastric Cancer." *Journal of Clinical Oncology* 22, no. 4 (2004): 658-63.

Al-Batran, S-E. , J. T. Hartmann, R. Hofheinz, *et al.* "Biweekly Fluorouracil, Leucovorin, Oxaliplation. and Docetaxel (FLOT) for Patients with Metastatic Adenocarcinoma of the Stomach or Esophagogastric Junction: A Phase II Trial of the Arbeitsgemeinschaft Internistische Onkologie ". *Annals of Oncology* 19 (2008): 1882-87.

Andre, Fabrice, Malcolm Ranson, Emma Dean, *et al.* "Abstract LB-145: Results of a Phase I Study of AZD4547, an Inhibitor of Fibroblast Growth Factor Receptor (FGFR), in Patients with Advanced Solid Tumors." Paper presented at the AACR 104th Annual Meeting 2013, Washington, DC, 2013.

Bang, Y., E. Cutsem, A. Feyereislova, *et al.* "Trastuzumab in Combination with Chemotherapy Versus Chemotherapy Alone for Treatment of HER2-Positive Advanced Gastric or Gastro-Oesophageal Junction Cancer (ToGA): A Phase 3, Open-Label, Randomised Controlled Trial." *The Lancet* 376 (2010): 687-97.

Bron, A., VE Evans, and JA Smith. "Grading of Corneal and Conjuctival Staining in the Context of Other Dry Eye Tests." *Cornea* 22, no. 7 (2003): 640-50.

Brown, Alan P., Cynthia L. Courtney, Lena M. King, *et al.* "Cartilage Dysplasia and Tissue Mineralization in the Rat Following Administration of a FGF Receptor Tyrosine Kinase Inhibitor." *Toxicologic Pathology* 33 (2005): 449-55.

Catenacci, D. , S. Rha, Y-J. Bang, *et al.* "Updated AntiTumor Activity and Safety of FPA144, and ADCC-Enhanced Isoform-Specific Monoclonal Antibody, in Patients with FGFR2b+ Gastric Cancer." *Journal of Clinical Oncology* 35, no. 15 (2017): 4067.

Chao, Y. , K.H. Yeh, C. Chang, *et al.* "Phase II Study of Weekly Oxaliplatin and 24-Hour Infusion of High-Dose 5-Fluorouracil and Folinic Acid in the Treatment of Advanced Gastric Cancer." *British Journal of Cancer* 91 (2004): 453-58.

Cheeseman, S. , S. Joel, J. Chester, *et al.* "A 'Modified de Gramont' Regimen of Fluorouracil, Alone and With Oxaliplatin, for Advanced Colorectal Cancer." *British Journal of Cancer* 87 (2002): 393-99.

Chen, J. , D. Hunter, M. Stampfer, *et al.* "Polymorphism in the Thymidylate Synthase Promoter Enhancer Region Modifies the Risk and Survival of Colorectal Cancer." *Cancer Epidemiology, Biomarkers & Prevention* 12 (2003): 958-62.

De Vita, F. , M. Orditura, E. Matano, *et al.* "A Phase II Study of BiWeekly Oxaplatin Plus Infusional 5-Fluorouracil and Folinic Acid (FOLFOX-4) as First-Line Treatment of Advanced Gastric Cancer Patients." *British Journal of Cancer* 92 (2005): 1644-49.

Deshpande, Amit M., Servando Palencia, David I. Bellovin, *et al.* "Expression of FGFR2b in Gastric Cancer as Measured by Immunohistochemistry With a Highly Specific Monoclonal Antibody." Paper presented at the American Association for Cancer Research San Diego, CA, 2014.

Dienstmann, Rodrigo, Rastilav Bahleda, Adamo. Barbara, *et al.* "First in human study of JNJ-42756493, a potent pan fibroblast growth factor receptor (FGFR) inhibitor in patients with advanced solid tumors." Paper presented at the AACR 105th Annual Meeting, San Diego, CA, 2014.

Ford, Hugo, Andrea Marshall, John Bridgewater, *et al.* "Docetaxel Versus Active Symptom Control for Refractory Oesophagogastric Adenocarcinoma (COUGAR-02): An Open-Label, Phase 3 Randomised Controlled Trial." *Lancet Oncology* 15 (2014): 78-86.

Fuchs, C. , J. Tomasek, C. Yong, *et al.* "Ramucirumab Monotherapy for Previously Treated Advanced Gastric or Gastro-Oesophageal Junction Adenocarcinoma (REGARD): an International, Randomised, Multicentre, Placebo-Controlled, Phase 3 Trial." *Lancet Oncology* 383 (2014): 31-39.

Gemo, Abigael , Amit Deshpande, Servando Palencia, *et al.* "FPA144: A Therapeutic Antibody for Treating Ptients with Gastric Cancers Bearing FGFR2 Gene Amplification." Paper presented at the AACR 105th Annual Meeting 2014, San Diego California, 2014.

GlaxoSmithKline. "Study to Evaluate GSK3052230 in Combination with Paclitaxel and Carboplatin, or Docetaxel or as Single Agent in Subjects with Solid Malignancies and Deregulated Fibroblast Growth Factor (FGF) Pathway Signaling " https://clinicaltrials.gov/ct2/show/NCT01868022?term=NCT01868022&rank=1.

Goode, E. , and E. Smyth. "Immunotherapy for Gastroesophageal Cancer." *Journal of Clinical Medicine* 5, no. 84 (2016): 1-14.

Gravalos, C. , and A. Jimeno. "HER2 in Gastric Cancer: A New Prognostic Factor and a Novel Therapeutic Target." *Annals of Oncology* 19 (2008): 1523-29.

Hall, T. , Y. Yu, S. Eathiraj, *et al.* "Preclinical Activity of ARQ 087, a Novel Inhibitor Targeting FGFR Dysregulation." *PLoS ONE* 11, no. 9 (2016): 1-19.

Hattori, Yutaka, Hiroshi Itoh, Shinya Uchino, *et al.* "Immunohistochemical Detection of K-sam Protein in Stomach Cancer." *Clinical Cancer Research* 2 (1996): 1373-81.

Hattori, Yutaka, Hiroki Odagiri, Hiroshi Nakatani, *et al.* "K-sam, an Amplified Gene in Stomach Cancer, is a Member of the Heparin-Binding Growth Factor Receptor Genes." *Proceedings of the National Academy of Sciences (PNAS)* 87 (1990): 5983-87.

Hecht, J. , Y. Bang, S. Qin, *et al.* "Lapatinib in Combination with Capecitabine Plus Oxaliplatin in Human Epidermal Growth Factor Receptor 2-Positive Advanced or Metastatic Gastric, Esophageal, or Gastroesophageal Adenocarcinoma: TRIO-013/LOGiC-A Randomized Phase III Trial." *Journal of Clinical Oncology* 34, no. 5 (2016): 443-51.

Herdman, M., C. Gudex, A. Lloyd, *et al.* "Development and Preliminary Testing of the New Five-Level Version of EQ-5D (EQ-5D-5L)." *Quality of Life Research* 20 (2011): 1727-36.

Hironaka, S. , S. Ueda, H. Yasui, *et al.* "Randomized, Open-Label, Phase III Study Comparing Irinotecan with Paclitaxel in Patients with Advanced Gastric Cancer Without Severe Peritoneal Metastasis After Failure of Prior Combination Chemotherapy Using Flouropyrimidine Plus Platnum: WJOG 4007 Trial." *Journal of Clinical Oncology* 31, no. 31 (2013): 4438-44.

Hochster, H. , L. Hart, R.K. Ramanathan, *et al.* "Safety and Efficacy of Oxaliplatin and Fluoropyrimidine Regimens with or without Bevacizumab As First-Line Treatment of Metastatic Colorectal Cancer: Results of the TREE Study." *Journal of Clinical Oncology* 26, no. 21 (2008).

Hofmann, M. , O. Stoss, D. Shi, *et al.* "Assessment of a HER2 Scoring System for Gastric Cancer: Results from a Validation Study." *Histopathology* 52 (2008): 797-805.

Jung, E. , E. Jung , S. Min, *et al.* "Fibroblast Growth Factor Receptor 2 Gene Amplification Status and its Clinicopathologic Significance in Gastric Carcinoma." *Human Pathology* 43, no. 10 (2012): 1559-66.

Kamangar, F. , G. Dores, and W. Anderson. "Patterns of Cancer Incidence, Mortality, and Prevalence Across Five Continents: Defining Priorities to Reduce Cancer Disparities in Different Geographic Regions of the World." *Journal of Clinical Oncology* 24, no. 14 (2006): 2137-50.

Kanagavel, D. , M. Fedyanin, A. Tryakin, *et al.* "Second-Line Treatment of Metastatic Gastric Cancer: Current Options and Future Directions." *World Journal of Gastroenterology* 21, no. 41 (2015): 11621-35.

Kang, Y-K. , W-K. Kang, D-B. Shin, *et al.* "Capecitabine/Cisplatin Versus 5-Fluorouracil/Cisplatin as First-Line Therapy in Patients with Advanced Gastric Cancer: A Randomised Phase III Noninferiority Trial." *Annals of Oncology* 20 (2009): 666-73.

Keam, B. , S-A. Im, S-W. Han, *et al.* "Modified FOLFOX-6 Chemotherapy in Advanced Gastric Cancer: Results of Phase II Study and Comprehensive Analysis of Polymorphisms as A Predictive and Prognostic Cancer." *BMC Cancer* 8, no. 148 (2008): 1-10.

Kelsen, D. , O. Atiq, L. Saltz, *et al.* "FAMTX Versus Etoposide, Doxorubicin, and Cisplatin: A Random Assignment Trial in Gastric Cancer." *Journal of Clinical Oncology* 10, no. 4 (1992): 541-48.

Kim, D. , J. Kim, S-H. Lee, *et al.* "Phase II Study of Oxaliplatin, 5- Fluorouracil and Leucovorin in Previously Platinum-Treated Patients with Advanced Gastric Cancer." *Annals of Oncology* 14 (2003): 383-87.

Kim, N. , Y. Park, D. Heo, *et al.* "A Phase III Randomized Study of 5-Fluorouracil and Cisplatin Versus 5- Fluorouracil, Doxorubicin, and Mitomycin C Versus 5- Fluorouracil Alone in the Treatment of Advanced Gastric Cancer." *Cancer* 71, no. 12 (1993): 3813-18.

Koizumi, W. . "Chemotherapy for Advanced Gastric Cancer: Review of Global Japanese Status." *Gastrointest Cancer Res* 1, no. 5 (2007): 197-203.

Li, Bohua, Lei Zhao, Huaizu Guo, *et al.* "Characterization of a Rituximab Variant with Potent Antitumor Activity Against Rituximab-Resistant B-Cell Lymphoma." *Blood* 114, no. 24 (2009): 5007-15.

Li, Jin, Shukui Qin, Jianming Xu, *et al.* "Randmized, Double-Blind, Placebo-Controlled Phase III Trial of Apatinib in Patients with Chemotherapy-Refractory Advanced or Metastitic Adenocarcinoma of the Stomach or Gastroesophageal Junction." *Journal of Clinical Oncology* 34, no. 14 (2016): 1448-54.

Lordick, F. , S. Lorenzen, J. Stollfuss, *et al.* "Phase II Study of Weekly Oxaplatin Plus Infusional Fluorouracil and Folinic Acid (FUFOX Regimen) as First-Line Treatment in Metastatic Gastric Cancer." *British Journal of Cancer* 93 (2005): 190-94.

Louvet, C. , T. Andre, J. Tigaud, *et al.* "Phase II Study of Oxaplatin, Fluorouracil, and Folinic Acid in Locally Advanced or Metastatic Gastric Cancer Patients." *Journal of Clinical Oncology* 20, no. 23 (2002): 4543-48.

MacDonald, J. , P. Schein, P. Woolley, *et al.* "5-Fluorouracil, Doxorubicin, and Mitomycin (FAM) Combination Chemotherapy for Advanced Gastric Cancer." *Annals of Internal Medicine* 93, no. 4 (1980): 533-36.

Matsumoto, K. , T. Arao, T. Hamaguchi, *et al.* "FGFR2 Gene Amplification and Clinicopathological Features in Gastric Cancer ". *British Journal of Cancer* 106, no. 4 (2012): 727-32.

Miki, Toru, Donald P. Bottaro, Timothy P. Fleming, *et al.* "Determination of Ligand-Binding Specificity by Alternative Splicing: Two Distinct Growth Factor Receptors Encoded by a Single Gene." *Proceedings of the National Academy of Sciences* 69 (1992): 246-50.

National Cancer Institute. "SEER Stat Fact Sheets: Esophageal Cancer." http://seer.cancer.gov/statfacts/html/esoph.html.

Okines, A. , A. Norman, P. McCloud, *et al.* "Meta-Analysis of the REAL-2 and ML17032 Trials: Evaluating Capecitabine-Based Combination Chemotherapy and Infused 5-Fluorouracil-Based Combination Chemotherapy for the Treatment of Advanced Oesphago- Gastric Cancer." *Annals of Oncology* 20, no. 9 (2009): 1529-34.

Powers, J. , S. Palencia, S. Foy, *et al.* "FPA144, A Therapeutic Monoclonal Antibody Targeting the FGFR2b Receptor, Promotes Antibody Dependent Cell-Mediated Cytotoxicity and Stimulates Sensitivity to PD-1 Blockade in the 4T1 Syngeneic Tumor Model in Mice." (2016): 1.

Pullarkat, S. , J. Stoehlmacher, V. Ghaderi, *et al.* "Thymidylate Synthase Gene Polymorphism Determines Response and Toxicity of 5-FU Chemotherapy." *The Pharmacogenics Journal* 1 (2001): 65-70.

Seo, S. , S. Park, M. Ryu, *et al.* "Prognostic Impact of Fibroblast Growth Factor Receptor 2 Gene Amplification in Patients Receiving Fluoropyrimidine and Platinum Chemotherapy for Metastatic and Locally Advanced Unresectable Gastric Cancers." *Oncotarget* (2016): 1-11.

Sequist, Lecia V., Phillippe Cassier, Andrea Varga, *et al.* "Phase I Study of BGJ398, a Selective Pan-FGFR Inhibitor in Genetically Preselected Advanced Solid Tumors." Paper presented at the AACR 2014 Annual Meeting, San Diego, CA, 2014.

Shinkawa, Toyohide, Kazuyasu Nakamura, Naoko Yamane, *et al.* "The Absence of Fucose but Not the Presence of Galactose or Bisecting N-Acetylglucosamine of Human IgG1 Complex-type Oligosaccharides Shows the Critical Role of Enhancing Antibody-dependent Cellular Cytotoxicity." *THe Journal of Biological Chemisty* 278, no. 5 (2003): 3466-73.

Smyth, E. , M. Verheij, W. Allum, *et al.* "Gastric Cancer: ESMO Clinical Practice Guidelines for Diagnosis, Treatment, and Follow-up ". *Annals of Oncology* 27, no. 5 (2016): v38-v49.

Su, X. , P. Zhan, P. Gavine, *et al.* "FGFR2 Amplification has Prognostic Significance in Gastric Cancer: Results From a Large International MultiCentre Study ". *British Journal of Cancer* 110 (2014): 967-75.

Taiho Oncology Inc. "A Dose Finding Study Followed by a Safety and Efficacy Study in Patients with Advanced Solid Tumors or Multiple Myeloma with FGF/FGFR-Related Abnormalities." https://clinicaltrials.gov/ct2/show/NCT02052778?term=NCT02052778&rank=1.

Teva Parenteral Medicines Inc. "ADRUCIL- Highlights of Prescribing Information." (2016): 1-12.

Teva Parenteral Medicines Inc. "Leucovorin Calcium for Injection Package Insert." (2014): 1-13.

Teva Pharmaceuticals USA. "Oxaliplatin: Highlights of Prescribing Information." (2012): 1-43.

Teva Pharmaceuticals USA Inc. "Warfarin Sodium Highlights of Prescribing Information." (2016): 1-51.

The GASTRIC Group. "Benefit of Adjuvant Chemotherapy for Resectable Gastric Cancer." *JAMA* 303, no. 1729-1737 (2010): 1729.

Thuss-Patience, P., A. Kretzschmar, D. Bichev, *et al.* "Survival Advantage for Irinotecan Versus Best Supportive Care as Second-Line Chemotherapy in Gastric Cancer- A Randosmied Phase III Study of the Arbeitsgemeinschaft Internistische Onkologie (AIO)." *European Journal of Cancer* 47 (2011): 2306-14.

Tournigand, C. , T. Andre, E. Achille, *et al.* "FOLFIRI Followed by FOLFOX6 or the Reverse Sequence in Advanced Colorectal Cancer: A Randomized GERCOR Study." *Journal of Clinical Oncology* 22, no. 2 (2004): 229-37.

Turner, N. , and R. Grose. "Fibroblast Growth Factor Signalling: From Development to Cancer." *Nature* 10 (2010): 116-29.

Ulrich, C. , J. Bigler, C. Velicer, *et al.* "Searching Expressed Sequence Tag Databases: Discovery and Confirmation of a Common Polymorphism in the Thymidylate Synthase Gene." *Cancer Epidemiology, Biomarkers & Prevention* 9 (2000): 1381-85.

van Hazel, G. , V. Heinemann, N. Sharma, *et al.* "SIRFLOX: Randomized Phase III Trial Comparing First-Line mFOLFOX6 (Plus or Minus Bevacizumab) Versus mFOLFOX6 (Plus or Minus Bevacizumab) Plus Selective Internal Radiation Therapy in Patients with Metastatic Colorectal Cancer." *Journal of Clinical Oncology* 34, no. 15 (2016): 1723-31.

Vanhoefer, U. , P. Rougier, H. Wilke, *et al.* "Final Results of a Randomized Phase III Trial of Sequential High-Dose Methotrexate, Fluorouracil, and Doxorubicin Versus Etoposide, Leucovorin, and Fluorouracil Versus Infusional Fluorouracil and Cisplatin in Advanced Gastric Cancer: A Trial of the European Organization for Research and Treatment of Cancer Gastrointestinal Tract Cancer Cooperative Group." *Journal of Clinical Oncology* 18, no. 14 (2000): 2648-57.

Waddell, T. , M. Verheij, W. Allum, *et al.* "Gastric Cancer: ESMO-ESSO-ESTRO Clinical Practice Guidelines for Diagnosis, Treatment, and Follow-up ". *Radiotherapy and Oncology* 110 (2014): 189-94.

Wagner, A. , W. Grothe, J. Haerting, *et al.* "Chemotherapy in Advanced Gastric Cancer: A Systematic Review and Meta-Analysis Based on Aggregrate Date." *Journal of Clinical Oncology* 24, no. 18 (2006): 2903-09.

Webb, A. , D. Cunningham, H. Scarffe, *et al.* "Randomized Trial Comparing Epirubicin, Cisplatin, and Fluorouracil Versus Fluorouracil, Doxorubicin, and Methotrexate in Advanced Esophagogastric Cancer." *Journal of Clinical Oncology* 15, no. 1 (1997): 261-67.

Wilke, H. , K. Muro, E. Custem, *et al.* "Ramucirumab Plus Paclitaxel Versus Placebo Plus Paclitaxel in Patients with Previously Treated Advanced Gastric or Gastro-Oesophageal Junction Adenocarcinoma (RAINBOW): A Double-Blind, Randomised Phase 3 Trial." *Lancet Oncology* 15 (2014): 1224-35.

Wils, J. , H. Klein, D. Wagener, *et al.* "Sequential High-Dose Methotrexate and Fluorouracil Combined with Doxorubicin- A Step Ahead in the Treatment of Advanced Gastric Cancer: A Trial of the European Organizaion for Research and Treatment of Cancer Gastrointestinal Tract Cooperative Group." *Journal of Clinical Oncology* 9, no. 5 (1991): 827-31.

Wohrer, S. , M. Raderer, and M. Hejna. "Palliative Chemotherapy for Advanced Gastric Cancer." *Annals of Oncology* 15 (2004): 1585-95.

Wu, Yi-Mi, Fengyun Su, Shanker Kalyana-Sundaram, *et al.* "Identifi cation of Targetable FGFR Gene Fusions in Diverse Cancers." *Cancer Discovery* 3, no. 6 (2013): 636-47.

Zhao, Xiaoxian, Shweta Singh, Cecile Pardoux, *et al.* "Targeting C-type Lectin-Like Molecule-1 for Antibody-Mediated Immunotherapy in Acute Myeloid Leukemia." *Haematologica* 95, no. 1 (2010): 71-78.

11 Appendices

The following appendices are provided for this protocol.

Appendix 1: Formula to Calculate Creatinine Clearance 121
Appendix 2: Schedule of Assessments – Dose-Escalation Safety Run-in (Phase 1)........122
Appendix 3: Schedule of Assessments –Randomized, Placebo-controlled Portion (Phase 3).125
Appendix 4: Laboratory Evaluations........129
Appendix 5: Study Flowchart for Pharmacokinetic, Immunogenicity, and Pharmacodynamic Blood Sample Collections for Phase 1........130
Appendix 6: Study Flowchart for Pharmacokinetic and Immunogenicity Blood Sample Collections for Phase 3........131
Appendix 7: ECOG Performance Status........132
Appendix 8: Fluorescein Stain Grading Systems........133

Appendix 1: Formula to Calculate Creatinine Clearance

Creatinine Clearance = Sex * ([140 – Age] / [Serum Creatinine]) * (Weight / 72)

Appendix 2: Schedule of Assessments – Dose-Escalation Safety Run-in (Phase 1)

	Screening	Study Treatment: Cycles 1 and 2					Study Treatment: Cycle 3, Cycle 4 and Subsequent Cycles				
	**Day --28 to Day 0**	**Cycle 1 Day 1**	**Cycle 1 Day 3**	**Cycle 1 Day 8**	**Cycle 2 Day 1**	**Cycle 2 Day 3**	**Cycle 3[b] Day 1**	**Cycle 4[c] Day 1**	**As Clinically Indicated**	**Unique Schedules**	**EOT[d,e]**
**Procedure[a]**	Week 0	Week 1	Week 1	Week 2	Week 3	Week 3	≥Week 4				
IHC analysis of FGFR2b expression[f]	X										
Sample for ctDNA blood assay[g]		X									
Informed Consent[h]	X										
Review/Confirm Eligibility Criteria	X	X									
Medical/Oncology and Medication History	X	X									
Demography/Baseline Characteristics	X										
Physical Examination[i,j]	X	X		X	X		X	X			X
ECOG Performance Status	X						X[k]				X
Vital Signs[l]	X	X	X	X	X	X	X	X	X		X
12-lead ECG[m]	X								X		X
Comprehensive Ophthalmologic Examination[n]	X						X		X		X
Slit Lamp Examination[o]										X	
Clinical Safety Laboratory Sampling[p]	X	X		X	X		X	X	X		X
Pregnancy Test[q]	X	X[q]					X[q]			X	X[q]
Urinalysis[r]	X	X					X			X	X

Procedure[a]	Screening	Study Treatment: Cycles 1 and 2					Study Treatment: Cycle 3, Cycle 4 and Subsequent Cycles				
	Day --28 to Day 0	Cycle 1 Day 1	Cycle 1 Day 3	Cycle 1 Day 8	Cycle 2 Day 1	Cycle 2 Day 3	Cycle 3[b] Day 1	Cycle 4[c] Day 1	As Clinically Indicated	Unique Schedules	EOT[d,e]
	Week 0	Week 1	Week 1	Week 2	Week 3	Week 3	≥Week 4				
PK Samples[s]		X	X	X	X	X	X			X	X
Immunogenicity Sampling[t]		X			X		X			X	X
Biomarker Assessment Sampling[u]		X	X				X			X	X
FPA144 Administration[v]		X			X		X	X			
mFOLFOX6 Administration[w]		X			X		X	X			
Adverse Events[x]	X	X ——————————————————————— X									
Concomitant Medications		X ——————————————————————— X									

[a] Unless specified, procedure is to be completed within ± 72 hours of scheduled time point and to be synchronized with the study treatment administration day.

[b] And subsequent odd cycles from Cycle 3 (except for pharmacokinetic, immunogenicity, and pharmacodynamic blood sample collections, which are described in Appendix 5).

[c] And subsequent even cycles from Cycle 4 (except for pharmacokinetic, immunogenicity, and pharmacodynamic blood sample collections, which are described in Appendix 5.

[d] End of Treatment (EOT) assessments should be performed 28 (±3) days following the last study treatment administration

[e] For the first 6 months after the EOT visit, any pregnancy that occurs should be reported to the Sponsor.

[f] Sample for retrospective IHC analysis of FGFR2b expression prior to enrollment.

[g] Sample for ctDNA blood assay will be collected prior to the first dose of study treatment (Cycle 1 Day 1) and analysed retrospectively for *FGFR2* amplification.

[h] Written, signed informed consent must be collected prior to any study-specific procedures. The most recent IRB/EC approved ICF must be signed.

[i] Complete physical examination and height will be measured at Screening only. Limited physical examinations should be conducted, including examination of the oropharynx, thereafter.

[j] After Cycle 1, the FPA144 dose will be recalculated at each infusion visit only if weight has changed >10% from Cycle 1, Day 1.

[k] ECOG Performance Status will be assessed at Cycle 3 Day 1 and Day 1 of every other subsequent cycle (odd cycles) until the EOT visit.

[l] Vital signs (blood pressure, pulse, respiration, and temperature[°C]) are to be measured at screening visit following 5 minutes of rest and on Cycle 1 Day 1 at the following time points: pre-dose, and 0.5, 1, 2, and 4 hours from the start of the FPA144 infusion. On subsequent dosing days, pre-dose and at 0.5, 1, and 2 hours from

the start of FPA144 infusion.

[m] With patient resting for 5 minutes prior to recording.

[n] Comprehensive ophthalmologic examinations (conducted at Screening, prior to Cycle 3 Day 1, and at the EOT visit only) include fundoscopic and slit lamp exam, ocular coherence tomography (OCT), visual acuity, completion of fluorescein staining score form, determination of intraocular pressure, and review of ocular/visual symptoms. The comprehensive ophthalmologic examination will be repeated at any point if changes in visual acuity or visual symptoms are reported by patients.

[o] Slit lamp examinations, (with completion of fluorescein staining score form), should be conducted for all patients every 8 weeks from Cycle 3 Day 1 through completion of the study. If the patient comes off study but has any persistent corneal findings, the assessments should continue every–8 weeks until resolution of findings, withdrawal of consent, death, or loss to follow up. OCT is not required at these time points.

[p] Blood tests (evaluated by local laboratories) are listed in Appendix 4. Baseline hematology and blood chemistry test results must be obtained within 96 hours of study treatment administration on Cycle 1 Day 1 to confirm eligibility. On subsequent dosing days, hematology and blood chemistry results must be obtained within 72 hours prior to dosing. Coagulation samples need to be obtained at baseline, at Cycles 1 through 4, and at any time clinically indicated (e.g., patients on anticoagulant therapy requiring close monitoring).

[q] Serum ß-hCG (evaluated by local laboratories) will be performed on all women of childbearing potential prior to Cycle 1 Day 1 and at EOT. On dosing days of odd cycles (every other cycle), urine pregnancy results must be available within 72 hours prior to dosing. If serum B-hCG test is performed at screening and is not within 96 hrs of C1D1, the test must be repeated to confirm negative results.

[r] Includes dipstick for protein, glucose, blood, pH, and ketones on dosing days of odd cycles (every other cycle). If dipstick findings are abnormal, then a microscopic evaluation will be performed to assess the abnormal findings.

[s] Blood samples for PK analysis. Refer to Appendix 5 for collection times.

[t] Blood samples for anti-FPA144 antibodies. Refer to Appendix 5 for collection times.

[u] Blood samples for exploratory biomarker analysis of the FGFR pathway: refer to Appendix 5 for collection times.

[v] FPA144 is administered every 14 days as a 30 minute infusion starting cycle 1, day1. The first 3 doses (cycles) of FPA144 should be administered every 14 days (±3 days) regardless of delays in mFOLFOX6 treatment. After the first 3 doses, FPA1444 may be delayed up to ±7 days to be synchronized with administered mFOLFOX6.

[w] mFOLFOX6 is administered every 14 days as a 46-hour continuous infusion, starting on Day 1 and completing on Day 3.

[x] AE collection begins following signing of the ICF for Screening. Events reported prior to the first on-study infusion will be considered pretreatment events and reported on the Medical History page of the eCRF, unless they directly correlate to a study-related procedure. Adverse event reporting will continue until completion of the EOT visit or until 28 days after the last dose of study treatment. All treatment-related SAEs will be monitored until the event has resolved or stabilized, any abnormal laboratory values have returned to baseline or stabilized at a level acceptable to the Investigator and Sponsor, there is a satisfactory explanation for the changes observed, or the patient is lost to follow-up. Serious AEs occurring after the EOT visit should be reported to the Sponsor if the Investigator considers there is a causal relationship with the study treatment.

Appendix 3: Schedule of Assessments –Randomized, Placebo-controlled Portion (Phase 3)

Procedure[a]	Pre-Screening	Screening	Study Treatment: Cycles 1 and 2			Study Treatment: Cycle 3, Cycle 4 and Subsequent Cycles				Follow-up		
		Day -21 to Day 0	Cycle 1 Day 1	Cycle 1 Day 8	Cycle 2 Day 1	Cycle 3[b] Day 1	Cycle 4[c] Day 1	As Clinically Indicated	Unique Schedules	EOT[d]	Scan Follow-up[bb]	Long-term Follow-up[e]
		Week 0	Week 1	Week 2	Week 3	≥Week 4						
Pre-screening informed consent[f]	X											
IHC analysis of FGFR2b expression	X											
Sample for blood-based biopsy (ctDNA) assay[g]	X											
Informed Consent[h]		X										
Review/Confirm Eligibility Criteria		X	X									
Medical/Oncology and Medication History		X	X									
Demography/Baseline Characteristics		X										
Physical Examination[i,j]		X	X	X	X	X	X			X	X	
ECOG Performance Status		X				X[k]			X[k]	X		
Patient Reported Outcomes (EQ-5D-5L and the EORTC QLQ-C30)[l]		X							X	X		
Vital Signs[m]		X	X	X	X	X	X	X		X	X	
12-lead ECG[n]		X						X		X		
Comprehensive Ophthalmologic Examination[o]		X				X		X		X		

Procedure[a]	Pre-Screening	Screening	Study Treatment: Cycles 1 and 2			Study Treatment: Cycle 3, Cycle 4 and Subsequent Cycles				Follow-up		
		Day -21 to Day 0	Cycle 1 Day 1	Cycle 1 Day 8	Cycle 2 Day 1	Cycle 3[b] Day 1	Cycle 4[c] Day 1	As Clinically Indicated	Unique Schedules	EOT[d]	Scan Follow-up[bb]	Long-term Follow-up[e]
		Week 0	Week 1	Week 2	Week 3	≥Week 4						
Slit Lamp Examination[p]									X			
Clinical Safety Laboratory Sampling[q]		X	X	X	X	X	X	X		X	X	
Pregnancy Test[r]		X	X[r]			X[r]			X	X[r]	X	X
Urinalysis[s]		X	X			X			X	X		
Radiological/Tumor Scans[t]		X							X	X[u]	X	
Randomization[cc]		X										
Survival Assessment												X
Immunogenicity Sampling[v]			X			X			X	X		
PK Samples[w]			X			X			X	X		
mFOLFOX6 Administration[x]			X		X	X	X					
IP Administration[y]			X		X	X	X					
Adverse Events[z]		X	X	X ———	———	———	———	———	———	——— X		
Concomitant Medications			X	X ———	———	———	———	———	———	——— X		X[aa]

[a] Unless specified, procedure is to be completed within ± 72 hours of scheduled time point and to be synchronized with the study treatment administration day.

[b] And subsequent odd cycles from Cycle 3 (except for pharmacokinetic, immunogenicity, and pharmacodynamic blood sample collections, which are described in Appendix 6).

[c] And subsequent even cycles from Cycle 4 (except for pharmacokinetic, immunogenicity, and pharmacodynamic blood sample collections, which are described in Appendix 6).

[d] End of Treatment (EOT) assessments should be performed 28 (±3) days following the last study treatment administration

[e] Patients will undergo long-term follow-up for survival by clinic visit or by telephone approximately every 3 months ± 28 days after the EOT visit until up to 24 months after the last patient is enrolled into the study, or until death, loss to follow-up, withdrawal of consent or study termination by the Sponsor (whichever occurs first) (see Section 7.3.5). For the first 6 months of the Long-term Follow-up Period any pregnancy that occurs should be reported to the Sponsor.

[f] Pre-screening ICF (see Section 1.8)

[g] Sample for blood-based biopsy (ctDNA) assay at Pre-screening for prospective analysis of *FGFR2* amplification (see Section 6.3).

[h] Written, signed informed consent must be collected prior to any study-specific procedures. The most recent IRB/EC approved ICF must be signed.

[i] Complete physical examination and height will be measured at Screening only. Limited physical examinations should be conducted, including examination of the oropharynx, thereafter.

[j] After Cycle 1, the IP dose will be recalculated at each infusion visit only if weight has changed >10% from Cycle 1, Day 1.

[k] ECOG Performance Status will be assessed at Cycle 3 Day 1 and Day 1 of every other subsequent cycle (odd cycles) until the EOT visit.

[l] The EQ-5D-5L and the EORTC QLQ-C30 will be administered at Screening (within 28 days of Cycle 1 Day 1) and then every 8 weeks until the EOT visit.

[m] Vital signs (blood pressure, heart rate, respiratory rate, and temperature) are to be measured at screening visit following 5 minutes of rest and on Cycle 1 Day 1 at the following time points: pre-dose, and 0.5, 1, 2, and 4 hours from the start the blinded IP infusion. On subsequent dosing days, pre-dose and at 0.5, 1, and 2 hours from the start of the blinded IP infusion.

[n] With patient resting for 5 minutes prior to recording.

[o] Comprehensive ophthalmologic examinations (conducted at Screening, prior to Cycle 3 Day 1, and at the EOT visit only) include fundoscopic and slit lamp exam, ocular coherence tomography (OCT), visual acuity, completion of fluorescein staining score form, determination of intraocular pressure, and review of ocular/visual symptoms. The comprehensive ophthalmologic examination will be repeated at any point if changes in visual acuity or visual symptoms are reported by patients.

[p] Slit lamp examinations without OCT for patients; every 8 weeks from Cycle 3 Day 1through study completion. If the patient comes off study but has any persistent corneal findings, the assessments should continue every 8 weeks until resolution of findings, withdrawal of consent, death, or loss to follow up. OCT is not required at these time points.

[q] Blood tests (evaluated by local laboratories) are listed in Appendix 4. Baseline hematology and blood chemistry test results must be obtained within 96 hours of study treatment administration on Cycle 1 Day 1 to confirm eligibility. On subsequent dosing days, hematology and blood chemistry results must be available within 72 hours prior to dosing. Coagulation samples need to be obtained at baseline, at Cycles 1 through 4, and at any time clinically indicated (e.g., patients on anticoagulant therapy requiring close monitoring).

[r] Serum ß-hCG (evaluated by local laboratories) will be performed on all women of childbearing potential within 96 hours prior to Cycle 1 Day 1 and at EOT. On dosing days of odd cycles (every other cycle), urine pregnancy results must be available within 72 hours prior to dosing. If serum B-hCG test is performed at screening and is not within 96 hrs of C1D1, the test must be repeated to confirm negative results.

[s] Includes dipstick for protein, glucose, blood, pH, and ketones on dosing days of odd cycles (every other cycle). If dipstick findings are abnormal, then a microscopic evaluation will be performed to assess the abnormal findings.

[t] Tumor scans will be performed at Screening (within 28 days of Cycle 1 Day 1) and then every 8 weeks (± 7 days) from Enrollment (Cycle 1 Day 1) thereafter.

[u] This scan can be omitted if the last scan was performed < 8 weeks prior to EOT visit or if tumor progression was previously determined.

[v] Blood samples for anti-FPA144 antibodies. Refer to Appendix 6 for collection times.

[w] Blood samples for PK analysis. Refer to Appendix 6 for collection times.

[x] mFOLFOX6 is administered every 14 days as a 46-hour continuous infusion, starting on Day 1 and completing on Day 3.

[y] FPA144 is administered every 14 days starting cycle 1, day 1. The first 3 doses (cycles) of FPA144 should be administered every 14 days (±3 days) regardless of delays in mFOLFOX6 treatment. After the first 3 doses, FPA144 may be delayed up to ±7 days to be synchronized with administered mFOLFOX6.

[z] AE collection begins following signing of the ICF for Screening. Events reported prior to the first on-study infusion will be considered pretreatment events and reported on the Medical History page of the eCRF, unless they directly correlate to a study-related procedure. Adverse event reporting will continue until completion of the EOT visit or until 28 days after the last dose of study treatment. All treatment-related SAEs experienced by a study patient will be monitored until the event has resolved or stabilized, any abnormal laboratory values have returned to baseline or stabilized at a level acceptable to the Investigator and Sponsor, there is a satisfactory explanation for the changes observed, or the patient is lost to follow-up. SAEs occurring after the EOT visit should be reported to the Sponsor by the Investigator if the Investigator considers there is a causal relationship with the study treatment.

[aa] For long-term follow-up, the only concomitant medication that needs to be collected is anti-cancer medication.

[bb] If a patient discontinues study treatment (IP and/or mFOLFOX6) for reasons other than disease progression or withdrawal of consent, they will have follow-up visits and continue to undergo tumor assessments according to the protocol schedule until radiographic progression or the initiation of additional anti-cancer therapy (at which point the patient would begin the long-term follow-up for survival).

[cc] Patients must receive first administration of study treatment within 3 days of randomization.

Appendix 4: Laboratory Evaluations

The following laboratory parameters will be determined in accordance with the Schedule of Assessments and can be performed locally:

**Hematology:**	
Complete blood cell (CBC) with differential:	
white blood cells (WBC)	platelets
ANC	hemoglobin
neutrophils (%)	hematocrit
eosinophils (%)	red blood cells (RBC)
basophils (%)	
lymphocytes (%)	
monocytes (%)	
**Urinalysis:**	
Dipstick (appearance, color, pH, specific gravity, ketones, protein, glucose, bilirubin, nitrite, urobilinogen, and occult blood)	
If dipstick is positive (2+ or greater) for blood or protein, perform a microscopic examination.	
**Clinical chemistry:**	
Albumin	globulin
alkaline phosphatase	glucose
ALT (SGPT)	lactate dehydrogenase (LDH)
AST (SGOT)	phosphate
blood urea nitrogen (BUN)	potassium
calcium	sodium
chloride	total bilirubin
carbon dioxide (CO_2 [bicarbonate])	total cholesterol
creatinine	total protein
direct bilirubin	uric acid
**Other chemistry tests:**	
Magnesium	
**Coagulation:**	
INR	APTT
**Serum pregnancy test:**	
In women of childbearing potential only.	

Appendix 5: Study Flowchart for Pharmacokinetic, Immunogenicity, and Pharmacodynamic Blood Sample Collections for Phase 1

Study Cycle	Study Day	Time Point	Type of Sample
Cycle 1	Day 1	≤ 4 hours prior to infusion	ctDNA blood assay sample
			FPA144 PK (serum)
			ADA (serum)
			Blood samples for exploratory biomarker analysis of FGFR pathway
		15 (±10) minutes after end of infusion	FPA144 PK (serum)
		4 hours (±60 minutes) after end of infusion	FPA144 PK (serum)
	Day 3	48 hours (±6 hours) after end of infusion	FPA144 PK (serum)
			Blood samples for exploratory biomarker analysis of FGFR pathway
	Day 8	168 hours (±1 day) after end of infusion	FPA144 PK (serum)
Cycle 2	Day 1	≤ 4 hours prior to infusion	FPA144 PK (serum)
			ADA (serum)
		15 (±10) minutes after end of infusion	FPA144 PK (serum)
	Day 3	48 hours (±6 hours) after end of infusion	FPA144 PK (serum)
Cycle 3, 5, 7, 9, 11	Day 1	≤ 4 hours prior to infusion	FPA144 PK (serum)
			ADA (serum) (Cycles 3 and 7)
			Blood samples for exploratory biomarker analysis of FGFR pathway
		15 (±10) minutes after end of infusion	FPA144 PK (serum)
Cycle 10	Day 1	15 (±10) minutes after end of infusion	FPA144 PK (serum)
	Day 3	48 hours (± 6 hours)	FPA144 PK (serum)
			Blood samples for exploratory biomarker analysis of FGFR pathway
Every 8 Cycles starting from Cycle 15	Day 1	≤ 4 hours prior to infusion	FPA144 PK (serum)
			ADA (serum)
			Blood samples for exploratory biomarker analysis of FGFR pathway
End of Treatment Follow-up	Visit date	During visit	FPA144 PK (serum)
			ADA (serum)
			Blood samples for exploratory biomarker analysis of FGFR pathway

Appendix 6: Study Flowchart for Pharmacokinetic and Immunogenicity Blood Sample Collections for Phase 3

Study Cycle	Study Day	Time Point	Type of Sample
Cycle 1	Day 1	≤ 4 hours prior to infusion	FPA144 PK (serum)
			ADA (serum)
		15 (±10) minutes after end of infusion	FPA144 PK (serum)
Cycle 3	Day 1	≤ 4 hours prior to infusion	FPA144 PK (serum)
			ADA (serum)
		15 (±10) minutes after end of infusion	FPA144 PK (serum)
Cycles 5, 9, and 17	Day 1	≤ 4 hours prior to infusion	FPA144 PK (serum)
			ADA (serum)
End of Treatment Follow-up	Visit date	During visit	FPA144 PK (serum)
			ADA (serum)

Appendix 7: ECOG Performance Status

Grade	Performance Status Criteria
0	Fully active, able to carry on all pre-disease activities without restriction.
1	Restricted in physically strenuous activity but ambulatory and able to carry out work of a light sedentary nature (light housework, office work).
2	Ambulatory and capable of all self-care but unable to carry out any work activities. Up and about more than 50% of waking hours.
3	Capable of only limited self-care, confined to bed or chair more than 50% of waking hours.
4	Completely disabled. Cannot carry on any self-care. Totally confined to bed or chair.

Appendix 8: Fluorescein Stain Grading Systems

<table>
<tr><td>DEWS</td><td>DRY EYE: DIAGNOSTIC TEST TEMPLATE</td><td></td></tr>
<tr><td>RAPPORTEUR</td><td>A.J.Bron</td><td>21st Oct 2004</td></tr>
<tr><td>TEST</td><td>Grading staining: Oxford Schema</td><td></td></tr>
<tr><td>TO DIAGNOSE</td><td>The scheme is used to estimate surface damage in dry eye.</td><td>REFERENCES</td></tr>
<tr><td>VERSION of TEST</td><td>[V 1]</td><td></td></tr>
<tr><td>DESCRIPTION</td><td>Surface damage to the exposed eye, assessed by staining, is graded against standard charts.</td><td></td></tr>
<tr><td>NATURE of STUDY</td><td>N. A.</td><td></td></tr>
<tr><td>CONDUCT of TESTS</td><td>Grading Schema:
Staining is represented by punctate dots on a series of panels (A-E). Staining ranges from 0-5 for each panel and 0-15 for the total exposed inter-palpebral conjunctiva and cornea. The dots are ordered on a log scale
<table>
<tr><th>PANEL</th><th>GRADE</th><th>CRITERIA</th></tr>
<tr><td>A 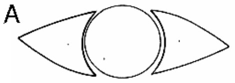</td><td>0</td><td>Equal to or less than panel A</td></tr>
<tr><td>B 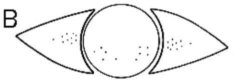</td><td>I</td><td>Equal to or less than panel B, greater than A</td></tr>
<tr><td>C 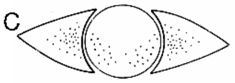</td><td>II</td><td>Equal to or less than panel C, greater than B</td></tr>
<tr><td>D 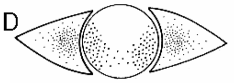</td><td>III</td><td>Equal to or less than panel D, greater than C</td></tr>
<tr><td>E 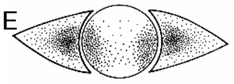</td><td>IV</td><td>Equal to or less than panel E, greater than D</td></tr>
<tr><td>>E</td><td>V</td><td>Greater than panel E</td></tr>
</table>
Conduct of Test:

Dye is instilled.
Slit-lamp is set (eg.16 magnification with x10 oculars with Haag-Streit).
Cornea: The upper eyelid is lifted slightly to grade the whole corneal surface,
Conjunctiva: To grade the temporal zone, the subject looks nasally; to grade the nasal zone the subject looks temporally.
(The upper and lower conjunctiva can also be
</td><td>Bron Evans Smith 2003.</td></tr>
</table>

Fluorescein Stain Grading Systems (Cont.)

	graded).<br>**Selection of dyes:**<br>A list dyes and filters can be found in the original paper.<br>With fluorescein, staining must be graded as quickly as possible after instillation, since the dye then diffuses rapidly into the tissue and its high luminosity blurring the stain margin.<br>Staining after rose bengal or lissamine green, persists at high contrast and may therefore be observed for a considerable period. This is convenient for both grading and photography.<br><br>**Fluorescein sodium**<br>**1. Quantified drop instillation**<br>eg 2 μl of 2 % sterile fluorescein instilled into each conjunctival sac with a micro-pipette (using a sterile tip). In very dry eye, larger volumes risk the possibility of inadequate dilution into the fluorescent range.<br>**2. Unquantified instillation – impregnated paper strips**<br>This is a convenient approach in the clinic using the following method of application:<br>• A single drop of unit dose saline is instilled onto a fluorescein-impregnated strip.<br>• When the drop has saturated the impregnated tip, the excess is shaken into a waste bin with a sharp flick.<br>• The right lower lid is then pulled down and the strip is tapped onto the lower tarsal conjunctiva. A similar procedure is carried out on the left.<br>If too large a volume is delivered then the concentration in the tear film will be too high, and the tear film and staining pattern will be non-fluorescent.<br>**3.Timing**<br>The fluorescein break-up time (FBUT) is usually performed prior to grading. Since fluorescein diffuses rapidly into tissues, punctate staining blurs after a short period. It is therefore essential to assess staining rapidly, in sequence, in the right and then the left eye, so that the staining patterns observed are equally crisp.<br>If it is intended to photograph the staining pattern for grading, then photography should follow immediately after each instillation.<br><br>**Exciter and Barrier Filters**<br>The absorption peak of fluorescein sodium occurs between 465 - 490 nm and the emission peak between 520 - 530 nm A suggested filter pair for detection of fluorescein staining is a yellow, Kodak Wratten 12 barrier filter (transmitting above 495 nm) or an orange Wratten 15 filter (transmitting above 510 nm) in combination with a blue Wratten 47 or 47A exciter filter. The 47A shows greater transmittance than the Wratten 47 over the absorption range. The 'cobalt' filter of many slit-lamps is suitable to use with a Wratten 12 or 15	

Fluorescein Stain Grading Systems (Cont.)

	barrier.<br><br>Where more light is required for photographic purposes, narrow band-pass, interference filters can be used.<br><br>The use of both exciter and barrier filters allows both the cornea and conjunctiva to be assessed using a single stain. This is a major advantage in clinical trials where it is otherwise customary to employ fluorescein to grade corneal staining and rose bengal or lissamine green to grade conjunctival staining.<br><br>**Disadvantages of Fluorescein Staining**<br>Blurred pattern if reading is delayed. Delay in photographing fluorescein staining results in blurred images of the staining pattern.<br><br>**Rose Bengal**<br>The intensity of rose bengal staining is dose dependent. If drop size or concentration is reduced to minimize stinging, the amount of staining is also reduced. Use of impregnated strips will give weaker staining than use of a full drop of 1% solution. Best results are achieved with, eg. 25 µl 1%, instilled into the conjunctival sac. Because rose bengal stings, instillation is best preceded by a topical anesthetic.<br><br>**Instillation Technique**<br>1) eg. A drop of Proxymetacaine is instilled into the conjunctival sac followed, after recovery, by;<br>2) A drop of rose bengal 1.0%. This is instilled onto the upper bulbar conjunctiva with the upper lid retracted and the patient looking down.<br>3) Since both anaesthetic and drop may stimulate reflex tearing, the test should follow measurement of the FBUT and of the Schirmer test. (Conjunctival staining due to insertion of the Schirmer paper can usually be distinguished from that due to dry eye disease).<br><br>Both eyes may be stained prior to grading, since there is no risk of the staining pattern in the first eye being obscured by the time the second eye is graded.<br><br>The cited paper gives advice about avoidance of overspill.<br><br>**Visibility**<br>Rose bengal staining on the conjunctiva shows up well against the sclera and may be enhanced using a red-free (green) light source. Corneal staining may show up well against a blue iris, but is difficult to see against a dark brown iris.<br><br>**Phototoxicity**<br>Photo-activation of rose bengal by sunlight increases post-instillation symptoms, especially in severe dry eye with heavy staining. This post-instillation pain can be minimised	

Fluorescein Stain Grading Systems (Cont.)

<table>
<tr><td></td><td>by liberal irrigation with normal saline at the end of the test.

Lissamine green stains the eye in a similar manner to rose bengal but is as well tolerated as fluorescein. Visibility and dose-dependency are the same as rose bengal and staining is persistant so that photography need not be performed immediately after instillation.
Lissamine green is available as impregnated strips or may be ordered as a pre-prepared solution. A 25 μl 1% drop will give more intense staining. Because the drop is well tolerated, no anaesthetic is required.

Visibility
As with rose bengal, lissamine green staining is easily visible on the conjunctiva. On the cornea, staining is seen well against a light blue iris background but is poorly visible against a dark brown iris background. For both rose bengal and lissamine green, because the dyes are poorly seen within the tear film, the dye in the tear film does not obscure the staining pattern. Also, since both dyes do not diffuse into the substantia propria of the conjunctiva, the staining pattern is retained for longer.

Visibility of staining may be enhanced using a white light source and a red barrier filter, to give a black pattern on a red ground. A suitable filter is a Hoya 25A, or a Kodak Wratten 92.</td><td></td></tr>
<tr><td>Web Video</td><td>Not available</td><td></td></tr>
<tr><td>Materials:</td><td>Oxford Grading Charts [redacted]</td><td></td></tr>
<tr><td>Standardization</td><td>Nil additional</td><td></td></tr>
<tr><td>Variations of technique</td><td></td><td></td></tr>
<tr><td>Diagnostic value</td><td>No stats supplied.</td><td></td></tr>
<tr><td>Repeatability</td><td>A small intra-interobserver study was carried out in 1986 and was presented but not published:

Intra-observer study: This study asked two trained ophthalmologists to grade a series of standard slides, showing corneal and conjunctival fluorescein staining, on 2 separate occasions. [note: -this study is only relevant to grading photographic records not patients.]

<table><tr><td colspan="3">Intra-observer κ for grading photographs of staining, using the Oxford scheme. Two observers.</td></tr><tr><td></td><td>Cornea</td><td>Conjunctiva</td></tr><tr><td>Observer 1</td><td>0.86</td><td>0.69</td></tr><tr><td>Observer 2</td><td>0.65</td><td>0.83</td></tr></table>
Not that values are in the good to excellent range.

Inter-observer study: In this study, the same 2 observers</td><td>Hardman Lea et al. 1986 AER abstract.</td></tr>
</table>

Fluorescein Stain Grading Systems (Cont.)

	graded fluorescein staining (blue exciter; yellow filter) in 13 dry eye patients at an interval within 2-3 weeks.<br><br>Inter-observer κ for grading patients with dry eye, using the Oxford scheme. Two observers. Fluorescein; bengal rose<br>Observer 1 v 2 / Cornea / Conjunctiva<br>Fluorescein / 0.88 / 0.48<br>Bengal rose / 0.87 / 0.54<br><br>It is of interest that observations are in the excellent category for cornea, with either stain and in the fair category for conjunctiva.	
**Sensitivity**	**(true positives)** [-]	
**Specificity**	**(100 – false positives)** [-]	

References:

Bron A, Evans VE, Smith JA. (2003). Grading of corneal and conjunctival staining in the context of other dry eye tests. *Cornea* 22(7): 640-50.

(Bron 2003)

FIGHT: A Phase 2 Randomized, Double-Blind, Controlled Study Evaluating FPA144 and Modified FOLFOX6 in Patients with Previously Untreated Advanced Gastric and Gastroesophageal Cancer: Phase 2 Preceded by Dose-Finding in Phase 1

Protocol Number:	FPA144-004
Investigational Product:	FPA144
IND Number:	117701
Development Phase:	Phase 1/2
Indication Studied:	Advanced Gastric and Gastroesophageal Cancer
Protocol Version:	Amendment 4
Date of Protocol:	10 March 2021
Supersedes	*Version 3, 05 June 2020*
Sponsor:	Five Prime Therapeutics, Inc.<br>[redacted]
Responsible Medical Officer:	[redacted]

Confidential

This document contains proprietary and confidential information of Five Prime Therapeutics, Inc. and may not be reproduced or disclosed in whole or in part without written authorization from Five Prime Therapeutics, Inc.

DocuSign Envelope ID: 20B82B7A-5340-49B0-8A1F-588EE5F9FDFE

Protocol Approval Signature Page

Declaration of Sponsor

FIGHT: A Phase 2 Randomized, Double-Blind, Controlled Study Evaluating FPA144 and Modified FOLFOX6 in Patients with Previously Untreated Advanced Gastric and Gastroesophageal Cancer: Phase 2 Preceded by Dose-Finding in Phase 1

The study will be conducted according to the protocol and in compliance with Good Clinical Practice (GCP), the Declaration of Helsinki, and other applicable regulatory requirements. Essential study documents will be archived in accordance with applicable regulations.

This study protocol was subjected to critical review. The information it contains is consistent with current knowledge of the risks and benefits of the investigational product, as well as with the moral, ethical, and scientific principles governing clinical research as set out in the Declaration of Helsinki, 1989, and the International Conference on Harmonization (ICH) guidelines on GCP.

[redacted]

10Mar2021

Date

Five Prime Therapeutics, Inc.

Declaration of the Investigator

FIGHT: A Phase 2 Randomized, Double-Blind, Controlled Study Evaluating FPA144 and Modified FOLFOX6 in Patients with Previously Untreated Advanced Gastric and Gastroesophageal Cancer: Phase 2 Preceded by Dose-Finding in Phase 1

All documentation for this study that is supplied to me and that has not been previously published will be kept in the strictest confidence. This documentation includes this study protocol, Investigator's Brochure (IB), electronic case report forms (eCRFs), and other scientific data.

The study will not be commenced without the prior written approval of a properly constituted Institutional Review Board (IRB) or Independent Ethics Committee (IEC). No changes will be made to the study protocol without the prior written approval of the Sponsor and the IRB or IEC, except as necessary to eliminate an immediate hazard to the patients.

I confirm that I have read the above-mentioned protocol/protocol amendments and its attachments. I agree to conduct the described trial in compliance with all stipulations of the protocol, applicable laws, regulations and ICH E6 Guideline for Good Clinical Practice (GCP).

____ ____________________

Principal Investigator's Signature Date

____

Name (printed)

____

Institution or Company Name

Protocol Synopsis

Title: **FIGHT: A Phase 2 Randomized, Double-Blind, Controlled Study Evaluating FPA144 and Modified FOLFOX6 in Patients with Previously Untreated Advanced Gastric and Gastroesophageal Cancer: Phase 2 Preceded by Dose-Finding in Phase 1**

Protocol Number: FPA144-004

Clinical Phase: 1/2

Sponsor: Five Prime Therapeutics, Inc.

Study Centers: Up to approximately 190 global study centers

Phase 1 Objectives:

Primary

To determine the recommended dose (RD) of FPA144 combined with a fixed dose of 5-fluorouracil (5-FU), leucovorin, and oxaliplatin (mFOLFOX6) (hereinafter referred to as FPA144 + mFOLFOX6) in patients with advanced gastrointestinal (GI) tumors

Secondary

- To evaluate the safety and tolerability of FPA144 + mFOLFOX6 in patients with GI tumors
- To characterize the pharmacokinetic (PK) profile of FPA144 + mFOLFOX6 in patients with GI tumors
- To characterize the immunogenicity of FPA144

Exploratory

To characterize the pharmacodynamic profile of FPA144 + mFOLFOX6 in patients with GI tumors

Phase 2 Objectives:

Primary

To compare investigator-assessed progression-free survival (PFS) in patients with fibroblast growth factor receptor 2 (FGFR2)-selected gastric or gastroesophageal cancer (GC) treated with FPA144 + mFOLFOX6, to those treated with placebo combined with mFOLFOX6 (hereafter referred to as placebo + mFOLFOX6)

Secondary

To compare the following in patients with FGFR2-selected GC treated with FPA144 + mFOLFOX6 to those treated with placebo + mFOLFOX6:

- Overall survival (OS)
- Investigator-assessed objective response rate (ORR)
- Safety and tolerability

Exploratory

To compare the following in patients with FGFR2-selected GC treated with FPA144 + mFOLFOX6 to those treated with placebo + mFOLFOX6:

- Duration of response (DOR)
- Patient-reported outcomes (PROs) and quality of life (QOL) outcomes until investigator-assessed disease progression
- To explore the association between FGFR2 status (in tumor tissue and/or blood) with clinical outcome
- To explore the concordance between FGFR2b overexpression in tumor tissue and *FGFR2* gene amplification in blood

To characterize the following:

- PK profile of FPA144 + mFOLFOX6 in patients with FGFR2-selected GC
- Immunogenicity of FPA144

Phase 1 Endpoints:

Primary

The incidence of Grade 2 or higher adverse events (AEs) assessed as related to FPA144 by the investigator and the incidence of clinical laboratory abnormalities defined as dose-limiting toxicities (DLTs)

Secondary

- The incidence of AEs, clinical laboratory abnormalities, corneal and retinal findings, and electrocardiogram (ECG) abnormalities
- PK parameters of FPA144, such as area under serum concentration-time curve (AUC), maximum serum concentration (C_{max}), trough serum concentration (C_{trough}), clearance (CL), terminal half-life ($t_{1/2}$), volume of distribution, the time to achieve steady state, dose-linearity, and accumulation ratio
- Incidence of treatment-emergent anti-FPA144 antibody response

Exploratory

Pharmacodynamic parameters, including exploratory pharmacodynamic biomarker analyses of the FGFR pathway in blood

Phase 2 Endpoints:

Primary

Investigator-assessed progression-free survival (PFS), defined as time from randomization until the date of disease progression based on investigator assessment (using Response Evaluation Criteria in Solid Tumors version 1.1 [RECIST v1.1]) or death from any cause, whichever comes first

Secondary

- Overall survival (OS), defined as time from randomization until death from any cause
- Objective response rate (ORR), defined as the proportion of patients with partial or complete response based on investigator assessment of tumor lesions per RECIST v1.1
- Incidence of AEs, clinical laboratory abnormalities, corneal and retinal findings, and ECG abnormalities

Exploratory

- DOR limited to patients who are responders to treatment, as determined by the investigator per RECIST v1.1, and defined as the time of first response to progression or death from any cause, whichever comes first
- Change from baseline in functional outcomes as measured by EuroQOL-5D-5L (EQ-5D-5L) and the European Organization for Research and Treatment of Cancer Quality of Life Questionnaire Version 3.0 (EORTC QLQ-C30)
- The correlation between identified FGFR2 status in tumor tissue and/or circulating tumor DNA (ctDNA) blood assay, as determined by immunohistochemistry (IHC) and blood-based molecular diagnostic assay, with OS, PFS, and ORR per RECIST v1.1
- The correlation between identified FGFR2b overexpression in tumor tissue by IHC and *FGFR2* gene amplification as determined by ctDNA blood assay
- Pharmacokinetic (PK) parameters, C_{max} and C_{trough} of FPA144 in combination with mFOLFOX6
- Incidence of treatment-emergent anti-FPA144 antibody response

Investigational Product

FPA144 drug product is supplied for intravenous (IV) administration as a sterile, aqueous, colorless to slightly yellowish, pyrogen-free solution supplied in single-use glass vials. The composition of the drug product contains 20 mg/mL active ingredient, 20 mM L-histidine, 270 mM sucrose, and 0.01% (w/v) polysorbate 20 at pH 6.0.

mFOLFOX6: Leucovorin (or levo-leucovorin), 5-FU, and oxaliplatin will be obtained from Sponsor-approved commercial sources at each participating site. Management (ie, handling, storage, administration, and disposal) of these products will be in accordance with the relevant local guidelines. For countries where the Sponsor is required to provide all study drugs, including standard-of-care drugs, the Sponsor will provide leucovorin, 5-FU, and oxaliplatin from a commercial supply that is clinically labeled in accordance with relevant local guidelines. For further

	details, refer to the manufacturer's prescribing information for the respective chemotherapies and the study Pharmacy Manual.<br>**Placebo** (only used in Phase 2) will match the FPA144 drug product. It will be supplied for IV administration as a sterile, aqueous, colorless to slightly yellowish, pyrogen-free solution supplied in single-use vials. The composition of matching placebo contains 20 mM L-histidine, 270 mM sucrose, and 0.01% (w/v) polysorbate 20 at pH 6.0.<br>Administration of Study Treatment is outlined below.
Study Design	This is a double-blind, randomized, controlled, multicenter Phase 1/2 study to evaluate the safety, tolerability, efficacy, and PK of FPA144 + mFOLFOX6 versus placebo + mFOLFOX6.<br>This study includes a Phase 1 safety run-in portion and a Phase 2 portion. Patients may enroll in either Phase 1 or Phase 2, but may not enroll in both phases of the study.<br>The Phase 1 safety run-in is an open-label dose-escalation of FPA144 + mFOLFOX6 in patients with GI tumors (not FGFR2-selected).<br>The Phase 2 part of this study was initially designed as a Phase 3 study in the front-line setting evaluating bemarituzumab (FPA144) with SOC chemotherapy. The study initiated following promising monotherapy activity of bemarituzumab in the Phase 1 study conducted in late-line gastric cancer. The Phase 3 statistical design assumed the incidence of FGFR2b positivity in front-line gastric cancer to be ~10%. However, over the conduct of the study, the true incidence has been 30%. Additionally 2 methods of testing for FGFR2b status, IHC and ctDNA, were utilized to identify eligible patients and the expectation was that the majority of positive tumors would be identified by both tests. In actuality, the vast majority of tumors have been identified by IHC alone, leading to a question of adequate study power. The study design has been modified in consideration of these factors.<br>The Phase 2 portion of the study (to follow the Phase 1 safety run-in) is a global, randomized, double-blind, controlled, study to evaluate the efficacy of FPA144 + mFOLFOX6 versus placebo + mFOLFOX6 in patients with FGFR2-selected GC (as determined by prospective IHC demonstrating FGFR2b overexpression and/or a ctDNA blood assay demonstrating *FGFR2* gene amplification).<br>**<u>FGFR2 Expression Requirements (Pre-Screening):</u>**<br>• Required provision of tissue and blood sample for FGFR2 positive status by 1 of these testing methods for enrollment in Phase 2: Provision of archival (or fresh biopsy if archival tissue is not available) for pre-screening FGFR2b overexpression testing by IHC is required; provision of a blood sample is also required for pre-screening by ctDNA for FGFR2 amplification.

- Results of the centralized FGFR2 testing may take approximately 2 weeks; patients are permitted to receive up to 1 dose of mFOLFOX6 during this interim time period (pre-screening) at the discretion of the investigator. This 1 dose of chemotherapy is not a requirement of the study and is not considered part of this clinical study.

Screening Period:

Pre-screening allows for collection of blood and tissue to determine FGFR2 positivity using ctDNA and IHC assays. Once a patient is confirmed to have a positive result based on central testing, the screening period initiates, allowing for evaluation of all other eligibility criteria.

All study assessments are outlined in the Schedules of Assessments in Protocol Appendix 2 and Appendix 3.

Phase 1 Safety Run-in

Dose Escalation Design

The Phase 1; after a maximum 28-day screening period, eligible patients will initiate study treatment.

In Phase 1, dose cohorts are planned at proposed doses beginning at an FPA144 dose level of 6 mg/kg per dose, and enrollment will depend on safety and tolerability. Phase 1 will include a standard 3+3 dose escalation design (Cohort 1), and rolling-6 design (Cohort 2, 1a, and 1b), until the RD of FPA144 to be administered in combination with mFOLFOX6 in Phase 2 is determined. A minimum of 2 dosing cohorts of FPA144 combined with mFOLFOX6 will be included in Phase 1.

Proposed FPA144 Dose Levels

The proposed FPA144 dose levels for dose escalation are as follows:

- Cohort 1: 6 mg/kg FPA144 once every 2 weeks (Q2W)
- Cohort 2: 15 mg/kg FPA144 Q2W with 1 dose of FPA144 7.5 mg/kg on Day 8 (Cycle 1 only)
- Cohort 1a (if needed, eg, if the Cohort 2 dose level or schedule is not tolerated): 15 mg/kg FPA144 Q2W
- Cohort 1b (if needed, eg, if Cohort 2 and 1a dose levels or schedules are not tolerated): Dose level lower than Cohort 1a, but higher than Cohort 1 to achieve tolerability with optimal target exposure.

Dose-Limiting Toxicity Observation

Patients will be observed for a 28-day DLT period, starting on the first day (Cycle 1 Day 1 [C1D1]) of treatment with FPA144 and mFOLFOX6, for safety assessments, PK, and occurrence of DLTs. If the first dose cohort (6 mg/kg) clears the 28-day DLT period, the second dose cohort (15 mg/kg Q2W with 1 dose of 7.5 mg/kg on Day 8 [Cycle 1 only]) will be tested in a rolling-6 design and will enroll 6 patients to explore the safety and efficacy at this dose level. Subsequent cohorts will also be

enrolled as a rolling 6 design and will only be enrolled if Cohort 2 is deemed intolerable.

Dose-Limiting Toxicity Definitions

DLTs during Phase 1 are defined as any of the following events considered by the investigator to be related to study drug:

- Absolute neutrophil count (ANC) < 0.5×10^9/L > 5 days' duration or febrile neutropenia (ie, ANC < 1.0×10^9/L with a single temperature of > 38.3°C, or fever ≥ 38°C for more than 1 hour). Use of granulocyte-colony stimulating factor (G-CSF) is permitted in accordance with institutional standards
- Platelets < 25×10^9/L
- Platelets < 50×10^9/L with bleeding requiring medical intervention
- Platelets < 50×10^9/L (> 3 days)
- Grade 4 anemia (ie, life-threatening consequences; urgent intervention indicated)
- Any Grade 2-3 ophthalmologic AE that does not resolve within 7 days
- Any Grade 4 ophthalmologic AE
- Any Grade 4 laboratory value
- Any Grade 3 laboratory values that are not of clinical significance according to investigator and Sponsor agreement if they do not resolve within 72 hours
- Aspartate aminotransferase/alanine aminotransferase (AST/ALT) ≥ 3× upper limit of normal (ULN) and concurrent total bilirubin ≥ 2× ULN not related to liver involvement with cancer
- Any non-hematological AE Grade 3 or greater (except nausea, vomiting, and diarrhea)
- Grade 3 nausea, vomiting or diarrhea that does not resolve with supportive care in 72 hours
- Grade 4 nausea, vomiting or diarrhea

Algorithm for Dose Escalation Decisions

The following algorithm will be used for dose escalation decisions in Phase 1:

Number of Patients with DLTs	Dose Escalation Decision
0/3	Open next cohort
1/3	Enroll 3 more patients in same cohort
≥ 2/3	Stop enrollment. If Cohort 1, then the study will be stopped.
1/6	Open next cohort
≥ 2/6	Stop enrollment at that level. If at Cohort 1, the study will end. If at Cohort 2 or Cohort 1a, then Cohorts 1a or Cohort 1b will open respectively and 6 patients will be enrolled.

After the 28-day DLT period, mFOLFOX6 or FPA144 may be dose adjusted (held or reduced) based on the toxicity experienced according to Section 5.1.3.4 and Section 5.2.3.1.

Determination of a Recommended Dose for Phase 2

The RD of FPA144 for Phase 2 will be identified by the Cohort Review Committee (CRC) based on an evaluation of all available safety, tolerability, and PK data and will not exceed 15 mg/kg administered IV Q2W with 1 dose of 7.5 mg/kg on Day 8 of Cycle 1 only. Based on the totality of the data, the chosen RD of FPA144 will be a dose that is not anticipated to lead to a decrease in the dose intensity of mFOLFOX6 to be administered. Cohort 1a will not be opened for enrollment unless the Cohort 2 dose level is not tolerated. Identifying a maximum tolerated dose (MTD), therefore is not a requirement of this study and the recommended dose (RD) may be lower than the MTD.

The MTD is defined as the maximum dose at which < 33% of patients experience a DLT during the DLT period. If a DLT is observed in 1 of 3 patients in Cohort 1, then 3 additional patients will be enrolled at that dose level. Dose escalation may continue until 2 of 3 to 6 patients treated at a dose level experience a DLT. The next lower dose will then be considered the MTD.

No additional doses of FPA144 or more than 2 doses of mFOLFOX6 should be administered during the 28-day DLT period. The doses of FPA144 and mFOLFOX6 on Day 1 of Cycle 2 do not need to be synchronized. For example, if mFOLFOX6 is delayed due to an AE that is deemed related only to mFOLFOX6 and not to FPA144, FPA144 should be administered as scheduled for Cycles 1 and 2 regardless of delays in the mFOLFOX6 dosing schedule.

Phase 2

Phase 2 Dose

Based on an assessment of overall safety, tolerability, and PK of FPA144 in combination with mFOLFOX6 by the CRC, the dose of 15 mg/kg Q2W with an additional dose of 7.5 mg/kg on Cycle 1 Day 8 will be used for the Phase 2 portion of the trial.

Global, Randomized, Double-Blind, Controlled Design

Enrollment in Phase 2 will begin when an RD for FPA144 has been identified by the CRC, which will not exceed the highest dose level evaluated and tolerated in Phase 1. Opening the Phase 2 portion of the study for enrollment will be at the discretion of the Sponsor.

The Phase 2 screening period will be up to 28 days; after a maximum 28-day screening period, eligible patients will initiate randomized study treatment.

Phase 2 will include 2 treatment arms:

- Arm 1: FPA144 combined with mFOLFOX6, administered Q2W
- Arm 2: Placebo combined with mFOLFOX6, administered Q2W

In Phase 2, patients must initiate first administration of study treatment within 3 days of enrollment.

Dosing

Administration of Study Treatment

Phase 1 (Open Label, FPA144 + mFOLFOX6)

FPA144 administration is outlined in Section 5.1.2 of the protocol; guidance for dose modification is provided in Section 5.1.3.

FPA144 will be administered over approximately 30 minutes (± 10 minutes) Q2W (± 3 days) on Day 1 of each 2-week cycle. FPA144 will be administered prior to mFOLFOX6 chemotherapy. Patients treated in Cohort 2 (only) will receive 1 additional dose of FPA144 on Day 8 of Cycle 1 (mFOLFOX6 will not be administered on this day).

mFOLFOX6 administration is outlined in Section 5.2 of the protocol; guidance for dose modification is provided in Section 5.2.3.1.

mFOLFOX6 will be administered Q2W beginning on C1D1 and will be administered at least 30 minutes after the end of the infusion of FPA144.

Leucovorin (or levo-leucovorin), 5-FU, and oxaliplatin will be administered in accordance with the relevant local guidelines and summary of product characteristics (SmPCs). For further details, refer to the manufacturer’s prescribing information for the respective chemotherapies and the study Pharmacy Manual.

Instructions for mFOLFOX6 administration include the following:

On Day 1 of each cycle (at least 30 minutes after FPA144/placebo):

- Oxaliplatin 85 mg/m^2 IV infusion over 120 minutes

- Leucovorin 400 mg/m^2 IV infusion over 120 minutes, can be administered concurrently with oxaliplatin if using a Y connector, (if not using a Y connector, administer drugs sequentially)
  - If leucovorin is unavailable, 200 mg/m^2 levo-leucovorin may be used. Study treatment may be administered without either agent in the event that both are unavailable. Leucovorin (or levo-leucovorin) may be given at either the protocol-recommended doses or as deemed appropriate by the investigator in accordance with institutional standard of care
- Immediately after oxaliplatin and leucovorin, 5 FU 400 mg/m^2 bolus over approximately 5 minutes
- Immediately after the 5-FU bolus, 5-FU 2400 mg/m^2 as a continuous IV infusion over approximately 48 hours

Premedication for mFOLFOX6 may be used at the discretion of the investigator based on the local standard of care.

Phase 2 (Double-Blind, Randomized, Controlled)

Patients in Phase 2 will be randomized to receive FPA144 + mFOLFOX6 (Arm 1) or placebo + mFOLFOX6 (Arm 2). FPA144 will be prepared and administered as in Phase 1, and the dosing and schedule of FPA144 will be 15 mg/kg Q2W with an additional dose of 7.5 mg/kg on Cycle 1 Day 8 (determined in Phase 1).

mFOLFOX6 will be administered as described for Phase 1: Q2W beginning at least 30 minutes after the end of the infusion of FPA144/placebo. For further details, refer to the manufacturer's prescribing information for the respective chemotherapies and to the study Pharmacy Manual.

Dose Modification, Duration, and Discontinuation

Dose Delays and Modifications

In the event a cycle of mFOLFOX6 is delayed due to chemotherapy-related toxicity during the first 3 cycles of treatment (42 days), FPA144/placebo should be administered on schedule (± 3 days). After the first 3 cycles, FPA144/placebo may be delayed up to 7 days to be synchronized with administered mFOLFOX6.

Duration of Dosing

There is no protocol-mandated maximum number of doses for FPA144/placebo or mFOLFOX6.

In Phase 1, upon completion of the DLT period (starting with Cycle 3), patients may continue receiving FPA144 in combination with mFOLFOX6. Ongoing administration of the mFOLFOX6 regimen beyond the DLT period will be according to the schedule noted in Section 5.1.2 with any modifications as noted in Section 5.2.3.1.

	In both study phases, FPA144 or placebo dosing will continue Q2W until investigator-assessed disease progression, unacceptable toxicity, or until the patient meets any of the other protocol-specified withdrawal criteria (refer to Section 4.5).<br>***Discontinuation***<br>Patients who discontinue all study treatment (all components of FPA144 + mFOLFOX6 and placebo + mFOLFOX6) for any reason other than consent withdrawal will undergo an EOT safety follow-up visit approximately 28 days after the last dose of the last administered component of treatment (oxaliplatin, leucovorin, 5-FU, or FPA144). In addition, patients in Phase 2 will continue to undergo tumor assessments according to the protocol schedule until investigator-assessed disease progression or the initiation of additional anticancer therapy, at which point they will enter a long term follow up (LTFU) period to assess survival.<br>Discontinuation of any component of the study treatment (mFOLFOX6, a component of mFOLFOX6, or FPA144/placebo) for any reason other than disease progression or any of the other protocol-specified withdrawal criteria does not mandate discontinuation of other components. The exception is the permanent discontinuation of 5-FU for any reason, which requires discontinuation of oxaliplatin and leucovorin.
**Study Duration**	The duration of study for an individual patient includes Screening (up to 28 days), treatment and an EOT visit approximately 28 days after the last dose. Since all patients are eligible to be treated until disease progression, the actual treatment duration for each individual patient will vary depending on the time to progression.<br>In addition, patients enrolled in Phase 2 will be followed for subsequent anticancer therapy and survival. LTFU for survival will be performed approximately every 3 months (± 1 month) after the EOT visit until up to 24 months after the last patient is enrolled in the study, or until death, loss to follow up, withdrawal of consent, or study termination by the Sponsor (whichever occurs first).
**Number of Patients**	The total number of patients planned for enrollment in this study is approximately 167.<br>• **Phase 1** will enroll approximately 9 to 21 patients depending on incidence of DLTs; this allows for evaluation of safety, PK, and pharmacodynamics at 1 or more dose levels.<br>• **Phase 2** will enroll approximately 155 patients with FGFR2b overexpression and/or FGFR2 gene amplification. Patients will be randomized 1:1 to receive FPA144 at the RD in combination with mFOLFOX6 versus placebo in combination with mFOLFOX6

Patient Replacements

Phase 1

To be evaluable in Phase 1, patients must have received 2 doses of FPA144 (except for Cohort 2 [must have received 3 doses of FPA144]) and 2 doses of mFOLFOX6 (regardless of cohort). All patients deemed unevaluable must be replaced, unless the reason they didn't receive the required minimum amount of therapy was due to an FPA144-related DLT. The replaced patient may continue on study at the investigator's discretion and after discussion with the Sponsor.

Phase 2

Patients are not replaced. All enrolled patients are deemed evaluable for the endpoint of survival.

Eligibility Criteria

Inclusion Criteria for Phase 1 and Phase 2 (criteria may not be sequentially numbered due to data management convention)

Patients enrolling in either Phase 1 or Phase 2 of the study must meet ***all*** of the following inclusion criteria:

1) Disease that is unresectable, locally advanced, or metastatic (not amenable to curative therapy)
2) Understand and sign an Institutional Review Board (IRB)/Independent Ethics Committee (IEC)-approved ICF prior to any study-specific evaluation
3) Life expectancy of at least 3 months in the opinion of the investigator
4) Eastern Cooperative Oncology Group (ECOG) performance status of 0 to 1
5) Age $\geq$ 18 years at the time the ICF is signed
6) In sexually active patients (women of child bearing potential [WOCBP] and males), willingness to use 2 effective methods of contraception, of which 1 must be a physical barrier method (condom, diaphragm, or cervical/vault cap) until 6 months after the last dose of FPA144. Other effective forms of contraception include:
    - Permanent sterilization (hysterectomy and/or bilateral oophorectomy, or bilateral tubal ligation with surgery, or vasectomy) at least 6 months prior to screening
    - WOCBP who are on stable oral contraceptive therapy or intrauterine or implant device for at least 90 days prior to the study, or abstain from sexual intercourse as a way of living
7) Adequate hematological and biological function, confirmed by the following laboratory values within 96 hours prior to enrollment:

Bone Marrow Function

- ANC ≥ 1.5 × 10^9/L
- Platelets ≥ 100 × 10^9/L
- Hemoglobin ≥ 9 g/dL

Hepatic Function

- AST and ALT < 3 × ULN; if liver metastases, then < 5 × ULN
- Bilirubin < 1.5 × ULN except in patients with Gilbert's disease

Renal Function

- Calculated creatinine clearance (CrCl) using Cockcroft Gault formula ≥ 50 mL/min *or* estimated glomerular filtration rate (eGFR) using the Chronic Kidney Disease Epidemiology Collaboration (CKD-EPI) formula ≥ 50 mL/min (refer to Appendix 1)

8) International normalized ratio (INR), or prothrombin time (PT) < 1.5 × the ULN except for patients receiving anticoagulation therapy who must be on a stable dose of warfarin for 6 weeks prior to enrollment
9) Measurable or non-measurable, but evaluable disease using RECIST v1.1

Additional Inclusion Criteria for Phase 1 Only

Patients enrolling in **Phase 1** of the study must also meet the following inclusion criteria:

10) Histologically or cytologically confirmed GI malignancy for which mFOLFOX6 is considered an appropriate treatment (eg, GC, colorectal carcinoma, pancreatic adenocarcinoma)
11) Patient must be a candidate to receive at least 2 doses of mFOLFOX6 chemotherapy

Additional Inclusion Criteria for Phase 2 Only

Patients enrolling in **Phase 2** of the study must also meet the following inclusion criteria:

14) Histologically documented gastric or gastroesophageal junction adenocarcinoma (not amenable to curative therapy)
15) Radiographic imaging of the chest, abdomen and pelvis (computed tomography [CT] preferred, magnetic resonance imaging [MRI] acceptable) performed within 28 days (+3 days) of treatment (C1D1)
16) FGFR2b overexpression as determined by a centrally performed IHC tissue test and/or *FGFR2* gene amplification as determined by a centrally performed ctDNA blood-based assay
17) Patient must be a candidate for mFOLFOX6 chemotherapy

18) No prior chemotherapy for metastatic or unresectable disease (except a maximum of 1 dose of mFOLFOX6 administered while waiting for results of FGFR2 testing during the pre-screening period)

19) If prior adjuvant or neo-adjuvant therapy (chemotherapy and/or chemoradiation) has been received, more than 6 months must have elapsed between the end of adjuvant therapy and the confirmation of radiographic disease progression

Exclusion Criteria for Phase 1 and Phase 2

Patients enrolling in either Phase 1 or Phase 2 will be excluded if *any* of the following criteria apply:

1) Untreated or symptomatic central nervous system (CNS) metastases (CNS imaging not required). Patients with asymptomatic CNS metastases are eligible provided they have been clinically stable for at least 4 weeks and do not require intervention such as surgery, radiation, or any corticosteroid therapy for management of symptoms related to CNS disease

2) Impaired cardiac function or clinically significant cardiac disease, including any of the following (Criteria a through g):

   a) Unstable angina pectoris ≤ 6 months prior to enrollment

   b) Acute myocardial infarction ≤ 6 months prior to enrollment

   c) New York Heart Association class II-IV congestive heart failure

   d) Uncontrolled hypertension (as defined as ≥ 160/90 despite optimal medical management)

   e) Uncontrolled cardiac arrhythmias requiring anti-arrhythmic therapy other than beta blockers or digoxin

   f) Active coronary artery disease

   g) Fridericia's correction formula (QTcF) ≥ 480

3) Peripheral sensory neuropathy ≥ Common Terminology Criteria for Adverse Events (CTCAE) Grade 2

4) Active infection requiring systemic treatment or any uncontrolled infection ≤ 14 days prior to enrollment

5) Known human immunodeficiency virus (HIV) or acquired immunodeficiency syndrome (AIDS)-related illness, or known active or chronic hepatitis B or C infection

6) History of interstitial lung disease (eg, pneumonitis or pulmonary fibrosis)

7) Evidence or history of bleeding diathesis or coagulopathy

8) Radiotherapy ≤ 28 days of enrollment. Patients must be recovered from all acute radiotherapy-related toxicities. No radiopharmaceuticals (strontium, samarium) within 8 weeks of enrollment
9) Prior treatment with any selective inhibitor (eg, AZD4547, BGJ398, JNJ-42756493, BAY1179470) of the FGF-FGFR pathway
10) Ongoing adverse effects from prior systemic treatment > NCI CTCAE Grade 1 (with the exception of Grade 2 alopecia and anemia)
11) Participation in another therapeutic clinical study or receiving any investigational agent within 28 days of enrollment or during this clinical study
12) Corneal defects, corneal ulcerations, keratitis, keratoconus, history of corneal transplant, or other known abnormalities of the cornea that may pose an increased risk of developing a corneal ulcer
13) Known positivity for human epidermal growth factor receptor 2 (HER2) (as defined by a positive IHC test of 3+ or IHC of 2+ with positive FISH)
14) Major surgical procedures not permitted ≤ 28 days prior to enrollment. Surgery requiring local/epidural anesthesia must be completed at least 72 hours before enrollment. In all cases, the patient must be sufficiently recovered and stable before treatment administration.
15) Women who are pregnant or breastfeeding (unless the patient is willing to interrupt breastfeeding during study treatment administration and then resume 6 months after study discontinuation); WOCBP must not consider getting pregnant during the study
16) Presence of any serious or unstable concomitant systemic disorder incompatible with the clinical study (e.g., substance abuse, psychiatric disturbance, or uncontrolled intercurrent illness including arterial thrombosis, and symptomatic pulmonary embolism)
17) Presence of any other condition that may increase the risk associated with study participation, or may interfere with the interpretation of study results, and, in the opinion of the investigator, would make the patient inappropriate for entry in the study
18) Known allergy, hypersensitivity or contraindication to components of the FPA144 formulation including polysorbate or to platinum-containing medications, 5-FU, or leucovorin
19) History of prior malignancy, except (Criteria a through f):
    a) Curatively treated non-melanoma skin malignancy
    b) Cervical cancer *in situ*

c) Curatively treated Stage I uterine cancer

d) Curatively treated ductal or lobular breast carcinoma in situ and not currently receiving any systemic therapy

e) Localized prostate cancer that has been treated surgically with curative intent and presumed cured

f) Solid tumor treated curatively more than 5 years previously without evidence of recurrence

No waivers of these inclusion or exclusion criteria will be granted.

Pharmacokinetic Assessments: Blood samples will be collected to measure serum levels of FPA144 at the time points outlined in Appendix 6 for Phase 1. Blood samples will be collected to measure levels of FPA144 at the time points outlined in Appendix 7 for Phase 2. PK parameters will be estimated using non-compartmental analysis, though compartmental analysis may be employed if appropriate. Serum concentration-time data from FPA144-004 will be pooled with data from other studies for integrated population PK analysis and exposure-response relationship assessment.

Immunogenicity Assessments: For all enrolled patients in Phase 1 and Phase 2, blood samples will be collected for anti-FPA144 antibodies at the time points specified in Appendix 6 and Appendix 7, respectively.

Immunogenicity, defined as an immune response to FPA144, will be assessed by measurement of total anti-FPA144 antibodies from all patients. Immunogenicity testing will consist of screening, confirmation, and determination of the antidrug antibody (ADA) concentration. Additional characterization of a confirmed anti-FPA144 antibody response will be conducted.

Efficacy Assessments: During Phase 2, tumor response assessment will be performed by the investigator per RECIST v1.1 guidelines. All radiology images will be analyzed by the investigator and this assessment will be used in the determination of progression.

Efficacy measures will include tumor assessments consisting of clinical examination and appropriate imaging techniques, preferably CT scans of the chest, abdomen, and pelvis with appropriate slice thickness per RECIST v1.1 guidelines. Alternatively, MRI is also acceptable at the discretion of the investigator. Scans will be done during the screening window (within 28 +3 days of C1D1). A scan performed prior to Screening as part of standard of care, performed no greater than 28 days (+3 days) prior to treatment (C1D1) is acceptable. Scans will be performed every 8 weeks (± 7 days) from C1D1 until 12 months from C1D1 and then every 12 weeks (± 14 days) thereafter.

Safety Assessments:

Safety measures will include AEs, hematology, clinical chemistry, urinalysis, vital signs, body weight, concomitant medications/procedures, ECOG performance status, targeted physical examinations, ECGs, and ophthalmology examinations in both Phase 1 and Phase 2. Safety measures will also include evaluation for DLTs in Phase 1 only.

In Phase 2, an independent Data Monitoring Committee (DMC) will evaluate safety including AEs and serious adverse events (SAEs) on a regular basis throughout the treatment phase.

Pharmacodynamic Assessments:

Phase 1

Tumor tissue provided for evaluation of FGFR2 status, if available, will be retrospectively analysed for FGFR2b overexpression using IHC.

Blood samples provided for evaluation of FGFR2 status will be collected prior to the first dose of study treatment and analysed retrospectively for *FGFR2* gene amplification using a ctDNA blood assay.

Blood samples for exploratory pharmacodynamic biomarker analysis of the FGFR pathway will be collected longitudinally according to Appendix 5.

No pharmacodynamic assessments will be performed during Phase 2.

Statistical Procedures:

Power and Sample Size

This Phase 2 study is designed to assess the hazard ratio (HR) of PFS for FPA144 + mFOLFOX6 compared with placebo + mFOLFOX6. It is planned to observe 84 PFS events in order to achieve 71% power to detect an HR of 0.67 for PFS, at the 1-sided alpha of 0.1. Assuming an exponential distribution, this corresponds approximately to an 50% increase in median PFS (e.g. from 5 months to 7.5 months). Statistical significance (at 1-sided alpha of 0.1) for PFS will occur with an observed HR=0.756, corresponding approximately to a 32.3% increase in observed median PFS (e.g. from 5 months to 6.6 months).

One hundred fifty-five patients have been randomized (1:1) during 13 months of accrual. It is projected to observe 84 PFS events with approximately 11 additional months of follow-up.

There is no planned interim analysis for this study.***Statistical Methods***

In Phase 1, all analyses will be descriptive and will be presented by dose cohort and cumulative as appropriate. Descriptive statistics will include number of observations, mean, standard deviation, median, range, and inter-quartile range for continuous variables, and the number and percent for categorical variables; 95% confidence intervals (CIs) will be presented where appropriate. Additionally, incidence of treatment-emergent adverse

events (TEAEs) leading to dosing reductions or dose discontinuation will be tabulated and summarized.

In Phase 2, efficacy and tolerability will be evaluated and presented by treatment arm (Arm 1, FPA144 + mFOLFOX6; Arm 2, placebo + mFOLFOX6).

Eligible patients will be stratified by:

- Geographic region
- Prior treatment status (*de novo* versus adjuvant/neo-adjuvant)
- Administration of a single dose of mFOLFOX6 prior to enrollment (yes or no)

In Phase 2, the primary efficacy analysis is the comparison of PFS between the treatment arms. The primary endpoint, PFS, is defined as time from randomization until the date of radiological disease progression based on investigator assessment (using Response Evaluation Criteria in Solid Tumors version 1.1 [RECIST v1.1]) or death from any cause, whichever comes first. The secondary efficacy endpoints include OS and ORR.

The analysis of PFS will be performed using the intent-to-treat (ITT) population and will be conducted using a stratified log-rank test. The stratification factors will be the same used to stratify the randomization schedule as documented in the interactive voice and web response system (IXRS).

The median PFS and the associated 95% CI for each treatment arm will be estimated using the Kaplan-Meier method. The hazard ratio (HR= $\lambda_{FPA144+mFOLFOX6}/ \lambda_{placebo+mFOLFOX6}$) will be estimated using a Cox regression model with treatment group as the only main effect and stratifying by the same stratification factors as were used for the stratified log-rank test. An unstratified HR will also be presented.

Analyses of secondary endpoints OS and ORR will be conducted hierarchically. The formal hypotheses regarding effects on OS and ORR will be tested hierarchically at a one-sided level of 0.1. The OS will be tested first and if it is significant, the ORR will be tested next. The family-wise type I error rate of testing primary and secondary endpoints will be in a control by employing this gate-keeping testing procedure at a one-sided level of 0.1.

Overall survival will be analyzed in a similar manner as for PFS.

The analysis of ORR will be performed based on the ITT population. In the analysis of ORR, patients without postbaseline adequate tumor assessments will be counted as non-responders. Formal hypothesis testing of ORR will be performed using the stratified Cochran-Mantel-Haenszel test at a one-sided level of 0.1. The stratification factors will be the same used to stratify the randomization schedule as documented in the IXRS.

Safety Analysis

All AEs will be coded using the Medical Dictionary for Regulatory Activities Version 20.1 (MedDRA v 20.1). The investigator will classify the severity of AEs using the CTCAE v 4.03 in Phase 1 and v 5.0 in Phase 2. A TEAE is defined as any event with an onset date on or after date of first dose of study treatment, or any event present before treatment that worsens after treatment.

Clinical laboratory data will be summarized by the type of laboratory test. The number and percentage of patients who experience abnormal (ie, outside of reference ranges) and/or clinically significant abnormal laboratories after study treatment administration will be presented for each clinical laboratory measurement. For each clinical laboratory measurement, descriptive statistics will be provided for baseline and all subsequent posttreatment scheduled visits. Changes from baseline to the posttreatment visits will also be provided. Descriptive statistics of vital signs will also be provided in a similar manner. In addition, shift from baseline in CTCAE grade (where applicable) and by high/low flags (where CTCAE grades are not defined) will be presented in a similar manner.

Safety Analyses in Phase 1

Safety analyses will be performed for patients included in the safety population. The incidence of DLTs, incidence of TEAEs, clinical laboratory abnormalities (eg, shift table), vital signs, corneal and retinal findings, and ECGs will be tabulated and summarized by dose level. Additionally, incidence of TEAEs leading to dosing reduction or dose discontinuation of FPA144 or any component of mFOLFOX6 will be tabulated and summarized.

Safety Analyses in Phase 2

The analyses of safety will include all patients who receive any study treatment (FPA144 + mFOLFOX6 or placebo + mFOLFOX6) throughout the study and will provide posttreatment safety information. The incidence of TEAEs, clinical laboratory abnormalities, vital signs, corneal and retinal findings, and ECGs will be tabulated and summarized by treatment group.

No formal comparisons of safety endpoints are planned.

PK Analysis and Immunogenicity

Individual and mean (± SD) serum FPA144 concentration-time data will be tabulated and plotted by dose level. PK parameters will be tabulated and summarized by dose level when appropriate and applicable. The impact of immunogenicity on FPA144 exposure will be assessed, tabulated, and summarized by dose level as data allow. Integrated population PK analysis and exposure-response relationship assessment will be presented in a separate report.

List of Key Study Personnel

Sponsor:	Five Prime Therapeutics, Inc.<br>[redacted]
Responsible Medical Officers:	[redacted]<br>Five Prime Therapeutics, Inc.<br>[redacted]
	[redacted]<br>Five Prime Therapeutics, Inc.<br>[redacted]
Contract Research Organization:	ICON Clinical Research Limited<br>[redacted]
Serious Adverse Event Reporting:	ICON Medical and Safety Services<br>[redacted]

TABLE OF CONTENTS

PROTOCOL APPROVAL SIGNATURE PAGE 2
DECLARATION OF THE INVESTIGATOR 3
PROTOCOL SYNOPSIS 4
LIST OF KEY STUDY PERSONNEL 22
TABLE OF CONTENTS 23
LIST OF TABLES 28
LIST OF FIGURES 28
LIST OF APPENDICES 28
LIST OF ABBREVIATIONS AND DEFINITIONS 29
1.0 INTRODUCTION 33
1.1 General Gastric Cancer 33
1.2 Targeted Agents 33
1.3 FPA144 34
1.3.1 Background 34
1.3.2 FPA144, an Fibroblast Growth Factor Receptor 2-Specific Antibody 35
1.3.3 Nonclinical Studies with FPA144 36
1.3.3.1 In Vivo Pharmacology 36
1.3.3.2 Analysis of Immune Effector Functions of FPA144 37
1.3.3.3 FPA144 Exposure Efficacy Relationships 39
1.3.4 Toxicology 40
1.3.5 Clinical Experience with FPA144 41
1.4 Rationale for mFOLFOX6 44
1.5 Rationale for Combination Therapy: FPA144 and mFOLFOX6 45
1.6 FPA144 and mFOLFOX6 Starting Dose Justification 46
1.7 Risk-Benefit Assessment of FPA144 and mFOLFOX6 47
1.8 Rationale for Phase 2 Pre-Screening 49
1.9 Rationale for Tumor Tissue and Blood Assessments 50
2.0 STUDY OBJECTIVES AND ENDPOINTS 51
2.1 Phase 1 Objectives 51
2.1.1 Primary 51
2.1.2 Secondary 51
2.1.3 Exploratory 51
2.2 Phase 2 Objectives 51
2.2.1 Primary 51

2.2.2 Secondary 51
2.2.3 Exploratory 51
2.3 Phase 1 Endpoints 52
2.3.1 Primary 52
2.3.2 Secondary 52
2.3.3 Exploratory 52
2.4 Phase 2 Endpoints 52
2.4.1 Primary 52
2.4.2 Secondary 52
2.4.3 Exploratory 53
3.0 STUDY DESIGN 54
3.1 Study Overview 54
3.2 Phase 1 55
3.2.1 Dose Escalation 55
3.2.2 Definition of Dose-Limiting Toxicity 56
3.2.3 Observation of Dose-Limiting Toxicity (the DLT Period) 57
3.2.4 Determination of Maximum Tolerated Dose and Recommended Dose 58
3.2.5 Phase 2 Dose of FPA144 59
3.2.6 Patient Replacement 59
3.2.7 Number of Doses 59
3.3 Phase 2 60
3.3.1 Treatment Arms 60
3.4 Study Schemas 62
3.5 Rationale for the Study Design 63
3.5.1 Rationale for Selection of Patients for FGFR2 63
3.6 Rationale for Stratification 64
3.6.1 Geographic Region 64
3.6.2 Prior Therapy 64
3.6.3 Administration of mFOLFOX6 Prior to Enrollment 64
4.0 STUDY ELIGIBILITY AND WITHDRAWAL CRITERIA 65
4.1 Planned Number of Patients and Study Centers 65
4.2 Inclusion Criteria for Phase 1 and Phase 2 65
4.3 Exclusion Criteria for Phase 1 and Phase 2 67
4.4 Patient Identification and Enrollment 69
4.5 Patient Withdrawal and Replacement 69

5.0 STUDY TREATMENT 71
5.1 FPA144 71
5.1.1 Identity, Packaging, Storage 71
5.1.2 Administration (Phase 1 and Phase 2) 71
5.1.3 FPA144 Dose Modifications 72
5.1.3.1 Dose Escalation 72
5.1.3.2 Weight 72
5.1.3.3 Infusion or Hypersensitivity Reactions 72
5.1.3.4 Toxicity: FPA144 Dose Modification Guidelines 73
5.2 mFOLFOX6 75
5.2.1 Identity, Packaging, Storage 75
5.2.2 Administration (Phase 1 and Phase 2) 75
5.2.3 mFOLFOX6 Dose Modifications 76
5.2.3.1 Toxicity: mFOLFOX6 Dose Modifications Guidelines 76
5.3 Placebo 79
5.4 Unblinding Treatment Assignment 79
5.5 Drug Accountability 79
5.6 Investigational Product Compliance 80
5.7 Concomitant Medication and Treatment 80
5.8 Prohibited Therapy 80
6.0 STUDY ASSESSMENTS AND PROCEDURES 81
6.1 Tumor Tissue Collection: Analysis for FGFR2b Overexpression for Patient Selection 81
6.1.1 Phase 1 81
6.1.2 Phase 2 81
6.2 Informed Consent Requirements 82
6.3 Screening 82
6.3.1 Phase 1 82
6.3.2 Phase 2 83
6.4 Study Enrollment 83
6.4.1 Phase 1 83
6.4.2 Phase 2 84
6.4.3 Blinding 84
6.5 Medical/Oncology History and Demographics 84
6.6 Safety Assessments 85
6.6.1 Vital Signs 85

6.6.2 Physical Examinations, Height, and Weight 85
6.6.3 Ophthalmologic Examinations 85
6.6.4 Electrocardiograms 86
6.6.5 Eastern Cooperative Oncology Group Performance Status 86
6.7 Clinical Laboratory Parameters 87
6.8 Urinalysis 87
6.9 Pregnancy 87
6.10 Tumor Assessments 88
6.11 Pharmacokinetic Assessment 89
6.12 Pharmacodynamic Biomarker Analysis Using Blood 89
6.13 Immunogenicity 90
6.14 Immunohistochemistry 90
6.15 Circulating Tumor DNA Blood Assay 90
6.16 Patient Reported Outcomes / Quality of Life Scales 90
6.17 Adverse Events 91
6.17.1 Collection of Adverse Events 91
6.17.2 Definitions 92
6.17.3 Assessment of Adverse Events 92
6.17.3.1 Seriousness 93
6.17.3.2 Causality 94
6.17.3.3 Outcome and Action Taken 95
6.17.4 Reporting Serious Adverse Events 96
6.17.5 Follow-up of Adverse Events 97
6.18 End of Treatment 98
6.19 Long Term Follow Up (Phase 2) 98
6.19.1 Additional Follow-up for Patients without Progression at the End of Treatment Visit (Phase 2) (Scan Follow-up) 98
7.0 STATISTICAL METHODS 99
7.1 Analysis Populations 99
7.2 General Considerations 99
7.3 Power and Sample Size in Phase 2 99
7.4 Efficacy Analyses 100
7.4.1 Analysis of Primary Efficacy Endpoint 100
7.4.2 Analysis of Secondary Efficacy Endpoints 101
7.4.3 Analysis of Exploratory Endpoints 102
7.4.3.1 Efficacy 102

7.4.3.2 Quality of Life....102
7.5 Safety Analyses....102
7.5.1 Safety Analyses in Phase 1....103
7.5.2 Safety Analyses in Phase 2....103
7.6 Pharmacokinetic Analyses....103
7.7 Changes in the Planned Analyses....104
8.0 ETHICAL, LEGAL, AND ADMINISTRATIVE ASPECTS....105
8.1 Data Quality Assurance....105
8.2 Data Protection....105
8.3 Electronic Case Report Forms and Source Documentation....106
8.4 Access to Source Data....106
8.5 Data Processing....107
8.6 Archiving Study Records....107
8.7 Good Clinical Practice....107
8.8 Informed Consent....108
8.9 Protocol Approval and Amendment....108
8.10 Study Committee and Central Review....108
8.10.1 Cohort Review Committee....108
8.10.2 Data Monitoring Committee....109
8.11 Premature Termination of the Study....109
8.12 Confidentiality....109
8.13 Other Ethical and Regulatory Issues....110
9.0 REFERENCES....111
10.0 APPENDICES....117

LIST OF TABLES

Table 1: Phase 1 Proposed Dose Levels by Cohort 56
Table 2: Phase 1 Dose Escalation Algorithm 58
Table 3: Phase 2 Treatment Arms 61
Table 4: Eligibility Based on FGFR2 Status 69
Table 5: Phase 2 FPA144 Dose Levels for Dose Reductions 73
Table 6: Dose Modification Guidelines for FPA144 Related Adverse Events (Any Noncorneal, Noninfusion Toxicity[1]) 74
Table 7: Dose Modification Guidelines for FPA144 (Any Related Corneal Toxicity1) 74
Table 8: Dose Reductions and Delays for mFOLFOX6 Chemotherapy 78

LIST OF FIGURES

Figure 1: Tumor Growth Inhibition in OCUM-2 Gastric Cancer Cell Line 36
Figure 2: *In Vitro* ADCC Activity of FPA144 38
Figure 3: FPA144, but not an ADCC-Deficient FGFR2b Antibody Leads to Tumor Suppression in a Syngeneic Tumor Model with Modest FGFR2b Expression 39
Figure 4: Phase 1/2 Study Schema 62
Figure 5: Phase 1 Patient Timeline 62
Figure 6: Phase 2 Patient Timeline 63

LIST OF APPENDICES

Appendix 1: Formulas to Calculate Renal Function 118
Appendix 2: Schedule of Assessments: Dose-Escalation Safety Run-in (Phase 1) 119
Appendix 3: Schedule of Assessments: Randomized, Placebo-Controlled Portion (Phase 2) 123
Appendix 4: Laboratory Evaluations 128
Appendix 5: Clinical Fixed Time Point Assessments 129
Appendix 6: Study Flowchart for Pharmacokinetic, Immunogenicity, and Exploratory Pharmacodynamic Biomarker Blood Sample Collections for Phase 1 131
Appendix 7: Study Flowchart for FPA144 Pharmacokinetic and Immunogenicity Blood Sample Collections for Phase 2 133
Appendix 8: ECOG Performance Status 134
Appendix 9: Fluorescein Stain Grading Systems 135

List of Abbreviations and Definitions

Abbreviation	Definition
ADCC	antibody-dependent cell-mediated cytotoxicity
ADA	antidrug antibody
AE	adverse event
AESI	adverse event of special interest
AIDS	acquired immunodeficiency syndrome
ALT	alanine aminotransferase
ANC	absolute neutrophil count
AST	aspartate aminotransferase
AUC	area under serum concentration-time curve
AUC_τ	AUC over the dose interval τ
β-hCG	β-human chorionic gonadotropin
BP	blood pressure
C1D1	Cycle 1 Day 1
CDC	complement-dependent cytotoxicity
CFR	Code of Federal Regulations
CHO	Chinese hamster ovary
CI	confidence interval
CL	total clearance
C_{max}	maximum observed serum concentration
CNS	central nervous system
CRC	Cohort Review Committee
CrCl	creatinine clearance
CRO	contract research organization
CT	computed tomography
CTCAE	Common Terminology Criteria for Adverse Events
ctDNA	circulating tumor DNA
DLT	dose-limiting toxicity
DMC	Data Monitoring Committee
DNA	deoxyribonucleic acid
DOR	duration of response
ECG	electrocardiogram
ECOG	Eastern Cooperative Oncology Group
eCRF	electronic case report forms
ELISA	enzyme linked immunosorbent assay
EORTC	European Organization for Research and Treatment of Cancer

Abbreviation	Definition
EOS	end of study
EOT	end of treatment
EQ-5D-5L	EuroQOL-5D-5L
EU	European Union
5-FU	5-fluorouracil
FDA	Food and Drug Administration
FGF	fibroblast growth factor
FGFR	fibroblast growth factor receptor
FISH	fluorescent in situ hybridization
FP	5-FU/cisplatin
FRS2	FGF receptor substrate-2
GC	gastric or gastroesophageal cancer
GCP	Good Clinical Practices
G-CSF	granulocyte-colony stimulating factor
GEJ	gastroesophageal junction
GI	gastrointestinal
GLP	GLP Good Laboratory Practices
HER2	human epidermal growth factor receptor 2 also known as ERBB2
hFc-G1	Fc fragment of human IgG1
HIV	human immunodeficiency virus
HNSTD	highest, non-severely toxic dose
HR	hazard ratio
HR	heart rate
HR	hazard ratio
IB	Investigator’s Brochure
ICF	informed consent form
ICH	International Conference on Harmonization
IEC	Independent Ethics Committee
IgG1	humanized monoclonal antibody
IHC	immunohistochemistry
IND	investigational new drug (application)
INR	international normalized ratio
IOP	intra-ocular pressure
IRB	Institutional Review Board
ITT	intent-to-treat
IV	intravenous
IXRS	interactive voice and Web response system

Abbreviation	Definition
LTFU	long term follow up
MedDRA	Medical Dictionary for Regulatory Activities
mFOLFOX6	modified FOLFOX (infusional 5-FU, leucovorin, and oxaliplatin)
mOS	median OS
mPFS	median progression free survival
MRI	magnetic resonance imaging
MTD	maximum tolerated dose
NCBI	National Center for Biotechnology Information
NCI	National Cancer Institute
NK	natural killer
OCT	ocular coherence tomography
ORR	objective response rate
OS	overall survival
PBMC	peripheral blood mononuclear cell(s)
PFS	progression-free survival
PK	pharmacokinetic(s)
PRO	patient reported outcomes
Q2W	once every 2 weeks
QLQ	quality of life questionnaire
QLQ-C30	Quality of Life Questionnaire Version 3.0
QOL	quality of life
QTcF	Fridericia’s correction formula
RD	recommended dose
RECIST	Response Evaluation Criteria in Solid Tumors
ROW	Rest of World
RPE	retinal pigment epithelium
RR	respiratory rate
SAE	serious adverse event
SAP	statistical analysis plan
SD	standard deviation
SEM	standard error of the mean
SmPCs	summaries of product characteristics
$t_{1/2}$	terminal half-life
TEAE	treatment-emergent adverse event
TKI	tyrosine kinase inhibitor
ULN	upper limit of normal
US	United States

**Abbreviation**	**Definition**
VAS	visual analogue scale
VEGF	vascular endothelial growth factor
WOCBP	women of childbearing potential

1.0 INTRODUCTION

1.1 General Gastric Cancer

Gastric or gastroesophageal cancer (GC), including gastroesophageal junction (GEJ) cancer, represents the fourth most common cancer worldwide (Kamangar 2006) and is a highly lethal disease, with 5-year overall survival (OS) rates below 30% in the United States (US) regardless of stage (National Cancer Institute 2015). Though intensive multimodal therapy for locoregional disease improves survival (The GASTRIC Group 2010, Waddell 2014) it does not cure most patients and chemotherapy for metastatic disease provides only short-term benefits (Wagner 2006, Waddell 2014). First-line chemotherapy used in patients with metastatic or recurrent disease consists of a fluoropyrimidine (5-fluorouracil [5-FU] or capecitabine) with a platinum agent (usually oxaliplatin or cisplatin) (Al-Batran 2008, Kang 2009). This treatment prolongs survival by 6 months compared to best supportive care (Wagner 2006), but the benefits are only short-term with a median OS (mOS) of 9 to 10 months and a progression-free survival (PFS) of 5 to 5.6 months (Kang 2009, Waddell 2014).

1.2 Targeted Agents

It is important to identify new treatments with acceptable toxicities for this patient population. Recent studies have identified important pathways involved in GC development. The availability of targeted agents has led to the development of strategies incorporating these agents in the therapy for patients with such a poor prognosis. Recently ramucirumab (a monoclonal antibody targeting the vascular endothelial growth factor [VEGF] pathway) was approved for treatment in patients with GC who progressed following first line treatment (Wilke 2014). OS for patients treated with ramucirumab with paclitaxel was 9.6 months compared to 7.4 months with paclitaxel and placebo.

One well established pathway is the human epithelial growth factor receptor 2 (HER-2 also known as ERBB2) (Gravalos 2008, Hofmann 2008). HER-2 overexpression has been identified in 9 to 38% of GCs depending on histology and tumor location (Gravalos 2008). The availability of trastuzumab, a monoclonal antibody targeting HER-2, led to the development of a randomized trial in newly diagnosed GC patients whose tumors overexpressed HER-2. About 22% of screened patients were HER-2 positive and the combination of standard chemotherapy with trastuzumab resulted in a median OS of 13.8 months compared to 11.1 months for patients treated with standard chemotherapy (Bang 2010). Based on these results, this has become standard care for patients overexpressing HER-2.

In spite of these improvements, the majority of GC patients succumb to their disease, and there are few treatment options following progression after first-line chemotherapy. Recently anti-PD1 therapies have been approved for treatment of later line GC in Japan and the United States. In September 2017, Japanese Ministry of Health, Labor and Welfare approved the PD1 inhibitor nivolumab (Opdivo®) for the treatment of unresectable advanced or recurrent gastric cancer which has progressed after chemotherapy. This approval was based on the Phase 3 study

ATTRACTION-2 (ONO-4538-12), in which Opdivo® significantly reduced patients' risk of death by 37% (HR 0.63 [95% CI: 0.51-0.78, p<0.0001]) when compared to placebo (Kang 2017). Additionally, in September 2017, the United States Food and Drug Administration (FDA) granted accelerated approval to pembrolizumab (KEYTRUDA®) (Fuchs 2017) for patients with recurrent locally advanced or metastatic, gastric or gastroesophageal junction adenocarcinoma whose tumors express PD-L1. Patients must have had disease progression on or after 2 or more prior systemic therapies, including fluoropyrimidine- and platinum-containing chemotherapy and, if appropriate, HER2/neu-targeted therapy. Pembrolizumab approval was based on data from 143 patients with tumors expressing PD-L1 and who were either microsatellite stable (MSS) or had unknown microsatellite instable (MSI) or mismatch repair deficient (dMMR) status, who demonstrated an objective response rate (ORR) of 13.3% (95% CI: 8.2, 20.0) and a duration of response (DOR) ranging from 2.8+ to 19.4+ months. The majority of patients, however, do not respond to anti-PD1 therapy and other late line therapies such as ramucirumab with or without paclitaxel, and irinotecan improve the mPFS by less than 2 months (Thuss-Patience 2011, Ford 2014, Fuchs 2014). Therefore, continued evaluation of agents that can target the mutations present in GC is imperative.

Amplification of fibroblast growth factor receptor 2 (FGFR2) has recently been identified as having prognostic importance in patients with GC (Jung 2012, Matsumoto 2012, Su 2014, Seo 2016). Patients with *FGFR2* gene amplification appear to have a worse prognosis (Su 2014, Seo 2016), suggesting that inhibition of FGFR2 may be an important target (Jung 2012, Matsumoto 2012). FPA144 is a humanized monoclonal antibody specific to the human FGFR2b receptor that blocks fibroblast growth factor (FGF) binding to the receptor. Evaluation of this agent in patients with GC whose tumors have alterations of FGFR2 would be an important strategy to improve the prognosis for these patients.

1.3 FPA144

1.3.1 Background

The role of the FGFR pathway in cancer is well known. FGFs can stimulate the transformation and proliferation of tumor cells and stimulate angiogenesis. There are 22 known human FGFs with the expression of individual FGFs generally restricted to specific tissues, cell types, and/or developmental stage. FGF signaling is mediated by a family of transmembrane tyrosine kinase receptors encoded by 4 distinct genes producing FGF receptor subtypes termed FGFR1–4 (Turner 2010).

The FGFR2 has 2 splicing variants, b and c. In general, FGFR2b is expressed in tissues of epithelial origin (eg, stomach, skin) (Miki 1992). The major ligands signaling through FGFR2b are FGF7, FGF10 and FGF22. Alteration in signaling in the FGF/FGFR2 pathway (eg, overexpression of FGFR2 protein or amplification of *FGFR2* gene) has been associated with gastric, breast, and other cancers, and appears to portend a worse prognosis (Turner 2010, Wu 2013). As early as 1990, subsets of patients with GC (approximately 3 to 9%) and breast cancer (1 to 2%) were noted to have amplification of the *FGFR2* gene, which resides on

chromosome 10q26 (Hattori 1990, Turner 2010). In GC, *FGFR2* gene amplification leads to high-level expression of the FGFR2b receptor on the surface of the cells.

1.3.2 FPA144, an Fibroblast Growth Factor Receptor 2-Specific Antibody

FPA144 is a humanized monoclonal antibody (IgG1 isotype) specific to the human FGFR2b receptor (National Cancer Institute 2015) that blocks FGF ligand binding to the receptor. FPA144 is directed against the third Ig region of the FGFR2b receptor isoform, the region that is alternatively spliced and regulates ligand specificity. This antibody is glycosylated but is produced in a Chinese hamster ovary (CHO) cell line that lacks the *FUT8* gene (α1,6-Fucosyltransferase) and therefore lacks a core fucose in the polysaccharide portion of the antibody. The absence of the core fucose results in higher affinity for the Fc receptor FcγRIIIa compared to the fucosylated molecule and potentially enhances immune cell-mediated tumor cell killing (Shinkawa 2003). The antibody has thus been glycoengineered for enhanced antibody-dependent cell-mediated cytotoxicity (ADCC) (Gemo 2014). FPA144 inhibits FGF ligand-stimulated FGFR2b phosphorylation and cell proliferation in cell culture in FGFR2b overexpressing gastric and breast cancer cell lines. FPA144 also inhibits tumor growth in FGFR2b overexpressing gastric and breast xenograft models. The 3 potential mechanisms of action of FPA144 thus include blocking ligand binding and downstream signaling, decreasing expression of the FGFR2b driver protein, and enhancing ADCC.

FPA144 can produce complete and durable tumor growth inhibition in FGFR2b-overexpressing and *FGFR2* gene-amplified GC xenografts in immune-compromised mice where FGFR2b is considered a driver of tumor growth (Gemo 2014). In addition, FPA144 demonstrates recruitment of natural killer (NK) cells and concomitant tumor growth inhibition in the 4T1 syngeneic tumor model with modest expression of FGFR2b (Powers 2016). These data suggest that ADCC may be efficacious in patients without *FGFR2* gene amplification with moderate FGFR2b overexpression, and that ADCC activity may be a major contributor to the mechanism of action in these patients.

Additionally, since FPA144 is specific for the FGFR2b receptor, it does not interfere with signaling of the other FGFs/ FGFRs, including FGFR2c. In contrast to the FGFR tyrosine kinase inhibitors (TKIs), FPA144 does not inhibit FGF23 signaling. FGF23 is a ligand involved in calcium/phosphate metabolism. Thus, treatment with FPA144 is not expected to cause the dose-limiting hyperphosphatemia associated with the FGFR TKIs (Brown 2005, Andre 2013, Dienstmann 2014, Sequist 2014).

As FPA144 is a targeted biologic, the clinical development of FPA144 will ultimately be in selected patients with alterations in the FGFR2 pathway that are most likely to respond to this novel agent. The tumor types most relevant to date include gastric, bladder, and possibly cholangiocarcinoma. Each of these cancers needs new therapeutic options. The FPA144-004 study is designed to evaluate the efficacy, safety, and PK of FPA144 in combination with modified FOLFOX (infusional 5-FU, leucovorin, and oxaliplatin) (mFOLFOX6) chemotherapy

treatment. Patients with gastrointestinal (GI) tumors will be enrolled in a Phase 1 safety run in, while the Phase 2 will enroll GC patients specifically selected for FGFR2 expression and/or *FGFR2* gene amplification (FGFR2 selected) who are eligible for first-line mFOLFOX6 chemotherapy.

1.3.3 Nonclinical Studies with FPA144

The 3 mechanisms of action of FPA144 described above have been explored both in vitro and in vivo.

1.3.3.1 In Vivo Pharmacology

FPA144 has been studied in a series of mouse xenograft models using human gastric and breast tumor cell lines that contain the *FGFR2* amplicon. These *FGFR2* amplified lines all express high levels of the FGFR2b protein and respond to FPA144 in a dose-dependent fashion. A dose response study (twice weekly dosing) was performed with the most sensitive model, a GC line, OCUM-2 (Figure 1). Mice were treated at the indicated concentrations of FPA144, and the tumor growth was compared to mice treated with albumin alone. Statistically significant tumor growth inhibition was seen at 0.3 mg/kg, but not at 0.1 mg/kg, and tumor regression was seen at 1 mg/kg with complete tumor regression starting at doses of 1.5 mg/kg (2/15 animals), 2 mg/kg (1/15 animals), 3 mg/kg (5/15 animals), and 5 mg/kg (8/15 animals). In the SNU-16 GC model, tumor growth inhibition was seen at 1 mg/kg, while in the MFM-223 tumor-bearing mice, 5 mg/kg led to tumor stasis.

Figure 1: Tumor Growth Inhibition in OCUM-2 Gastric Cancer Cell Line

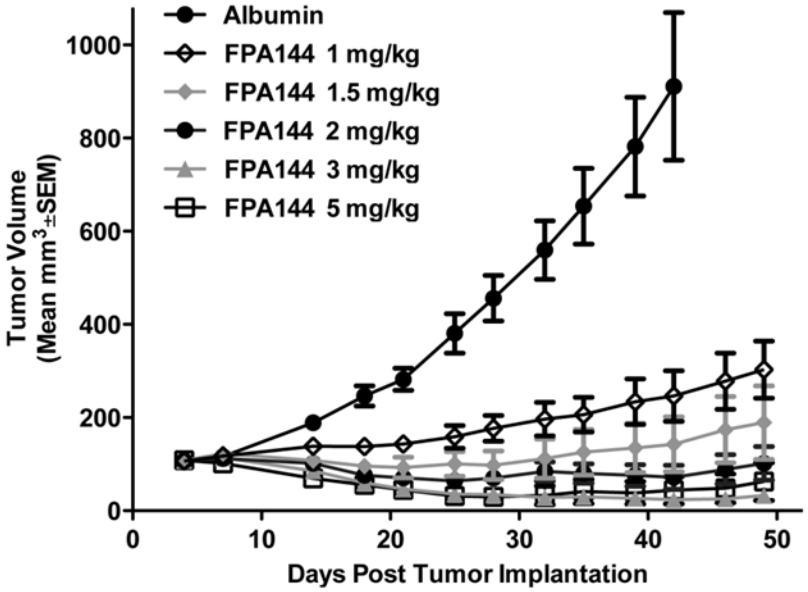

These tumor models require immunodeficient mice for tumor engraftment. Because these mice lack a fully functioning immune system, and because the mouse Fcγ receptor (the receptor on immune cells required for ADCC) has lower affinity for human antibodies than the human Fcγ receptor, ADCC is impaired in these models of FPA144 mediated tumor growth inhibition. Thus, in patients with FGFR2 overexpressing tumors, ADCC may further contribute to antitumor activity in the clinical setting. To understand the contribution of Fc receptor engagement and ADCC on FPA144 antitumor efficacy, a mutant antibody was engineered that cannot bind Fc receptors, thereby rendering it incapable of promoting ADCC. The syngeneic 4T1 model of breast cancer was employed in immune competent mice that express FGFR2b, but to a lesser degree than amplified or overexpressed FGFR2b xenografts, such as OCUM-2M or SNU-16 xenograft tumors. 4T1 tumors were grown to a volume of approximately 100 mm^3, sorted into groups of equivalent tumor volume, then treated bi-weekly with FPA144, the ADCC-deficient FGFR2b antibody, or the Fc fragment of human IgG1 (hFc-G1) as control. FPA144 decreased tumor burden by 30% versus hFc-G1 control ($p=0.001$) while the mutant antibody showed no effect. These data support a role for ADCC activity through Fc receptor binding as a required mechanism for FPA144 efficacy in a model that expresses modest amounts of FGFR2b protein.

Mechanistically, FPA144 blocks FGFR2b phosphorylation, downregulates the receptor and inhibits downstream signaling. The effect on downstream signaling was measured by examing phosphorylation of a protein that is directly phosphorylated by the FGFR2 protein, FGF receptor substrate-2 (FRS2). This has been demonstrated in the SNU-16 *FGFR2*-amplified GC xenograft model. In this experiment, mice were treated twice weekly with 10 mg/kg FPA144. When tumors had reached approximately 500 mm^3, the animals were sacrificed and protein levels in tumors were measured via Western Blotting. FPA144 treatment resulted in decreased FGFR phosphorylation, total receptor expression, and phosphorylation of the downstream signal transduction molecule, FRS2.

In contrast to the results with *FGFR2*-amplified GC models, FPA144 has minimal impact on xenograft models that are not *FGFR2* amplified or do not express the FGFR2b protein. Mice bearing NCI-87 gastric tumors, which do not express FGFR2b, were dosed intraperitoneally twice a week with FPA144 once the tumors reached approximately 100 mm^3. The tumor growth rate was indistinguishable between animals treated with either FPA144 (5 mg/kg) or control animals administered albumin.

1.3.3.2 Analysis of Immune Effector Functions of FPA144

Some therapeutic antibodies containing IgG1 Fc are capable of recruiting immune effector function, specifically ADCC and complement-dependent cellular toxicity. Once antibodies of the IgG1 isotype bind to their target on tumor cells, immune cells which express the Fc gamma receptor IIIa (FcγRIIIa), especially NK cells and macrophages, are recruited to the tumor cells and promote cell death in a process known as ADCC. FPA144 is specifically engineered for enhanced ADCC. This antibody lacks a core fucose in the polysaccharide portion of the antibody, and the lack of fucose results in higher affinity of FPA144 for FcγRIIIa compared to

the fucosylated molecule and potentially enhanced immune cell mediated tumor cell killing. In some in vitro studies, including ADCC assays, and in vivo studies, including toxicology studies, the fucosylated form of FPA144 (FPA144-F) was compared to the afucosylated form (FPA144).

FPA144 was compared to FPA144-F for in vitro ADCC activity. The target cell in the assay was an engineered cell line that expresses the full-length human FGFR2b described as Ba/F3 FGFR2b, and the effector cells were peripheral blood mononuclear cells (PBMCs) obtained fresh from individual human donors. As a negative control, FPA144 was also tested using a target cell line that was engineered to express the FGFR2c variant of the receptor (Ba/F3 FGFR2c cells), to which FPA144 does not bind. The data are shown in Figure 2. FPA144 and FPA144-F both showed ADCC activity, but the degree to which FPA144 killed the target cells was substantially greater than what was measured for FPA144-F. As expected, FPA144 showed no ADCC activity in the negative control.

Figure 2: *In Vitro* ADCC Activity of FPA144

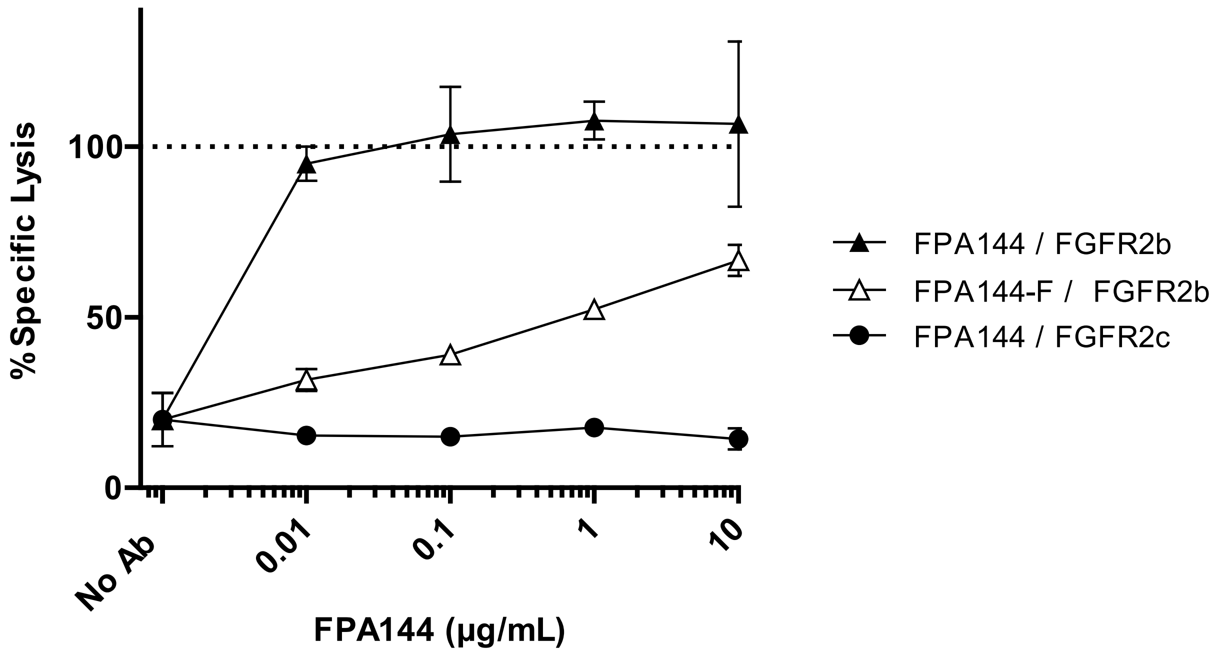

Abbreviations: ADCC = antibody-dependent cell-mediated cytotoxicity; FGFR = fibroblast growth factor receptor; No Ab = no antibody.

The ability of FPA144 to mediate complement-dependent cytotoxicity (CDC) of 4 gastric cell lines with high FGFR2b was tested using previously published methods (Li 2009, Zhao 2010). No CDC was observed under any conditions tested, although positive controls (rituximab tested with RAMOS and RAJI cells) did induce CDC (these data not shown).

1.3.3.2.1 In vivo Antibody-Dependent Cell-Mediated Cytotoxicity Activity of FPA144

To understand the contribution of ADCC on FPA144 antitumor efficacy, a mutant antibody, FPA144 N297Q, which cannot bind Fc receptors, was compared to FPA144 in the syngeneic 4T1 model that expresses FGFR2b, but to a lesser degree than amplified or overexpressed FGFR2b xenografts, such as OCUM-2M or SNU-16 xenograft tumors. 4T1 tumors were grown to a volume of approximately 100 mm3, then treated with FPA144, FPA144 N297Q, or the hFc-IgG1 as control. FPA144 decreased tumor burden versus hFc-IgG1 control while FPA144 N297Q showed no effect (Figure 3). These data support a role for ADCC activity through Fc receptor binding as a required mechanism for FPA144 efficacy in a model that expresses modest amounts of FGFR2b protein.

Figure 3: FPA144, but not an ADCC-Deficient FGFR2b Antibody Leads to Tumor Suppression in a Syngeneic Tumor Model with Modest FGFR2b Expression

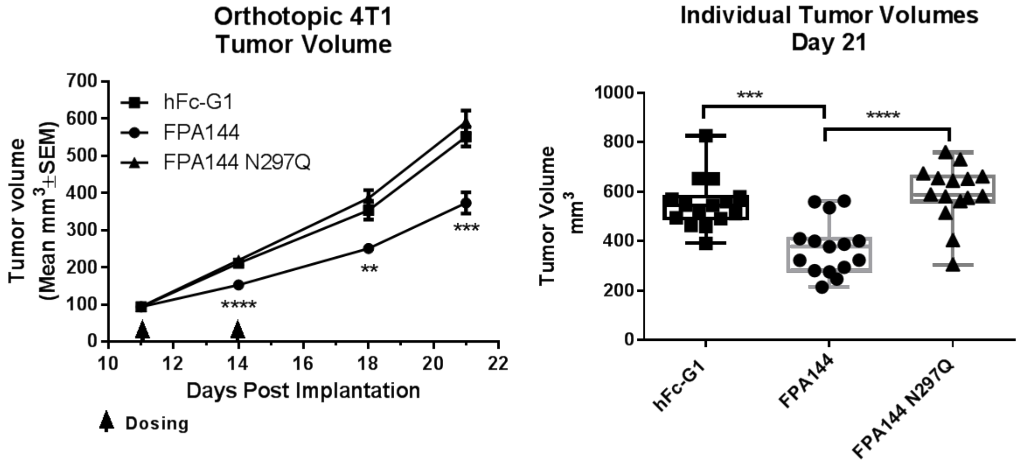

1.3.3.3 FPA144 Exposure Efficacy Relationships

To translate the efficacy results in animal models to cancer patients, the relationship between FPA144 trough concentrations and efficacy in animal models was examined. Intraperitoneal FPA144 doses of 1 mg/kg twice weekly were associated with tumor growth inhibition while greater efficacy depicted by tumor regression was noted at doses ≥ 3 mg/kg. A dose of 1 mg/kg in the mouse xenograft model led to steady state trough plasma concentrations of about 1 µg/ml, while 3 mg/kg resulted in significant tumor regression and trough plasma concentrations of 67 to 109 µg/mL.

1.3.4 Toxicology

Toxicology studies with FPA144 have been performed in rat and cynomolgus monkey. The studies performed have included pilot single dose PK/tolerability and repeat-dose studies as well as Good Laboratory Practices (GLP) repeat-dose studies. The longest of these studies involved intravenous (IV) administration of 13 weekly doses in rats and monkeys.

In pilot repeat-dose toxicology studies, rats and cynomolgus monkeys received 4 weekly IV doses of FPA144 up to 150 mg/kg. There were no changes in clinical signs and symptoms or clinical chemistry. The most significant findings from these repeat-dose pilot studies were microscopic findings in corneal epithelium. FPA144-treated animals displayed a dose-dependent thinning that represents both attenuation and reduction in the number of cells present in the corneal epithelium. In addition, microscopic changes in the retinal pigment epithelium (RPE) in rat were noted that included RPE atrophy in 1 high-dose animal that received 4 weekly 150 mg/kg doses. Retinal changes were not observed in the 13-week GLP toxicology studies with a high dose of 100 mg/kg.

In the 13-week repeat-dose GLP toxicology studies, FPA144 was administered by IV at dose levels of 1, 5, or 100 mg/kg/dose to both rats and monkeys for 13 weekly doses.

In the rat, FPA144 resulted in adverse findings including: tooth (incisor) abnormalities (clinical, macroscopic, and microscopic findings) and body weight loss/lack of weight gain, which were most likely secondary to the tooth findings that necessitated early euthanasia at the 100 mg/kg/dose, ocular findings (ophthalmic and microscopic findings), and macroscopic and/or microscopic findings in the Harderian gland (not present in humans) and oral mucosa (hard palate) at 5 and 100 mg/kg/dose, and macroscopic and/or microscopic findings in the tongue at all dose levels. FGFR2 pathway signaling is known to play a critical role in maintaining the health of rat incisors, but has not been found to be relevant in human dentition. FPA144-related, but non-adverse microscopic findings, were also noted in the mammary gland of animals at all dose levels. Administration of FPA144 also resulted in exacerbation of background microscopic findings in the prostate gland of males given 1, 5, and 100 mg/kg, the non-glandular stomach of animals given 5 and 100 mg/kg/dose, and the lung of animals given 100 mg/kg/dose. With the exception of FPA144-related effects on incisor teeth, some degree of recovery up to total recovery was evident for all findings at the end of recovery. The absence of FPA144-related findings in the eye (ophthalmic or microscopic findings), Harderian gland, mammary gland, and prostate gland at the end of the recovery period indicated complete reversibility of the findings in these tissues. Since all findings in the 1 mg/kg/dose group were minimal, without clinical consequences, and recoverable, the highest, non-severely toxic dose (HNSTD) in rats was determined to be 1 mg/kg/dose when given weekly for 13 weeks. The lowest dose of 1 mg/kg/dose level was associated with mean maximum observed serum concentration (C_{max}) and area under serum concentration-time curve (AUC) over the dose interval τ ($AUC_τ$) (τ=168 hours) of 27.7 µg/mL and 789 h*µg/mL, respectively, for combined sexes on Day 85 of the dosing phase.

In the 13-week repeat-dose GLP toxicology study performed in cynomolgus monkeys, FPA144 was generally well tolerated. FPA144-related effects were limited to microscopic findings of corneal atrophy (slight to moderate) in animals given 5 and 100 mg/kg/dose and mammary gland atrophy (moderate to marked severity) in females from all dose groups. These findings in the cornea and mammary gland were not associated with clinical sequelae and were not observed at the end of the recovery phase, indicating complete recovery. Therefore, based on the lack of correlative clinical findings or changes (eg, ophthalmic findings or clinical observations) and the demonstrated reversal during a recovery period, neither finding was considered adverse. The 100-mg/kg/dose level is considered below the severely toxic dose level in monkeys for the study. This represents a > 300-fold safety factor over the proposed starting dose of 0.3 mg/kg. The highest dose of 100 mg/kg was associated with mean C_{max} and AUC_{τ} (τ=168 hours) values of 3,266 µg/mL and 252,787 h*µg/mL, respectively, for combined sexes on Day 85 of the dosing phase.

In addition to in vivo toxicology studies, a GLP-compliant tissue cross reactivity study has been performed to compare the binding of FPA144 to a panel of 36 tissues from rat, cynomolgus monkey, and human. In general, the binding pattern of FPA144 was similar among the 3 species and agreed with literature reports on the expression of FGFR2b being epithelial-based.

Additional details of the nonclinical program for FPA144 are provided in the Investigator's Brochure (IB), which contains comprehensive information on the investigational product.

1.3.5 Clinical Experience with FPA144

Please refer to the bemarituzumab Investigator's Brochure for updated results of the clinical experience with FPA144.

FPA144 underwent evaluation in 2 Phase 1 dose escalation studies. The first Phase 1, first-in-human study, FPA144-001, entitled "A Phase 1 Open-Label, Dose-Finding Study Evaluating Safety and Pharmacokinetics of FPA144 in Patients with Advanced Solid Tumors" started in December 2014 and was conducted in the US, South Korea, and Taiwan. This open-label study assessed the safety, pharmacokinetics (PK), and preliminary efficacy of FPA144 monotherapy in patients with solid tumors. The study was comprised of 3 parts: a dose escalation portion in unselected solid tumor patients (Part 1A), a dose escalation portion in GC patients (Part 1B), and a dose expansion portion for patients with FGFR2b-selected tumors including FGFR2b-selected GC, and FGFR2b-selected bladder cancer (Part 2).

Two parallel dose escalations were performed: Part 1A enrolled patients with solid tumors (19 patients), and Part 1B enrolled patients with GC (8 patients) to evaluate early evidence of efficacy and PKs to support the RD for the dose expansion cohort. FPA144 was well tolerated in doses up to 15 mg/kg administered every 2 weeks (Q2W) in patients with advanced solid tumors. There were no dose-limiting toxicities (DLTs) observed during dose-escalation and a maximum

tolerated dose (MTD) was not reached. Based on an assessment of safety, tolerability, and PK, an RD of 15 mg/kg administered Q2W was selected.

As of 20 March 2017, a total of 64 patients across the dose escalation (Parts 1A and 1B: 27 patients) and the dose expansion (Part 2: 37 patients) enrolled in the study and 60 received at least 1 dose of FPA144. Of these 64 patients, 41 patients had GC and 21 of those were identified as having GC with strong FGFR2b overexpression (or FGFR2b$^+$ high, defined as IHC 3+ intensity in ≥ 10% of tumor cells). Of those 21 patients, 6 patients were enrolled in the Part 1B dose escalation and 15 patients in the Part 2 dose expansion (Cohort A). In addition, 4 GC patients with low FGFR2b overexpression (or FGFR2b$^+$ low, defined as IHC 2+ intensity in < 10% of tumor cells or any immunohistochemistry (IHC) 1+ staining) have been enrolled in Cohort E and 10 GC patients (IHC 0–2) have been enrolled in Cohort C in Part 2 (dose expansion).

Safety and tolerability of FPA144 is supported by a total of 64 patients from the Phase 1 study (FPA144-001) who have received at least 1 dose of FPA144.

Safety data from 64 patients enrolled in FPA144-001 including 37 patients treated at 15 mg/kg administered Q2W (expansion dose) are described here. Adverse events (AEs) have been reported in 58 of 64 patients (90.6%). Thirty-two of the 64 patients reported an AE that was deemed by the investigator to be drug related. Of the drug-related AEs, none were Grade 4 or 5. There were 6 Grade 3 events: an infusion reaction in 1 patient, an aspartate aminotransferase (AST) elevation and alkaline phosphatase increase in 1 patient, nausea in 2 patients and a transient decrease in neutrophil count (which resolved without dose interruption or modification) in 1 patient. Three patients discontinued treatment due to an AE. One for E. coli sepsis, a second for cancer pain, both considered unrelated to study treatment and a third patient (post data cut) with limbic stem cell deficiency after approximately 14 months of therapy, considered related to study treatment also discontinued treatment. All other patients discontinued treatment as a result of disease progression. Treatment-related serious adverse events (SAEs) were reported in 3 patients: 1 patient (15 mg/kg) with the Grade 3 infusion reaction, 1 patient (15 mg/kg) with the Grade 2 corneal ulcer, and 1 patient (10 mg/kg) with the Grade 2 limbic stem cell deficiency (post data cut). In all 3 patients with treatment-related SAEs, the events resolved. The patient with the infusion reaction resumed drug administration after premedication and a reduced dose. The patient with the Grade 2 corneal ulcer temporarily interrupted FPA144 and received ophthalmic drugs with resolution of the event. The patient with the Grade 2 limbic stem cell deficiency discontinued study treatment and her symptoms and signs resolved after approximately 3 months.

Additional development of FPA144 for the treatment of GC includes an ex-US, non-US investigational new drug (IND) study, FPA144-002, entitled “A Phase 1 Open-Label, Dose-Finding Study of FPA144 in Japanese Patients with Advanced Gastric or Gastroesophageal Cancer.” This dose escalation study was designed to assess the PK and safety of single agent

FPA144 and identified the RD for single agent FPA144 in Japanese patients. No DLTs were reported in the FPA144-004 study.

Please reference the bemarituzumab investigator's brochure for an updated review of the ocular events that have occurred in clinical studies with FPA144. As discussed in Section 1.3.4, preclinical animal toxicity studies support a need for comprehensive ophthalmologic examinations. The comprehensive ophthalmologic examinations in the Phase 1 study (FPA144-001) includedd fundoscopic and slit lamp examination, ocular coherence tomography (OCT), visual acuity, and review of ocular and visual symptoms at screening, prior to the third dose, and at the end of treatment (EOT) visit. Slit lamp examinations (with completion of fluorescein staining score form), were conducted at regular intervals for all patients.

Twenty-three of 79 (29%) patients treated with FPA144 monotherapy in study FPA144-001 reported ocular adverse events. The most common ocular events ($\geq$ 5%) were dry eye (14 patients [18%]) and increased lacrimation (5 patients [6%]). Among these patients, 3 events, a Grade 2 ulcerative keratitis, a Grade 2 limbic stem cell deficiency, and a Grade 2 corneal dystrophy, required treatment and follow-up from an ophthalmologist.

In the phase 1 part of study FPA144-004, two events of punctate keratitis and one each of corneal abrasion, corneal disorder and limbal stem cell deficiency were reported. As of October 29, 2019, 18 of 134 (13%) patients treated in the phase 2 part of the FPA144-004 study had reported ocular adverse events involving the cornea or retina. Eight patients experienced keratitis (grade 1 (1); grade 2 (5), grade 3 (2)), three patients experienced punctate keratitis (grade 1; grade 2; grade 3 (1 each)), two experienced limbal stem cell deficiency (grade 3), two patients experienced retinal hemorrhage (grade 1), two patients experienced ulcerative keratitis (grade 2 (1); grade 3 (1)),one patient each experienced corneal erosion (grade 1), corneal infiltrate (grade 1) and xerophthalmia (grade 2). The retinal hemorrhages were asymptomatic incidental findings during comprehensive ophthalmologic evaluation and did not preclude ongoing treatment with FPA144/placebo. The study drug/placebo was withdrawn in eight patients (2 patients with keratitis, 1 patient each with corneal infiltrates, corneal ulcer, eye disorder, dry eye, ulcerative keratitis, xerophthalmia) and was delayed in four patients (2 cases of keratitis, and one patient each with superficial punctate lentitis both eyes and punctate epithelial erosion).

Evidence of early efficacy in the FGFR2b^{+} high gastric and GEJ cancer patient population is supported by a confirmed response rate (using Response Evaluation Criteria in Solid Tumors version 1.1 [RECIST v1.1]) of 19.0% [5.4-41.9%]) in 21 patients in the target patient population with a duration of response (DOR) of 15.4 weeks (95% confidence interval [CI] 9.1, 19.1 weeks) and a median PFS of 11 weeks (95% CI 5.7, 20.6 weeks).

FPA144 serum concentration was measured by a validated enzyme linked immunosorbent assay (ELISA) (refer to the FPA144 IB). FPA144 demonstrated nonlinear clearance (CL) from 0.3 mg/kg to 1 mg/kg and close to linear CL from 1 mg/kg to 15 mg/kg in patients with solid

tumors including GCs tested in Parts 1A and 1B, suggesting target-mediated CL. The estimated half-life ($t_{1/2}$) ranged from 6.01 to 11.7 days across cohorts, which supports every 2-week dosing. As derived from the mouse efficacy study using the OCUM2 *FGFR2*-amplified GC xenograft model, 60 µg/ml was selected as target trough serum concentration at steady state ($C_{trough\ ss}$). Based on the PK data in combination with safety data and evidence of efficacious activity from Part 1 of the clinical study, the 15 mg/kg Q2W dose was selected to test in the Part 2 expansion cohorts as this dose level was expected to achieve target $C_{trough\ ss}$ of ≥ 60 µg/mL. Limited PK data in Part 2 from a total of 18 GC patients dosed at 15 mg/kg Q2W, including 8 patients with high FGFR2b overexpression (Cohort A) and 10 patients without FGFR2b overexpression defined as IHC 0 (Cohort C), suggested that a dose of 15 mg/kg Q2W will achieve $C_{trough\ ss}$ target of ≥ 60 µg/mL (refer to the FPA144 IB) in approximately 70% of patients at Day 28. PK modeling suggests that the addition of a single dose of 7.5 mg/kg administered on Day 8 of the first cycle allows at least 90% of patients to achieve the target trough of ≥ 60 ug/ml at Day 15, without significantly increasing the C_{max}.

1.4 Rationale for mFOLFOX6

Chemotherapy for advanced GC prolongs survival and improves symptoms (Wagner 2006, Al-Batran 2008, Kang 2009, Okines 2009, Waddell 2014). The combination of a platinum agent with a fluoropyrimidine has become a frequently used combination (Kang 2009) and in a recent meta-analysis has been identified as superior to single agent treatment and best supportive care (Wagner 2006). Although there is no single standard globally accepted first line reference chemotherapeutic regimen for advanced GC, the combination of a fluoropyrimidine (5-FU, capecitabine or S1) and a platinum agent (cisplatin or oxaliplatin) is an accepted standard of care in both Western and Asian countries (Wagner 2006, Al-Batran 2008, Keam 2008, Kang 2009, Okines 2009).

The antitumor treatment effect of fluorouracil derivatives (5FU, capecitabine) are believed to result from inhibiting the enzyme thymidylate synthase (TS). (Ulrich 2000, Pullarkat 2001, Chen 2003). Leucovorin, also known as folinic acid, causes a biochemical modulation of 5-FU enhances the treatment effect of 5-FU in patients with GC (Kim 2003).

Oxaliplatin is a cisplatin analog that functions through its ability to form DACH-platinum adducts and block deoxyribonucleic acid (DNA) replications. Oxaliplatin exhibits additive or synergistic properties when combined with 5-FU and has proven to be effective even when treating 5-FU resistant cell lines or cell lines resistant to cisplatin (Kim 2003, Keam 2008).

In a randomized Phase 3 trial comparing 5-FU/LV/oxaliplatin with 5-FU/LV/cisplatin (FLP) in the treatment of 220 patients with GC reported a statistically insignificant improved time-to-progression; however, the oxaliplatin based regimen was associated with meaningful reductions in Grade 3–4 AEs, including anemia, nausea, vomiting and renal toxicity, but with more neuropathy (Al-Batran 2008). Subsequent studies have supported the safety and efficacy of mFOLFOX6 as the first line of treatment for advanced GC (Keam 2008).

Accordingly, mFOLFOX6 is used as standard therapy in advanced/metastatic GC patients in the US, Europe, and Asia.

1.5 Rationale for Combination Therapy: FPA144 and mFOLFOX6

Since advanced GC is not cured with the currently available chemotherapy regimens, there is a continued need to provide improvement to available treatments. The FGFR pathway has been shown to play an important role in the transformation and proliferation of tumor cells, and inhibiting FGF ligand-stimulated FGFR2b phosphorylation and cell proliferation has been shown to reduce tumor growth in both FGFR2b overexpressing gastric and breast cell lines and xenograft models.

FPA144 as monotherapy has demonstrated objective tumor responses in preclinical studies, blocking ligand binding and downstream signaling, decreasing expression of the FGFR2b driver protein and enhancing ADCC. Preliminary data from the first-in-human study of FPA144 indicate that FPA144 monotherapy has activity in patients with heavily pretreated FGFR2b-selected GC. The safety profile has been tolerable, with no DLTs encountered to date.

The combination of FPA144 and paclitaxel demonstrated enhanced activity in both the OCUM-2M and HSC-39 xenograft models of GC compared to monotherapy of either agent at the doses tested (Gemo 2014). Using a more aggressive chemotherapy regimen in these same models, such as cisplatin and 5-FU or oxaliplatin and 5-FU chemotherapy, provided near complete growth suppression in the HSC-39 model and therefore no additional benefit was observed with the addition of FPA144. Alternatively, the OCUM-2M *FGFR2*-amplified model, demonstrated near complete tumor suppression with FPA144 at the doses tested, and therefore no additional benefit was observed in combination with cisplatin + 5-FU or oxaliplatin + 5-FU chemotherapy. Importantly, the addition of FPA144 to any of these chemotherapy regimens did not increase the toxicity associated with chemotherapy as measured by weight loss in mice.

Advanced stage GC has been demonstrated to be heterogeneous, and the development of additional heterogeneity is hypothesized to be induced by standard front line chemotherapy (Smyth 2016). Specifically, GC metastases are more likely to overexpress FGFR2b compared to the primary tumor, suggesting that emergence of an FGFR2b overexpressing tumor is a later stage event (Ahn 2016), and may even be induced by prior systemic chemotherapy. Sequential biopsies and circulating tumor DNA (ctDNA) testing have demonstrated that subclones of tumors with different phenotype emerge after targeted therapy treatment (Smyth 2016, Catenacci 2017). The hypothesis that chemotherapy and FPA144 target different subclones of GC is supported by the ability of FPA144 or mFOLFOX6 to drive complete growth suppression of GC tumors in distinct xenograft models (Gemo 2014).

Further, the hypothesis that adding a targeted biologic agent to standard chemotherapy in GC may be beneficial in selected patients is further supported by the demonstration of the clinical benefit (PFS and OS) of adding trastuzumab to chemotherapy in patients with GC whose tumors

overexpress HER2 (Bang 2010) and the addition of ramucirumab to paclitaxel in patients who progressed after front-line therapy (Wilke 2014).

1.6 FPA144 and mFOLFOX6 Starting Dose Justification

In study FPA144-001, no DLTs were reported at doses tested from 0.3 mg/kg to 15 mg/kg administered Q2W and no MTD of FPA144 was identified. Therefore, the RD for expansion was based on the observation of clinical efficacy and tolerability in the human FPA144-001 Phase 1 study, combined with preclinical data from the OCUM2 *FGFR2*-amplified GC xenograft mouse model, which identified 60 µg/mL as the target trough serum concentration to achieve maximum efficacy. Supporting the hypothesis that ≥ 60 µg/mL should be the target minimum trough serum concentration (C_{trough}), all patients who demonstrated a partial response in the ongoing FPA144-001 study achieved the target $C_{trough\ ss}$ of ≥ 60 µg/mL. Patients receiving a lower dose of 10 mg/kg Q2W also achieved a target trough of ≥60 µg/mL, but not until steady state approximately 3 months after the initiation of FPA144. At the earlier time point of 28 days, only 3 of 6 patients dosed at the 10 mg/kg dose achieved the target C_{trough} of ≥ 60 µg/mL compared to 21 of 29 patients dosed at 15 mg/kg Q2W based on data as of 20 March 2017.

To safely minimize the time needed to reach the target FPA144 target trough concentration while increasing the potential for earlier efficacy in this rapidly progressing cancer, a dose of 7.5 mg/kg FPA144 will be administered on Day 8 of Cycle 1 for patients enrolled in Cohort 2. PK modeling suggests that the schedule of 15 mg/kg Q2W with 1 dose of 7.5 mg/kg on Day 8 (Cycle 1 only) will allow at least 90% of patients to achieve the biologically active target trough concentration by Day 15 instead of by Day 28. Achieving the target trough concentration earlier is clinically important in this patient population with rapidly progressive disease.

No drug-drug interactions are expected between FPA144 and mFOLFOX6 based on known mechanisms of CL. However, based on FGFR2b expression in tissues of epithelial origin (eg, stomach and skin), and the AEs reported in the ongoing FPA144-001 study, FGFR2 inhibition could increase the rate of mucositis, nausea, vomiting and diarrhea observed with mFOLFOX6 alone.

The Phase 1 portion of this study will initiate FPA144 combined with mFOLFOX6 a dose of 6 mg/kg administered Q2W. Assuming the dose of 6 mg/kg is demonstrated to be safe, tolerable, and without clinical or pharmacologic evidence of a drug interaction, the second dose to be tested will be 15 mg/kg Q2W with 1 dose of 7.5 mg/kg on Day 8 (Cycle 1 only). If Cohort 1 (6 mg/kg Q2W) clears the 28 day DLT period, but ≥ 2 DLTs are observed in Cohort 2 (15 mg/kg Q2W with 1 dose of 7.5 mg/kg on Day 8 of Cycle 1 only), based on observed safety, tolerability and PK, a dose level between Cohorts 1 and 2 (this new cohort will be Cohort 1a, proposed to be 15 mg/kg Q2W) may be evaluated in a rolling 6 design. If ≥ 2 DLTs are observed in Cohort 1a, then the Cohort Review Committee (CRC) may decide to evaluate a dose between the dose levels tested in Cohort 1a and Cohort 1 (this new cohort will be Cohort 1b) to

achieve tolerability with optimal target exposure. At least 6 patients will be evaluated for safety, tolerability and PK at the final RD prior to initiating the Phase 2 portion of the study.

1.7 Risk-Benefit Assessment of FPA144 and mFOLFOX6

This overview is not intended to replace the complete information presented in the FPA144 IB. Please consult the IB for more detailed information.

GC is a highly lethal disease, the treatment of which depends significantly on the stage of the disease. Intensive multimodal therapy for locoregional disease fails to cure most patients and standard chemotherapy for metastatic disease provides only short-term benefits. First-line chemotherapy used in metastatic or recurrent disease generally consists of a fluoropyrimidine (5-FU, S1 or capecitabine) with a platinum agent (usually oxaliplatin or cisplatin) and the mOS is 9 to 11 months with a median PFS of 5 to 7.4 months (Kang 2009). Patients with GC and *FGFR2* gene amplification (Seo 2016) or FGFR2b overexpression (Ahn 2016) have a worse prognosis (Hattori 1996, Gemo 2014) thus warranting evaluation of a new targeted agent against FGFR2+ GC .

mFOLFOX6 chemotherapy is associated with myelosuppression, most frequently Grade 3-4 neutropenia reported in about 40–50% of patients, peripheral neuropathy (> Grade 2) reported in about 70% of patients, diarrhea (> Grade 2) reported in about 25% of patients, and nausea/vomiting reported in about 3% of patients each (Tournigand 2004, van Hazel 2016). Vomiting and mucositis were not observed in FPA144 toxicology studies, however, 20.4% of patients in the ongoing FPA144-001 study with monotherapy FPA144 reported nausea and/or vomiting, with 3.1% reporting Grade 3 (no Grade 4 or higher was reported). Although no mucositis or diarrhea has been reported (n=64), based on mechanism of action, inhibition of FGFR2 may increase this risk. In patients with underlying metastatic GC receiving mFOLFOX6 chemotherapy, an increase in the incidence of any of these GI symptoms could be clinically meaningful.

The protocol includes standard anti-emetic therapy, close clinical monitoring and drug modifications and discontinuation. Additionally, an independent data monitoring committee (DMC) will monitor safety and toxicity on the study (refer to Section 8.10.2).

The individual agents used in the FOLFOX regimen (5-FU, oxaliplatin and leucovorin) are also associated with risks. Refer to local prescribing information for complete details.

The risks associated with fluorouracil include: myelosuppression, diarrhea, nausea and vomiting, mucositis, anorexia, palmar-plantar erythrodysthesia, alopecia, cardiac toxicity, neurotoxicity and hyperammonemic encephalopathy. Due to a potential drug-drug interaction between 5-FU and warfarin, there is a risk of an elevated international normalized ratio (INR) for patients on warfarin who also receive 5-FU (Teva Pharmaceuticals USA Inc 2016).

The risks associated with oxaliplatin include allergic reactions, neuropathy, pulmonary toxicity, hepatotoxicity, cardiovascular toxicity, rhabdomyolysis, nausea/vomiting, diarrhea, mucositis, myelosuppression, fatigue.

As of November 2017, no drug has been approved in the US, EU, or Asia specifically for the subset of patients with FGFR2-selected GC. Based on the emerging data from the Phase 1 FPA144-001 trial, FPA144 may provide a meaningful clinical benefit with an acceptable tolerability profile in heavily pretreated patients with GC whose tumors overexpressed FGFR2. FGF signaling pathways appear to be a valid target for clinical investigation in human cancer based on preclinical models as well as ongoing clinical trials with other molecules that broadly target FGF signaling (Taiho Oncology Inc 2015, Hall 2016, GlaxoSmithKline 2017).

As detailed in Section 1.5, the addition of FPA144 to current standard of care first-line treatment for advanced GC with mFOLFOX6 is anticipated to improve PFS compared to chemotherapy alone. Preliminary data from the first-in-human study of FPA144 indicate that FPA144 monotherapy has activity in patients with FGFR2b-selected gastric and bladder cancer. The safety profile has been tolerable, with no DLTs reported.

In addition to the study design (dose escalation) and eligibility criteria that exclude patients with significant organ dysfunction, the following precautions will be taken in this study:

- Based on non-clinical toxicology, FPA144 has an expected on-target effect leading to corneal thinning which may increase the risk of developing a corneal ulcer or a secondary corneal infection. Based on the aggregate clinical data, keratitis has been identified as an adverse drug reaction to bemarituzumab treatment. Accordingly, patients who at baseline have a history of corneal disease such as keratitis or corneal transplant will be excluded. Patients found to have ocular abnormalities involving the cornea during screening will also be excluded, including corneal defects, corneal ulcerations, keratoconus, or other abnormalities that can pose an increased risk of developing a corneal ulcer. Ophthalmology examinations will be performed at baseline and at regular protocol required intervals during the study to monitor potential ocular effects (described in Section 6.6.2).
- Based on non-clinical data, FPA144 may have on-target effects on the epithelium of the oropharynx, although redundancy in the FGF pathway in these organs may limit and the toxicity beyond what is anticipated from mFOLFX6 alone. To date, 1 event of Grade 1 dry mouth has been reported in a patient during Cycle 6 of FPA144 given at 3 mg/kg. Fluoropyrimidines have a known toxicity of mucositis (Teva Parenteral Medicines Inc 2016). Patients will undergo physical examination approximately every 2 weeks, including examination of the oropharynx.

- Patients will be closely monitored for infusion-related reactions which are known potential toxicities of both oxaliplatin and FPA144. The FPA144 infusion rate may be reduced at the investigator's discretion based on occurrence of infusion-related reactions, such as changes in vital signs, nausea, vomiting, or other constitutional symptoms or allergic reactions occurring during infusion or up to 2 hours after cessation of the infusion.
  - Routine premedication is not generally administered for the initial FPA144 dose; patients who develop infusion-related AEs may be premedicated prior to subsequent infusions of FPA144 at the discretion of the investigator. Pre-medication for mFOLFOX6 should be at the discretion of the investigator, administered according to the institution's standard practice, and captured on the patient's electronic case report form (eCRF).
  - Epinephrine for subcutaneous injection, diphenhydramine (or equivalent) for IV injection, and any other medications and resuscitation equipment for emergency management of anaphylactic reactions must be available in the room where the infusions are being performed.

1.8 Rationale for Phase 2 Pre-Screening

FPA144 is an antibody designed to recognize the FGFR2 receptor when expressed on gastric tumors. The current hypothesis is that the presence of FGFR2 will be an important predictor of how patients with FGFR2-selected GC will respond to treatment with FPA144. This is based on the preclinical observation that only tumors that overexpressed FGFR2b responded to FPA144 treatment in xenograft studies, as well as early results from the ongoing first-in-human study demonstrating objective responses in patients with FGFR2b overexpression.

Eligibility for enrollment in the Phase 2 portion is based on FGFR2b overexpression **and/or** *FGFR2* gene amplification as determined by a centrally performed, validated IHC or ctDNA blood assay. Patients are required to provide both a tissue sample and a blood sample to test for FGFR2 and patients unable to provide both samples will not be eligible for this trial. Patients who are positive for FGFR2b overexpression and/or *FGFR2* gene amplification are referred to in this protocol as FGFR2-selected. Positivity based on only 1 assay is adequate to meet eligibility requirements (eg, positive by ctDNA blood assay, but negative by IHC) (refer to Section 4.2).

Pre-screening allows for collection of blood and tissue to determine FGFR2 positivity using ctDNA and IHC assays. Once a patient is confirmed to have a positive result based on central testing, the screening period initiates, allowing for evaluation of all other eligibility criteria (refer to Section 4.2).

Eligible patients for the Phase 2 portion must be naïve to prior chemotherapy for metastatic or unresectable disease, with the exception that patients may have received prior adjuvant or neo-adjuvant therapy (chemotherapy and/or chemoradiation) greater than 6 months prior to enrollment.

Since the IHC and ctDNA blood results may require approximately 2 weeks to complete, patients eligible for entering Phase 2 of this study are permitted to receive 1 dose of mFOLFOX6 during this interim time period (pre-screening period) at the discretion of the investigator. This 1 dose of chemotherapy is not a requirement of the study and is not considered part of this clinical study.

1.9 Rationale for Tumor Tissue and Blood Assessments

Patients who do not demonstrate either FGFR2b overexpression using IHC or *FGFR2* gene amplification using a ctDNA blood assay will not be eligible for enrollment. Positivity based on either 1 or both assays is adequate to meet the eligibility requirements (eg, positive by ctDNA blood assay, but negative by IHC). The blood test will reveal DNA amplification of *FGFR2*, while the IHC test will show the extent of protein expression. Five Prime has developed an anti-FGFR2 antibody for nonclinical use, whose sensitivity and specificity to detect FGFR2 by IHC has been optimized (Deshpande 2014).

In studies evaluating GC samples, *FGFR2* gene amplification has been uniformly associated with significant FGFR2b surface expression, as detected by IHC (Gemo 2014). The antitumor effect of FPA144 that was observed in preclinical testing was predicated upon the overexpression of FGFR2b in the tumor cell lines. Patients without overexpression of FGFR2 are unlikely to see a significant benefit from treatment with FPA144 in combination with mFOLFOX6.

The selection of patients with FGFR2-positive tumors for treatment with FPA144 is supported by data from the ongoing Phase 1, first-in-human study of FPA144-001. Confirmed responses have only been reported in patients having tumors with FGFR2b overexpression, validating the strategy of selecting these patients for treatment with FPA144 based on FGFR2 status. Additionally, since GC is heterogeneous, the use of ctDNA has been incorporated to identify tumors that shed FGFR2 into the blood stream, but whose tumors do not overexpress FGFR2b (due to tissue sampling error). Allowing patients to enroll who either test positive for FGFR2b by tissue or positive for FGFR2 by blood will maximize the inclusion of patients with FGFR2 amplified tumors in the trial.

2.0 STUDY OBJECTIVES AND ENDPOINTS

2.1 Phase 1 Objectives

2.1.1 Primary

To determine the RD of FPA144 combined with a fixed dose of mFOLFOX6 (hereinafter referred to as FPA144 + mFOLFOX6) in patients with advanced GI tumors

2.1.2 Secondary

- To evaluate the safety and tolerability of FPA144 + mFOLFOX6 in patients with GI tumors
- To characterize the PK profile of FPA144 + mFOLFOX6 in patients with GI tumors
- To characterize the immunogenicity of FPA144

2.1.3 Exploratory

To characterize the pharmacodynamic profile of FPA144 + mFOLFOX6 in patients with GI tumors

2.2 Phase 2 Objectives

2.2.1 Primary

To compare investigator-assessed PFS in patients with FGFR2-selected GC treated with FPA144 + mFOLFOX6 to those treated with placebo combined with mFOLFOX6 (hereafter referred to as placebo + mFOLFOX6)

2.2.2 Secondary

To compare the following in patients with FGFR2-selected GC treated with FPA144 + mFOLFOX6 to those treated with placebo + mFOLFOX6:

- Overall survival (OS)
- Investigator-assessed ORR
- Safety and tolerability

2.2.3 Exploratory

To compare the following in patients with FGFR2-selected GC treated with FPA144 + mFOLFOX6 to those treated with placebo + mFOLFOX6:

- DOR
- Patient-reported outcomes (PROs) and quality of life (QOL) outcomes until investigator-assessed disease progression

- To explore the association between FGFR2 status (in tumor tissue and/or blood) with clinical outcome
- To explore the concordance between FGFR2b overexpression in tumor tissue and *FGFR2* gene amplification in blood

To characterize the following:

- PK profile of FPA144 + mFOLFOX6 in patients with FGFR2-selected GC
- Immunogenicty of FPA144

2.3 Phase 1 Endpoints

2.3.1 Primary

- The incidence of Grade 2 or higher AEs assessed as related to FPA144 by the investigator and the incidence of clinical laboratory abnormalities defined as DLTs

2.3.2 Secondary

- The incidence of AEs, clinical laboratory abnormalities, corneal and retinal findings, and electrocardiogram (ECG) abnormalities
- PK parameters of FPA144, such AUC, C_{max}, C_{trough}, CL, $t_{1/2}$, volume of distribution, the time to achieve steady state, dose-linearity, and accumulation ratio
- Incidence of treatment-emergent anti-FPA144 antibody response

2.3.3 Exploratory

Pharmacodynamic parameters, including exploratory pharmacodynamic biomarker analyses of the FGFR pathway in blood

2.4 Phase 2 Endpoints

2.4.1 Primary

PFS, defined as time from randomization until the date of disease progression based on investigator assessment per RECIST v1.1 or death from any cause, whichever comes first

2.4.2 Secondary

- OS, defined as time from randomization until death from any cause
- ORR, defined as the proportion of patients with partial or complete response in all enrolled patients based on investigator assessment of tumor lesions per RECIST v1.1
- Incidence of AEs, clinical laboratory abnormalities, corneal and retinal findings, and ECG abnormalities

2.4.3 Exploratory

- DOR limited to patients who are responders to treatment, as determined by the investigator per RECIST v1.1, and defined as the time of first response to progression or death from any cause, whichever comes first
- Change from baseline in functional outcomes as measured by EuroQOL-5D-5L (EQ-5D-5L) and the European Organization for Research and Treatment of Cancer Quality of Life Questionnaire Version 3.0 (EORTC QLQ-C30)
- The correlation between identified FGFR2 status in tumor tissue and/or ctDNA blood assay, as determined by IHC and blood-based molecular diagnostic assay, with OS, PFS, and objective response per RECIST v1.1
- The correlation between identified FGFR2b overexpression in tumor tissue by IHC and *FGFR2* gene amplification as determined by ctDNA blood assay
- PK parameters, C_{max} and C_{trough} of FPA144 in combination with mFOLFOX6
- Incidence of treatment-emergent anti-FPA144 antibody response

3.0 STUDY DESIGN

3.1 Study Overview

The Phase 2 portion of this study was initially designed as a Phase 3 with overall survival as the primary endpoint. The study design has been changed considering the findings of a higher proportion of FGFR2 positive tumors in patients in the front-line gastric cancer setting than expected (~ 30% vs the expected rate of 10%) and that the vast majority of tumors are positive via IHC analysis for FGFR2b overexpression rather than by ctDNA analysis of FGFR2 amplification. The randomized, double-blind study design will be maintained through the primary analysis of the Phase 2 study.

This is a double-blind, randomized, controlled, multicenter Phase 1/2 study to evaluate the safety, tolerability, efficacy, and PK of FPA144 + mFOLFOX6 versus placebo + mFOLFOX6. Patients may enroll into either Phase 1 or Phase 2 but may not enroll in both phases of the study.

This study includes a Phase 1 safety run-in portion and a Phase 2 portion. The Phase 1 safety run-in is an open-label dose-escalation of FPA144 + mFOLFOX6 in patients with GI tumors (not FGFR2 selected). Provision of tissue for retrospective FGFR2b overexpression testing by IHC, and blood for FGFR2 gene amplification by ctDNA, are not required for enrollment in Phase 1, but will be tested retrospectively if available (refer to Section 6.1).

The Phase 2 portion of the study (to follow the Phase 1 safety run-in) is a global, randomized, double-blind, controlled, study to evaluate the efficacy of FPA144 + mFOLFOX6 versus placebo + mFOLFOX6 in patients with FGFR2-selected GC. FGFR2b overexpression will be determined by prospective IHC analysis and/or a ctDNA blood assay demonstrating FGFR2 gene amplification. The duration of the study is expected to be approximately 43 months to complete; this includes Phase 1 (6 months for completion) and Phase 2 (13 months for accrual and 24 months of long term follow up (LTFU) from the last patient enrolled).

In Phase 1, the starting dose level of FPA144 is 6 mg/kg per dose. Subsequent dose escalations between cohorts in Phase 1 are described in Section 3.2.1. The dose of FPA144 for Phase 2 will be determined by evaluation of the data from Phase 1 of the study, as described in Sections 3.2.3 and 3.2.4.

Additional study design details are provided below for each study phase. The study schemas are provided in Section 3.4.

3.2 Phase 1

3.2.1 Dose Escalation

In Phase 1, dose cohorts are planned at proposed doses beginning at an FPA144 dose level of 6 mg/kg per dose Q2W, and enrollment will depend on safety and tolerability. Phase 1 includes a 3+3 and rolling 6 design until the RD of FPA144 to be administered in combination with mFOLFOX6 in Phase 2 is determined. A minimum of 2 dosing cohorts of FPA144 + mFOLFOX6 will be included in Phase 1 to determine the RD of FPA144 in combination with mFOLFOX6.

Patients enrolled in Phase 1 will be treated with escalating doses of FPA144 in combination with a fixed-dose chemotherapy regimen of mFOLFOX6 in 2-week cycles, as outlined in Table 1. Intra-patient dose escalation will not be permitted.

Additional details regarding dose escalation and the DLT period are provided in Section 3.2.3. Proposed dose levels are outlined in Table 1 and also in Section 5.1.2 and Section 5.2 of the protocol; guidance for dose modification is provided in Section 5.1.3.4.

Decisions on how to next proceed will be based on safety, tolerability, and PK data, and will be determined by the CRC.

Administration of FPA144 will be over approximately 30 minutes (± 10 minutes) Q2W ± 3 days on Day 1 of each 2-week cycle. FPA144 will be administered prior to mFOLFOX6 chemotherapy. Patients treated in Cohort 2 (only) will receive 1 additional dose of FPA144 on Day 8 of Cycle 1 (mFOLFOX6 will not be administered on this day).

Table 1: Phase 1 Proposed Dose Levels by Cohort

<table>
<tr><th>Cohort</th><th>FPA144</th><th>mFOLFOX6
(mFOLFOX6 will be administered at a fixed dose to all FPA144 dose cohorts)</th></tr>
<tr><td>Cohort 1</td><td>6 mg/kg Q2W beginning on C1D1</td><td rowspan="4">Q2W beginning on C1D1, at least 30 minutes after the end of infusion of FPA144:
• Oxaliplatin 85 mg/m^2 IV infusion over 120 minutes
• Leucovorin 400 mg/m^2 IV infusion over 120 minutes, can be administered concurrently with oxaliplatin if using a Y connector, (if not using a Y connector, administer drugs sequentially)
– If leucovorin is unavailable, 200 mg/m^2 levo-leucovorin may be used. Study treatment may be administered without either agent in the event that both are unavailable. Leucovorin (or levo-leucovorin) may be given at either the protocol-recommended doses or as deemed appropriate by the investigator in accordance with institutional standard of care
• Immediately after oxaliplatin and leucovorin, 5 FU 400 mg/m^2 bolus over approximately 5 minutes
• Immediately after the 5-FU bolus, 5-FU 2400 mg/m^2 as a continuous IV infusion over approximately 48 hours
For further details, refer to the manufacturer's prescribing information for the respective chemotherapies and the study Pharmacy Manual.</td></tr>
<tr><td>Cohort 2</td><td>15 mg/kg Q2W beginning on C1D1, plus 1 dose of 7.5 mg/kg on Day 8 (Cycle 1 only)</td></tr>
<tr><td>Cohort 1a</td><td>(if needed, eg, if the Cohort 2 dose level or schedule is not tolerated)
15 mg/kg Q2W beginning on C1D1</td></tr>
<tr><td>Cohort 1b</td><td>(if needed, eg, if the Cohort 2 and 1a dose levels or schedules are not tolerated)
Dose level lower than Cohort 1a, but higher than Cohort 1 to achieve tolerability with optimal target exposure</td></tr>
</table>

Abbreviations: C1D1 = Cycle 1 Day 1; IV = intravenous; Q2W = every 2 weeks.

Note: If leucovorin is unavailable, 200 mg/m2 levofolinic acid may be used. Study treatment may be administered without either agent in the event that both are unavailable. Folinic acid (or levofolinic acid) may be given at either the protocol-recommended doses or as deemed appropriate by the investigator in accordance with institutional standard of care.

3.2.2 Definition of Dose-Limiting Toxicity

DLTs are defined as any of the following events that occur during the first 28 days of treatment and are assessed by the CRC as related to FPA144. As applicable, events will be classified according to the National Cancer Institute (NCI) Common Terminology Criteria for Adverse Events (CTCAE) (Version 4.03).

- Absolute neutrophil count (ANC) $< 0.5 \times 10^9$/L > 5 days' duration or febrile neutropenia (ie, ANC $< 1.0 \times 10^9$/L with a single temperature of > 38.3°C, or fever ≥ 38 °C for more than 1 hour). Use of granulocyte-colony stimulating factor (G-CSF) is permitted in accordance with institutional standards
- Platelets $< 25 \times 10^9$/L
- Platelets $< 50 \times 10^9$/L with bleeding requiring medical intervention
- Platelets $< 50 \times 10^9$/L (> 3 days)

- Grade 4 anemia (ie, life-threatening consequences, urgent intervention indicated)
- Any Grade 2–3 ophthalmologic AE that does not resolve within 7 days
- Any Grade 4 ophthalmologic AE
- Any Grade 4 laboratory value
- Any Grade 3 laboratory values that are not of clinical significance according to investigator and Sponsor agreement that do not resolve within 72 hours
- AST/ alanine aminotransferase (ALT) ≥ 3 × upper limit of normal (ULN) and concurrent total bilirubin ≥ 2 × ULN not related to liver involvement with cancer
- Any non-hematological AE Grade 3 or greater (except nausea, vomiting, and diarrhea)
- Grade 3 nausea, vomiting, or diarrhea that does not resolve with supportive care in 72 hours
- Grade 4 nausea, vomiting, or diarrhea

3.2.3 Observation of Dose-Limiting Toxicity (the DLT Period)

Beginning on the first day (Cycle 1 Day 1 [C1D1]) of treatment with FPA144 and mFOLFOX6, each patient will be observed for 28 days (the DLT period) for safety, PK, and occurrence of DLTs. DLTs are defined in Section 3.2.2.

FPA144 dose escalation will occur as follows (refer to Table 2 for the dose escalation algorithm):

- Cohort 1 begins with a FPA144 dose of 6 mg/kg per dose in a 3+3 design.
- If Cohort 1 at 6 mg/kg (3+3 design) clears the 28-day DLT period, then Cohort 2 at 15 mg/kg Q2W with 1 dose of 7.5 mg/kg on Day 8 (Cycle 1 only) will be tested in a rolling 6 design and enroll 6 patients.
- Upon initiation of enrollment in Cohort 2, 6 patients will be enrolled to explore the safety, tolerability and PK at this dose level.
- If ≥ 2 DLTs are observed in Cohort 2, then a dose level between Cohorts 1 and 2 may be evaluated in a rolling 6 design (this cohort will be Cohort 1a 15 mg/kg Q2W).
- If ≥ 2 DLTs are observed in Cohort 1a, then a dose level lower than Cohort 1a, but higher than Cohort 1 may be evaluated in a rolling 6 design (this cohort will be Cohort 1b at a dose to be determined).

Dose escalation decisions will be agreed upon by the CRC, consisting of the Sponsor and investigators. Dose escalation decisions will be based on an assessment of DLTs, overall safety, and tolerability, and will be made after the last patient enrolled in each cohort has completed the 28-day DLT period (completion of 2 treatment cycles of FPA144 and mFOLFOX6). Review of

safety and PK parameters may inform decisions to add cohorts with alternative dose levels to reach an optimal target exposure.

No additional doses of FPA144 or more than 2 doses of mFOLFOX6 should be administered during the 28-day DLT period. The doses of FPA144 and mFOLFOX6 on Day 1 of Cycle 2 do not need to be synchronized. For example, if mFOLFOX6 is delayed due to an AE that is deemed related only to mFOLFOX6 and not to FPA144, FPA144 should be administered as described in Section 5.1 for Cycles 1 and 2 regardless of delays in the mFOLFOX6 dosing schedule.

DLTs are defined in detail in Section 3.2.2. The algorithm shown in Table 2 will be used for Phase 1 dose escalation decisions.

Table 2: Phase 1 Dose Escalation Algorithm

Number of Patients with DLTs	Action
0/3	Open next cohort
1/3	Enroll 3 more patients in same cohort
≥ 2/3	Stop enrollment. If Cohort 1, then the study will end.
1/6	Open next cohort
≥ 2/6	Stop enrollment at that level. If at Cohort 1, the study will end. If at Cohort 2 or Cohort 1a, then Cohort 1a or Cohort 1b will open respectively and 6 patients will be enrolled.

Abbreviations: DLT = dose-limiting toxicity.

Upon completion of the DLT period (starting with Cycle 3), patients may continue to receive FPA144 + mFOLFOX6. Additional doses may be administered Q2W (1 cycle) until disease progression, unacceptable toxicity, or until the patient meets any of the other protocol-specified withdrawal criteria (refer to Section 4.0). There is no maximum number of doses of FPA144. Ongoing administration of the mFOLFOX6 regimen beyond the DLT period will be according to local standard of care.

3.2.4 Determination of Maximum Tolerated Dose and Recommended Dose

The RD of FPA144 for Phase 2 will be identified by the CRC based on an evaluation of the overall safety, tolerability, and PK and will not exceed 15 mg/kg administered IV Q2W with 1 dose of 7.5 mg/kg on Day 8 of Cycle 1 only. In determining the RD, the CRC will consider toxicities observed during the DLT evaluation period, any toxicities observed beyond the DLT evaluation period, as well as dose reductions and discontinuations of mFOLFOX6 or FPA144 due to toxicities that do not meet the DLT criteria. Based on the totality of the data, the chosen RD of FPA144 will be a dose that is not anticipated to lead to a decrease in the dose intensity of mFOLFOX6 to be administered. The RD, therefore, may or may not be the same as the identified MTD. For example, if the MTD is not reached, or if data from subsequent cycles of

treatment from Phase 1 provide additional insight on the safety profile, then the RD may be a different, though not higher, dose than the MTD.

The MTD is defined as the maximum dose at which < 33% of patients experience a DLT during the DLT period. If a DLT is observed in 1 of 3 patients in Cohort 1, then 3 additional patients will be enrolled at that same dose level. Dose escalation may continue until 2 of 3 to 6 patients treated at a dose level experience a DLT (dose level not to exceed the highest dose level tolerated in Phase 1). The next lower dose will then be considered the MTD.

3.2.5 Phase 2 Dose of FPA144

There were no DLTs observed during the Phase 1 safety run-in and an MTD was not reached. Based on an assessment of overall safety, tolerability, and PK of FPA144 in combination with mFOLFOX6 by the CRC, the dose of 15 mg/kg Q2W with an additional dose of 7.5 mg/kg on Cycle 1 Day 8 will be used for the Phase 2 portion of the trial.

3.2.6 Patient Replacement

Any patient who does not receive the total number of doses of FPA144 defined by the cohort or 2 complete doses of mFOLFOX6 during the DLT period due to a reason that is not a DLT or an AE related to FPA144, will be considered unevaluable and the patient will be replaced. The replaced patient may continue on study at the investigator's discretion and after discussion with the Sponsor.

3.2.7 Number of Doses

There is no protocol-mandated maximum number of doses for FPA144 or mFOLFOX6. Ongoing administration of the mFOLFOX6 regimen beyond the DLT period will be according to Section 3.2 or 5.2 with dose adjustments as detailed in Section 5.2.3.1. Initiation of a new cycle of mFOLFOX6 following a dosing delay may be synchronized with administration of an FPA144 infusion but is not a study requirement. In the Phase 1 portion of the study, if FPA144 is permanently discontinued for any reason the patient will undergo an EOT visit approximately 28 days after the last dose of FPA144 or mFOLFOX6. For these patients, the end of FPA144 treatment is the end of study (EOS) and no further follow-up will be conducted.

If mFOLFOX6 is discontinued for any reason other than investigator-assessed disease progression, or any of the other protocol-specified withdrawal criteria, FPA144 may be continued as a single agent therapy at the investigator's discretion, and administered Q2W until investigator-assessed disease progression, unacceptable toxicity, or until the patient meets any of the other protocol-specified withdrawal criteria.

In the event a cycle of mFOLFOX6 is delayed beyond the next scheduled administration due to chemotherapy-related toxicity during the first 3 cycles of treatment, FPA144 should be administered on schedule (± 3 days). After the first 3 cycles, FPA144 may be delayed up to 7 days to be synchronized with administered mFOLFOX6.

3.3 Phase 2

Enrollment in Phase 2 will begin when an RD for FPA144 has been identified by the CRC which does not exceed the highest dose level evaluated and tolerated in Phase 1. Opening Phase2 for enrollment will be at the discretion of the Sponsor.

Provision of archival (or fresh biopsy if archival tissue is not available) for pre-screening FGFR2b overexpression testing by IHC is required (refer to Section 6.1); a blood sample is also required for pre-screening by ctDNA for FGFR2 amplification. FGFR2 positive status by 1 of these testing methods is required for enrollment in Phase 2. The results of the centralized FGFR2 testing may take approximately 2 weeks; patients are permitted to receive up to 1 dose of mFOLFOX6 during this interim time period (pre-screening) at the discretion of the investigator. This 1 dose of chemotherapy is not a requirement of the study and is not considered part of this clinical study.

In the Phase 2, a patient is considered enrolled when he/she is randomized. The enrollment date is the same as randomization date. Phase 2 patients must initiate the first administration of study treatment within 3 days of enrollment.

FPA144 will be prepared and administered as in Phase 1 (Section 5.1), and the dosing and schedule of FPA144 will be at the RD as determined in Phase 1.

3.3.1 Treatment Arms

Phase 2 will be randomized 1:1 to 1 of 2 treatment arms, as outlined in Table 3.

Table 3: Phase 2 Treatment Arms

<table>
<tr><td>Arm 1
FPA144 +
mFOLFOX6</td><td>FPA144
15 mg/kg Q2W with an additional dose of 7.5 mg/kg on Cycle 1 Day 8</td><td rowspan="2">mFOLFOX6 will be administered at a fixed dose to both Phase 2 treatment arms:
Q2W beginning on C1D1, at least 30 minutes after the end of infusion of FPA144:
• Oxaliplatin 85 mg/m^2 IV infusion over 120 minutes
• Leucovorin 400 mg/m^2 IV infusion over 120 minutes, can be administered concurrently with oxaliplatin if using a Y connector, (if not using a Y connector, administer drugs sequentially)
If leucovorin is unavailable, 200 mg/m^2 levo-leucovorin may be used. Study treatment may be administered without either agent in the event that both are unavailable. Leucovorin (or levo-leucovorin) may be given at either the protocol-recommended doses or as deemed appropriate by the investigator in accordance with institutional standard of care
• Immediately after oxaliplatin and leucovorin, 5 FU 400 mg/m^2 bolus over approximately 5 minutes
• Immediately after the 5-FU bolus, 5-FU 2400 mg/m^2 as a continuous IV infusion over approximately 48 hours
For further details, refer to the manufacturer's prescribing information for the respective chemotherapies and the study Pharmacy Manual.</td></tr>
<tr><td>Arm 2
Placebo +
mFOLFOX6</td><td>Placebo</td></tr>
</table>

Abbreviations: C1D1 = Cycle 1 Day 1; IV = intravenous; Q2W = every 2 weeks; RD = recommended dose.

Note: If leucovorin is unavailable, 200 mg/m2 levofolinic acid may be used. Study treatment may be administered without either agent in the event that both are unavailable. Folinic acid (or levofolinic acid) may be given at either the protocol-recommended doses or as deemed appropriate by the investigator in accordance with institutional standard of care.

Enrolled patients may continue treatment in 2-week cycles of mFOLFOX6 with or without FPA144 until investigator-assessed disease progression, unacceptable toxicity, or the patient meets any of the other protocol-specified withdrawal criteria. There is no protocol-mandated maximum number of doses for FPA144 or mFOLFOX6.

Discontinuation of any component of the study (mFOLFOX6, a component of mFOLFOX6, or FPA144) for any reason other than disease progression, does not necessarily mandate discontinuation of the other components. An exception is the permanent discontinuation of 5-FU for any reason, which requires concurrent discontinuation of oxaliplatin and leucovorin.

The first 3 cycles of FPA144 should be administered as scheduled (± 3 days) regardless of delays in mFOLFOX6 treatment. After the first 3 cycles, FPA144 may be delayed up to 7 days to be synchronized with administered mFOLFOX6. However, synchronization of FPA144 and mFOLFOX6 is not a protocol requirement. If after 7 days, the patient is still unable to receive mFOLFOX6, FPA144 should continue as monotherapy Q2W.

Patients who discontinue study treatment (FPA144 and/or mFOLFOX6) for reasons other than disease progression or withdrawal of consent will continue to undergo tumor assessments according to the protocol schedule until disease progression or the initiation of additional anticancer therapy, at which point they would undergo LTFU for survival.

3.4 Study Schemas

The study schema (Phase 1/2) is shown in Figure 4. Patient timelines are shown in both Figure 5 (Phase 1) and Figure 6 (Phase 2).

Figure 4: Phase 1/2 Study Schema

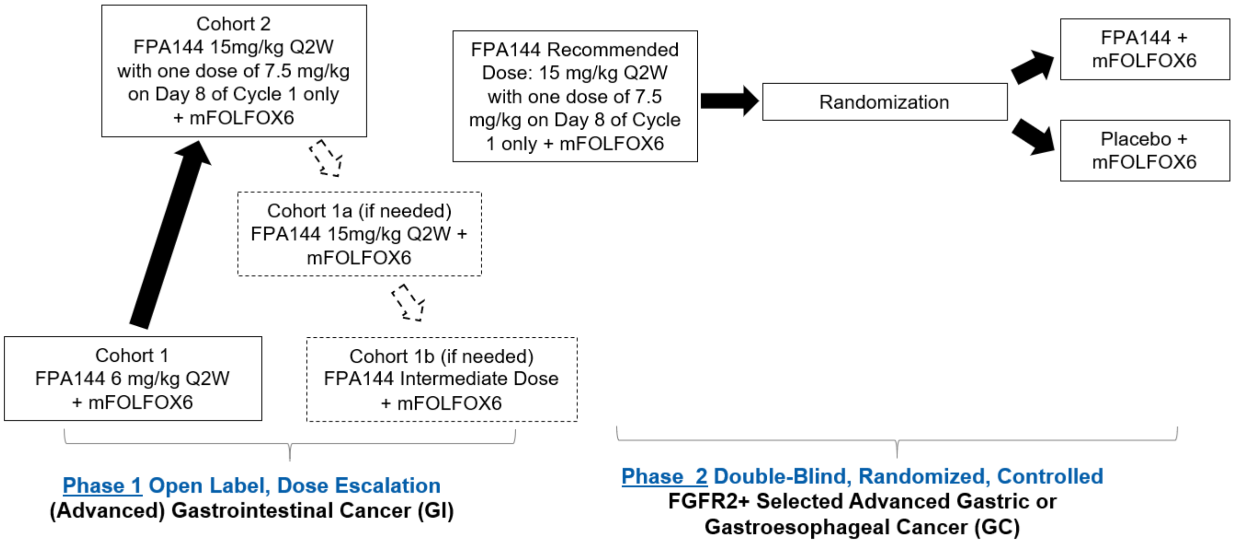

Abbreviations: Q2W = every 2 weeks.

Figure 5: Phase 1 Patient Timeline

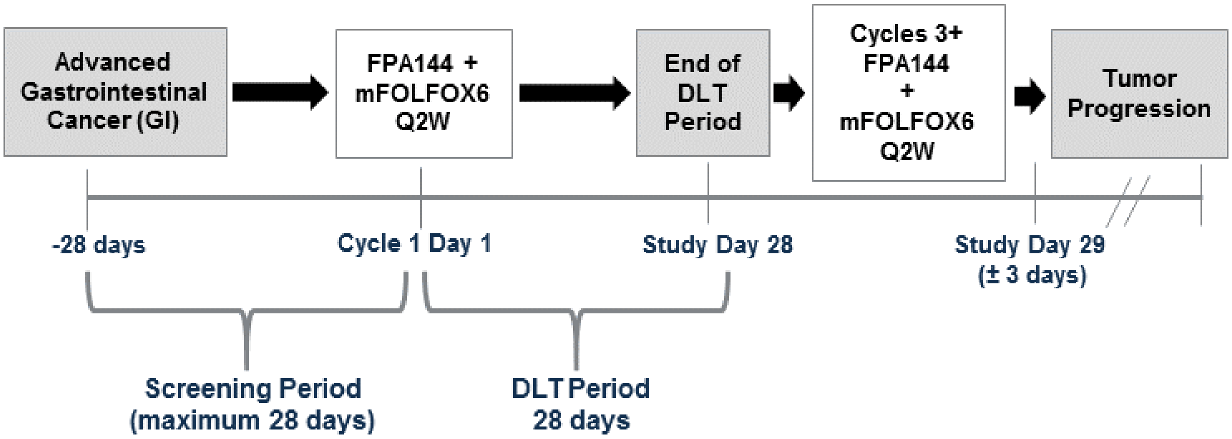

Abbreviations: DLT = dose-limiting toxicity; Q2W = every 2 weeks.

Figure 6: Phase 2 Patient Timeline

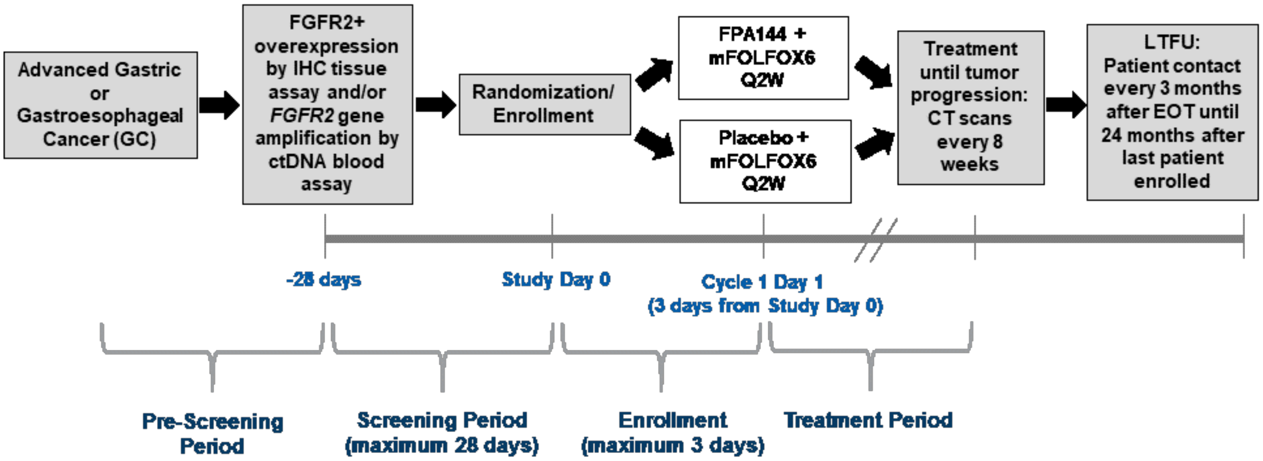

Abbreviations: CT = computed tomography; ctDNA = circulating tumor DNA; EOT = end of treatment; FGFR2 = fibroblast growth factor receptor 2; LTFU = long term follow up; Q2W = every 2 weeks.

3.5 Rationale for the Study Design

The aim of this study is to compare efficacy and safety of FPA144 versus placebo in combination with an accepted standard therapy, mFOLFOX6, in previously untreated patients with metastatic GC that is classified as both HER2 negative and FGFR2 positive. A placebo control will be administered with the chemotherapy to avoid any observational or other potential bias in the assessment of both efficacy and safety of the study treatment. This control group will be instrumental in assessing the relative benefit or risk of adding FPA144 to chemotherapy.

3.5.1 Rationale for Selection of Patients for FGFR2

Selecting patients for *FGFR2* gene amplification and overexpression targets patients that are most likely to obtain a clinical benefit from FPA144. It is hypothesized that the presence of FGFR2 will be an important predictor of how patients with FGFR2-selected gastric or gastroesophageal cancer will respond. This is based on the preclinical observation that only tumors that overexpressed FGFR2b responded to FPA144 treatment in xenograft studies, as well as early results from the ongoing first-in-human study (FPA144-001) demonstrating objective responses in patients with FGFR2b overexpression. To be eligible, patients must demonstrate either FGFR2b overexpression using IHC or *FGFR2* gene amplification using a ctDNA blood assay. Positivity based on either 1 or both assays is adequate to meet the eligibility requirements. The blood test will reveal DNA amplification of *FGFR2*, while the IHC test will show the extent of protein expression. In studies evaluating GC samples, *FGFR2* gene amplification has been uniformly associated with significant FGFR2b surface overexpression, as detected by IHC (Gemo 2014). Patients without overexpression of FGFR2 are unlikely to see a significant benefit from treatment with FPA144 and mFOLFOX6.

3.6 Rationale for Stratification

To balance the disease-related risk factors across the treatment arms, patients will be stratified at study entry. A permuted-block randomization scheme will be used to ensure an approximately equal sample size and a similar distribution of stratification factors for the two treatment arms. Patients will be stratified as outlined in Sections 3.6.1, 3.6.2, and 3.6.3.

3.6.1 Geographic Region

Although GC is a global disease, there is significant heterogeneity with respect to survival outcomes between Eastern and Western populations, with better OS reported in Asian populations and in particular Japanese patients (Ohtsu 2011, Davidson 2016). The cause of the different outcomes is unknown, but has been hypothesized to be driven by variation in initial staging, biology or subsequent treatment (Ohtsu 2006). Historically, large global trials have not included a significant proportion of patients from China, so it is unknown if the outcome is more similar to US and EU or the rest of Asia (Davidson 2016). As it is anticipated that approximately half the enrollment in this clinical trial will be from China, China will be a separate geographic region from the rest of Asia and accordingly, stratification will be by 4 geographic areas:

- Region 1: including US, Europe, and Australia
- Region 2: China
- Region 3: Rest of Asia including Japan, South Korea, Taiwan, and Thailand
- Region 4: Rest of World

3.6.2 Prior Therapy

Patients will be stratified based on history of prior chemotherapy administered for neo-adjuvant or adjuvant therapy. This stratification is included because this has been shown to be a prognostic factor associated with longer OS in some studies (Bang 2010, Ohtsu 2011, Sawaki 2011).

3.6.3 Administration of mFOLFOX6 Prior to Enrollment

Patients will be stratified based on whether they have received a single dose of mFOLFOX6 chemotherapy for advanced stage disease prior to enrollment. Although it is anticipated that a low proportion of patients will receive this single dose of mFOLFOX6 prior to enrollment, patients who receive a prior dose of chemotherapy are likely have characteristics that would be associated with a different prognosis than patients who do not receive this single dose of mFOLFOX6. Specifically, their extent of disease is likely to be greater or the rate of progression of the disease in these patients is likely to be greater than patients who can wait until results for FGFR2 testing is available.

4.0 STUDY ELIGIBILITY AND WITHDRAWAL CRITERIA

4.1 Planned Number of Patients and Study Centers

In the dose escalation Phase 1, at least 2 dose cohorts of FPA144 are anticipated using a 3+3 design for patients enrolled in Cohort 1 and a minimum of 2 patients enrolled in subsequent cohorts, which will enroll using a rolling 6 design. The total enrollment for Phase 1 will therefore be approximately 9 to 21 patients.

In Phase 2, approximately 155 FGFR2-selected GC patients will be randomized 1:1 to be treated with FPA144 + mFOLFOX6 or placebo + mFOLFOX6 in 2-week cycles at an RD selected after assessment of data obtained in Phase 1. Opening of Phase 2 for enrollment will be at the discretion of the Sponsor.

The total enrollment planned for this study is approximately 167 patients.

The Phase 1 study will be conducted only in the US, while the Phase 2 study will be conducted at up to approximately 190 global study centers.

4.2 Inclusion Criteria for Phase 1 and Phase 2

Patients enrolling in either Phase 1 or Phase 2 of the study must meet ***all*** of the following inclusion criteria:

1) Disease that is unresectable, locally advanced, or metastatic (not amenable to curative therapy)
2) Understand and sign an Institutional Review Board (IRB)/Independent Ethics Committee (IEC)-approved ICF prior to any study-specific evaluation
3) Life expectancy of at least 3 months in the opinion of the investigator
4) Eastern Cooperative Oncology Group (ECOG) performance status of 0 to 1
5) Age ≥ 18 years at the time the ICF is signed
6) In sexually active patients (women of child bearing potential [WOCBP] and males), willingness to use 2 effective methods of contraception, of which 1 must be a physical barrier method (condom, diaphragm, or cervical/vault cap) until 6 months after the last dose of FPA144. Other effective forms of contraception include:
    - Permanent sterilization (hysterectomy and/or bilateral oophorectomy, or bilateral tubal ligation with surgery, or vasectomy) at least 6 months prior to screening
    - WOCBP who are on stable oral contraceptive therapy or intrauterine or implant device for at least 90 days prior to the study, or abstain from sexual intercourse as a way of living

7) Adequate hematological and biological function, confirmed by the following laboratory values within 96 hours prior to enrollment:

Bone Marrow Function

- ANC ≥ 1.5 × 10^9/L
- Platelets ≥ 100 × 10^9/L
- Hemoglobin ≥ 9 g/dL

Hepatic Function

- AST and ALT < 3 × ULN; if liver metastases, then < 5 × ULN
- Bilirubin < 1.5 × ULN except in patients with Gilbert's disease

Renal Function

- Calculated CrCl using Cockcroft Gault formula ≥ 50 mL/min *or* estimated glomerular filtrate rate (eGFR) using the Chronic Kidney Disease Epidemiology Collaboration (CKD-EPI) formula ≥ 50 mL/min (refer to Appendix 1)

8) INR or prothrombin time (PT) < 1.5 × the ULN except for patients receiving anticoagulation, who must be on a stable dose of warfarin for 6 weeks prior to enrollment
9) Measurable or non-measurable, but evaluable disease using RECIST v1.1

Additional Inclusion Criteria for Phase 1 Only

Patients enrolling in **Phase 1** of the study must also meet the following inclusion criteria:

10) Histologically or cytologically confirmed GI malignancy for which mFOLFOX6 is considered an appropriate treatment (eg, GC, colorectal carcinoma, pancreatic adenocarcinoma)
11) Patient must be a candidate to receive at least 2 doses of mFOLFOX6 chemotherapy (according to Section 3.2 of the protocol)

Additional Inclusion Criteria for Phase 2 Only

Patients enrolling in **Phase 2** of the study must also meet the following inclusion criteria:

14) Histologically documented gastric or gastroesophageal junction (GEJ) adenocarcinoma (not amenable to curative therapy)
15) Radiographic imaging of the chest, abdomen and pelvis (computed tomography [CT] preferred, magnetic resonance imaging [MRI] acceptable) performed within 28 days (+3 days) of treatment (C1D1)
16) FGFR2b overexpression as determined by a centrally performed IHC tissue test and/or *FGFR2* gene amplification as determined by a centrally performed ctDNA blood based assay

17) Patient must be a candidate for mFOLFOX6 chemotherapy
18) No prior chemotherapy for metastatic or unresectable disease (except a maximum of 1 dose of mFOLFOX6 administered while waiting for results of FGFR2 testing during the pre-screening period)
19) If prior adjuvant or neo-adjuvant therapy (chemotherapy and/or chemoradiation) has been received, more than 6 months must have elapsed between the end of adjuvant therapy and the confirmation of radiographic disease progression

4.3 Exclusion Criteria for Phase 1 and Phase 2

Patients enrolling in either Phase 1 or Phase 2 will be excluded if *any* of the following criteria apply:

1) Untreated or symptomatic central nervous system (CNS) metastases (CNS imaging not required). Patients with asymptomatic CNS metastases are eligible provided they have been clinically stable for at least 4 weeks and do not require intervention such as surgery, radiation, or any corticosteroid therapy for management of symptoms related to CNS disease
2) Impaired cardiac function or clinically significant cardiac disease, including any of the following (Criteria a through g):
   a) Unstable angina pectoris ≤ 6 months prior to enrollment
   b) Acute myocardial infarction ≤ 6 months prior to enrollment
   c) New York Heart Association Class II-IV congestive heart failure
   d) Uncontrolled hypertension (as defined as ≥ 160/90 despite optimal medical management)
   e) Uncontrolled cardiac arrhythmias requiring anti-arrhythmic therapy other than beta blockers or digoxin
   f) Active coronary artery disease
   g) Fridericia's correction formula (QTcF) ≥ 480
3) Peripheral sensory neuropathy ≥ CTCAE Grade 2
4) Active infection requiring systemic treatment or any uncontrolled infection ≤ 14 days prior to enrollment
5) Known human immunodeficiency virus (HIV) or acquired immunodeficiency syndrome (AIDS)-related illness, or known active or chronic hepatitis B or C infection
6) History of interstitial lung disease (eg, pneumonitis or pulmonary fibrosis)
7) Evidence or history of bleeding diathesis or coagulopathy

8) Radiotherapy ≤ 28 days of enrollment. Patients must be recovered from all acute radiotherapy-related toxicities. No radiopharmaceuticals (strontium, samarium) within 8 weeks of enrollment

9) Prior treatment with any selective inhibitor (eg, AZD4547, BGJ398, JNJ-42756493, BAY1179470) of the FGF-FGFR pathway

10) Ongoing adverse effects from prior systemic treatment > NCI CTCAE Grade 1 (with the exception of Grade 2 alopecia and anemia)

11) Participation in another therapeutic clinical study or receiving any investigational agent within 28 days of enrollment or during this clinical study

12) Corneal defects, corneal ulcerations, keratitis, keratoconus, history of corneal transplant, or other known abnormalities of the cornea that may pose an increased risk of developing a corneal ulcer

13) Known positivity for HER2 (as defined by a positive IHC test of 3+ or IHC of 2+ with fluorescent in situ hybridization [FISH])

14) Major surgical procedures not permitted ≤ 28 days prior to enrollment. Surgery requiring local/epidural anesthesia must be completed at least 72 hours before enrollment. In all cases, the patient must be sufficiently recovered and stable before treatment administration

15) Women who are pregnant or breastfeeding (unless the patient is willing to interrupt breastfeeding during study treatment administration and then resume 6 months after study discontinuation); WOCBP must not consider getting pregnant during the study

16) Presence of any serious or unstable concomitant systemic disorder incompatible with the clinical study (eg, substance abuse, psychiatric disturbance, uncontrolled intercurrent illness including arterial thrombosis, or symptomatic pulmonary embolism)

17) Presence of any other condition that may increase the risk associated with study participation, or may interfere with the interpretation of study results, and, in the opinion of the investigator, would make the patient inappropriate for entry in the study

18) Known allergy, hypersensitivity or contraindication to components of the FPA144 formulation including polysorbate or to platinum-containing medications, 5-FU, or leucovorin

19) History of prior malignancy, except (Criteria a through f):

    a) Curatively treated non-melanoma skin malignancy

    b) Cervical cancer in situ

    c) Curatively treated Stage I uterine cancer

    d) Curatively treated ductal or lobular breast carcinoma in situ and not currently receiving any systemic therapy

e) Localized prostate cancer that has been treated surgically with curative intent and presumed cured

f) Solid tumor treated curatively more than 5 years previously without evidence of recurrence

No waivers of these inclusion or exclusion criteria will be granted.

4.4 Patient Identification and Enrollment

Patients must be able to provide written informed consent and meet all eligibility criteria prior to enrollment. Patients who qualify for Phase 1 of the study will be enrolled in the first available cohort. A patient may be enrolled in either Phase 1 or Phase 2 of the study, but not both.

In Phase 2, patients first undergo pre-screening in which both a tissue test and a ctDNA blood assay are required. Tissue may be archival or fresh and if archival must be fresh section (cannot be previously cut slides). Patients who are determined to be FGFR2 positive by either test may immediately enter the screening period and be evaluated for eligibility for the Phase 2 study (refer to Section 3.3).

Table 4: Eligibility Based on FGFR2 Status

***FGFR2* Gene Amplification[1] (using a ctDNA blood assay)[2]**	**FGFR2b Overexpression[1] (using a tissue-based IHC assay)[3]**	**Eligibility**
Blood (+)	IHC (+)	Eligible
Blood (-)	IHC (+)	Eligible
Blood (+)	IHC (-)	Eligible
Blood (-)	IHC (-)	Ineligible

Abbreviations: ctDNA = circulating tumor DNA; FGFR2 = fibroblast growth factor receptor 2; IHC = immunohistochemistry.
Note: Patients must be FGFR2-positive by 1 of the 2 methods to be enrolled in the trial.
1 Both tests will be carried out at central laboratories
2 Requires 2 × 10 mL
3 Minimum of 5 slides required

In both Phase 1 and 3, the investigator may repeat qualifying laboratory tests and vital signs/ECGs during the screening period one time prior to enrollment if a non-qualifying finding is considered an error or an acute finding is likely to meet eligibility criteria on repeat testing.

4.5 Patient Withdrawal and Replacement

A patient must be discontinued from protocol-prescribed therapy if any of the following apply:

- Consent withdrawal at the request of the patient or their legally authorized representative
- Progression of patient's disease as assessed by the investigator per RECIST v1.1

- Any event that would pose an unacceptable safety risk to the patient
- A concurrent illness that would affect assessments of the clinical status to a significant degree
- Pregnancy at any time during the study
- At the specific request of the Sponsor or its authorized representative (eg, if the study is terminated for reasons of patient safety)

Patient replacement will be as follows:

- Patients in the Phase 1 safety run-in will be replaced if they are unevaluable for DLT (according to Section 3.2.6)
- Patients in the Phase 2 study will not be replaced

5.0 STUDY TREATMENT

5.1 FPA144

5.1.1 Identity, Packaging, Storage

FPA144 drug product is supplied for IV administration as a sterile, aqueous, colorless to slightly yellowish, pyrogen-free solution supplied in single-use glass vials. The composition of the drug product contains 20 mg/mL active ingredient, 20 mM L-histidine, 270 mM sucrose, and 0.01% (w/v) polysorbate 20 at pH 6.0. The final drug product will be provided as 2° to 8°C refrigerated liquid protected from light which is diluted for administration according to instructions provided in a separate Pharmacy Manual.

FPA144 will be supplied in a sterile vial for dilution in an IV bag for administration by the study center.

5.1.2 Administration (Phase 1 and Phase 2)

The FPA144 proposed doses were outlined above in Table 1. In Phase 1 the starting dose level of FPA144 is 6 mg/kg per dose in combination with a fixed dose chemotherapy regimen of mFOLFOX6 (Section 5.2). In Phase 2, the dose of FPA144 in combination with a fixed dose chemotherapy regimen of mFOLFOX6 will be determined by evaluation of the data from Phase 1 of the study.

FPA144 will be administered only to patients in this study using procedures described in this protocol. The dose of FPA144 is based on body weight at C1D1 and should be recalculated only if the weight changes > 10% from the C1D1 weight. If the dose is recalculated due to a >10% weight change from C1D1, the weight used for the recalculated dose should function as the new baseline for subsequent evaluation of dose recalculations. Doses should be rounded to the nearest 10 mg.

A pharmacist (or other responsible person) will prepare FPA144 for administration. After calculating the number of vials based on the patient's weight, FPA144 will be diluted in a 0.9% sodium chloride solution. Prepared FPA144 should be administered ≤ 8 hours after preparation (ambient temperature). FPA144 will be administered under medical supervision over approximately 30-minute (± 10 minutes) IV infusion via a peripheral vein or central venous catheter. The IV administration set for FPA144 infusion must contain a 0.22 µm in-line filter or a 0.22 µm syringe filter.

Infusion of FPA144 must be stopped, reduced, interrupted, or discontinued according to Sections 3.2.2 and 5.1.3.4. If a patient experiences an infusion reaction, the patient's vital signs (temperature, blood pressure [BP], heart rate [HR], and respiration rate [RR]) should be monitored during the infusion as well as every 30 minutes after the infusion for a minimum of 2 hours and until resolution of the infusion reaction.

Patients may continue receiving FPA144 administered in 2-week cycles until investigator-assessed disease progression (Phase 2 only), unacceptable toxicity, or until the patient meets any of the other protocol-specified withdrawal criteria. There is no maximum number of doses of FPA144.

Further instructions on drug preparation and administration are provided in the Pharmacy Manual.

5.1.3 FPA144 Dose Modifications

Dose modifications discussed in this section include intrapatient dose modifications. Dose escalations/modifications between cohorts in Phase 1 are described in Section 3.2.1. The dose of FPA144 for Phase 2 was determined by evaluation of the data from Phase 1 of the study, as described in Sections 3.2.3 and 3.2.4.

5.1.3.1 Dose Escalation

In Phase 1, intrapatient dose escalation will not be permitted. In Phase 2, patients will be treated at the MTD and/or RD as determined from Phase 1.

5.1.3.2 Weight

A complete physical examination including height and weight will be performed at Screening. The dose of FPA144 will be based on body weight at C1D1 and should be recalculated only if the weight changes > 10% from the C1D1 weight. If the dose is recalculated due to a >10% weight change from C1D1, the weight used for the recalculated dose should function as the new baseline for subsequent evaluation of dose recalculations.

5.1.3.3 Infusion or Hypersensitivity Reactions

If a patient experiences a Grade 3 or higher infusion or hypersensitivity reaction prior to completion of FPA144 infusion, the infusion must be stopped, and the patient promptly managed and monitored according to signs, symptoms, and local standard protocol until complete resolution of the event. Signs and symptoms of infusion or hypersensitivity reactions may include, but are not limited to fever, chills, rigors, urticaria, hypotension or hypertension, headache, wheezing, shortness of breath, hypoxia, and pulmonary infiltrates. Appropriate interventions include, but are not limited to antihistamines, corticosteroids, bronchodilators, and/or IV fluids.

If a patient experiences an infusion reaction, the patient's vital signs (temperature, BP, HR, and RR) should be monitored every 30 minutes after the infusion has been discontinued for a minimum of 2 hours or resolution of the infusion reaction, whichever takes longer.

At the investigator's discretion, the infusion of FPA144 may also be stopped if a less severe AE (Grade 1 or 2) occurs during the infusion.

If the infusion reaction resolves, at investigator discretion the infusion may be restarted at half the previous infusion rate. If, despite appropriate premedication, signs or symptoms of a hypersensitivity/infusion reaction recur, the infusion should be discontinued, and no further dosing of FPA144 will occur without consultation with the Sponsor or Sponsor's designee.

After experiencing a Grade 3 or higher infusion or hypersensitivity reaction, all subsequent infusions of FPA144 for the patient should be administered at the reduced rate (over 60 minutes) with premedication. Pre-medication may include medications such as corticosteroids, antihistamine and/or acetaminophen in accordance with local standard of care.

5.1.3.4 Toxicity: FPA144 Dose Modification Guidelines

Dose reductions for FPA144 may be permitted for patients on treatment beyond the DLT period in Phase 1 or any patient in Phase 2 and are based on local laboratories and clinical assessment. If a patient in Phase 1 requires a dose reduction or is unable to receive FPA144 during the DLT period, their reported toxicity is considered a DLT and be permanently discontinued from FPA144. Doses may be held or reduced for FPA144-related AEs following the guidelines outlined in Table 5 (Phase 2 FPA144 dose levels for dose reductions), Table 6 (non-corneal toxicities), and Table 7 (corneal toxicities).

Any patient with a corneal event which occurs within 100 days of last receiving a dose of FPA144, regardless if deemed related or not related to FPA144 should be evaluated by an ophthalmologist. Any patient with FPA144-related retinal toxicity should permanently discontinue FPA144. If dose reductions or interruptions that do not fall within these guidelines are being considered by the investigator, these will require discussion with and approval by the Sponsor or designee. Patients may resume the FPA144 if the event returns to baseline or ≤ Grade 1 in accordance with the guidelines outlined in Table 6, and Table 7.

Patients who require FPA144 dose reductions will receive the reduced dose for the remainder of the study. Cycles may be delayed to manage toxicity. Cycle delays of longer than 28 days should be discussed with the medical monitor prior to reinitiation.

Table 5: Phase 2 FPA144 Dose Levels for Dose Reductions

Dose Level	FPA144 Dose
0	15 mg/kg Q2W + 7.5mg/kg D8[1]
-1	6 mg/kg Q2W

[1] For patients who require dose reduction prior to C1D8, the C1D8 dose should be skipped, continuing with 15mg/kg Q2W

Table 6: Dose Modification Guidelines for FPA144 Related Adverse Events (Any Noncorneal, Noninfusion Toxicity[1])

FPA144-Related Toxicity Grade	Dose Schedule	New FPA144 Dose
Grade 1 or 2	No delay or missed dose required	100% of dose
Grade 3 (first occurrence)	Delay or miss dose until recovery to baseline or Grade 1	If recovery to baseline or Grade 1 within 28 days, may resume at 100% of starting dose or 1 dose lower[2]
Grade 3 (second occurrence)	Delay or miss dose until recovery to baseline or Grade 1	If recovery to baseline or Grade 1 within 28 days, may resume at 1 dose level lower[2] than previous dose or discontinue
Grade 3 (third occurrence)<br>Grade 3 which does not recover to baseline or Grade 1 within 28-day of onset event<br>Any Grade 4	Permanently Discontinue	N/A

Abbreviations: N/A = not applicable.

1 mFOLFOX6 dosing in case of FPA144 toxicity may continue regardless of FPA144 dose modifications.

2 See Table 5 for dose levels for dose reductions in Phase 2.

Any patient who reports pain or irritation of the eye or change in vision should be evaluated by an ophthalmologist.

Table 7: Dose Modification Guidelines for FPA144 (Any Related Corneal Toxicity[1])

FPA144/IMP-related Toxicity Grade	Dose Schedule	New FPA144 Dose
Grade 1	No Delay	100%
Grade 2 and Grade 3	Delay dosing, see ophthalmologist and treat with topical (ophthalmologic) antibiotics	If recovery to baseline or Grade 1 within 28 days, may resume at 100% dose
Grade 2 or Grade 3 which does not return to baseline within 28 days since last dose<br>Any Grade 4	Permanently discontinue dosing of FPA144	N/A

Abbreviations: N/A = not applicable.

1 mFOLFOX6 dosing may continue regardless of FPA144 dose modifications.

There is a ± 3-day window for the first 3 cycles (42 days) of FPA144 dosing regardless of delays in mFOLFOX6 in Phase 2 only. After the first 3 cycles, FPA144 can be delayed up to a maximum of 7 days to synchronize with mFOLFOX6 chemotherapy infusion. However, synchronization of administration of FPA144/placebo and mFOLFOX6 is not a protocol requirement. Vital signs and clinical laboratory tests must be performed within 72 hours prior to either FPA144 or mFOLFOX6 administration. Patients should only have 2 consecutive doses of FPA144 within 7 days during Cycle 1 of treatment if enrolled in Cohort 2 (Phase 1) or if the RD

of FPA144 is the dose used in Cohort 2 (Phase 2). In subsequent cycles, FPA144 is administered Q2W. The first dose of each cycle is considered Day 1 of each cycle. Cycles will repeat every 2 weeks unless there is a treatment delay. Intrapatient dose escalation above the starting dose for each patient will not be permitted. Any patient whose dose of FPA144 is decreased cannot be subsequently increased.

5.2 mFOLFOX6

Leucovorin (or levo-leucovorin), 5-FU, and oxaliplatin will be obtained from commercial sources at each participating site. Management (ie, handling, storage, administration, and disposal) of these products will be in accordance with the relevant local guidelines. For countries where the Sponsor is required to provide all study drugs, including standard-of-care drugs, the Sponsor designee will provide leucovorin, 5-FU, and oxaliplatin from a commercial supply that is clinically labelled in accordance with relevant local guidelines. For further details, refer to the manufacturer's prescribing information for the respective chemotherapies and the study Pharmacy Manual.

5.2.1 Identity, Packaging, Storage

Refer to the most current package insert for packaging, labeling, and storage information of mFOLFOX6.

FPA144 will be packaged and labeled as a 20 mL fill in ISO 20R vials by the Sponsor (or designee).

All FPA144 vials must be stored refrigerated at 2° to 8°C in accordance with the manufacturer's instructions as provided in the Pharmacy Manual. Until dispensed to patients, FPA144 will be stored in a securely locked area, accessible to authorized personnel only.

5.2.2 Administration (Phase 1 and Phase 2)

mFOLFOX6 will be administered Q2W beginning on C1D1, and will be administered at least 30 minutes after the end of the infusion of FPA144.

For further prescribing information, refer to the manufacturer's prescribing information for the respective chemotherapies and the study Pharmacy Manual. Oxaliplatin, 5-FU, and leucovorin will be administered by each site as detailed below (and as was outlined above in Table 1). The mFOLFOX6 regimen will be administered Q2W (± 3 days) until investigator-assessed disease progression (Phase 2 only), clinical disease progression, unacceptable toxicity, or the patient meets any of the other protocol-specified withdrawal criteria.

Instructions for mFOLFOX6 administration include the following:

On Day 1 of each cycle (at least 30 minutes after FPA144/placebo):

- Oxaliplatin 85 mg/m^2 IV infusion over 120 minutes
- Leucovorin 400 mg/m^2 IV infusion over 120 minutes, can be administered concurrently with oxaliplatin if using a Y connector, (if not using a Y connector, administer drugs sequentially)
  - If leucovorin is unavailable, 200 mg/m^2 levo-leucovorin may be used. Study treatment may be administered without either agent in the event that both are unavailable. Leucovorin (or levo-leucovorin) may be given at either the protocol-recommended doses or as deemed appropriate by the investigator in accordance with institutional standard of care
- Immediately after oxaliplatin and leucovorin, 5 FU 400 mg/m^2 bolus over approximately 5 minutes
- Immediately after the 5-FU bolus, 5-FU 2400 mg/m^2 as a continuous IV infusion over approximately 48 hours

Premedication may be used at the discretion of the investigator based on the local standard of care.

5.2.3 mFOLFOX6 Dose Modifications

Guidelines for dose interruptions, delays, and modifications due to toxicity are outlined in Section 5.2.3.1. If mFOLFOX6 is delayed due to toxicity, vital signs and clinical laboratory tests need to be performed within 72 hours of its administration.

In the event that oxaliplatin administration is discontinued for any reason prior to disease progression, 5-FU/leucovorin therapy may continue on a Q2W schedule until disease progression, unacceptable toxicity, or other cause for study withdrawal. In the case 5-FU/leucovorin therapy is permanently discontinued then oxaliplatin must be discontinued.

5.2.3.1 Toxicity: mFOLFOX6 Dose Modifications Guidelines

Patients should be closely monitored for mFOLFOX6 toxicity. In Phase 1, any patient who does not receive 2 complete doses of mFOLFOX6 during the DLT period due to a reason that is not a DLT or an AE related to FPA144, will be considered unevaluable and the patient will be replaced. Beyond the DLT period in Phase 1, or at any time in Phase 2, dose adjustments for any component of mFOLFOX6 are permitted according to the guidelines outlined below.

The dose of mFOLFOX6 is based on body weight at C1D1 and should be recalculated only if the weight changes > 10% from the C1D1 weight. If the dose is recalculated due to a >10% weight change from C1D1, the weight used for the recalculated dose should function as the new baseline for subsequent evaluation of dose recalculations.

Patients should be counseled to avoid exposure to cold weather during and for approximately 72 hours after each infusion.

Correct hypokalemia and hypomagnesemia prior to initiating oxaliplatin.

Evaluation for dihydropyrimidine dehydrogenase deficiency should be considered if the following events occur after 5-FU: severe diarrhea, mucositis, and myelosuppression.

The leucovorin dose is given for d, l-racemic mixture. Use half the dose for levo-leucovorin (l-leucovorin). Leucovorin will stay at a fixed dose of (400 mg/m2 and or as deemed appropriate by the investigator) will be given prior to each 5-FU dose. If 5-FU is delayed, leucovorin will be delayed. If leucovorin is not available, levo-leucovorin (200 mg/m2 or as deemed appropriate by the investigator) may be administered. Leucovorin or levo-leucovorin may be omitted from study treatment in the event that they are both unavailable.

Patients who require chemotherapy dose reductions will receive the reduced dose for the remainder of the study. The only exception to this practice will be in the case of nausea/ vomiting. If nausea and/or vomiting occur despite antiemetic therapy, the chemotherapy dose should be reduced by 25% for the next dose. If tolerated, an increase back to a 100% dose may be allowed at the investigator's discretion. Any patient who required 2 dose reductions and experienced persistent toxicity with a third dose reduction will be discontinued from all chemotherapy. Chemotherapy cycles may be delayed to manage toxicity. Cycle delays of longer than 28 days should be discussed with the medical monitor prior to reinitiation of treatment.

Dose adjustments at the start of each 14-day cycle will be based on nadir hematologic counts or maximum non-hematologic toxicity from the preceding cycle of therapy.

Dose adjustments of each agent may be made independently based on the specific types of toxicities observed.

Recommended dose adjustments for mFOLFOX6 toxicity are shown in Table 8.

Table 8: Dose Reductions and Delays for mFOLFOX6 Chemotherapy

Toxicity	Grade	Oxaliplatin	5-FU
Neurotoxicity	Persistent (≥14 days) Grade 2 neurotoxicity	Decrease from 85 mg/m² to 65 mg/m² [a]	No change
	Transient (> 7 days and ≤ 14 days) Grade 3 neurotoxicity	Decrease from 85 mg/m² to 65 mg/m² [a]	No change
	Persistent (> 14 days)<br>≥ Grade 3 neurotoxicity or any Grade 4 neurotoxicity	Permanent<br>Discontinuation	No change
Gastrointestinal	≥ Grade 3 (after prophylaxis)	Hold until toxicity is ≤ Grade 1, decrease from 85 mg/m² to 65 mg/m² [a]	Hold until toxicity is<br>≤ Grade 1, decrease by 20% [a]
Hematologic	≥ Grade 3 platelets	Hold until platelets are ≥ 75,000 then decrease from 85 mg/m² to 65 mg/m² [a]	Reduce by 20% [a]
	≥ Grade 3 neutropenia	Hold until ANC is ≥ 1500, then decrease from 85 mg/m² to 65 mg/m² [a]	Reduce by 20% [a]
Skin	≥ Grade 3 hand/foot syndrome	Hold until 5-FU resumes, then no change	Hold until ≤ Grade 1, then decrease by 20% [a]
Other	≥ Grade 3	Hold until ≤ Grade 1, then decrease from 85 mg/m² to 65 mg/m² [a]	Hold until ≤ Grade 1, then reduce by 20% [a]
Pharyngolaryngeal dysesthesia	Any	Stop infusion, then consider increase duration of infusion up to 6 hours	No change
Pneumonitis	Any	Hold, investigate; discontinue permanently if confirmed	
Hepatic Impairment	Bilirubin 1–2× ULN	No change	No change, consider decrease by 20%[a]
	Bilirubin > 2–4× ULN and/or AST/ALT is 2–4× ULN	No change	No change, consider decrease by 20%
	Bilirubin > 4× ULN and/or AST/ALT is > 4× ULN	Discontinue	Discontinue
Renal Impairment (Creatinine Clearance)	> 50 mL/min	No change	No change
	30 to < 50 mL/min	No change, consider decrease to 65 mg/m²[a]	No change
	< 30 mL/min	Discontinue	Decrease dose by 20%[a]

[a] If toxicity recurs at the same grade level after dose reduction; consider permanent discontinuation. Note that if 5-FU is permanently discontinued, oxaliplatin and leucovorin should be permanently discontinued.

Adapted from (Cheeseman 2002, Hochster 2008, Teva Pharmaceuticals USA 2012, Teva Parenteral Medicines Inc 2014, Teva Parenteral Medicines Inc 2016)

If a patient experiences Grade 1 or 2 allergic reaction to oxaliplatin, premedication should be given according to institutional practice prior to subsequent further study drug administration. If Grade 1–2 allergic reaction persists into the next cycle, escalating the appropriate premedication should be given according to institutional practice prior to administration of oxaliplatin.

For patients experiencing Grade 3-4 allergic reactions, treatment with oxaliplatin should be discontinued.

5.3 Placebo

Placebo product (only used in Phase 2) will match the FPA144 drug product. It will be supplied for IV administration as a sterile, aqueous, colorless to slightly yellowish, pyrogen-free solution supplied in single-use vials. The composition of matching placebo contains 20 mM L-histidine, 270 mM sucrose, and 0.01% (w/v) polysorbate 20 at pH 6.0. Placebo will be administered in a method that matches the administration of FPA144 to maintain blinding.

5.4 Unblinding Treatment Assignment

The Phase 2 portion of this study is double-blind. Placebo will be matched to FPA144. Treatment codes should not be broken except in emergency situations. With the exception of unblinding for the analysis (to be defined in the Statistical Analysis Plan [SAP]), all individuals involved in the conduct of the study (eg, all site staff and participants, monitoring personnel, Sponsor personnel) will be blinded to randomized treatment assignment. The decision and ability to unblind the treatment code in emergency situations resides with the investigator.

The investigator should document and provide an explanation for any premature unblinding (eg, accidental unblinding or unblinding because of a serious adverse event).

5.5 Drug Accountability

The investigator or appropriately qualified staff is responsible for maintaining accurate study treatment accountability records throughout the study.

The investigator is responsible for destroying or returning all unused study treatment to the Sponsor (or designee), and must verify that no remaining supplies are in the investigator's possession. The study site is permitted to destroy unused, used or partially used study treatment vials according to the site policy once Sponsor (or designee) approval of their documented destruction procedure has been obtained. On completion of the study, the number of FPA144 vials shipped, destroyed, and returned must be reconciled.

5.6 Investigational Product Compliance

Only qualified trained study center personnel may administer FPA144. Pharmacy personnel trained in the study requirements will monitor compliance with the treatment assignments. Records of study medication administered (date, time, and dose administered relative to time of preparation) will be recorded on the patient's eCRF.

5.7 Concomitant Medication and Treatment

Supportive care (eg, antiemetics, analgesics for pain control) may be used at the investigator's discretion and in accordance with institutional procedures. Patients should receive antiemetic and other prophylactic treatments according to the local standard of care and manufacturer's instruction. Patients should receive full supportive care, transfusions of blood and blood products, antibiotics, antiemetics, etc., when appropriate. Hematopoietic stimulating agents may be used if indicated. Concomitant anticancer therapies of any kind are not permitted.

Patients receiving oxaliplatin should not receive oral cryotherapy as this may exacerbate laryngopharyngeal dysesthesia caused by oxaliplatin. For patients on anticoagulant therapy, close monitoring of coagulation parameters is recommended.

5.8 Prohibited Therapy

Vaccination with a live vaccine should be avoided in patients receiving 5-FU because of the potential for serious or fatal infections.

6.0 STUDY ASSESSMENTS AND PROCEDURES

Unless otherwise specified, all assessments and procedures must be conducted in accordance with the Schedule of Assessments (Appendix 2 and Appendix 3).

Descriptions of assessments are provided in Sections 6.1 through 6.19 below.

Additional guidance for study assessments and procedures is provided in the following appendices:

- Schedule of Assessments for Phase 1: Appendix 2
- Schedule of Assessments for Phase 2: Appendix 3
- List of safety laboratory assessments for Phase 1 and Phase 2: Appendix 4
- Clinical Fixed Time Point Assessments: Appendix 5
- Instructions for the collection time points of PK, immunogenicity, and exploratory pharmacodynamic biomarker samples for Phase 1 are provided in Appendix 6
- Instructions for the collection time points of PK and immunogenicity samples for Phase 2 are provided in Appendix 7

6.1 Tumor Tissue Collection: Analysis for FGFR2b Overexpression for Patient Selection

6.1.1 Phase 1

Provision of tissue for retrospective FGFR2b overexpression testing by IHC, and blood for FGFR2 amplification by ctDNA, are not required for enrollment in Phase 1, but will be tested retrospectively if available.

Patients eligible for Phase 1 have unselected GI cancer (with or without FGFR2 positive status) with unresectable, locally advanced, or metastatic disease, and are candidates to receive both FPA144 and mFOLFOX6 chemotherapy. FGFR2b overexpression will be determined retrospectively using central assessment by IHC and *FGFR2* gene amplification will be determined retrospectively by using central assessment by a ctDNA blood assay.

6.1.2 Phase 2

Provision of archival (or fresh biopsy if archival tissue is not available) for Pre-Screening FGFR2b overexpression testing by IHC is required; a blood sample is also required for Pre-Screening by ctDNA for FGFR2 gene amplification. FGFR2 positive status by one of these testing methods is required for enrollment in Phase 2. Results of the centralized FGFR2 testing may take approximately 2 weeks; patients are permitted to receive up to 1 dose of mFOLFOX6 during this interim time period (pre-screening) at the discretion of the investigator. This 1 dose

of chemotherapy is not a requirement of the study and is not considered part of this clinical study.

Patients in Phase 2 of this study must consent to tumor tissue analysis and blood sample analysis. Patients will be selected for enrollment based on FGFR2b overexpression and/or *FGFR2* gene amplification, as determined by a validated IHC or ctDNA blood assay, respectively, using the central laboratory. Patients who do not demonstrate either FGFR2b overexpression using IHC or *FGFR2* gene amplification using a ctDNA blood assay will not be eligible for enrollment. However, positivity based on 1 or both of the assays is adequate to meet eligibility requirements (eg, positive by ctDNA blood assay, but negative by IHC, and vice versa) (refer to Table 4). It is the responsibility of each investigator to obtain an adequate tumor specimen for analysis of FGFR2 positivity for enrollment. A minimum of 5 slides are required. Tumor slide or tumor block specimen processing, labeling, and shipping instructions are detailed in the Laboratory Manual that will be distributed with the specimen collection kit.

Central laboratories will perform the FGFR2b overexpression and *FGFR2* gene amplification analysis using a validated IHC test and ctDNA blood assay.

For Phase 2, once tumor and blood specimens are received, analysis will be performed as efficiently as possible, and results will be communicated back to the investigator or designee.

6.2 Informed Consent Requirements

Written, signed informed consent must be collected prior to any study-specific procedures (including pre-screening). Patients who have fully consented to participation in the main study will undergo screening assessments within 28 days prior to administration of the first infusion of study treatment.

6.3 Screening

Unless otherwise specified, all Screening assessments must be completed prior to enrollment in accordance with the Schedule of Assessments (Appendix 2). During the screening period, the patient will undergo protocol-specified screening procedures to ensure all eligibility criteria are met.

6.3.1 Phase 1

For Phase 1, all patients will undergo screening assessments within 28 days (as outlined in Appendix 2). After a maximum 28-day screening period, eligible patients will initiate study treatment as described in Section 5.0. Patients who screen fail may undergo repeat screening procedures one time, but all procedures must be done within the 28-day screening window, inclusive of informed consent.

Patients may have initiated or received mFOLFOX6 chemotherapy prior to enrollment in Phase 1, but eligibility requires that the patient be a candidate to receive at least 2 additional doses of mFOLFOX6 chemotherapy (there is no upper limit on the number of previous mFOLFOX6 doses that patients may have received, nor is there a requirement for prior treatment with mFOLFOX6 in Phase 1).

Patients should have tumor tissue collected from archival material (if available) for analysis of FGFR2b overexpression by IHC retrospectively.

6.3.2 Phase 2

For Phase 2, all patients will undergo screening assessments within 28 days. After a maximum 28-day screening period, eligible patients will initiate randomized study treatment as described below. Patients who screen fail for Phase 2 may repeat screening procedures one time (not applicable to pre-screening when tissue or blood sample is determined to be evaluable and FGFR2 negative), but all procedures must be done within the 28-day screening window, inclusive of informed consent.

The description of testing for FGFR2 positivity is described in Section 3.3. Eligibility for Phase 2 will be evaluated in 2 steps:

- Step 1: Patients will provide informed consent to allow testing for FGFR2b overexpression by archival or fresh tissue with IHC and a blood sample for FGFR2 amplification by ctDNA. The time between providing informed consent and site notification of the result of the test is considered the pre-screening period (refer to Section 3.3).
- Step 2: Pre-screening allows for collection of blood and tissue to determine FGFR2 positivity using ctDNA and IHC assays. Once a patient is confirmed to have a positive result based on central testing, the screening period initiates, allowing for evaluation of all other eligibility criteria (refer to Section 4.2). Patients who test negative for FGFR2b overexpression or FGFR2 amplification will be considered pre-screen failed. Patients who test positive and enter the screening period, but do not enroll, will be considered screen failed.

6.4 Study Enrollment

6.4.1 Phase 1

Phase 1 is an open-label dose-escalation safety run-in. There is no randomization in this phase. Patients who are determined to be eligible will be enrolled sequentially. For patients participating in Phase 1, the date of enrollment is the date of first administration of study treatment.

FGFR2 status is not a requirement for enrollment in Phase 1. FGFR2b overexpression will be tested retrospectively by IHC (if tissue is available) and FGFR2 gene amplification will be tested retrospectively by ctDNA blood assay.

6.4.2 Phase 2

In Phase 2, patients who meet eligibility will be randomized 1:1 to FPA144 + mFOLFOX6 or placebo + mFOLFOX6. The date of enrollment is the date of randomization. Patients must initiate the first administration of study treatment within 3 days of enrollment. The date of first administration of study treatment is C1D1 or Study Day 1.

Enrollment in Phase 2 will begin when an RD for FPA144 has been identified by the CRC, which will not exceed the highest dose level evaluated and tolerated in Phase 1; based on the Phase 1 results, the Phase 2 dose of FPA144 is 15 mg/kg Q2W with an additional dose of 7.5 mg/kg on Cycle 1 Day 8. Patients may enroll in either Phase 1 or Phase 2, but may not enroll in both phases of the study. Opening the Phase 2 portion of the study for enrollment will be at the discretion of the Sponsor. A patient is considered enrolled when he/she is randomized.

In Phase2, FGFR2b overexpression will be determined by prospective IHC analysis and/or a ctDNA blood assay demonstrating FGFR2 gene amplification.

Eligible patients whose tumors have FGFR2b overexpression by IHC and/or *FGFR2* gene amplification by blood may consent to study participation and subsequently undergo Screening procedures, including a comprehensive ophthalmologic examination, to ensure the eligibility criteria are met.

Baseline radiographic imaging is also a study requirement. Imaging performed as standard of care that includes imaging of chest, abdomen and pelvis may be used if it has been performed within 28 days (+3 days) of treatment (C1D1).

6.4.3 Blinding

The Phase 1 part of this study will be open label.

For Phase 2, all individuals involved in the conduct of the study (ie, site staff and patients, CRO personnel, Sponsor personnel) will be blinded to randomized treatment assignment.

To facilitate the Phase 2 analysis, certain Sponsor representatives and Sponsor designees will be unblinded to treatment assignments prior to and during the analysis (including the biostatistician and external groups for bioanalytical PK/pharmacodynamic, and PRO analyses). Additional details of the analyses and unblinding will be provided in the SAP.

6.5 Medical/Oncology History and Demographics

In both Phase 1 and Phase 2, patient medical and surgical history recorded during the Screening visit includes a thorough review of significant past medical and surgical history, current conditions, concomitant therapies, alcohol and smoking history, and smoking status. At C1D1,

the medical, disease, and medication history should be updated to capture any changes from Screening.

Demographics including age, gender, race, and ethnicity will be recorded.

6.6 Safety Assessments

Safety measures will include AEs, hematology, clinical chemistry, urinalysis, vital signs, body weight, concomitant medications/procedures, ECOG performance status, targeted physical examinations, ECGs, and ophthalmology examinations. Safety measures are to be collected in both Phase 1 and Phase 2 in accordance with the Schedules of Assessments (Appendix 2, Appendix 3, and Appendix 4).

Safety measures will also include evaluation for DLTs in Phase 1 only (refer to Section 3.2.3).

An independent DMC will evaluate safety study data (AEs and SAEs) on a regular basis throughout the entire treatment phase (as prescribed in the DMC Charter) in Phase 2 (refer to Section 8.10.2).

6.6.1 Vital Signs

Vital signs (BP, HR, RR, and temperature) are to be measured in both Phase 1 and Phase 2 in accordance with the Schedules of Assessments (Appendix 2, Appendix 3, Appendix 5).

6.6.2 Physical Examinations, Height, and Weight

A complete physical examination including height and weight will be performed at Screening. Limited physical examinations (eg, symptom-directed examination of specific organ systems/body area) should be conducted in both Phase 1 and Phase 2 in accordance with the Schedules of Assessments (Appendix 2, Appendix 3) and include weight and examination of the oropharynx.

The dose of FPA144 and mFOLFOX6 is based on body weight at C1D1 and should be recalculated only if the weight changes > 10% from the C1D1 weight. If the dose is recalculated due to a > 10% weight change from C1D1, the weight used for the recalculated dose should function as the new baseline for subsequent evaluation of dose recalculations.

6.6.3 Ophthalmologic Examinations

Ophthalmologic examinations, including comprehensive ophthalmologic examinations and slit lamp examinations with fluorescein score, will be performed in both Phase 1 and Phase 2 according to Appendix 2, Appendix 3, Appendix 5. Any abnormal OCT examinations reports should be provided to the Sponsor. In addition, if a patient has any persistent ophthalmologic findings, the ophthalmologic assessments should continue every 8 weeks until resolution of findings, withdrawal of consent, death, or loss to follow up.

Comprehensive ophthalmologic examinations include fundoscopic and slit lamp examination, OCT, visual acuity, completion of fluorescein staining score form (Appendix 9), determination of intraocular pressure (IOP), and review of ocular/visual symptoms. When performing the ocular examination by the ophthalmologist, the following should be noted:

- IOP can be measured by tonometer or applanation, but should be done before dilation
- Confrontation visual fields is adequate
- OCT should include the macula: Any abnormal OCT examinations identified during the study should be provided to the Sponsor.
- Corneal staining and scoring should be done before dilation and before the IOP check as both may disrupt corneal integrity

During Phase 1, comprehensive ophthalmologic examinations will be performed during Screening, at approximately 4 weeks from C1D1, at approximately 8 weeks from C1D1, at the EOT visit (± 7 days), and at any time there are ophthalmologic symptoms up to 100 days after the last dose of FPA144. In addition to the comprehensive ophthalmologic exams, slit lamp examinations with completion of fluorescein staining score will be performed starting at Week 16 (± 7 days) from C1D1 and continue every 8 weeks until study completion.

During Phase 2, comprehensive ophthalmologic examinations will be conducted at Screening, at 8 weeks from C1D1 (± 7 days), at the EOT visit (± 7 days), and at any time there are ophthalmologic symptoms up to 100 days after the last dose of FPA144. In addition to the comprehensive ophthalmologic examinations, slit lamp examinations with completion of fluorescein staining score form will be performed starting at 16 weeks (± 7 days) from C1D1 and then every 8 weeks (± 7 days) until study completion.

In both Phase 1 and Phase 2, after the EOT visit, if the patient has any persistent corneal findings, the assessments should continue every 8 weeks until resolution of findings, withdrawal of consent, death, or loss to follow up.

6.6.4 Electrocardiograms

Twelve-lead ECGs will be performed in both Phase 1 and Phase 2 in accordance with the Schedules of Assessments (Appendix 2, Appendix 3), and Table of Clinical Fixed Time Point Assessments (Appendix 5). The investigator must review the ECG, document this review in the source documents, and record any clinically significant changes that occur during the study as an AE in the eCRF.

6.6.5 Eastern Cooperative Oncology Group Performance Status

ECOG performance status will be assessed in all patients in both Phase 1 and Phase 2 at the time points outlined in Appendix 2 and Appendix 3. The ECOG performance status is a scale used to assess how a patient's disease is progressing, assess how the disease affects the daily living

abilities of the patient, and determine appropriate treatment and prognosis. The ECOG scale is shown in Appendix 8.

6.7 Clinical Laboratory Parameters

Laboratory assessments (listed in Appendix 4) will be performed locally in both Phase 1 and Phase 2 at each study center's laboratory by means of their established methods. Before starting the study, the investigator will provide the Sponsor (or designee) with a list of the normal ranges and units of measurement. Local hematology and blood chemistry test results must be obtained within 96 hours prior to enrollment. On subsequent dosing days for both FPA144 and mFOLFOX6 (Phase 1) or FPA144/placebo and mFOLFOX6 (Phase 2), hematology and blood chemistry results must be obtained within 72 hours prior to dosing. Coagulation results need to be obtained at baseline, at Day 1 of Cycles 1 through 4 within 72 hours prior to dosing with FPA144, and whenever clinically indicated. Ongoing evaluation of INR or PT should be continued for patients who are receiving therapeutic anticoagulation according to the local standard of care.

Blood samples should be taken using standard venipuncture techniques. Laboratory assessments will be performed in both Phase 1 and Phase 2 in accordance with the Schedule of Assessments (Appendix 2, Appendix 3). Abnormal laboratory results that lead to a change in patient treatment management (eg, dose delay, requirement for additional medication or monitoring) are considered clinically significant for the purposes of this study and will be recorded on the AE page of the eCRF. Values meeting SAE criteria must be reported as SAEs. Refer to Section 6.17.2 for details around the reporting of abnormal laboratory findings as AEs.

6.8 Urinalysis

Urinalysis will be performed in both Phase 1 and Phase 2 in accordance with the Schedule of Assessments (Appendix 2, Appendix 3). Urinalysis includes protein, glucose, blood, pH, and ketones on Day 1 for both FPA144 and mFOLFOX6 of odd cycles (every other cycle). If findings clinically significant, a microscopic evaluation will be performed per institutional standard.

6.9 Pregnancy

Pregnancy is an exclusion criterion and WOCBP must not be considering getting pregnant during the study. Serum beta-human chorionic gonadotropin (ß-hCG) (evaluated by local laboratories) and urine pregnancy tests will be performed only on WOCBP. A serum pregnancy test in WOCBP obtained within 96 hours prior to enrollment is mandatory in both Phase 1 and Phase 2. Subsequent pregnancy tests should be performed in accordance with the Schedule of Assessments (Appendix 2, Appendix 3).

In the event of suspected pregnancy, a serum pregnancy test should be repeated. Patients who become pregnant during the study must discontinue study treatment immediately.

The Sponsor must be notified of any patient who becomes pregnant while participating in this study. Although pregnancy is not an AE, all pregnancies must be followed to conclusion to determine their outcome. Male patients should immediately inform the investigator if their partner becomes pregnant during the study or within 6 months after the last dose of study medication. It is the responsibility of the investigator or designee to report any pregnancy in a patient that occurs during the study by completing the Pregnancy Reporting Form. Please contact the study monitor to receive the Pregnancy Reporting Form upon learning of a pregnancy.

Notification of the pregnancy including the anticipated date of birth should be provided on a Pregnancy Reporting Form within 24 hours of awareness and reported using the same procedure as described for reporting SAEs (Section 6.17.4). If the pregnancy is to be terminated, the anticipated date of termination should be provided. A pregnancy report form should also be completed by the investigator within 24 hours after learning of the pregnancy in the partner of a male patient during the study or within 6 months after the last dose of study medication using the procedure for SAE reporting (Section 6.17.4). An investigator who is contacted by the male patient or his pregnant partner may provide information on the risks of the pregnancy and the possible effects on the fetus, to support an informed decision in cooperation with the treating physician and/or obstetrician.

The patient will be asked to provide information on the outcome of the pregnancy, including premature termination should the case arise. Spontaneous miscarriages, premature termination of the pregnancy, congenital abnormalities and any other abnormalities in the mother or fetus/newborn will be reported as SAEs. Information on the status of the mother and child will be forwarded to the Sponsor. Attempts should also be made to collect and report details of the course and outcome of any pregnancy in the partner of a male patient exposed to drug. The pregnant partner will need to sign and Authorization for Use and Disclosure of Pregnancy Health Information to allow for follow-up of the pregnancy. Once the authorization has been signed the investigator will update the Pregnancy Report reform with additional information on the course and outcome of the pregnancy. Generally, follow-up will be in accordance with regulatory guidance and at least 6 to 8 weeks after the estimated delivery date.

Pregnancies that occur during the first 6 months of the Follow-up Period should be reported to the Sponsor and followed as described above.

6.10 Tumor Assessments

Tumor assessments will be performed in Phase 2 only. Tumor assessments should consist of clinical examination and appropriate imaging techniques (preferably CT scans with appropriate slice thickness per RECIST v1.1; MRIs are acceptable). The same methods used to detect lesions at baseline are to be used to follow the same lesions throughout the clinical study.

Tumor response assessment will be performed by the investigator per RECIST v1.1 guidelines.

Tumor scans will be performed at Screening (within 28 days +3 days) of treatment [C1D1] and then every 8 weeks (± 7 days) from C1D1 until 12 months and then every 12 weeks (± 14 days) thereafter. Scans should be obtained per schedule regardless of drug interruption or discontinuation.

Patients who discontinue study treatment (the last administered dose of FPA144/placebo and mFOLFOX6) for reasons other than disease progression or withdrawal of consent, radiographic tumor assessments will continue according to the protocol (approximately every 8 weeks ± 7 days) until 12 months and then every 12 weeks ± 14 days thereafter) until either the patient initiates additional anticancer therapy or experiences disease progression.

6.11 Pharmacokinetic Assessment

Blood samples to determine serum FPA144 concentration will be acquired from each patient as outlined in the Study Flowchart for PK, Immunogenicity, and Exploratory Biomarker Blood Sample Collections for Phase 1 (Appendix 6) and in the Study Flowchart for PK and Immunogenicity Blood Samples Collections for Phase 2 (Appendix 7). These samples will be collected and processed according to the instructions provided in a separate Laboratory Manual.

PK parameters will be estimated using noncompartmental analysis, though compartment analysis may be employed if appropriate. Serum concentration-time data from FPA144-004 will be pooled with data from other studies for integrated population PK analysis and exposure-response relationship assessment.

6.12 Pharmacodynamic Biomarker Analysis Using Blood

Pharmacodynamic assessments will only be conducted in Phase 1.

Pharmacodynamic samples will be collected at the time points specified in Appendix 6. Tumor tissue provided for evaluation of FGFR2 status, if available, will be retrospectively analysed for FGFR2b overexpression using IHC.

Blood samples provided for evaluation of FGFR2 status will be collected prior to the first dose of study treatment and analysed retrospectively for *FGFR2* gene amplification using a ctDNA blood assay.

Blood samples for exploratory pharmacodynamic biomarker analysis (eg, FGFR2 pathway markers FGF7 and FGF10) will be collected from patients enrolled in the Phase 1 portion of the study at the time points specified in Appendix 6 and processed by the central laboratory.

6.13 Immunogenicity

Immunogenicity, defined as an immune response to FPA144, will be assessed by measurement of total anti-FPA144 antibodies from all patients. Immunogenicity testing will consist of screening, confirmation, and ADA concentration. Additional characterization of a confirmed anti-FPA144 antibody response may be conducted.

Samples for immunogenicity, anti-drug antibody (ADA) assessment will be drawn in both Phase 1 and Phase 2 from each patient at the time points outlined in Appendix 6 and Appendix 7. Samples for immunogenicity testing will be collected and processed at central laboratory according to the instruction provided in the Laboratory Manual.

6.14 Immunohistochemistry

In Phase 1 collection of samples is optional for FGFR2b overexpression using IHC (archival sample with fresh cuts).

Phase 2 requires collection of samples for FGFR2b overexpression using IHC (archival sample with fresh cuts or a fresh biopsy). Patients whose samples show FGFR2b overexpression will be considered positive. For patients in Phase 1, results of the IHC do not need to be available prior to enrollment and dosing. For patients participating in the Phase 2 portion of the trial, a blood sample for ctDNA testing is required prior to consent and enrollment. Patients may be enrolled on Phase 2 based on positive IHC or ctDNA (refer to Section 6.15).

6.15 Circulating Tumor DNA Blood Assay

In both phases of the study, samples for ctDNA blood assay will be collected prior to the first dose of study treatment (C1D1) and analyzed for *FGFR2* gene amplification in the central laboratory. For patients in Phase 1, results of the ctDNA do not need to be available prior to enrollment and dosing. For patients participating in the Phase 2 portion of the trial, a blood sample for ctDNA testing is required prior to consent and enrollment. Patients may be enrolled based on a positive ctDNA or IHC test.

6.16 Patient Reported Outcomes / Quality of Life Scales

In Phase 2, the EQ-5D-5L QOL questionnaire and the EORTC QLQ-C30 will be administered in accordance with Appendix 3.

The EQ-5D-5L questionnaire was developed by the EuroQol Group, which is a standardized measure to provide utilities for clinical and economic appraisal. It uses a descriptive system and a visual analogue scale (VAS). The descriptive system has 5 dimensions: mobility, self-care, usual activities, pain/discomfort, and anxiety/depression, and each dimension has 5 levels consisting of no problems, slight problems, moderate problems, severe problems, and extreme problems.

The EORTC QLQ is an integrated system for assessing the health-related quality of life of cancer patients participating in international clinical trials. The EORTC uses a modular approach to QOL assessment, consisting of a core questionnaire (EORTC QLQ-C30) to be administered, if necessary with a module specific to tumor site, treatment modality or a QOL dimension (eg, GC-specific module is QLQ-STO22).

6.17 Adverse Events

Assessment of AEs will follow the guidelines provided in the NCI CTCAE version 4.03 in Phase 1 and version 5.0 in Phase 2. AEs will be assessed as outlined in Section 6.17.3. Abnormal laboratory results that lead to a change in patient treatment management (eg, dose delay, requirement for additional medication or monitoring) are considered clinically significant for the purposes of this study and will be recorded on the AE page of the eCRF. Events meeting SAE criteria must be reported as SAEs (Section 6.17.3.1).

6.17.1 Collection of Adverse Events

In Phase 1, any new symptoms, injury, or worsening of symptoms that occur during the screening period (ie, following signing of the ICF, but prior to first infusion [C1D1]), will be considered pretreatment events and reported on the Medical History page of the eCRF, unless they directly correlate to a study-related procedure in which case they will be reported on the AE eCRF page. Otherwise, AE reporting will begin at the time of infusion of C1D1 (day of first infusion) and continue until completion of the EOT visit or 4 weeks (28 days) after the last dose of FPA144.

In Phase 2, AEs should not be reported during the pre-screening period unless they are related to a pre-screening procedure, as patients are not yet enrolled on the study at that time. The worst grade of AEs occurring during the screening period should be reported as described above for the Phase 1 screening period. AE reporting will continue until the EOT visit. SAE reporting will initiate at study enrollment unless the event is related to a study procedure. Symptomatic corneal and all retinal events are considered of special interest and should be reported up to 100 days from the last dose of FPA144.

Since the IHC and ctDNA blood results may require up to 2 weeks to complete, patients eligible for entering Phase 2 of this study may not be able to delay start of standard chemotherapy while waiting to receive the IHC or ctDNA blood result. Therefore, patients are permitted to receive 1 dose of mFOLFOX6 prior to enrolling in this clinical trial, while waiting for FGFR2 status to be confirmed. AEs due to this single dose of therapy that are ongoing on Study Day 1 will be captured on the medical history eCRF.

SAEs occurring after the EOT visit should be reported to the Sponsor by the investigator only if the investigator considers them related to FPA144 or mFOLFOX6. SAEs should always be recorded on the AE eCRF and reported to the Sponsor using the SAE report form.

6.17.2 Definitions

An AE is any untoward medical occurrence that occurs in a patient administered a pharmaceutical product, and which does not necessarily have to have a causal relationship with this treatment. An AE can therefore be any unfavorable and unintended sign (including abnormal laboratory finding), symptom, or disease temporally associated with the use of an investigational product, whether or not considered related to the product. Abnormal laboratory findings that are not considered clinically significant will be recorded only on the laboratory eCRF pages and not on the AE pages. Abnormal laboratory results that are considered clinically significant in the investigator's opinion are also to be recorded on the AE eCRF. Relationship (reasonable causal relationship) to drug therapy and counter measures undertaken will be noted on the eCRF.

All AEs including intercurrent illnesses that occur during the study, from the time of administration of study treatment, will be documented on the AE eCRF. Concomitant illnesses, which existed prior to the day of the first study infusion, will not be considered AEs unless they worsen by at least 1 grade during the treatment period. Intensity (severity) grade will be defined according to the NCI-CTCAE, version 4.03 in Phase 1 and version 5.0 in Phase 2. Pre-existing conditions will be recorded on the Medical History eCRF.

A treatment-emergent AE will be defined as an AE that begins or worsens in severity after at least 1 dose of study treatment (FPA144 and/or mFOLFOX6) has been administered.

Diagnostic and therapeutic non-invasive and invasive procedures, such as surgery, will not be reported as an AE, but the procedure and/or therapeutic treatment should be recorded on the appropriate eCRF. The medical condition for which the procedure was performed must be reported as an AE (or as part of the patient's medical history, if the procedure precedes the initiation of study-prescribed treatment). Signs and symptoms associated with disease progression itself should not be reported as an AE or SAE if the diagnosis is available. Disease progression itself is an endpoint and not an AE or SAE.

6.17.3 Assessment of Adverse Events

Each AE will be assessed by the investigator with regard to seriousness, intensity (severity), causality, and the outcome and action taken. All AEs, regardless of the relationship to study treatment, will be recorded on the AE eCRF. This includes potential end-organ toxicity, eg, renal (proteinuria), hepatic, and cardiovascular (increased BP) effects, and effects on wound healing. All AE reports should contain a brief description of the event, date of onset, ongoing or date of resolution, intensity, treatment required, relationship to study treatment, action taken with the study treatment, outcome, and whether the event is classified as serious as described below.

6.17.3.1 Seriousness

An SAE is defined as any untoward medical occurrence that at any dose:

- Results in death. Death may occur as a result of the underlying disease process. All events other than progression of underlying disease that result in death during the reporting period up to 28 days following the last dose of study treatment must be treated as an SAE and reported as such
- Is life-threatening (patient is at immediate risk of death from the event as it occurred)
- Requires inpatient hospitalization (formal admission to a hospital for medical reasons) or prolongation of existing hospitalization
- Results in persistent or significant disability or incapacity
- Is a congenital anomaly or birth defect

Medically significant events that may not result in death, are not life-threatening, or do not require hospitalization may be considered SAEs when, based on appropriate medical judgment, they may jeopardize the patient and may require medical or surgical intervention to prevent one of the outcomes listed in this definition. Examples of such events are intensive treatment in an emergency room or at home for allergic bronchospasm, blood dyscrasias, or convulsions that do not result in hospitalization, or development of drug dependency or drug abuse.

Medical and scientific judgment should be exercised in deciding whether a case is considered medically significant or serious and whether expedited reporting is appropriate.

Hospitalization for an event solely related to disease progression is not considered an SAE. Hospitalization for an elective or planned procedure to treat a pre-existing condition is not considered an SAE unless it results in one of the outcomes listed above.

6.17.3.1.1 Adverse Events of Special Interest (AESI)

An AESI (serious or non-serious) is an event of medical concern considered potentially associated to the investigational product or disease under study, for which ongoing monitoring and rapid communication by the investigator to the Sponsor is necessary. Ocular events associated with symptomatic corneal involvement and symptomatic and asymptomatic retinal involvement are considered events of special interest in this study. Such events might warrant further investigation in order to characterize the safety profile of the product. Depending on the nature of these events, rapid communication by FivePrime to other parties (eg, regulators) might also be warranted.

6.17.3.1.2 Intensity (Severity)

Investigators need to assess the severity of AEs according to the guidelines provided in NCI-CTCAE, version 4.03 in Phase 1 and version 5.0 in Phase 2.

CTCAE v 4.03 and v 5.0 Severity Grades are:

- Grade 1: Mild; asymptomatic or mild symptoms; clinical or diagnostic observations only; intervention not indicated; mild AE
- Grade 2: Moderate; minimal, local or non-invasive intervention indicated; limiting age-appropriate instrumental activities of daily living; moderate AE
- Grade 3: Severe or medically significant, but non-immediately life-threatening; hospitalization or prolongation of hospitalization indicated; disabling; limiting self-care activities of daily living; severe AE
- Grade 4: Life-threatening consequences; urgent intervention indicated
- Grade 5: Fatal AE

If the AE is not specified in the CTCAE or the study protocol, the grading of severity will be assessed as mild (Grade 1), moderate (Grade 2), severe (Grade 3), life-threatening (Grade 4), or death due to the AE (Grade 5) using the following definitions:

- Mild: The patient is aware of the event or symptom, but the event or symptom is easily tolerated.
- Moderate: The patient experiences sufficient discomfort to interfere with or reduce his or her usual level of activity.
- Severe: Significant impairment of functioning: the patient is unable to carry out usual activities.
- Very severe (life-threatening): The patient's life is at risk from the event.

6.17.3.2 Causality

The investigator will assess the causality/relationship between the study treatment and the AE and record that assessment on the eCRF.

The most likely cause of an SAE (eg, disease under treatment, concomitant disease, concomitant medication, other) will be indicated on the eCRF with details of the concomitant disease or medication or other cause.

The causal relationship of the AE to study treatment will be assessed by means of a question: 'Is there a reasonable possibility that the AE may have been caused by the study treatment?' Answer Yes or No.

The description below provides guiding principles for the investigator to make casualty assessments.

- Yes, there is a reasonable possibility that the AE may have been caused by the study treatment:
  - Follows a reasonable temporal sequence from administration of the study treatment
  - Could not be reasonably explained by the patient's clinical state, environmental or toxic factors, or other therapies administered to the patient
  - Disappears or decreases on cessation or reduction in dose of the study treatment
  - Follows a known pattern of response to the study treatment
  - Reappears or worsens on re-challenge
- No, there is no reasonable possibility that the AE may have been caused by the study treatment:
  - Does not follow a reasonable temporal sequence from administration of the study treatment
  - Could be reasonably explained by the patient's clinical state, environmental or toxic factors, or other therapies administered to the patient
  - Does not follow a known pattern of response to the study treatment
  - Does not reappear or worsen on re-challenge

The relatedness for SAEs will also be assessed and documented on the AE eCRF.

If the causality of the AE requiring discontinuation is confirmed to be due to one of the study treatments in the combination therapy, the other drug may be continued according to the protocol schedule under the following scenarios:

- Timely resolution of the AE based on the treatment modification table
- Clinical benefit is shown by the patient based on investigator assessment

6.17.3.3 Outcome and Action Taken

The investigator will record the action taken and outcome for each AE according to the following criteria:

- Action Taken
  - Dose Not Changed
  - Drug Interrupted
  - Drug Withdrawn

  - Drug Delayed
  - Dose Increased
  - Dose Reduced
  - Not Applicable
  - Unknown
- Outcome
  - Fatal
  - Not Recovered/Not Resolved
  - Recovered/Resolved
  - Recovered/Resolved with Sequelae
  - Recovering/Resolving

6.17.4 Reporting Serious Adverse Events

Any SAEs, whether or not considered related to treatment with FPA144 or mFOLFOX6, must be reported by the investigator to the Sponsor or Sponsor's designee within 24 hours of the investigator becoming aware of the event and must be recorded on both the SAE form and AE eCRF. Additional SAE information including medications or other therapeutic measures used to treat the event, action taken with the study treatment due to the event, and outcome/resolution of the event will be recorded on the SAE form. Forms for reporting SAEs will also be provided to the study centers.

A copy of the SAE forms must be faxed or electronically communicated **within 24 hours** to the attention of the ICON Pharmacovigilance Safety Specialist:

ICON Medical and Safety Services
[redacted]

The investigator should not wait to receive additional information to fully document the event before notification of a SAE, though additional information may be requested. The minimum information that is required for an initial SAE report is as follows:

- Patient study number
- Investigator name and study center number
- Event term
- Event onset date

- Serious criteria
- Relationship to study treatment(s)

As applicable, information from relevant laboratory results, hospital case records, and autopsy reports should be obtained.

Serious AEs occurring after the EOT visit should be reported to the Sponsor by the investigator if the investigator considers the event related to the study treatment. Events of special interest (symptomatic corneal and all retinal events) should be reported to the Sponsor up to 100 days from last dose of FPA144.

The investigator and Sponsor will review each SAE report and evaluate the seriousness and causal relationship of the event to study treatment. In the event of a disagreement about causality, the most conservative assessment will be used. In addition, the Sponsor will evaluate the expectedness according to the FPA144 IB. Based on the investigator and Sponsor's assessment of the event, a decision will be made concerning the need for further action.

The Sponsor or its designee is responsible for submitting reports of AEs associated with the use of the drug that are both serious and unexpected the Food and Drug Administration (FDA), according to 21 Code of Federal Regulations (CFR) 312.32, and to other regulatory authorities, according to national law and/or local regulations. All investigators participating in ongoing clinical studies with the study medication will receive copies of these reports for prompt submission to their IRB or IEC.

The Sponsor or its designee will submit all safety updates and periodic reports to the regulatory authorities as required by applicable regulatory requirements.

6.17.5 Follow-up of Adverse Events

All treatment-related SAEs experienced by a study patient, will be monitored until the event has resolved or stabilized, any abnormal laboratory values have returned to baseline or stabilized at a level acceptable to the investigator and Sponsor, there is a satisfactory explanation for the changes observed, or the patient is lost to follow-up.

All unresolved related AEs should be followed by the investigator until the events are resolved, the patient is lost to follow-up, or the AE is otherwise explained. The investigator should notify the study Sponsor of any death or AE occurring at any time after a patient has discontinued or terminated study participation that may reasonably be related to this study. Follow-up of events of special interest (symptomatic corneal and all retinal events) will be determined on a case by case basis depending on the prognostic resolution of the event. The Sponsor should also be notified if the investigator should become aware of the development of cancer or of a congenital anomaly in a subsequently conceived offspring of a patient that has participated in this study.

6.18 End of Treatment

EOT assessments are outlined in Appendix 2 and in Appendix 3 for Phase 1 and Phase 2, respectively. All patients should return to the clinic approximately 28 days after their last dose of study treatment (ie, the last administered dose of FPA144 or any component of mFOLFOX6), or in the event that a patient discontinues prematurely from the study, for EOT follow-up assessments.

In Phase 2, after discontinuation of study treatment for reasons other than disease progression or withdrawal of consent, tumor assessments will continue until the patient initiates additional anticancer therapy or progresses.

Symptomatic corneal and all retinal events are considered of special interest and should be reported up to 100 days from the last dose of FPA144.

6.19 Long Term Follow Up (Phase 2)

LTFU assessments are outlined in Appendix 3 for Phase 2 only. In Phase 2, LTFU assessments for survival consist of clinic visits, telephone calls, or patient registries (in line with national legislation and prevailing data protection laws) approximately every 3 months (± 1 month) after the EOT visit until up to 24 months after the last patient is enrolled in the study, or until death, loss to follow-up, withdrawal of consent, or study termination by the Sponsor (whichever occurs first), which will be considered the EOS. During the Follow-up Period, if the patient undergoes anticancer therapy, this should be documented.

During the first 6 months of the LTFU Period (for patients in Phase 2), and for the first 6 months after the EOT visit (for patients in Phase 1), any known pregnancy that occurs should be reported to the Sponsor and followed as described in Section 6.9.

Serious AEs occurring after the EOT visit should be reported to the Sponsor by the investigator if the investigator considers there is a causal relationship with the study treatment (refer to Section 6.17.3.2).

6.19.1 Additional Follow-up for Patients without Progression at the End of Treatment Visit (Phase 2) (Scan Follow-up)

Scan Follow-Up assessments are outlined in Appendix 3 for Phase 2 only. If a patient discontinues study treatment (FPA144 and/or mFOLFOX6) for reasons other than disease progression or withdrawal of consent, they will have follow-up visits and continue to undergo tumor assessments until disease progression or the initiation of additional anticancer therapy.

7.0 STATISTICAL METHODS

Before database lock, a SAP will be finalized, providing detailed methods for the analyses including the summary of conduct of study and comparability of treatment group in Phase 2. Any deviations from the planned analyses will be described and justified in the final clinical study report.

7.1 Analysis Populations

The following analysis populations are defined for the study:

- Safety Population: all enrolled patients who have received any portion of at least 1 dose of study treatment (FPA144 + mFOLFOX6 or placebo + mFOLFOX6).
- DLT-evaluable Population: all patients enrolled in Phase 1 of the study who received at least 2 doses of FPA144 (except for Cohort 2 [must have received 3 doses of FPA144]) and mFOLFOX6 and completed Cycles 1 and 2 of treatment, or who experienced a DLT in Cycle 1 or Cycle 2.
- PK-evaluable Population: all patients who have received at least 1 dose of FPA144 and have had adequate PK assessments drawn for determination of the FPA144 concentration. Adequacy will be determined on a case-by-case basis and will be assessed prior to analysis of the blood samples.
- Intent-to-treat (ITT) Population: all randomized patients (Phase 2).
- Efficacy-evaluable Population: all patients who met eligibility criteria, received at least 1 dose of study treatment (FPA144 + mFOLFOX6 or placebo + mFOLFOX6), and have at least 1 postbaseline disease assessment and no major protocol deviation that could introduce bias in any efficacy assessment.

7.2 General Considerations

The total enrollment planned for this study is approximately 167 patients. 12 patients evaluable for DLT were enrolled into Phase 1.

For Phase 2, efficacy and tolerability will be examined by enrollment of approximately 155 patients with FGFR2-selected GC, randomized 1:1 to receive FPA144 + mFOLFOX6 or placebo + mFOLFOX6. Eligible patients will be stratified according to geographic region, prior treatment status (*de novo* versus adjuvant/neo-adjuvant), and administration of a single dose of mFOLFOX6 prior to enrollment (yes or no).

7.3 Power and Sample Size in Phase 2

This Phase 2 study is designed to assess the hazard ratio (HR) of PFS for FPA144 + mFOLFOX6 compared with placebo + mFOLFOX6. It is planned to observe 84 PFS events in order to achieve 71% power to detect an HR of 0.67 for PFS, at the 1-sided alpha of 0.1. Assuming an

exponential distribution, this corresponds approximately to an 50% increase in median PFS (e.g. from 5 months to 7.5 months). Statistical significance (at 1-sided alpha of 0.1) for PFS will occur with an observed HR=0.756, corresponding approximately to a 32.3% increase in observed median PFS (e.g. from 5 months to 6.6 months).

One hundred fifty-five patients have been randomized (1:1) during 13 months of accrual. It is projected to observe 84 PFS events with approximately 11 additional months of follow-up.

There is no planned interim analysis for this study.

Statistical significance for PFS will occur with an estimated HR= 0.756 at the final analysis, corresponding approximately to an increase of 32.3% in median progression-free survival of from 5 months to 6.61 months.

Approximately 155 patients will be randomized (1:1) during 13 months of accrual, with approximately 12 additional months of follow-up in order to achieve the targeted number of primary events with 15% dropout.

Power and sample size estimates were estimated using EAST®(V6.4).

7.4 Efficacy Analyses

7.4.1 Analysis of Primary Efficacy Endpoint

In Phase 2, the primary efficacy analysis is the comparison of PFS in patients treated with FPA144 + mFOLFOX6 versus placebo + mFOLFOX6.

The primary endpoint, PFS, is defined as time from randomization until the date of radiological disease progression based on investigator assessment (using RECIST v1.1) or death from any cause, whichever comes first. A clinical deterioration determined by an investigator will not be considered as a progression event. Data will be censored at the date of last adequate tumor assessment for subjects:

- who do not have documented progression or die, or
- who start new anticancer therapy before documented progression or death without documented progression, or
- who have ≥ 2 consecutive missing tumor assessments before documented progression or death without documented progression

If a subject does not have a baseline tumor assessment, then PFS will be censored at the date of randomization, regardless of whether or not radiographic progression or death has been observed.

The analyses of PFS will be performed using the ITT population, and will be conducted using a stratified log-rank test. The stratification factors will be the same used to stratify the randomization schedule as documented in the interactive voice and web response system (IXRS).

The median PFS and the associated 95% CI for each treatment arm will be estimated using the Kaplan-Meier method. The hazard ratio (HR= $\lambda_{FPA144+ mFOLFOX6}/ \lambda_{placebo+mFOLFOX6}$) will be estimated using a Cox regression model with treatment group as the only main effect and stratifying by the same stratification factors as were used for the stratified log-rank test. An unstratified HR will also be presented.

The detailed sensitivity analyses of PFS based upon different definitions of progression events and censoring rules will be provided in a SAP to address the following:

- To correct for potential ascertainment bias in follow-up schedules between the 2 treatment groups;
- To evaluate PFS based upon investigator claims: progression events will include investigator assessment of disease progression and initiation of subsequent anticancer therapy

7.4.2 Analysis of Secondary Efficacy Endpoints

Analyses of secondary endpoints OS and ORR will be conducted hierarchically. The formal hypotheses regarding effects on OS and ORR will be tested hierarchically at a one-sided level of 0.1. The OS will be tested first and if it is significant, the ORR will be tested next. The family-wise type I error rate of testing primary and secondary endpoints will be in a control by employing this gate-keeping testing procedure at a one-sided level of 0.1.

Overall survival is defined as time from randomization until death from any cause. Subjects who are lost to follow-up or do not have a date of death at the time of data cutoff will be censored at the last date that they were known to be alive. Overall survival will be analyzed in the similar manner as for PFS.

The analysis of ORR will be performed based on the ITT population. In the analysis of ORR, patients who don’t have any postbaseline adequate tumor assessments will be counted as non-responders. Formal hypothesis testing of ORR will be performed using the stratified Cochran-Mantel-Haenszel test at a one-sided level of 0.1. The stratification factors will be the same used to stratify the randomization schedule as documented in the IXRS. In addition, the analysis of ORR will be performed based on Efficacy-evaluable Population as sensitivity analysis.

7.4.3 Analysis of Exploratory Endpoints

7.4.3.1 Efficacy

Duration of response (DOR) is defined, for patients with an objective response, as the time from onset of radiographic documentation of objective response to disease progression evaluated by investigator using RECIST v1.1 or death due to any cause. Patients who have not experienced disease progression or death at the time of analysis will be censored at the time of the last adequate tumor assessment. Median duration of response and its associated 95% CI will be estimated, by treatment group, using Kaplan-Meier methods.

The detailed analyses of exploring correlation between identified FGFR2 status in tumor tissue and/or ctDNA blood assay, as determined by IHC and blood-based molecular diagnostic assay, with PFS, OS, and objective response per RECIST v1.1 will be provided in the SAP.

7.4.3.2 Quality of Life

The analysis of PROs (assessed using the EORTC QLQ-C30 questionnaires) will be performed according to the EORTC Scoring and Reference Values Manual. The details of the analyses will be specified in the Statistical Analysis Plan. The respective subscales from the EORTC QLQ-C30 will be used to evaluate and compare the time to deterioration in abdominal pain, reflux, eating restrictions (premature safety), weight loss, appetite loss, and fatigue between treatment arms. All scores and subscales will be assessed through descriptive summary statistics.

7.5 Safety Analyses

All AEs will be coded using the Medical Dictionary for Regulatory Activities Version 20.1 (MedDRA v 20.1). The investigator will classify the severity of AEs using the CTCAE v 4.03 in Phase 1 and version 5.0 in Phase 2. A treatment-emergent adverse event (TEAE) is defined as any event with an onset date on or after date of first dose of study treatment, or any event present before treatment that worsens after treatment. Only TEAEs with an onset date prior to 28 days after the date of last dose will be tabulated in summary tables. The exceptions are symptomatic corneal events or any retinal events which will be captured within 100 days of the last dose of FPA144.

Clinical laboratory data will be summarized by the type of laboratory test. The number and percentage of patients who experience abnormal (ie, outside of reference ranges) and/or clinically significant abnormal laboratories after study treatment administration will be presented for each clinical laboratory measurement. For each clinical laboratory measurement, descriptive statistics will be provided for baseline and all subsequent posttreatment scheduled visits. Changes from baseline to the posttreatment visits will also be provided. Descriptive statistics of vital signs will also be provided in a similar manner. In addition, shift from baseline in CTCAE

grade (where applicable) and by high/low flags (where CTCAE grades are not defined) will be presented in a similar manner.

7.5.1 Safety Analyses in Phase 1

Safety analyses will be performed for patients included in the safety population. The incidence of DLTs, incidence of TEAEs, clinical laboratory abnormality (eg, shift table), vital signs, corneal and retinal findings, and ECGs will be tabulated and summarized by dose level. Additionally, incidence of TEAEs leading to dosing reduction or dose discontinuation will be tabulated and summarized

7.5.2 Safety Analyses in Phase 2

The analyses of safety will include all patients who receive any study treatment (FPA144 + mFOLFOX6, or placebo + mFOLFOX6) throughout the study and provide any posttreatment safety information. The incidence of TEAEs, clinical laboratory abnormality (eg, shift table), vital signs, corneal and retinal findings, and ECGs will be tabulated and summarized by treatment group.

No formal comparisons of safety endpoints are planned.

7.6 Pharmacokinetic Analyses

PK blood samples will be collected to measure the concentration of FPA144 in serum. Immunogenicity blood samples will be collected to measure the level of anti-FPA144 antibodies in serum. Samples for PK and immunogenicity assessment will be drawn from each patient at the time points outlined in Appendix 6 (for Phase 1) and Appendix 7 (for Phase 2). These samples will be collected and processed according to the instructions provided in a separate Laboratory Manual.

Individual and mean (± SD) serum FPA144 concentration-time data will be tabulated and plotted by dose level. FPA144 PK parameters will be estimated from the serum FPA144 concentration-time data from Phase 1 using a non-compartmental analysis method with IV infusion input. Alternative methods may be considered. The C_{max} and C_{trough} of FPA144 will be estimated from the serum concentration-time data for Phase 2. The time to achieve steady state, dose linearity, and accumulation ratio will be evaluated as data allow. Estimated individual and mean (± SD) PK parameters will be tabulated and summarized by dose level. Other descriptive statistics might be reported for serum FPA144 concentration-time data and estimated PK parameters.

The impact of immunogenicity on FPA144 exposure will be assessed, tabulated, and summarized by dose level as appropriate and applicable.

Serum concentration-time data from FPA144-004 will be pooled with data from other studies for integrated population PK analysis and exposure-response relationship assessment, which will be presented in a separate report.

7.7 Changes in the Planned Analyses

If discrepancies exist between the text of the statistical analysis as planned in the protocol and the final SAP, a protocol amendment will not be issued, and the SAP will prevail.

8.0 ETHICAL, LEGAL, AND ADMINISTRATIVE ASPECTS

8.1 Data Quality Assurance

The Sponsor (or designee) may conduct a site visit prior to study initiation at a site to verify the qualifications of each investigator, inspect the site facilities, and inform the investigator of responsibilities and for ensuring study compliance and procedures for adequate and correct documentation.

The investigator is required to prepare and maintain adequate and accurate case histories designed to record all observations and other pertinent data for each study patient. All information recorded on the eCRFs for this study must be consistent with the patients' source documentation (ie, medical records).

8.2 Data Protection

The investigator is required to prepare and maintain adequate and accurate case histories designed to record all observations and other pertinent data for each study patient. All information recorded in the case histories or eCRFs for this study must be consistent with the patients' source documentation (ie, medical records).

Investigator and study center must maintain the anonymity of participating patients. Each patient will be assigned a unique patient identifier by the Sponsor that may consist of one or more of the following: the patient's patient number, initials, and/or birth date. Investigator and study center will include only such patient identifier on any eCRFs or other patient records, datasets or other documents that are transferred or submitted to the Sponsor or Sponsor's designee during the course of the study and will redact patient's name and all other personally-identifiable patient information from such documents. In addition, investigator must maintain in confidence any documents that include personally-identifiable patient information (e.g., the signed ICF) and take all reasonable precautions to prevent the disclosure of any personally identifiable patient information by any employee or agent of the study center to any third party or otherwise into the public domain.

The investigator or study center must inform study patients that their personal study-related data will be used by the Sponsor in accordance with local data protection law and must explain the level of disclosure to patients. In addition, each patient must be informed that his/her medical records may be examined by Clinical Quality Assurance auditors or other authorized personnel appointed by the Sponsor, by IRB/IEC members of the study center, and by inspectors from regulatory authorities.

The Sponsor (or designee) may conduct a site visit prior to study initiation at a site to verify the qualifications of each investigator, inspect the site facilities, and inform the investigator of investigator's responsibilities for ensuring study compliance and procedures for preparing and maintaining adequate and correct eCRFs.

8.3 Electronic Case Report Forms and Source Documentation

All data obtained during this study should be entered in the eCRFs promptly. All source documents from which eCRF entries are derived should be placed in the patient's medical records. eCRF fields for which source documents will typically be needed include laboratory assessments, physical examination reports, nursing notes, ECG recordings, hospital records, CT scans, and/or MRI) reports.

The eCRFs for each patient will be checked against source documents at the study site by the site monitor.

Instances of missing or uninterpretable data will be discussed with the investigator for resolution.

8.4 Access to Source Data

During the study, a monitor will perform routine site visits to review protocol compliance, compare eCRFs and individual patient's medical records, assess drug accountability, and ensure that the study is being conducted according to pertinent regulatory requirements. eCRF entries will be verified with source documentation. The review of medical records will be performed in a manner to ensure that patient confidentiality is maintained.

In accordance with ICH Good Clinical Practices (GCP) guidelines, the investigator must ensure provision of sufficient time, reasonable space, and adequate qualified personnel for the monitoring visits. The purpose of the visits is verifying adherence to the study protocol and the completeness, consistency, and accuracy of data entered on the eCRF and other documents. Moreover, regulatory authorities, IRBs, IECs, and/or the Sponsor's Quality Assurance group may wish to carry out such source data checks and/or on-site audit inspections. Direct access to source data will be required for these inspections and audits; they will be carried out giving due consideration to data protection and medical confidentiality. The investigator must assure that the Sponsor and/or Sponsor's designee will receive the necessary support to complete these activities.

All participating centers should take particular care in ensuring that original imaging source data (e.g., CT images, MRI images, echo images, etc.) are maintained and accessible for monitoring, and that these original source data are then archived on a long-term basis in compliance with ICH GCP Section 8. These images must be stored in a secure location until the Sponsor or Sponsor's designee authorizes their destruction and must be retrievable by study patient number in the event of an audit.

8.5 Data Processing

The Data Management Plan, to be developed during the initiation phase of the study, will include specifications for consistency and plausibility checks on data and will also include data-handling rules for obvious data errors. All processes for data processing and query handling will be described in the Data Management Plan.

8.6 Archiving Study Records

Each study site will maintain a study file, which should contain, at minimum, the IB, the protocol and any amendments, the protocol for tissue sampling, drug accountability records, correspondence with the IEC/IRB and the Sponsor (or designee), and other study-related documents.

The investigator agrees to keep records and those documents that include (but are not limited to) the identification of all participating patients, medical records, study-specific source documents, source worksheets, all original signed and dated ICFs, copies of all eCRFs, query responses, and detailed records of drug disposition to enable evaluations or audits from regulatory authorities and the Sponsor or its designees.

The investigator shall retain records required to be maintained for a period of 5 years following the date a marketing application in an ICH region is approved for the drug for the indication for which it is being investigated or, if no application is to be filed or if the application is not approved for such indication, until at least 5 years after the investigation is discontinued. However, these documents should be retained for a longer period if required by the applicable regulatory requirement(s) or if needed by the Sponsor. In addition, the investigator must make provision for the patients' medical records to be kept for the same period of time.

No data should be destroyed without the agreement of the Sponsor. Should the investigator wish to assign the study records to another party or move them to another location, the Sponsor must be notified in writing of the new responsible person and/or the new location, as applicable.

Patients' medical records and other original data will be archived in accordance with the archiving regulations or facilities of the investigational site.

8.7 Good Clinical Practice

The procedures set out in this study protocol are designed to ensure that the Sponsor and investigator abide by GCP guidelines of the ICH and the Declaration of Helsinki (1989). The study also will be carried out in compliance with local legal requirements.

8.8 Informed Consent

All information about the clinical study, including the patient information and the ICFs, is prepared and used for the protection of the human rights of the patient according to ICH GCP guidelines and the Declaration of Helsinki.

The ICF, prepared by the investigator with the assistance of the Sponsor, must be approved along with the study protocol by the IEC/IRB and be acceptable to the Sponsor before any patient is enrolled on the study. Written informed consent will be obtained from each patient according to applicable regulatory and legal requirements. Copies of the signed ICFs will be retained by the patient and the original will be filed in the investigator's study center file, unless otherwise agreed by the Sponsor the the study center. The investigator will not undertake any investigation specifically required only for the clinical study until valid consent has been obtained. The terms of the consent and when it was obtained must be documented in the source documents and in the eCRF.

If a protocol amendment is required, the ICFs may need to be revised to reflect the changes to the protocol. If the ICF is revised, it must be reviewed and approved by the appropriate IRB/IEC and the revised version signed by all patients subsequently enrolled in the study as well as those currently enrolled in the study.

8.9 Protocol Approval and Amendment

Before the start of the study, the study protocol and/or other relevant documents will be approved by the IRB/IEC, in accordance with local legal requirements. The Sponsor, Sponsor's agents, and investigator must ensure that all ethical and legal requirements have been met before the first patient is enrolled in the study.

This protocol is to be followed exactly. To alter the protocol, amendments must be written, receive approval from the appropriate personnel, and receive IRB/IEC approval prior to implementation (if appropriate). Following approval, the protocol amendment will be submitted to the IND application under which the study is being conducted.

8.10 Study Committee and Central Review

8.10.1 Cohort Review Committee

The Phase 1 safety run-in for this study will have a CRC consisting of representatives from the Sponsor, the Medical Monitors and 1 or more investigators from actively participating sites. The CRC will assess the safety of the dose escalation (Phase 1) on a regular basis. All dose escalation decisions will be based on assessment of DLTs, overall safety and tolerability, and will be made after the last patient enrolled in each cohort has completed the first 2 treatment cycles. In addition, when selecting the dose(s) to be developed further, consideration will be made of toxicities observed both during and beyond the DLT evaluation period and assessment

of the proportion of patients who receive planned doses at various dose levels and the percentage of patients that required dose reductions and dose discontinuations for toxicity.

8.10.2 Data Monitoring Committee

The study will have a DMC that will operate independently from the Sponsor and the clinical investigators. The primary responsibilities of the DMC are to review the accumulating safety data from the Phase 2 study on a regular and ad hoc basis. In addition, the DMC will review the unblinded results of the OS interim analysis along with safety data to balance risk and benefit, and make recommendations to the Sponsor based on the data totality. Safety data from the Phase 2 study will be provided at regular intervals to the DMC in the form of unblinded summary reports and/or data listings from an independent statistical center designated by the Sponsor.

The first meeting of the DMC is planned to occur after a minimum number of patients (ie, 50 patients) have had the opportunity to complete a cycle of study treatment. Subsequent meetings are planned to occur periodically (eg, quarterly) and ad hoc at the request of the DMC members or the Sponsor. Details regarding DMC membership, schedule and format of meetings, format for presentation of data, access to interim data, method and timing of providing interim reports to the DMC, and other issues relevant to committee operations will be described in the DMC charter.

8.11 Premature Termination of the Study

If the investigator, Sponsor, or Medical Monitor becomes aware of conditions or events that suggest a possible hazard to patients if the study continues, the study may be terminated. The study may also be terminated early at the Sponsor's discretion in the absence of such a finding.

Conditions that may warrant termination include, but are not limited to:

- Discovery of an unexpected, significant, or unacceptable risk to the patients enrolled in the study
- Failure to enroll patients at an acceptable rate
- Decision on the part of the Sponsor to suspend or discontinue development of the drug

8.12 Confidentiality

All study findings and documents will be regarded as confidential. The investigator and members of his/her research team must not disclose such information without prior written approval from the Sponsor.

The anonymity of participating patients must be maintained. Patients will be identified on eCRFs and other documents submitted to the Sponsor or Sponsor's designee by their patient number, initials, and/or birth date. Study patients are not to be identified by name, and any information sent to the Sponsor or Sponsor's designee should have patient identifiers redacted and replaced with patient numbers. Documents that include the name of the patient (e.g., the signed informed consent) must be maintained in confidence by the investigator. The investigator will take all reasonable precautions to prevent the disclosure by any employee or agent of the study center to any third party or otherwise into the public domain.

8.13 Other Ethical and Regulatory Issues

If a significant safety issue is identified, either from an individual case report or review of aggregate data, then the Sponsor will issue prompt notification to applicable regulatory authorities and investigators. Investigators will then notify local IRB/IECs as deemed appropriate based on individual IRB/IEC policy.

A significant safety issue is one that has a significant impact on the course of the clinical trial or program (including the potential for suspension of the trial program or amendments to protocols) or warrants immediate update of informed consent.

9.0 REFERENCES

Ahn, S., J. Lee, M. Hong, *et al.* (2016). "FGFR2 in Gastric Cancer: Protein Overexpression Predicts Gene Amplification and High H-Index Predicts Poor Survival." Modern Pathology: 1-9.

Al-Batran, S.-E., J. T. Hartmann, R. Hofheinz, *et al.* (2008). "Biweekly Fluorouracil, Leucovorin, Oxaliplation. and Docetaxel (FLOT) for Patients with Metastatic Adenocarcinoma of the Stomach or Esophagogastric Junction: A Phase II Trial of the Arbeitsgemeinschaft Internistische Onkologie " Annals of Oncology **19**: 1882-1887.

Andre, F., M. Ranson, E. Dean, *et al.* (2013). Abstract LB-145: Results of a Phase I Study of AZD4547, an Inhibitor of Fibroblast Growth Factor Receptor (FGFR), in Patients with Advanced Solid Tumors. AACR 104th Annual Meeting 2013, Washington, DC, American Association of Cancer Research.

Bang, Y., E. Cutsem, A. Feyereislova, *et al.* (2010). "Trastuzumab in Combination with Chemotherapy Versus Chemotherapy Alone for Treatment of HER2-Positive Advanced Gastric or Gastro-Oesophageal Junction Cancer (ToGA): A Phase 3, Open-Label, Randomised Controlled Trial." The Lancet **376**: 687-697.

Bron, A., V. Evans and J. Smith (2003). "Grading of Corneal and Conjuctival Staining in the Context of Other Dry Eye Tests." Cornea **22**(7): 640-650.

Brown, A. P., C. L. Courtney, L. M. King, *et al.* (2005). "Cartilage Dysplasia and Tissue Mineralization in the Rat Following Administration of a FGF Receptor Tyrosine Kinase Inhibitor." Toxicologic Pathology **33**: 449-455.

Catenacci, D., S. Rha, Y.-J. Bang, *et al.* (2017). "Updated AntiTumor Activity and Safety of FPA144, and ADCC-Enhanced Isoform-Specific Monoclonal Antibody, in Patients with FGFR2b+ Gastric Cancer." Journal of Clinical Oncology **35**(15): 4067.

Cheeseman, S., S. Joel, J. Chester, *et al.* (2002). "A 'Modified de Gramont' Regimen of Fluorouracil, Alone and With Oxaliplatin, for Advanced Colorectal Cancer." British Journal of Cancer **87**: 393-399.

Chen, J., D. Hunter, M. Stampfer, *et al.* (2003). "Polymorphism in the Thymidylate Synthase Promoter Enhancer Region Modifies the Risk and Survival of Colorectal Cancer." Cancer Epidemiology, Biomarkers & Prevention **12**: 958-962.

Davidson, M. and I. Chau (2016). "Variations in Outcome for Advanced Gastric Cancer Between Japanese and Western Patients: A Subgroup Analysis of the RAINBOW Trial." Transl Gastroenterol Hepatol **1**(46): 1-4.

Deshpande, A. M., S. Palencia, D. I. Bellovin, *et al.* (2014). Expression of FGFR2b in Gastric Cancer as Measured by Immunohistochemistry With a Highly Specific Monoclonal Antibody. American Association for Cancer Research, San Diego, CA, Five Prime Therapeutics, Inc.

Dienstmann, R., R. Bahleda, A. Barbara, *et al.* (2014). First in human study of JNJ-42756493, a potent pan fibroblast growth factor receptor (FGFR) inhibitor in patients with advanced solid tumors. AACR 105th Annual Meeting, San Diego, CA.

Ford, H., A. Marshall, J. Bridgewater, *et al.* (2014). "Docetaxel Versus Active Symptom Control for Refractory Oesophagogastric Adenocarcinoma (COUGAR-02): An Open-Label, Phase 3 Randomised Controlled Trial." Lancet Oncology **15**: 78-86.

Fuchs, C., T. Doi, R. Jang, *et al.* (2017). "KEYNOTE-059 Cohort 1: Efficacy and Safety of Pembrolizumab (Pembro) Monotherapy in Patients with Previously Treated Advanced Gastric Cancer." Journal of Clinical Oncology **35**(15): 4003.

Fuchs, C., J. Tomasek, C. Yong, *et al.* (2014). "Ramucirumab Monotherapy for Previously Treated Advanced Gastric or Gastro-Oesophageal Junction Adenocarcinoma (REGARD): an International, Randomised, Multicentre, Placebo-Controlled, Phase 3 Trial." Lancet Oncology **383**: 31-39.

Gemo, A., A. Deshpande, S. Palencia, *et al.* (2014). FPA144: A Therapeutic Antibody for Treating Ptients with Gastric Cancers Bearing FGFR2 Gene Amplification. AACR 105th Annual Meeting 2014, San Diego California.

GlaxoSmithKline (2017). "Study to Evaluate GSK3052230 in Combination with Paclitaxel and Carboplatin, or Docetaxel or as Single Agent in Subjects with Solid Malignancies and Deregulated Fibroblast Growth Factor (FGF) Pathway Signaling ". from https://clinicaltrials.gov/ct2/show/NCT01868022?term=NCT01868022&rank=1.

Gravalos, C. and A. Jimeno (2008). "HER2 in Gastric Cancer: A New Prognostic Factor and a Novel Therapeutic Target." Annals of Oncology **19**: 1523-1529.

Hall, T., Y. Yu, S. Eathiraj, *et al.* (2016). "Preclinical Activity of ARQ 087, a Novel Inhibitor Targeting FGFR Dysregulation." PLoS ONE **11**(9): 1-19.

Hattori, Y., H. Itoh, S. Uchino, *et al.* (1996). "Immunohistochemical Detection of K-sam Protein in Stomach Cancer." Clinical Cancer Research **2**: 1373-1381.

Hattori, Y., H. Odagiri, H. Nakatani, *et al.* (1990). "K-sam, an Amplified Gene in Stomach Cancer, is a Member of the Heparin-Binding Growth Factor Receptor Genes." Proceedings of the National Academy of Sciences (PNAS) **87**: 5983-5987.

Hochster, H., L. Hart, R. K. Ramanathan, *et al.* (2008). "Safety and Efficacy of Oxaliplatin and Fluoropyrimidine Regimens with or without Bevacizumab As First-Line Treatment of Metastatic Colorectal Cancer: Results of the TREE Study." Journal of Clinical Oncology **26**(21).

Hofmann, M., O. Stoss, D. Shi, *et al.* (2008). "Assessment of a HER2 Scoring System for Gastric Cancer: Results from a Validation Study." Histopathology **52**: 797-805.

Jung, E., E. Jung , S. Min*, et al.* (2012). "Fibroblast Growth Factor Receptor 2 Gene Amplification Status and its Clinicopathologic Significance in Gastric Carcinoma." Human Pathology **43**(10): 1559-1566.

Kamangar, F., G. Dores and W. Anderson (2006). "Patterns of Cancer Incidence, Mortality, and Prevalence Across Five Continents: Defining Priorities to Reduce Cancer Disparities in Different Geographic Regions of the World." Journal of Clinical Oncology **24**(14): 2137-2150.

Kang, Y.-K., W.-K. Kang, D.-B. Shin*, et al.* (2009). "Capecitabine/Cisplatin Versus 5-Fluorouracil/Cisplatin as First-Line Therapy in Patients with Advanced Gastric Cancer: A Randomised Phase III Noninferiority Trial." Annals of Oncology **20**: 666-673.

Kang, Y., N. Boku, T. Satoh*, et al.* (2017). "Nivolumab in Patients with Advanced Gastric or Gastro-Oesophageal Junction Cancer Refractory to or Intolerant of, at Least Two Previous Chemotherapy Regimens (ONO-4538-12, ATTRACTION-2): A Randomised, Double-Blind, Placebo-Controlled, Phase 3 Trial." The Lancet **390**: 2461-2471.

Keam, B., S.-A. Im, S.-W. Han*, et al.* (2008). "Modified FOLFOX-6 Chemotherapy in Advanced Gastric Cancer: Results of Phase II Study and Comprehensive Analysis of Polymorphisms as A Predictive and Prognostic Cancer." BMC Cancer **8**(148): 1-10.

Kim, D., J. Kim, S.-H. Lee*, et al.* (2003). "Phase II Study of Oxaliplatin, 5- Fluorouracil and Leucovorin in Previously Platinum-Treated Patients with Advanced Gastric Cancer." Annals of Oncology **14**: 383-387.

Li, B., L. Zhao, H. Guo*, et al.* (2009). "Characterization of a Rituximab Variant with Potent Antitumor Activity Against Rituximab-Resistant B-Cell Lymphoma." Blood **114**(24): 5007-5015.

Matsumoto, K., T. Arao, T. Hamaguchi*, et al.* (2012). "FGFR2 Gene Amplification and Clinicopathological Features in Gastric Cancer " British Journal of Cancer **106**(4): 727-732.

Miki, T., D. P. Bottaro, T. P. Fleming*, et al.* (1992). "Determination of Ligand-Binding Specificity by Alternative Splicing: Two Distinct Growth Factor Receptors Encoded by a Single Gene." Proceedings of the National Academy of Sciences **69**: 246-250.

National Cancer Institute (2015). "SEER Stat Fact Sheets: Stomach Cancer." Retrieved February 25, 2016, from http://seer.cancer.gov/statfacts/html/stomach.html.

Ohtsu, A., M. Shah, E. Van Cutsem*, et al.* (2011). "Bevacizumab in Combination with Chemotherapy As First-Line Therapy in Advanced Gastric Cancer: A Randomized, Double-Blind, Placebo-Controlled Phase III Study." Journal of Clinical Oncology **29**(30): 3968-3976.

Ohtsu, A., S. Yoshida and N. Saijo (2006). "Disparities in Gastric Cancer Chemotherapy Between the East and West " Journal of Clinical Oncology **24**(14): 2188-2196.

Okines, A., A. Norman, P. McCloud, *et al.* (2009). "Meta-Analysis of the REAL-2 and ML17032 Trials: Evaluating Capecitabine-Based Combination Chemotherapy and Infused 5-Fluorouracil-Based Combination Chemotherapy for the Treatment of Advanced Oesphago- Gastric Cancer." Annals of Oncology **20**(9): 1529-1534.

Powers, J., S. Palencia, S. Foy, *et al.* (2016). "FPA144, A Therapeutic Monoclonal Antibody Targeting the FGFR2b Receptor, Promotes Antibody Dependent Cell-Mediated Cytotoxicity and Stimulates Sensitivity to PD-1 Blockade in the 4T1 Syngeneic Tumor Model in Mice." 1.

Pullarkat, S., J. Stoehlmacher, V. Ghaderi, *et al.* (2001). "Thymidylate Synthase Gene Polymorphism Determines Response and Toxicity of 5-FU Chemotherapy." The Pharmacogenics Journal **1**: 65-70.

Sawaki, A., Y. Ohashi, Y. Omuro, *et al.* (2011). "Efficacy of Trastuzumab in Japanese Patients with HER2-Positive Advanced Gastric or Gastroesophageal Junction Cancer: A Subgroup Analysis of the Trastuzumab for Gastric Cancer (ToGA) Study." Gastric Cancer **15**: 313-322.

Seo, S., S. Park, M. Ryu, *et al.* (2016). "Prognostic Impact of Fibroblast Growth Factor Receptor 2 Gene Amplification in Patients Receiving Fluoropyrimidine and Platinum Chemotherapy for Metastatic and Locally Advanced Unresectable Gastric Cancers." Oncotarget: 1-11.

Sequist, L. V., P. Cassier, A. Varga, *et al.* (2014). Phase I Study of BGJ398, a Selective Pan-FGFR Inhibitor in Genetically Preselected Advanced Solid Tumors. AACR 2014 Annual Meeting, San Diego, CA.

Shinkawa, T., K. Nakamura, N. Yamane, *et al.* (2003). "The Absence of Fucose but Not the Presence of Galactose or Bisecting N-Acetylglucosamine of Human IgG1 Complex-type Oligosaccharides Shows the Critical Role of Enhancing Antibody-dependent Cellular Cytotoxicity." THe Journal of Biological CHemisty **278**(5): 3466-3473.

Smyth, E., M. Verheij, W. Allum, *et al.* (2016). "Gastric Cancer: ESMO Clinical Practice Guidelines for Diagnosis, Treatment, and Follow-up " Annals of Oncology **27**(5): v38-v49.

Su, X., P. Zhan, P. Gavine, *et al.* (2014). "FGFR2 Amplification has Prognostic Significance in Gastric Cancer: Results From a Large International MultiCentre Study " British Journal of Cancer **110**: 967-975.

Taiho Oncology Inc (2015). "A Dose Finding Study Followed by a Safety and Efficacy Study in Patients with Advanced Solid Tumors or Multiple Myeloma with FGF/FGFR-Related Abnormalities." from https://clinicaltrials.gov/ct2/show/NCT02052778?term=NCT02052778&rank=1.

Teva Parenteral Medicines Inc (2014). "Leucovorin Calcium for Injection Package Insert." 1-13.

Teva Parenteral Medicines Inc (2016). "ADRUCIL- Highlights of Prescribing Information." 1-12.

Teva Pharmaceuticals USA (2012). "Oxaliplatin: Highlights of Prescribing Information." 1-43.

Teva Pharmaceuticals USA Inc (2016). "Warfarin Sodium Highlights of Prescribing Information." 1-51.

The GASTRIC Group (2010). "Benefit of Adjuvant Chemotherapy for Resectable Gastric Cancer." JAMA **303**(1729-1737): 1729.

Thuss-Patience, P., A. Kretzschmar, D. Bichev, *et al.* (2011). "Survival Advantage for Irinotecan Versus Best Supportive Care as Second-Line Chemotherapy in Gastric Cancer- A Randosmied Phase III Study of the Arbeitsgemeinschaft Internistische Onkologie (AIO)." European Journal of Cancer **47**: 2306-2314.

Tournigand, C., T. Andre, E. Achille, *et al.* (2004). "FOLFIRI Followed by FOLFOX6 or the Reverse Sequence in Advanced Colorectal Cancer: A Randomized GERCOR Study." Journal of Clinical Oncology **22**(2): 229-237.

Turner, N. and R. Grose (2010). "Fibroblast Growth Factor Signalling: From Development to Cancer." Nature **10**: 116-129.

Ulrich, C., J. Bigler, C. Velicer, *et al.* (2000). "Searching Expressed Sequence Tag Databases: Discovery and Confirmation of a Common Polymorphism in the Thymidylate Synthase Gene." Cancer Epidemiology, Biomarkers & Prevention **9**: 1381-1385.

van Hazel, G., V. Heinemann, N. Sharma, *et al.* (2016). "SIRFLOX: Randomized Phase III Trial Comparing First-Line mFOLFOX6 (Plus or Minus Bevacizumab) Versus mFOLFOX6 (Plus or Minus Bevacizumab) Plus Selective Internal Radiation Therapy in Patients with Metastatic Colorectal Cancer." Journal of Clinical Oncology **34**(15): 1723-1731.

Waddell, T., M. Verheij, W. Allum, *et al.* (2014). "Gastric Cancer: ESMO-ESSO-ESTRO Clinical Practice Guidelines for Diagnosis, Treatment, and Follow-up " Radiotherapy and Oncology **110**: 189-194.

Wagner, A., W. Grothe, J. Haerting, *et al.* (2006). "Chemotherapy in Advanced Gastric Cancer: A Systematic Review and Meta-Analysis Based on Aggregrate Date." Journal of Clinical Oncology **24**(18): 2903-2909.

Wilke, H., K. Muro, E. Custem, *et al.* (2014). "Ramucirumab Plus Paclitaxel Versus Placebo Plus Paclitaxel in Patients with Previously Treated Advanced Gastric or Gastro-Oesophageal Junction Adenocarcinoma (RAINBOW): A Double-Blind, Randomised Phase 3 Trial." Lancet Oncology **15**: 1224-1235.

Wu, Y.-M., F. Su, S. Kalyana-Sundaram, *et al.* (2013). "Identifi cation of Targetable FGFR Gene Fusions in Diverse Cancers." Cancer Discovery **3**(6): 636-647.

Zhao, X., S. Singh, C. Pardoux*, et al.* (2010). "Targeting C-type Lectin-Like Molecule-1 for Antibody-Mediated Immunotherapy in Acute Myeloid Leukemia." Haematologica **95**(1): 71-78.

10.0 APPENDICES

Appendix 1: Formulas to Calculate Renal Function....118
Appendix 2: Schedule of Assessments: Dose-Escalation Safety Run-in (Phase 1)....119
Appendix 3: Schedule of Assessments: Randomized, Placebo-Controlled Portion (Phase 2)....123
Appendix 4: Laboratory Evaluations....128
Appendix 5: Clinical Fixed Time Point Assessments....129
Appendix 6: Study Flowchart for Pharmacokinetic, Immunogenicity, and Exploratory Pharmacodynamic Biomarker Blood Sample Collections for Phase 1....131
Appendix 7: Study Flowchart for FPA144 Pharmacokinetic and Immunogenicity Blood Sample Collections for Phase 2....133
Appendix 8: ECOG Performance Status....134
Appendix 9: Fluorescein Stain Grading Systems....135

APPENDIX 1: FORMULAS TO CALCULATE RENAL FUNCTION

Renal function should be determined using either the Cockcroft Gault formula *or* the Chronic Kidney Disease Epidemiology Collaboration (CKD-EPI) formula.

Cockcroft Gault formula:

$$CrCl = \frac{(140-age)*weight}{72*SCr}(* \, 0.85 \; if \; female)$$

Abbreviations/Units: age in years; weight in kg; SCr = serum creatinine in mg/dL.

CKD-EPI formula:

eGFR = 141 x min(SCr/κ, 1)α x max(SCr /κ, 1)-1.209 x 0.993Age x 1.018 [if female] x 1.159 [if Black]

Abbreviations/Units: eGFR (estimated glomerular filtration rate) = mL/min/1.73 m^2; SCr = standardized serum creatinine in mg/dL; κ = 0.7 (females) or 0.9 (males); α = -0.329 (females) or -0.411 (males); min = indicates the minimum of SCr.

APPENDIX 2: SCHEDULE OF ASSESSMENTS: DOSE-ESCALATION SAFETY RUN-IN (PHASE 1)

	Screening	Study Treatment Period: Cycles 1 and 2					Study Treatment Period: Cycle 3, Cycle 4 and Subsequent Cycles					
	Day --28 to Day 0	Cycle 1 Day 1	Cycle 1 Day 3	Cycle 1 Day 8	Cycle 2 Day 1	Cycle 2 Day 3	Cycle 3[b] Day 1	Cycle 4[c] Day 1	Clinical Fixed Time Point Assessments[d]	As Clinically Indicated	Other Time Points	EOT[e,f]
**Procedure[a]**	**Week 0**	**Week 1**	**Week 1**	**Week 2**	**Week 3**	**Week 3**	**≥Week 4**					
Archival Tissue Provided for IHC (if available)[g]	X											
Sample for ctDNA Blood Assay[h]		X										
Informed Consent[i]	X											
Review/Confirm Eligibility Criteria	X											
Medical/Oncology and Medication History	X	X										
Demography/Baseline Characteristics	X											
Physical Examination[j,k]	X[j]	X		X	X		X	X				X
ECOG Performance Status[l]	X						X[l]				X[l]	X
Vital Signs[m]	X	X	X	X	X	X	X	X		X		X
12-lead ECG[n]	X									X		X
Comprehensive Ophthalmologic Examination (including Slit Lamp)[o]	X								X[d,o]	X	X[o]	X
Slit Lamp Examination[p]									X[d,p]		X[p]	
Clinical Safety Laboratory Sampling[q]	X[q]	X[q]		X	X[q]		X[q]	X[q]		X		X
Pregnancy Test[r]	X[r]	X[r]					X[r]			X	X[r]	X
Urinalysis[s]	X	X					X[s]			X	X[s]	X
PK Samples[t]		X	X	X	X	X	X	X			X	X
Immunogenicity Sampling[u]		X			X		X				X	X

APPENDIX 2: SCHEDULE OF ASSESSMENTS: DOSE-ESCALATION SAFETY RUN-IN (PHASE 1) (CONTD)

	Screening	Study Treatment Period: Cycles 1 and 2					Study Treatment Period: Cycle 3, Cycle 4 and Subsequent Cycles					
	Day --28 to Day 0	Cycle 1 Day 1	Cycle 1 Day 3	Cycle 1 Day 8	Cycle 2 Day 1	Cycle 2 Day 3	Cycle 3[b] Day 1	Cycle 4[c] Day 1	Clinical Fixed Time Point Assessments[d]	As Clinically Indicated	Other Time Points	EOT[e,f]
**Procedure[a]**	**Week 0**	**Week 1**	**Week 1**	**Week 2**	**Week 3**	**Week 3**	**≥Week 4**					
FPA144 Administration[j,w,x]		X		X[x]	X		X	X				
mFOLFOX6 Administration[y]		X			X		X	X				
Adverse Events[Z]	X[z]	X ————————————————————————— X										
Concomitant Medications	X	X ————————————————————————— X										

Note: A cycle of treatment is 2 weeks.

a Unless specified, procedure is to be completed within ± 72 hours of scheduled time point and to be synchronized with the study treatment administration day.

b And subsequent odd cycles beyond Cycle 3 (except for PK, immunogenicity, and pharmacodynamic blood sample collections, which are described in Appendix 6).

c And subsequent even cycles beyond Cycle 4 (except for PK, immunogenicity, and pharmacodynamic blood sample collections, which are described in Appendix 6).

d Clinical Fixed Time Point Assessments should be completed as originally scheduled from C1D1 regardless of dosing days or dose delays. Refer to Appendix 5 for collection days.

e EOT assessments should be performed approximately 28 (+3) days following the last study treatment administration

f For the first 6 months after the EOT visit, any pregnancy that occurs should be reported to the Sponsor.

g Tumor tissue collection from archival material (if available) for retrospective IHC analysis of FGFR2b overexpression by IHC upon eligibility confirmation.

h Sample for ctDNA blood assay will be collected prior to the first dose of study treatment (Cycle 1 Day 1; C1D1) and analyzed retrospectively for *FGFR2* gene amplification.

i Written, signed informed consent must be collected prior to any study-specific procedures. The most recent IRB/EC approved ICF must be signed.

j Complete physical examination including height and weight will be measured at Screening only. Limited physical examinations should be conducted, including weight and examination of the oropharynx, thereafter.

k After Cycle 1, the FPA144 should be recalculated only if the weight changes > 10% from the C1D1 weight. If the dose is recalculated due to a >10% weight change from C1D1, the weight used for the recalculated dose should function as the new baseline for subsequent evaluation of dose recalculations. Doses should be rounded to the nearest 10 mg.

l ECOG Performance Status will be assessed at Cycle3 Day 1 and Day 1 of every other subsequent cycle (odd cycles) until the EOT visit.

[m] Vital signs (blood pressure, heart rate, respiration, and temperature [°C]) are to be measured after 5 minutes of rest, at one time at the Screening visit and Cycle 1 Day 3, Cycle 2 Day 3, EOT and as clinically indicated. On C1D1 (all cohorts), and Cycle 1 Day 8 (Cohort 2 only) vital sign measurements should be at the following time points: Predose, and 0.5, 1, 2, and 4 hours from the start of the FPA144 infusion. On subsequent dosing days, vital sign measurements should be predose and at 0.5, 1, and 2 hours from the start of FPA144 infusion.

[n] 12-lead ECG after patient rests for 5 minutes prior to recording.

[o] Comprehensive ophthalmologic examinations (conducted at Screening, at 4 weeks from C1D1, at 8 weeks from C1D1, and at the EOT visit only, ±7 days) include fundoscopic and slit lamp examination (with fluorescein staining score form), ocular coherence tomography (OCT), visual acuity, completion of fluorescein staining score form, determination of intraocular pressure, and review of ocular/visual symptoms. The comprehensive ophthalmologic examination will be repeated at any time if clinically indicated when changes in visual acuity or visual symptoms are reported by patients up to 100 days after the last dose of FPA144. After the EOT visit if the patient has any persistent ophthalmologic findings, the assessments should continue every 8 weeks until resolution of findings, withdrawal of consent, death, or loss to follow up. All abnormal OCT examinations should be submitted to the Sponsor.

[p] Slit lamp examinations alone, (with completion of fluorescein staining score form), should be conducted for all patients starting at approximately week 16 and then every 8 weeks through EOT visit and at any time if clinically indicated including after the EOT visit. After the EOT visit if the patient has any persistent corneal findings, the assessments should continue every 8 weeks until resolution of findings, withdrawal of consent, death, or loss to follow up.

[q] Blood tests (evaluated by local laboratories) are listed in Appendix 4. Baseline hematology and blood chemistry test results must be obtained within 96 hours prior to enrollment. On subsequent dosing days, for both FPA144 or mFOLFOX6, hematology and blood chemistry results must be obtained within 72 hours prior to the start of dosing. Coagulation results need to be obtained at baseline, at Day 1 of Cycles 1 through 4 within 72 hours prior to dosing with FPA144, and at any time clinically indicated (eg, patients on anticoagulant therapy requiring close monitoring). Hematology, blood chemistry and coagulation samples are collected at the EOT visit.

[r] Serum ß-hCG (evaluated by local laboratories) will be performed on all women of childbearing potential at Screening within 96 hours prior to enrollment and when clinically indicated. If serum B-hCG test is performed at Screening and is not within 96 hours prior to enrollment, the test must be repeated to confirm the patient is not pregnant. Urine pregnancy tests and results are required on Day 1 for subsequent odd cycles (every other cycle) for both FPA144 and mFOLFOX6 prior to dosing. Urine pregnancy tests are also required at EOT.

[s] Includes protein, glucose, blood, pH, and ketones on Day 1 for both FPA144 and mFOLFOX6 of odd cycles (every other cycle). If findings are clinically significant, a microscopic evaluation will be performed per institutional standard.

[t] Blood samples for PK analysis. Refer to Appendix 6 for collection times.

[u] Blood samples for anti-FPA144 antibodies. Refer to Appendix 6 for collection times.

[v] Blood samples for exploratory pharmacodynamic biomarker analysis of the FGFR pathway: refer to Appendix 6 for collection times.

[w] FPA144 is administered Q2W as a 30-minute infusion starting C1D1. The first 3 doses (cycles) of FPA144 should be administered Q2W (±3 days) regardless of delays in mFOLFOX6 treatment. After the first 3 doses, FPA144 may be delayed up to 7 days to be synchronized with administered mFOLFOX6.

[x] FPA144 is administered only for Cohort 2 at Cycle 1 Day 8; subsequent cycles for Cohort 2 do not receive Day 8 study treatment. No other dose levels receive a Cycle 1 Day 8 FPA144 administration.

[y] mFOLFOX6 is administered Q2W at least 30 minutes after FPA144 as a continuous IV infusion over approximately 48 hours, starting on Day 1 and completing on Day 3.

[z] AE collection begins after signing of the ICF for Screening. Events reported prior to the first on-study infusion will be considered pretreatment events and reported on the Medical History page of the eCRF, unless they directly correlate to a study-related procedure. Adverse event reporting will continue until completion of the EOT visit or 28 days after the last dose of study treatment. SAE reporting will initiate at study enrollment unless the event is related to a study procedure. All treatment-related SAEs will be monitored until the event has resolved or stabilized, any abnormal laboratory values have returned to baseline or stabilized at a level acceptable to the investigator and Sponsor, there is a satisfactory explanation for the changes observed, or the patient is lost to follow-up. Serious AEs occurring after the EOT visit and ophthalmological events of any grade occurring up to 100 days after EOT visit should be reported to the Sponsor if the investigator considers there is a causal relationship with the study treatment.

APPENDIX 3: SCHEDULE OF ASSESSMENTS: RANDOMIZED, PLACEBO-CONTROLLED PORTION (PHASE 2)

Procedure[a]	Pre-Screening	Screening	Study Treatment: Cycles 1 and 2			Study Treatment: Cycle 3, Cycle 4 and Subsequent Cycles					EOT[e]	Follow-up Scans Follow-up[ff]	Long Term Follow Up[g]
		Day -28 to Day 0	Cycle 1 Day 1	Cycle 1 Day 8	Cycle 2 Day 1	Cycle 3[b] Day 1	Cycle 4[c] Day 1	Clinical Fixed Time Points Assessments[d]	As Clinically Indicated	Other Time Points			
		Week 0	Week 1	Week 2	Week 3	≥Week 4							
Pre-Screening Informed Consent[h]	X												
IHC Analysis of FGFR2b Overexpression[i]	X												
Sample for Blood-Based (ctDNA) Assay[j]	X												
Informed Consent[k]		X											
Review/Confirm Eligibility Criteria		X											
Medical/Oncology and Medication History		X	X										
Demography/Baseline Characteristics		X											
Physical Examination[l]		X[l]	X	X	X	X	X				X	X	
ECOG Performance Status[n]		X				X[n]				X[n]	X		
Patient-Reported Outcomes (EQ-5D-5L and the EORTC QLQ-C30)[o]		X					X[o]			X[o]	X		
Vital Signs[p]		X	X	X	X	X	X		X		X	X	
12-lead ECG[q]		X							X		X		
Comprehensive Ophthalmologic Examination (including Slit Lamp) [r]		X						X[d,r]	X	X[r]	X		

APPENDIX 3: SCHEDULE OF ASSESSMENTS: RANDOMIZED, PLACEBO-CONTROLLED PORTION (PHASE 2) (CONTD)

Procedure[a]	Pre-Screening	Screening	Study Treatment: Cycles 1 and 2			Study Treatment: Cycle 3, Cycle 4 and Subsequent Cycles					EOT[e]	Follow-up Scans Follow-up[ff]	Long Term Follow Up[g]
		Day -28 to Day 0	Cycle 1 Day 1	Cycle 1 Day 8	Cycle 2 Day 1	Cycle 3[b] Day 1	Cycle 4[c] Day 1	Clinical Fixed Time Points Assessments[d]	As Clinically Indicated	Other Time Points			
		Week 0	Week 1	Week 2	Week 3	≥Week 4							
Slit Lamp Examination[t]								X[d,t]		X[t]			
Clinical Safety Laboratory Sampling[u]		X	X[u]	X	X[u]	X[u]	X[u]		X		X		
Pregnancy Test[v]		X[v]	X[v]			X[v]			X	X[v]	X		
Urinalysis[w]		X	X			X[w]			X	X[w]	X		
Radiological/Tumor Assessment[x]		X						X[ff]		X[x]	X[s]	X	
Randomization[y]		X											
Survival Assessment													X
Immunogenicity Sampling[z]			X			X				X[z]	X		
PK Samples[aa]			X			X				X[aa]	X		
mFOLFOX6 Administration[bb]			X		X	X	X						
FPA144 Administration[m,y,cc]			X	X[f]	X	X	X						
Adverse Events[dd]		X[dd]	X[dd]	X ----- X								X[dd]	X[dd]
Concomitant Medications[ee]		X	X	X——X								X	X[ee]

A cycle of treatment is 2 weeks

[a] Unless specified, procedure is to be completed within ± 72 hours of scheduled time point and to be synchronized with the study treatment administration day.

[b] And subsequent odd cycles from Cycle 3 (except for PK, immunogenicity, and pharmacodynamic blood sample collections, which are described in Appendix 7).

[c] And subsequent even cycles from Cycle 4 (except for PK, immunogenicity, and pharmacodynamic blood sample collections, which are described in Appendix 7).

[d] Clinical Fixed Time Point Assessments should be completed as originally scheduled from C1D1 regardless of dosing days or dose delays. Refer to Appendix 5 for collection days.

[e] EOT assessments should be performed 28 (+14) days following the last study treatment administration. In Phase 2, after discontinuation of study treatment for reasons other than disease progression or withdrawal of consent, tumor assessments will continue until the patient initiates additional anticancer therapy or progresses.

[f] One dose of FPA144 is administered at 7.5 mg/kg on Cycle 1 Day 8 only; subsequent cycles do not receive Day 8 study treatment. For all other dose levels, no FPA144 administration on Cycle 1 Day 8.

[g] Patients will complete LTFU for survival approximately every 3 months ± 1 month after the EOT visit until up to 24 months after the last patient is enrolled in the study, or until death, loss to follow-up, withdrawal of consent or study termination by the Sponsor (whichever occurs first) (refer to Section 6.19).

[h] Informed consent must be obtained prior to obtaining blood for ctDNA and provision of tumor sample (refer to Section 1.8)

[i] Provision of archival tissue (or fresh biopsy if archival tissue is not available) for pre-screening FGFR2b overexpression testing by IHC is required. FGFR2 positive status by IHC or ctDNA testing methods is required for enrollment in Phase 2.

[j] Sample for blood-based biopsy (ctDNA) assay at Pre-Screening for prospective analysis of *FGFR2* gene amplification (refer to Appendix 7). FGFR2 positive status by IHC or ctDNA testing methods is required for enrollment in Phase 2.

[k] Written, signed informed consent must be collected prior to study related screening procedures. The most recent IRB/EC approved ICF must be signed. Patients who have fully consented to participation in the study will undergo screening assessments within 28 days prior to enrollment.

[l] Complete physical examination including height and weight will be measured at screening only. Limited physical examinations should be conducted, including weight and examination of the oropharynx, thereafter.

[m] After Cycle 1, the FPA144 dose should be recalculated only if the weight changes > 10% from the C1D1 weight. If the dose is recalculated due to a >10% weight change from C1D1, the weight used for the recalculated dose should function as the new baseline for subsequent evaluation of dose recalculations. Doses should be rounded to the nearest 10 mg.

[n] ECOG Performance Status will be assessed at Screening, Cycle 3 Day 1 and Day 1 of every other subsequent cycle (odd cycles), and at the EOT visit.

[o] The EQ-5D-5L and the EORTC QLQ-C30 will be collected at Screening (within 28 days of C1D1), prior to dosing on Cycle 4 Day 1, every 8 weeks until EOT, and at the EOT visit.

[p] Vital signs (blood pressure, heart rate, respiratory rate, and temperature) are to be measured, after 5 minutes of rest, at one time at the Screening visit, EOT and as clinically indicated. On C1D1, vital sign measurements should be at the following time points: Pre-dose, and 0.5, 1, 2, and 4 hours from the start of FPA144 infusion. On subsequent dosing days, vital sign measurements should be measured at the following time points: Predose and at 0.5, 1, and 2 hours from the start of FPA144 infusion.

[q] 12-lead ECG after patient rests for 5 minutes prior to recording.

[r] Comprehensive ophthalmologic examinations (conducted at Screening, at 8 weeks from C1D1 (±7 days), and at the EOT visit only (±7 days), include fundoscopic and slit lamp examination (with fluorescein staining score form), ocular coherence tomography (OCT), visual acuity, completion of fluorescein staining score form, determination of intraocular pressure, and review of ocular/visual symptoms. The comprehensive ophthalmologic examination will be repeated at any point if clinically indicated when changes in visual acuity or visual symptoms are reported by patients up to 100 days after the last dose of

FPA144. After the EOT visit if the patient has any persistent ophthalmologic findings, the assessments should continue every 8 weeks until resolution of findings, withdrawal of consent, death, or loss to follow up. All abnormal OCT examinations should be submitted to the Sponsor.

[s] This scan can be omitted if the last scan was performed < 8 weeks prior to EOT visit or if tumor progression was previously determined.

[t] Slit lamp examinations (with completion of fluorescein staining score form), should be conducted for all patients starting at Week 16 from C1D1 then every 8 weeks through EOT visit and at any time if clinically indicated including after the EOT visit. After the EOT visit if the patient has any persistent corneal findings, the assessments should continue every 8 weeks until resolution of findings, withdrawal of consent, death, or loss to follow up.

[u] Blood tests (evaluated by local laboratories) are listed in Appendix 4. Screening hematology and blood chemistry test results must be obtained within 96 hours of enrollment. On dosing days for both FPA144/placebo and mFOLFOX6, hematology and blood chemistry results must be obtained within 72 hours prior to dosing. Coagulation results need to be obtained at Screening, at Day 1 of Cycles 1 through 4 within 72 hours prior to dosing with FPA144, and at any time clinically indicated (eg, patients on anticoagulant therapy requiring close monitoring). Hematology, blood chemistry and coagulation samples are collected at EOT.

[v] Serum ß-hCG (evaluated by local laboratories) will be performed on all women of childbearing potential at Screening within 96 hours prior to enrollment and when clinically indicated. If serum B-hCG test is performed at Screening and is not within 96 hours prior to enrollment, the test must be repeated to confirm the patient is not pregnant. Urine pregnancy tests and results are required on Day 1 for subsequent odd cycles (every other cycle) for both FPA144/placebo and mFOLFOX6 prior to dosing. Urine pregnancy tests are also required at EOT.

[w] Includes protein, glucose, blood, pH, and ketones on Screening, C1D1, dosing days of odd cycles (every other cycle), and EOT. If findings are clinically significant, a microscopic evaluation will be performed per institutional standard.

[x] Radiological/tumor assessments will be performed within 28 days (+3 days) prior to start of treatment (C1D1) and including clinical examination and appropriate imaging techniques (preferably CT scans with appropriate slice thickness per RECIST v1.1; MRIs are acceptable); imaging performed as standard of care that includes imaging of chest, abdomen and pelvis may be used if it has been performed within 28 days (+3 days) of treatment (C1D1). Tumor scans will be performed at Screening (within 28 days [+3 days] of Cycle 1 Day 1), then every 8 weeks from Cycle 1 Day 1 (± 7 days) until 12 months and then every 12 weeks (± 14 days) thereafter. In Phase 2, after discontinuation of study treatment for reasons other than disease progression or withdrawal of consent, tumor assessments will continue until radiographic progression or the initiation of additional anticancer therapy.

[y] Patients must initiate the first administration of study treatment within 3 days of enrollment.

[z] Blood samples for anti-FPA144 antibodies. Refer to Appendix 7 for collection times

[aa] Blood samples for PK analysis. Refer to Appendix 7 for specific collection times.

[bb] mFOLFOX6 is administered Q2W as a continuous IV infusion over approximately 48 hours, starting on Day 1 and completing on Day 3 (if mFOLFOX6 is delayed secondary to toxicity, vital signs and clinical laboratory tests need to be performed within 72 hours of its administration).

[cc] C1D1 of FPA144 administered by IV infusion over 30 minutes (± 10 minutes) must occur within 3 days of being enrolled. FPA144 is administered Q2W starting C1D1. The first 3 doses (cycles) of FPA144 should be administered every 14 days (±3 days) regardless of delays in mFOLFOX6 treatment. After the first 3 doses, FPA144 may be delayed up to 7 days to be synchronized with administered mFOLFOX6.

[dd] AEs should not be reported during the pre-screening period unless they are related to a study procedure, as patients are not yet enrolled on the study at that time. AE collection begins following signing of the ICF for Screening. Events reported prior to the first on-study infusion will be considered pretreatment events and reported on the Medical History page of the eCRF, unless they directly correlate to a study-related procedure. Adverse event reporting will continue until completion of the EOT visit or 28 days after the last dose of study treatment. SAE reporting will initiate at study enrollment unless the event is related to a study procedure. All treatment-related SAEs experienced by a study patient will be monitored until the event has resolved or stabilized, any abnormal laboratory values have returned to baseline or stabilized at a level acceptable to the investigator and Sponsor, there is a satisfactory explanation for the changes observed, or the patient is lost to follow-up. SAEs occurring after the EOT visit and ophthalmological events of any grade occurring up to 100 days after EOT visit should be reported to the Sponsor by the investigator if the investigator considers there is a causal relationship with the study treatment.

[ee] For long term follow up, the only concomitant medication that needs to be collected is anticancer medication.

[ff] If a patient discontinues study treatment (FPA144 and/or mFOLFOX6) for reasons other than disease progression or withdrawal of consent, they will have follow-up visits and continue to undergo tumor assessments according to the protocol schedule until radiographic progression or the initiation of additional anticancer therapy (at which point the patient would begin the long term follow up for survival).

APPENDIX 4: LABORATORY EVALUATIONS

The following laboratory parameters will be determined in accordance with the Schedule of Assessments and can be performed locally:

**Hematology:**	
Complete blood cell (CBC) with differential:	
white blood cells (WBC)	platelets
absolute neutrophil count (ANC)	hemoglobin
neutrophils (%)	hematocrit
eosinophils (%)	red blood cells (RBC)
basophils (%)	
lymphocytes (%)	
monocytes (%)	
**Urinalysis:**	
Appearance, color, pH, specific gravity, ketones, protein, glucose, bilirubin, nitrite, urobilinogen, and occult blood	
If findings are clinically significant, a microscopic evaluation will be performed per institutional standard.	
**Clinical chemistry:**	
albumin	lactate dehydrogenase (LDH)
alkaline phosphatase	magnesium
ALT (SGPT)	phosphate
AST (SGOT)	potassium
blood urea nitrogen (BUN) or urea	sodium
calcium	total bilirubin
chloride	total cholesterol
creatinine	total protein
direct bilirubin	glucose
uric acid	
**Coagulation:**	
international normalized ratio (INR)	activated partial thromboplastin time (APTT)
**Serum and urine pregnancy testing:**	
In women of childbearing potential only.	

APPENDIX 5: CLINICAL FIXED TIME POINT ASSESSMENTS

Clinical Fixed Time Point Assessments should be completed as originally scheduled from Cycle 1 Day 1 (C1D1), regardless of dosing days or dose delays. Cycles are 2 weeks in length. Collection days are as follows:

**Procedure:**	**Study Treatment Period:**<br>**Cycle 3, Cycle 4 and Subsequent Cycles**
Comprehensive Ophthalmologic Examination (including Slit Lamp)	Should be conducted at:<br>Phase 1:<br>• At Screening (within 28 days of C1D1)<br>• At 4 weeks from C1D1<br>• At 8 weeks from C1D1 ±7 days thereafter<br>• At the EOT visit ±7 days<br>Phase 2:<br>• At Screening (within 28 days of C1D1)<br>• At 8 weeks from C1D1 ±7 days<br>• At the EOT visit ±7 days<br>For both Phase 1 and Phase 2, should include fundoscopic and slit lamp examination (with fluorescein staining score form), ocular coherence tomography (OCT), visual acuity, completion of fluorescein staining score form, determination of intraocular pressure, and review of ocular/visual symptoms.<br>The comprehensive ophthalmologic examination should be repeated at any time if clinically indicated when changes in visual acuity or visual symptoms are reported by patients up to 100 days after the last dose of FPA144. After the EOT visit if the patient has any persistent ophthalmologic findings, the assessments should continue every 8 weeks until resolution of findings, withdrawal of consent, death, or loss to follow up. All abnormal OCT examinations should be submitted to the Sponsor.
Slit Lamp Examination	Phase 1 and Phase 2:<br>Should be conducted at:<br>• Week 16 and then every 8 weeks through EOT visit<br>• at any time if clinically indicated, including after the EOT visit

APPENDIX 5: CLINICAL FIXED TIME POINT ASSESSMENTS (CONTD)

Procedure:	Study Treatment Period:<br>Cycle 3, Cycle 4 and Subsequent Cycles
Radiological/Tumor Assessment	Phase 2 only:<br>Should be conducted at:<br>• At Screening (within 28 days +3 days of treatment C1D1)<br>• Every 8 weeks from C1D1 ±7 days until 12 months and then every 12 weeks ±14 days thereafter. Scans should be obtained per schedule regardless of drug interruption or discontinuation.<br>• At the EOT visit ±7 days (this scan can be omitted if the last scan was performed < 8 weeks prior to EOT visit or if tumor progression was previously determined)<br>• *Scan follow-up visits, if applicable<br>Tumor assessments will be performed within 28 days (+3 days) prior to start of treatment (C1D1) and including clinical examination and appropriate imaging techniques (preferably CT scans with appropriate slice thickness per RECIST v1.1; MRIs are acceptable); imaging performed as standard of care that includes imaging of chest, abdomen and pelvis may be used if it has been performed within 28 days +3 days of treatment (C1D1).<br>• *After discontinuation of study treatment for reasons other than disease progression or withdrawal of consent, tumor assessments will continue until radiographic progression or the initiation of additional anticancer therapy.

APPENDIX 6: STUDY FLOWCHART FOR PHARMACOKINETIC, IMMUNOGENICITY, AND EXPLORATORY PHARMACODYNAMIC BIOMARKER BLOOD SAMPLE COLLECTIONS FOR PHASE 1

Study Cycle	Study Day	Time Point	Type of Sample
Cycle 1	Day 1	≤ 4 hours prior to infusion (predose)	ctDNA blood assay sample
			FPA144 PK (serum)
			ADA (serum)
			Blood samples for exploratory pharmacodynamic biomarker analysis of FGFR pathway
		15 (±10) minutes after end of infusion	FPA144 PK (serum)
		4 hours (±60 minutes) after end of infusion	FPA144 PK (serum)
	Day 3	48 hours (±6 hours) after end of infusion	FPA144 PK (serum)
			Blood samples for exploratory pharmacodynamic biomarker analysis of FGFR pathway
	Day 8	168 hours (±1 day) after end of infusion	FPA144 PK (serum)
	Day 8* (Cohort 2 patients only)	≤ 4 hours prior to infusion (predose)	FPA144 PK (serum)
		15 (±10) minutes after end of infusion	FPA144 PK (serum)
		4 hours (±60 minutes) after end of infusion	FPA144 PK (serum)
Cycle 2	Day 1	≤ 4 hours prior to infusion (predose)	FPA144 PK (serum)
			ADA (serum)
		15 (±10) minutes after end of infusion	FPA144 PK (serum)
	Day 3	48 hours (±6 hours) after end of infusion	FPA144 PK (serum)
Cycle 3	Day 1	≤ 4 hours prior to infusion (predose)	FPA144 PK (serum)
			ADA (serum)
			Blood samples for exploratory pharmacodynamic biomarker analysis of FGFR pathway
		15 (±10) minutes after end of infusion	FPA144 PK (serum)

APPENDIX 6: STUDY FLOWCHART FOR PHARMACOKINETIC, IMMUNOGENICITY, AND EXPLORATORY PHARMACODYNAMIC BIOMARKER BLOOD SAMPLE COLLECTIONS FOR PHASE 1 (CONTD)

Study Cycle	Study Day	Time Point	Type of Sample
Cycle 4	Day 1	≤ 4 hours prior to infusion (predose)	FPA144 PK (serum)
		15 (±10) minutes after end of infusion	Blood samples for exploratory pharmacodynamic biomarker analysis of FGFR pathway
			FPA144 PK (serum)
Cycle 5, 7, 9, 11	Day 1	≤ 4 hours prior to infusion (predose)	FPA144 PK (serum)
			ADA (serum) (Cycle 7)
			Blood samples for exploratory pharmacodynamic biomarker analysis of FGFR pathway
		15 (±10) minutes after end of infusion	FPA144 PK (serum)
Cycle 10	Day 1	15 (±10) minutes after end of infusion	FPA144 PK (serum)
	Day 3	48 hours (± 6 hours)	FPA144 PK (serum)
			Blood samples for exploratory pharmacodynamic biomarker analysis of FGFR pathway
	Day 1	≤ 4 hours prior to infusion (predose)	FPA144 PK (serum)
			ADA (serum)
Every 8 Cycles Starting from Cycle 15			Blood samples for exploratory pharmacodynamic biomarker analysis of FGFR pathway
End of Treatment Follow-up	Visit date	Single time point	FPA144 PK (serum)
			ADA (serum)
			Blood samples for exploratory pharmacodynamic biomarker analysis of FGFR pathway

* For Cohort 2 patients only

APPENDIX 7: STUDY FLOWCHART FOR FPA144 PHARMACOKINETIC AND IMMUNOGENICITY BLOOD SAMPLE COLLECTIONS FOR PHASE 2

Study Cycle	Study Day	Time Point	Type of Sample
Cycle 1	Day 1	≤ 4 hours prior to infusion (predose)	FPA144 PK (serum)
			ADA (serum)
		15 (±10) minutes after end of infusion	FPA144 PK (serum)
Cycle 3	Day 1	≤ 4 hours prior to infusion (predose)	FPA144 PK (serum)
			ADA (serum)
		15 (±10) minutes after end of infusion	FPA144 PK (serum)
Cycles 5, 9, and 17	Day 1	≤ 4 hours prior to infusion (predose)	FPA144 PK (serum)
			ADA (serum)
End of Treatment Follow-up	Visit date	Single time point	FPA144 PK (serum)
			ADA (serum)

APPENDIX 8: ECOG PERFORMANCE STATUS

Grade	Performance Status Criteria
0	Fully active, able to carry on all predisease activities without restriction.
1	Restricted in physically strenuous activity, but ambulatory and able to carry out work of a light sedentary nature (light housework, office work).
2	Ambulatory and capable of all self-care, but unable to carry out any work activities. Up and about more than 50% of waking hours.
3	Capable of only limited self-care, confined to bed or chair more than 50% of waking hours.
4	Completely disabled. Cannot carry on any self-care. Totally confined to bed or chair.

APPENDIX 9: FLUORESCEIN STAIN GRADING SYSTEMS

<table>
<tr><td>DEWS</td><td>DRY EYE: DIAGNOSTIC TEST TEMPLATE</td><td></td></tr>
<tr><td>RAPPORTEUR</td><td>A.J.Bron</td><td>21st Oct 2004</td></tr>
<tr><td>TEST</td><td>Grading staining: Oxford Schema</td><td></td></tr>
<tr><td>TO DIAGNOSE</td><td>The scheme is used to estimate surface damage in dry eye.</td><td>REFERENCES</td></tr>
<tr><td>VERSION of TEST</td><td>[V 1]</td><td></td></tr>
<tr><td>DESCRIPTION</td><td>Surface damage to the exposed eye, assessed by staining, is graded against standard charts.</td><td></td></tr>
<tr><td>NATURE of STUDY</td><td>N. A.</td><td></td></tr>
<tr><td>CONDUCT of TESTS</td><td>Grading Schema:
Staining is represented by punctate dots on a series of panels (A-E). Staining ranges from 0-5 for each panel and 0-15 for the total exposed inter-palpebral conjunctiva and cornea. The dots are ordered on a log scale
<table>
<tr><th>PANEL</th><th>GRADE</th><th>CRITERIA</th></tr>
<tr><td>A 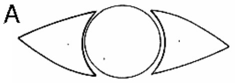</td><td>0</td><td>Equal to or less than panel A</td></tr>
<tr><td>B 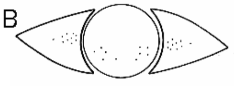</td><td>I</td><td>Equal to or less than panel B, greater than A</td></tr>
<tr><td>C 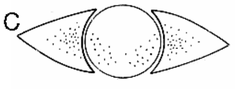</td><td>II</td><td>Equal to or less than panel C, greater than B</td></tr>
<tr><td>D 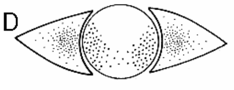</td><td>III</td><td>Equal to or less than panel D, greater than C</td></tr>
<tr><td>E 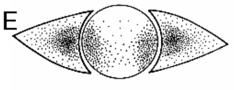</td><td>IV</td><td>Equal to or less than panel E, greater than D</td></tr>
<tr><td>>E</td><td>V</td><td>Greater than panel E</td></tr>
</table>
Conduct of Test:

Dye is instilled.
Slit-lamp is set (eg.16 magnification with x10 oculars with Haag-Streit).
Cornea: The upper eyelid is lifted slightly to grade the whole corneal surface,
Conjunctiva: To grade the temporal zone, the subject looks nasally; to grade the nasal zone the subject looks temporally.
(The upper and lower conjunctiva can also be
</td><td>Bron Evans Smith 2003.</td></tr>
</table>

APPENDIX 9: FLUORESCEIN STAIN GRADING SYSTEMS (CONTD)

graded).

Selection of dyes:

A list dyes and filters can be found in the original paper.

With fluorescein, staining must be graded as quickly as possible after instillation, since the dye then diffuses rapidly into the tissue and its high luminosity blurring the stain margin.

Staining after rose bengal or lissamine green, persists at high contrast and may therefore be observed for a considerable period. This is convenient for both grading and photography.

Fluorescein sodium

1. Quantified drop instillation

eg 2 µl of 2 % sterile fluorescein instilled into each conjunctival sac with a micro-pipette (using a sterile tip). In very dry eye, larger volumes risk the possibility of inadequate dilution into the fluorescent range.

2. Unquantified instillation – impregnated paper strips

This is a convenient approach in the clinic using the following method of application:

- A single drop of unit dose saline is instilled onto a fluorescein-impregnated strip.
- When the drop has saturated the impregnated tip, the excess is shaken into a waste bin with a sharp flick.
- The right lower lid is then pulled down and the strip is tapped onto the lower tarsal conjunctiva. A similar procedure is carried out on the left.

If too large a volume is delivered then the concentration in the tear film will be too high, and the tear film and staining pattern will be non-fluorescent.

3.Timing

The fluorescein break-up time (FBUT) is usually performed prior to grading. Since fluorescein diffuses rapidly into tissues, punctate staining blurs after a short period. It is therefore essential to assess staining rapidly, in sequence, in the right and then the left eye, so that the staining patterns observed are equally crisp.

If it is intended to photograph the staining pattern for grading, then photography should follow immediately after each instillation.

Exciter and Barrier Filters

The absorption peak of fluorescein sodium occurs between 465 - 490 nm and the emission peak between 520 - 530 nm A suggested filter pair for detection of fluorescein staining is a yellow, Kodak Wratten 12 barrier filter (transmitting above 495 nm) or an orange Wratten 15 filter (transmitting above 510 nm) in combination with a blue Wratten 47 or 47A exciter filter. The 47A shows greater transmittance than the Wratten 47 over the absorption range. The 'cobalt' filter of many slit-lamps is suitable to use with a Wratten 12 or 15

APPENDIX 9: FLUORESCEIN STAIN GRADING SYSTEMS (CONTD)

	barrier.<br><br>Where more light is required for photographic purposes, narrow band-pass, interference filters can be used.<br><br>The use of both exciter and barrier filters allows both the cornea and conjunctiva to be assessed using a single stain. This is a major advantage in clinical trials where it is otherwise customary to employ fluorescein to grade corneal staining and rose bengal or lissamine green to grade conjunctival staining.<br><br>**Disadvantages of Fluorescein Staining**<br>Blurred pattern if reading is delayed. Delay in photographing fluorescein staining results in blurred images of the staining pattern.<br><br>**Rose Bengal**<br>The intensity of rose bengal staining is dose dependent. If drop size or concentration is reduced to minimize stinging, the amount of staining is also reduced. Use of impregnated strips will give weaker staining than use of a full drop of 1% solution. Best results are achieved with, eg. 25 µl 1%, instilled into the conjunctival sac. Because rose bengal stings, instillation is best preceded by a topical anesthetic.<br><br>**Instillation Technique**<br>1) eg. A drop of Proxymetacaine is instilled into the conjunctival sac followed, after recovery, by;<br>2) A drop of rose bengal 1.0%. This is instilled onto the upper bulbar conjunctiva with the upper lid retracted and the patient looking down.<br>3) Since both anaesthetic and drop may stimulate reflex tearing, the test should follow measurement of the FBUT and of the Schirmer test. (Conjunctival staining due to insertion of the Schirmer paper can usually be distinguished from that due to dry eye disease).<br><br>Both eyes may be stained prior to grading, since there is no risk of the staining pattern in the first eye being obscured by the time the second eye is graded.<br><br>The cited paper gives advice about avoidance of overspill.<br><br>**Visibility**<br>Rose bengal staining on the conjunctiva shows up well against the sclera and may be enhanced using a red-free (green) light source. Corneal staining may show up well against a blue iris, but is difficult to see against a dark brown iris.<br><br>**Phototoxicity**<br>Photo-activation of rose bengal by sunlight increases post-instillation symptoms, especially in severe dry eye with heavy staining. This post-instillation pain can be minimised	

APPENDIX 9: FLUORESCEIN STAIN GRADING SYSTEMS (CONTD)

<table>
<tr><td></td><td>by liberal irrigation with normal saline at the end of the test.

Lissamine green stains the eye in a similar manner to rose bengal but is as well tolerated as fluorescein. Visibility and dose-dependency are the same as rose bengal and staining is persistant so that photography need not be performed immediately after instillation.
Lissamine green is available as impregnated strips or may be ordered as a pre-prepared solution. A 25 µl 1% drop will give more intense staining. Because the drop is well tolerated, no anaesthetic is required.

Visibility
As with rose bengal, lissamine green staining is easily visible on the conjunctiva. On the cornea, staining is seen well against a light blue iris background but is poorly visible against a dark brown iris background. For both rose bengal and lissamine green, because the dyes are poorly seen within the tear film, the dye in the tear film does not obscure the staining pattern. Also, since both dyes do not diffuse into the substantia propria of the conjunctiva, the staining pattern is retained for longer.

Visibility of staining may be enhanced using a white light source and a red barrier filter, to give a black pattern on a red ground. A suitable filter is a Hoya 25A, or a Kodak Wratten 92.</td><td></td></tr>
<tr><td>Web Video</td><td>Not available</td><td></td></tr>
<tr><td>Materials:</td><td>Oxford Grading Charts - [redacted]</td><td></td></tr>
<tr><td>Standardization</td><td>Nil additional</td><td></td></tr>
<tr><td>Variations of technique</td><td></td><td></td></tr>
<tr><td>Diagnostic value</td><td>No stats supplied.</td><td></td></tr>
<tr><td>Repeatability</td><td>A small intra-interobserver study was carried out in 1986 and was presented but not published:

Intra-observer study: This study asked two trained ophthalmologists to grade a series of standard slides, showing corneal and conjunctival fluorescein staining, on 2 separate occasions. [note: -this study is only relevant to grading photographic records not patients.]

<table><tr><td colspan="3">Intra-observer κ for grading photographs of staining, using the Oxford scheme. Two observers.</td></tr><tr><td></td><td>Cornea</td><td>Conjunctiva</td></tr><tr><td>Observer 1</td><td>0.86</td><td>0.69</td></tr><tr><td>Observer 2</td><td>0.65</td><td>0.83</td></tr></table>
Not that values are in the good to excellent range.

Inter-observer study: In this study, the same 2 observers</td><td>Hardman Lea et al. 1986 AER abstract.</td></tr>
</table>

APPENDIX 9: FLUORESCEIN STAIN GRADING SYSTEMS (CONTD)

<table>
<tr><td></td><td>graded fluorescein staining (blue exciter; yellow filter) in 13 dry eye patients at an interval within 2-3 weeks.

Inter-observer κ for grading patients with dry eye, using the Oxford scheme. Two observers. Fluorescein; bengal rose
Observer 1 v 2 | Cornea | Conjunctiva
Fluorescein | 0.88 | 0.48
Bengal rose | 0.87 | 0.54

It is of interest that observations are in the excellent category for cornea, with either stain and in the fair category for conjunctiva.</td><td></td></tr>
<tr><td>Sensitivity</td><td>(true positives) [-]</td><td></td></tr>
<tr><td>Specificity</td><td>(100 – false positives) [-]</td><td></td></tr>
</table>

References:

Bron A, Evans VE, Smith JA. (2003). Grading of corneal and conjunctival staining in the context of other dry eye tests. *Cornea* 22(7): 640-50.

(Bron 2003)

STATISTICAL ANALYSIS PLAN

**Study Title:**	FIGHT: A Phase 2 Randomized, Double-Blind, Controlled Study Evaluating FPA144 and Modified FOLFOX6 in Patients with Previously Untreated Advanced Gastric and Gastroesophageal Cancer: Phase 2 Preceded by Dose-Finding in Phase 1
**Name of Test Drug:**	FPA144
**Protocol Number:**	FPA144-004
**Protocol Version (Date):**	Amendment 3 (5 June 2020)
**Analysis Type:**	Final Analysis
**Analysis Plan Version:**	1.0
**Analysis Plan Date:**	20 October 2020
**Analysis Plan Author:**	[redacted]

Approved by:

FivePrime

Lead Biostatistician (Print Name)	Signature	Date
Head of Biometrics (Print Name)	Signature	Date
Clinical Pharmacology Lead (Print Name)	Signature	Date
Clinical Team Lead (Print Name)	Signature	Date
Head of Clinical Development (Print Name)	Signature	Date

TABLE OF CONTENTS

1 Background and Rationale 8
1.1 Study Design 8
1.1.1 Treatment Assignment 9
1.1.2 Blinding and Unblinding 9
1.2 Study Objectives 9
1.2.1 Phase 1 Objectives 9
1.2.2 Phase 2 Objectives 10
1.3 Study Endpoints 11
1.3.1 Phase 1 Endpoints 11
1.3.2 Phase 2 Endpoints 11
1.4 Sample Size and Power 12
2 Type of Planned Analyses 13
2.1 Interim Analyses 13
2.2 Final Analysis 13
2.3 Follow-up Analysis 13
3 General Considerations 14
3.1 Analysis Sets 14
3.1.1 Intent-to-treat (ITT) Analysis Set 14
3.1.2 Efficacy-Evaluable Analysis Set 14
3.1.3 Safety Analysis Set 15
3.1.4 DLT-Evaluable Analysis Set 15
3.1.5 PK-Evaluable Analysis Set 15
3.1.6 ADA-Evaluable Analysis Set 15
3.2 Subject Grouping 15
3.3 Stratification Factors 16
3.4 Examination of Subject Subgroups 16
3.5 Multiple Comparisons 17
3.6 Missing Data and Outliers 17
3.6.1 Missing Data 17
3.6.2 Outliers 17
3.7 Data Handling Conventions and Transformations 17
3.8 Analysis Visit Windows 18
3.8.1 Definition of Study Day 18

3.8.2 Analysis Visit Windows 18
4 Subject Disposition 20
4.1 Subject Enrollment and Disposition 20
4.2 Extent of Exposure and Adherence 20
5 Demographics and Baseline Characteristics 22
5.1 Demographics 22
5.2 Other Baseline Disease Characteristics 22
5.3 Medical History 22
5.4 Prior and Concomitant Medications 23
5.5 Prior Anticancer Therapy 23
6 Efficacy Analyses 24
6.1 Efficacy Endpoints in Phase 1 24
6.2 Primary Efficacy Endpoint in Phase 2 24
6.2.1 Definition of the Primary Efficacy Endpoint 24
6.2.2 Statistical Hypothesis for the Primary Efficacy Endpoint 25
6.2.3 Analysis of the Primary Efficacy Endpoint 25
6.2.4 Sensitivity Analysis of the Primary Efficacy Endpoint 25
6.2.5 Exploratory Analysis of the Primary Efficacy Endpoint 26
6.3 Secondary Efficacy Endpoints in Phase 2 27
6.3.1 Definition of the Secondary efficacy Endpoints 27
6.3.2 Analysis of the Secondary Efficacy Endpoints 27
6.4 Exploratory Efficacy Endpoints in Phase 2 29
6.4.1 Duration of Response 29
6.4.2 Time to Response 30
6.4.3 Change in Tumor Size 30
6.4.4 Patient-Reported Outcome Assessments 30
6.4.5 ECOG PS 31
6.5 Changes from Protocol-Specified Efficacy Analysis 31
7 Safety analyses 32
7.1 Adverse Events and Deaths 32
7.1.1 Adverse Event Dictionary 32
7.1.2 Adverse Event Severity 32
7.1.3 Relationship of Adverse Event to Study Drug 32
7.1.4 Serious Adverse Events 32
7.1.5 Treatment-Emergent Adverse Events 32

7.1.6 Summary of Adverse Events and Deaths 33
7.1.7 Adverse Events of Special Interest 34
7.1.8 Dose-Limiting Toxicity 35
7.2 Clinical Laboratory Evaluations 35
7.3 Body Weight and Vital Signs 36
7.4 Electrocardiograms 36
7.5 Other Safety Measures 36
8 Pharmacokinetic and immunogenicity Analyses 38
8.1 Bioanalytical Methods 38
8.2 PK Analysis 38
8.2.1 PK Sample Collection 38
8.2.2 Estimation of Pharmacokinetic Parameters 38
8.2.3 Assessment of Dose-Proportionality for Phase 1 40
8.2.4 Assessment of Steady-State Attainment for both Phase 1 and Phase 2 40
8.2.5 Assessment of Immunogenicity Impact on Serum FPA144 Concentration and Pharmacokinetic Parameters 41
8.2.6 Summary of Serum Concentration data and Pharmacokinetic Parameters 41
8.2.7 Quality Control Methods for PK data Analysis 42
8.3 Immunogenicity Analysis 42
8.3.1 Immunogenicity Samples Collection 42
8.3.2 Summary of Immunogenicity Results 42
9 References 43
10 Software 44

LIST OF FIGURES

Figure 1: Phase 1/2 Study Schema 8
Figure 2: Phase 2 Patient Timeline 9

LIST OF Abbreviations

ADA	anti-drug antibody
AE	adverse event
AESI	adverse event of special interest
ATC	Anatomical Therapeutic Chemical classification
AUC	area under the observed concentration-time curve
BMI	body mass index
BOR	best overall response
C_{max}	maximum observed concentration determined from the observed concentration values post first dose
C_{trough}	minimum observed concentration determined from the observed concentration values
CI	confidence interval
CL	total body clearance
CMH method	Cochran-Mantel-Haenszel method
CR	complete response
CSR	Clinical Study Report
CVT	computed tomography
DLT	dose-limited toxicity
DOR	duration of response
ECG	electrocardiogram
ECOG PS	Eastern Cooperative Oncology Group Performance Status
EORTC QLQ-C30	European Organization for Research and Treatment of Cancer Quality of Life Questionnaire Version 3.0
EOT	end of treatment
EQ-5D-5L	EuroQOL-5D-5L
GC	gastroesophageal cancer
GI	gastrointestinal tumors
GLP	Good Laboratory Practices
HR	hazard ratio
ITT	intent-to-treat
IXRS	interactive voice or web response system
LTFU	long-term follow-up
MedDRA	Medical Dictionary for Regulatory Activities
NCA	non-compartmental analysis method
NCI-CTCAE	National Cancer Institute Common Toxicity Criteria for Adverse Events
NE	not evaluable
OECD	Organization for Economic Cooperation and Development
ORR	objective response rate
OS	overall survival
PFS	progression-free survival
PK	pharmacokinetic
PR	partial response

PROs	patient-reported outcomes
PT	preferred term
Q1, Q3	first quantile, third quantile
Q2W	every 2 weeks
QOL	quality of life
RECIST v1.1	New Response Evaluation Criteria in Solid Tumors (version 1.1)
SAE	serious adverse event
SAP	Statistical Analysis Plan
SE	standard error
SMQ	standardised MedDRA queries
SOC	system organ class
StD	standard deviation
$t_{1/2}$	terminal half-life
TEAE	treatment-emergent adverse event
TFLs	Tables, Figures, and Listings
TTR	time to response
WHODD	World Health Organization Drug Dictionary

1 BACKGROUND AND RATIONALE

This statistical analysis plan (SAP) describes the statistical analysis methods and data presentations to be used in tables, figures and listings (TFLs) of the final analysis in the clinical study report (CSR) for study FPA144-004. This SAP is based on the study protocol Amendment 3 dated 05 June 2020. The SAP will be finalized prior to data finalization for the final analysis (Section 1.5.1).

1.1 Study Design

This is a double-blind, randomized, controlled, multicenter Phase 1/2 study to evaluate the safety, tolerability, efficacy, pharmacokinetic (PK), of FPA144 + mFOLFOX6 versus placebo + mFOLFOX6. This study includes a Phase 1 safety run-in portion and a Phase 2 portion. Patients may enroll in either Phase 1 or Phase 2 but may not enroll in both phases of the study.

The Phase 1 safety run-in is an open-label dose-escalation of FPA144 + mFOLFOX6 in patients with gastrointestinal (GI) tumors (not FGFR2 selected). The Phase 2 portion of the study (to follow the Phase 1 safety run-in) is a global, randomized, double-blind, controlled, study to evaluate the efficacy of FPA144 + mFOLFOX6 versus placebo + mFOLFOX6 in patients with FGFR2-selected gastroesophageal cancer (GC), as determined by prospective IHC demonstrating FGFR2b overexpression and/or a ctDNA blood assay demonstrating FGFR2 gene amplification.

The study schema (Phase 1/2) is shown in Figure 1. Patient timelines are shown in Figure 2 (Phase 2).

Figure 1: Phase 1/2 Study Schema

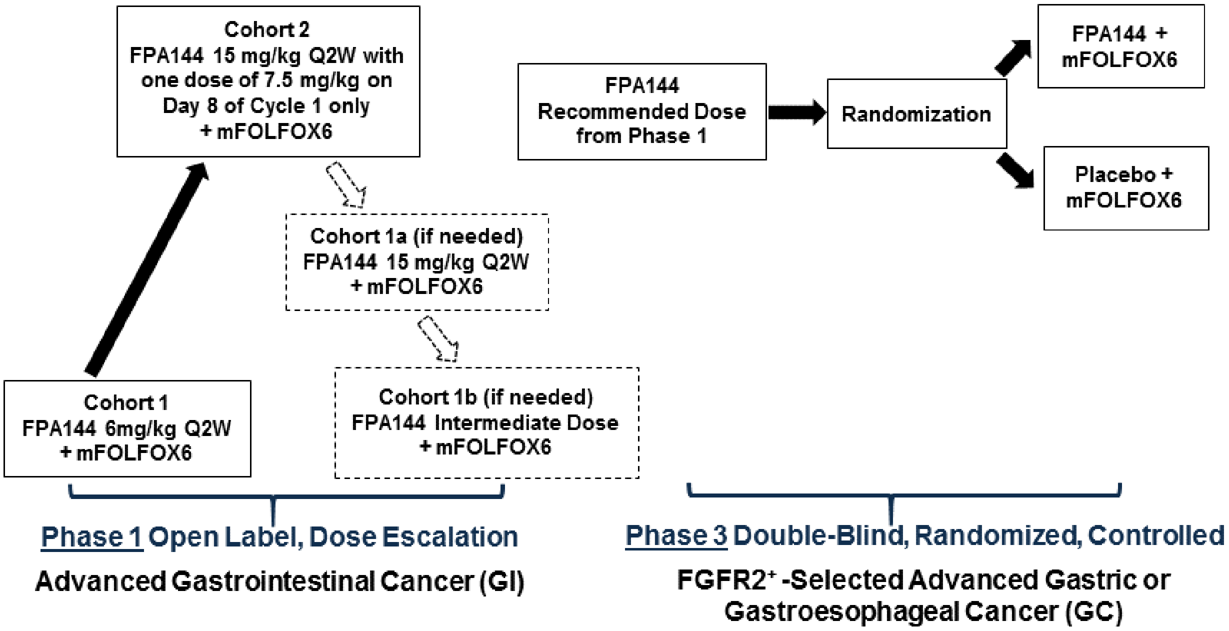

Abbreviations: Q2W = every 2 weeks.

Figure 2: Phase 2 Patient Timeline

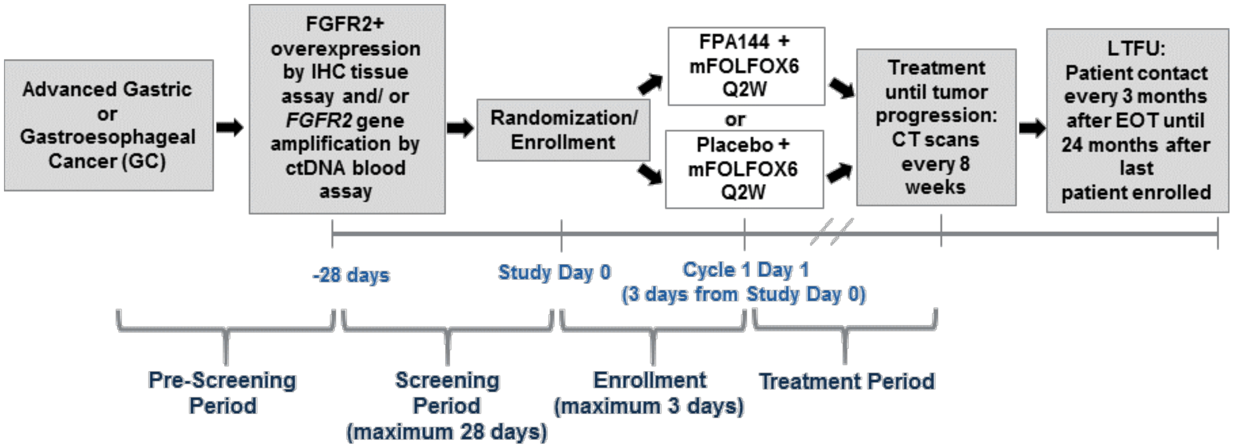

Abbreviations: CVT = computed tomography; EOT = end of treatment; LTFU = long term follow up; Q2W = every 2 weeks.

1.1.1 Treatment Assignment

Based on an assessment of Phase 1 overall safety, tolerability, and PK data of FPA144 in combination with mFOLFOX6 by the CRC, the dose of 15 mg/kg Q2W with an additional dose of 7.5 mg/kg on Cycle 1 Day 8 will be used for the Phase 2 portion of the trial.

In Phase 2, approximately 155 FGFR2-selected GC patients will be randomized 1:1 to be treated with either FPA144 + mFOLFOX6 or placebo + mFOLFOX6 in 2-week cycles.

1.1.2 Blinding and Unblinding

The Phase 2 portion of this study is double-blind. Placebo will be matched to FPA144. Treatment codes should not be broken except in emergency situations. With the exception of unblinding for the analysis, all individuals involved in the conduct of the study (eg, all site staff and participants, monitoring personnel, Sponsor personnel) will remain blinded to randomized treatment assignment.

The investigator should document and provide an explanation for any premature unblinding (eg, accidental unblinding or unblinding because of a serious adverse event).

1.2 Study Objectives

1.2.1 Phase 1 Objectives

The primary objective of the Phase 1 portion is to determine the RD of FPA144 combined with a fixed dose of mFOLFOX6 (hereinafter referred to as FPA144 + mFOLFOX6) in patients with advanced GI tumors.

The secondary objectives are:

- To evaluate the safety and tolerability of FPA144 + mFOLFOX6 in patients with GI tumors
- To characterize the PK profile of FPA144 in the presence of mFOLFOX6 in patients with GI tumors
- To characterize the immunogenicity of FPA144

The exploratory objective is to characterize the pharmacodynamic profile of FPA144 + mFOLFOX6 in patients with GI tumors.

1.2.2 Phase 2 Objectives

The primary objective of the Phase 2 portion is to compare investigator-assessed progression-free survival (PFS) in patients with FGFR2-selected GC treated with FPA144 + mFOLFOX6 to those treated with placebo combined with mFOLFOX6 (hereafter referred to as placebo + mFOLFOX6).

The secondary objective is to compare the following in patients with FGFR2-selected GC treated with FPA144 + mFOLFOX6 to those treated with placebo + mFOLFOX6:

- Overall survival (OS)
- Investigator-assessed objective response rate (ORR)
- Safety and tolerability

The exploratory objective is to compare the following in patients with FGFR2-selected GC treated with FPA144 + mFOLFOX6 to those treated with placebo + mFOLFOX6:

- Duration of response (DOR)
- Patient-reported outcomes (PROs) and quality of life (QOL) outcomes until investigator-assessed disease progression
- To explore the association between FGFR2 status (in tumor tissue and/or blood) with clinical outcome
- To explore the concordance between FGFR2b overexpression in tumor tissue and *FGFR2* gene amplification in blood

To characterize the following:

- PK profile of FPA144 in the presence of mFOLFOX6 in patients with FGFR2-selected GC
- Immunogenicty of FPA144

1.3 Study Endpoints

1.3.1 Phase 1 Endpoints

The primary endpoint of Phase 1 portion is the incidence of Grade 2 or higher adverse events (AEs) assessed as related to FPA144 by the investigator and the incidence of clinical laboratory abnormalities defined as Dose-Limited Toxicity (DLT).

The secondary endpoints are:

- The incidence of AEs, clinical laboratory abnormalities, corneal and retinal findings, and electrocardiogram (ECG) abnormalities
- PK parameters of FPA144, such as AUC, C_{max}, C_{trough}, CL, $t_{1/2}$, volume of distribution, the time to achieve steady state, dose-linearity, and accumulation ratio
- Incidence of treatment-emergent anti-FPA144 antibody response

The exploratory endpoint is the pharmacodynamic parameters, including exploratory pharmacodynamic biomarker analyses of the FGFR pathway in blood.

1.3.2 Phase 2 Endpoints

The primary endpoint of the Phase 2 portion is PFS, defined as time from randomization until the date of disease progression based on investigator assessment per RECIST v1.1 or death from any cause, whichever comes first.

The secondary endpoints are:

- OS, defined as time from randomization until death from any cause
- ORR, defined as the proportion of patients with partial or complete response in all enrolled patients based on investigator assessment of tumor lesions per RECIST v1.1
- Incidence of AEs, clinical laboratory abnormalities, corneal and retinal findings, and ECG abnormalities

The exploratory endpoints are:

- DOR limited to patients who are responders to treatment, as determined by the investigator per RECIST v1.1, and defined as the time of first response to progression or death from any cause, whichever comes first
- Change from baseline in functional outcomes as measured by EuroQOL-5D-5L (EQ-5D-5L) and the European Organization for Research and Treatment of Cancer Quality of Life Questionnaire Version 3.0 (EORTC QLQ-C30)

- The correlation between identified FGFR2 status in tumor tissue and/or ctDNA blood assay, as determined by IHC and blood-based molecular diagnostic assay, with OS, PFS, and objective response per RECIST v1.1
- The correlation between identified FGFR2b overexpression in tumor tissue by IHC and *FGFR2* gene amplification as determined by ctDNA blood assay
- PK parameters, such as C_{max} and C_{trough} of FPA144 in combination with mFOLFOX6
- Incidence of treatment-emergent anti-FPA144 antibody response

1.4 Sample Size and Power

The Phase 1 portion planned to enroll approximately 9 to 21 patients depending on incidence of DLTs; this allows for evaluation of safety, PK, and pharmacodynamics at 1 or more dose levels.

The Phase 2 portion is designed to assess the hazard ratio (HR) for PFS for the FPA144 + mFOLFOX6 compared with placebo + mFOLFOX6. It is planned to observe at least 84 PFS events in order to achieve 71% power to detect an HR of 0.67 for PFS at a 1-sided significance level of 0.1. Assuming an exponential distribution, this corresponds approximately to an 50% increase in median PFS (e.g. from 5 months to 7.5 months). Statistical significance (at 1-sided alpha of 0.1) for PFS will occur with an observed HR=0.756, corresponding approximately to a 32.3% increase in observed median PFS (e.g. from 5 months to 6.6 months).

2 TYPE OF PLANNED ANALYSES

2.1 Interim Analyses

No formal interim efficacy analysis, which may lead to early termination for efficacy or futility, is planned in the study.

2.2 Final Analysis

The final efficacy analysis will be conducted after at least 84 PFS events are observed. It is expected that this number of PFS events will occur approximately 11 months after the last patient is enrolled. Once outstanding data queries have been resolved, the database will be cleaned and finalized, and the final analysis of the data will be performed.

2.3 Follow-up Analysis

After the final analysis, additional supplemental analyses of efficacy and safety may be performed for long-term efficacy (eg, overall survival) and follow-up safety assessments.

3 GENERAL CONSIDERATIONS

All statistical tabulations and analyses will be done using SAS®, Version 9.3 or higher.

Unless otherwise noted, continuous variables will be summarized using the number of subjects (n), mean, standard error (SE) or standard deviation (StD), median, minimum, and maximum; categorical variables will be summarized using the number and percentage of subjects in each category.

By-subject listings will be presented for all subjects in the Safety Analysis Set (for Phase 1) or Intent-to-Treat (ITT) Analysis Set (for Phase 2) and sorted by subject ID number, visit date, and time (if applicable). Data collected on log forms, such as AEs, will be presented in chronological order within the subject. The treatment group to which subjects were enrolled/randomized will be used in the listings. Age, sex at birth, race, and ethnicity will be included in the listings, as space permits.

The summaries of the efficacy data will be presented by treatment group. Unless otherwise specified, non-efficacy analyses will be descriptive and will be presented by treatment group and overall.

Unless stated otherwise, Phase 1 and Phase 2 portions of the study will be summarized and listed separately.

3.1 Analysis Sets

3.1.1 Intent-to-treat (ITT) Analysis Set

The ITT Analysis Set is defined for Phase 2 portion of the study. It includes all subjects who were randomized in the study.

The ITT Analysis Set will be used in the summary of subject disposition, demographics and baseline characteristics and the primary analyses for efficacy endpoints.

3.1.2 Efficacy-Evaluable Analysis Set

The Efficacy-Evaluable Analysis Set is defined for Phase 2 portion of the study. It includes all randomized subjects who met key eligibility criteria and received at least 1 dose of study drug (FPA144 + mFOLFOX6 or placebo + mFOLFOX6), had at least 1 postbaseline evaluable tumor assessment, had ≥75% exposure intensity of FPA144/placebo, with no major protocol deviations that could introduce bias in efficacy analysis. Major protocol deviations that could bias efficacy analysis will be assessed and determined on a case-by-case basis prior to unblinding.

The Efficacy-Evaluable Analysis Set is the secondary analysis set for efficacy analyses.

3.1.3 Safety Analysis Set

Phase 1

The Safety Analysis Set includes all enrolled subjects who have received any portion of at least 1 dose of study treatment (FPA144 + mFOLFOX6).

Phase 2

The Safety Analysis Set included all subjects in the ITT Analysis Set who have received any portion of at least 1 dose of study treatment (FPA144 + mFOLFOX6 or placebo + mFOLFOX6).

The Safety Analysis Set will be used in the summary for safety data as well as study treatment administration.

3.1.4 DLT-Evaluable Analysis Set

The DLT-Evaluable Analysis Set is defined for Phase 1 portion of the study. It includes all enrolled subjects who received at least 2 doses of FPA144 and mFOLFOX6 and completed Cycles 1 and 2 of treatment, or who experienced a DLT in Cycle 1 or Cycle 2.

3.1.5 PK-Evaluable Analysis Set

The PK-Full Analysis Set and the PK-Evaluable Analysis Set are defined for Phase 1 and Phase 2 portion separately. The PK-Full Analysis Set includes all enrolled subjects (for Phase 1) or all randomized subjects (for Phase 2) who received at least 1 dose of FPA144 and have at least 1 serum FPA144 concentration datapoint.

Pharmacokinetic-Evaluable Analysis Set includes all subjects in the PK Full Analysis Set who had sufficient PK data for the reliable calculation of at least one PK parameter.

The PK-Evaluable Analysis Set is the primary analysis set for all PK analyses.

3.1.6 ADA-Evaluable Analysis Set

The Anti-Drug Antibody (ADA)-Evaluable Analysis Set is defined for Phase 1 and Phase 2 portion separately. It includes all enrolled subjects (for Phase 1) or all randomized subjects (for Phase 2) who received at least 1 dose of FPA144 and have at least 1 ADA sample drawn at any timepoint with available ADA data.

The ADA-Evaluable Analysis Set is the primary analysis set for ADA analyses.

3.2 Subject Grouping

For analyses based on the ITT Analysis Set or Efficacy-Evaluable Analysis Set, subjects will be grouped according to the treatment to which they were randomized.

For analyses based on the Safety Analysis Set, DLT-Evaluable Analysis Set, PK-Evaluable Analysis Set, or ADA-Evaluable Analysis Set, subjects will be grouped according to the actual treatment received. In the Phase 2 portion, the actual treatment received will differ from the randomized treatment only when their actual treatment differs from randomized treatment for the entire treatment duration.

3.3 Stratification Factors

In the Phase 2 portion of the study, subjects will be randomized 1:1 to receive FPA144 + mFOLFOX6 or placebo + mFOLFOX6, stratified based on the following factors:

- Geographic region: Region 1 (including US, Europe and Australia) vs. Region 2 (China) vs. Region 3 (Rest of Asia including Japan, South Korea, Taiwan and Thailand) vs. Region 4 (Rest of World)
- Prior treatment status: *de novo* vs. adjuvant/neo-adjuvant
- Administration of a single dose of mFOLFOX6 prior to enrollment: Yes vs. No

If there are discrepancies in stratification factor values between the interactive voice or web response system (IXRS) and the clinical database, the values recorded in the clinical database will be used for analyses.

Given the small number of subjects in the adjuvant/neo-adjuvant stratums, the stratification factor of prior treatment status will not be considered for the stratified efficacy analysis in the final analysis.

Analyses for efficacy endpoints will be adjusted for the stratification factors. In the situation where there is insufficient information in a stratum (ie, if there are < 20 subjects or there are no informative events in a stratum), pooling of the stratum with the smallest adjacent stratum for stratified analyses will be considered; the smallest stratum is defined as the stratum having the fewest number of subjects or the fewest number of events in case the former is a tie and the adjacent stratum is defined as a stratum having 1 factor of the 2 at the same level and the other factor at an adjacent level.

3.4 Examination of Subject Subgroups

In the analysis of the primary and secondary efficacy endpoints, subgrouping of subjects based on randomization stratification factors will be explored for subgroup analyses. In addition, subgroups defined by presumed prognostic baseline characteristics may also be explored. The presumed prognostic baseline characteristics include but not limited to the following:

- Age (<65 years and ≥65 years)
- Sex (male and female)

- FGFR2b expression
  - Overexpression by IHC irrespective of ctDNA
  - Amplification by ctDNA irrespective of IHC
  - Both overexpressed by IHC and amplified by ctDNA
  - Tumor IHC staining score of 2+ or 3+ in greater than 10% of cells
  - Tumor IHC staining score of 2+ or 3+ in greater than 5% of cells

To graphically display treatment effect changes across subsets, forest plots of PFS and OS hazard ratios, and difference in ORR will be provided.

3.5 Multiple Comparisons

In the Phase 2 portion of the study, the primary endpoint, PFS, will be tested first at a one-sided level of 0.1. If the null hypothesis is rejected, then the secondary endpoints OS and ORR will be tested hierarchically at the same one-sided level of 0.1. OS will be tested first and if it is significant, the ORR will be tested. The family-wise Type I error rate of testing the primary and the secondary endpoints will be controlled by employing this gate-keeping testing procedure at one-sided level of 0.1.

3.6 Missing Data and Outliers

3.6.1 Missing Data

In general, missing data will not be imputed unless methods for handling missing data are specified.

The handling of missing or incomplete dates for disease diagnosis and prior anticancer therapy is described in Section 5.3; for prior and concomitant medications in described in Section 5.4; for the single dose of mFOLFOX6 allowed prior to enrollment is described in Section 5.5; for new anticancer therapy is described in Section 6.1.1, for death date is described in Section 6.3.1, for AE onset is described in Section 7.1.5.

3.6.2 Outliers

Outliers will be identified during the data management and data analysis process, but no sensitivity analyses will be conducted. All data will be included in the data analysis.

3.7 Data Handling Conventions and Transformations

In PK analysis, individual serum FPA144 concentrations, if deemed to be anomalous, may be excluded from the analysis at the discretion of the pharmacokineticist following a review of

available documentation (eg, bioanalytical report). Any such exclusion will be communicated and clearly listed in the study report along with a justification for the exclusion.

Individual serum concentrations will be excluded from descriptive statistics for serum concentration data, including mean concentration versus time plots, if the sample was planned to be collected predose but was actually collected postdose.

Entire serum concentration-time profiles for a patient may be excluded following review of available documentation (eg, bioanalytical report) and communication. Any such exclusion will be communicated and clearly listed in the study report along with justification for exclusion. Results of the analysis with and without the excluded profiles will be presented in the study report when it is necessary.

All serum concentrations reported as No Result (NR or Not Collected/Not Done, ND) values will be treated as missing and will appear in the data set as ".". For the purpose of calculating or plotting mean concentration-time data, or calculating PK parameters, concentration values determined to be below the limit of quantitation (BLQ) will be treated as zero if they occur prior to the first measurable concentration; all other BLQ values will be treated as missing and set to ".". Quantifiable concentrations after two consecutive BLQ values following the same dose will also be set to "." for the purposes of calculating PK parameters.

3.8 Analysis Visit Windows

3.8.1 Definition of Study Day

For subjects in the Phase 1 portion, study day will be calculated from the first dosing date of any portion of the study drug:

- Postdose Study Days = Assessment Date – First Dosing Date + 1
- Study Day prior to First Dose = Assessment Date – First Dosing Date

For subjects in the Phase 2 portion, study day will be calculated from the randomization date:

- Postdose Study Days = Assessment Date – Randomization Date + 1
- Study Day prior to Randomization = Assessment Date – Randomization Date

3.8.2 Analysis Visit Windows

No analysis visit window will be assigned in the analysis. No summary by visit is planned except for PK analysis. In the by-visit summary provided for PK data, nominal visit will be used.

In general, the baseline value will be the last nonmissing value on or prior to the first dosing date of study drug (for Phase 1 subjects) or randomization date (for Phase 2 subjects) unless specified differently.

For continuous measurements, if multiple measurements occur on the same day, the last nonmissing value will be considered as the baseline value. If these multiple measurements occur at the same time or the time is not available, the average of these measurements will be considered the baseline value. For categorical measurements, if multiple measurements occur on the same day, the last nonmissing value will be considered as the baseline value. If these multiple measurements occur at the same time or the time is not available, the value with the lowest severity will be considered the baseline value.

Values collected after the first dosing date of study drug (for Phase 1 subjects) or randomization date (for Phase 2 subjects) will be considered as postbaseline values. For PK data, the measurement collected at protocol specified nominal timepoint will be selected for analysis.

4 SUBJECT DISPOSITION

4.1 Subject Enrollment and Disposition

A summary of subject disposition will be provided by treatment group. Percentages will be based on the Safety Analysis Set for Phase 1 portion, and ITT Analysis Set for Phase 2 portion. The number of subjects in the following categories will be provided:

- Pre-screened (Phase 2 only)
- Signed the inform consent
- Randomized (Phase 2 only)
- Randomized but not treated (Phase 2 only)
- Received any study treatment
- Continuing study treatment
- Discontinued from study treatment with reasons for treatment discontinuation
- Continuing study
- Discontinued from study with reasons for study discontinuation

4.2 Extent of Exposure and Adherence

Descriptive statistics of extent of exposure will be presented by treatment group for each component of treatment (FPA144/placebo, Oxaliplatin, Leucovorin, 5FU) separately:

- Duration of exposure (weeks)
- Cumulative exposure by week
- Number of infusions
- Dose intensity (Phase 2 only)

Total duration of exposure to study drug (in weeks) will be defined as (last available dosing date – first dosing date + 14)/7, regardless of any temporary interruptions in study drug administration.

Dose intensity is defined as 100* (Total study drug administered in mg/ Total study drug expected to be administered in mg during exposure to study drug). Percentage of subjects in the intensity categories (<75% and ≥75%) will be provided.

The number and percentage of subjects who have dose reduction, dose delay or interruption, and infusion interruption will be summarized with reasons.

Summaries of exposure will be performed with the Safety Analysis Set. A by-subject listing of study drug administration will be provided.

5 DEMOGRAPHICS AND BASELINE CHARACTERISTICS

5.1 Demographics

Demographic data will be summarized using descriptive summary statistics for the Safety Analysis Set (Phase 1 portion) or the ITT Analysis Set (Phase 2 portion). The demographic characteristics include age, sex, race, ethnicity, body height (in cm), body weight (in kg) and body mass index (BMI; in kg/m^2).

A by-subject listing will be provided for demographic data.

5.2 Other Baseline Disease Characteristics

Baseline characteristics including baseline Eastern Cooperative Oncology Group Performance Status (ECOG PS), geographic region (US and EU vs China versus Rest of Asia [including Japan, South Korea, Taiwan and Thailand]), prior treatment status (de novo vs adjuvant/neo-adjuvant), and administration of a single dose of mFOLFOX6 prior to enrollment (yes vs no) will be summarized by treatment group for the Safety Analysis Set (Phase 1 portion) or the ITT Analysis Set (Phase 2 portion) as part of the disease-specific baseline characteristics.

5.3 Medical History

Medical history will be collected at screening for disease-specific and general conditions (ie, conditions not specific to the disease being studied).

A summary of disease-specific medical history will be provided for the Safety Analysis Set (Phase 1 portion) or the ITT Analysis Set (Phase 2 portion) as part of the disease-specific baseline characteristics. Time since initial diagnosis of cancer (months) and time since diagnosis of unresectable disease (months) will be calculated by (date of randomization – date of diagnosis) / 30.4375. They will be summarized using summary statistics for a continuous variable. Disease stage at diagnosis and at screening will be summarized using summary statistics for a categorical variable.

In deriving the time since diagnosis, all partial dates of diagnosis and last regimen will be identified, and the partial dates will be imputed as follows:

- If day and month are missing but year is available, then the imputed day and month will be 01 Jan.
- If day is missing but the month and year are available, then the imputed day will be the first day of the month.
- Partial date will not be imputed if the year is missing.

General medical history data will be coded according to Medical Dictionary for Regulatory

Activities (MedDRA) Version 20.1. It will be listed only. A by-subject listing will be provided for disease-specific medical history.

5.4 Prior and Concomitant Medications

Prior and concomitant medications will be coded using the World Health Organization Drug Dictionary (WHODD) and classified according to Anatomical Therapeutic Chemical classification (ATC) codes levels 2 (therapeutic sublevel) and 4 (chemical sublevel).

All medications with an end date prior to the first dose of any study drug will be considered as prior medication regardless of the stop date. If a partial start date is entered the medication will be considered prior unless the month and year (if day is missing) or year (if day and month are missing) of the start date are after the first dosing date. Medications with a completely missing start date will be considered as prior medication, unless otherwise specified

Concomitant medications are defined as medications taken while a subject took study drug. If a partial stop date is entered, any medication with the month and year (if day is missing) or year (if day and month are missing) prior to the date of first study drug administration will not be considered as concomitant medication. If a partial start date is entered, any medication with the month and year (if day is missing) or year (if day and month are missing) after the study drug stop date will not be considered as concomitant medication. Medications with completely missing start and stop dates will be considered as concomitant medication, unless otherwise specified.

All prior and concomitant medications (other than per-protocol study drugs) will be provided in a by-subject listing.

5.5 Prior Anticancer Therapy

Number of prior regimens, time since the completion of last regimen will be summarized by treatment group using descriptive statistics. The best response to the last regimen will be summarized using summary statistics for a categorical variable. The summaries will be based on the Safety Analysis Set (Phase 1 portion) or the ITT Analysis Set (Phase 2 portion) as part of the disease-specific baseline characteristics. A partial completion date will be imputed using the algorithm defined in Section 4.3. The prior anticancer therapy will be listed by subject.

For the single dose of mFOLFOX6 allowed prior to enrollment, a partial dose date will be imputed as follows:

- If the day is missing but the month and year are available, then impute the day as first day of the month, or the informed consent date for pre-screening if they have the same month and year, whichever is later.
- If the day and month are missing but year is available, then impute the day and the month as 01Jan, or the informed consent date for prescreening if they have the same year, whichever is later.
- Partial date will not be imputed if the year is missing.

6 EFFICACY ANALYSES

The efficacy analysis for Phase 1 and Phase 2 portions will be performed and reported separately. Efficacy summaries will be presented by treatment group based on the Safety Analysis Set (Phase 1 portion) or the ITT Analysis Set (Phase 2 portion).

6.1 Efficacy Endpoints in Phase 1

Best overall response (BOR) is reported directly by the investigators in the Phase 1 portion of the study. A summary of the number of subjects in response categories and the overall response rate, defined as the proportion of subjects who achieve BOR of either complete response (CR) or partial response (PR), will be provided. A by-subject listing of BOR will also be provided.

6.2 Primary Efficacy Endpoint in Phase 2

6.2.1 Definition of the Primary Efficacy Endpoint

The primary efficacy endpoint in the Phase 2 portion of the study is PFS, defined as time from randomization until the date of radiographic disease progression based on investigator assessment (using RECIST v1.1) or death from any cause, whichever comes first.

The PFS analysis will include only radiographic progression events as determined by the investigator’s assessment per RECIST v1.1 and deaths. A clinical deterioration determined by an investigator will not be considered as a progression event. Data will be censored on the date of last adequate tumor assessment for subjects:

- who do not have documented progression or die, or
- who start new anticancer therapy before documented progression or death without documented progression, or
- who have ≥2 consecutive missing tumor assessments before documented progression or death without documented progression

If a subject does not have a baseline tumor assessment, then PFS will be censored at the date of randomization, regardless of whether or not radiographic progression or death has been observed.

When the date of initiation of new anticancer therapy other than the study treatment is incomplete or missing, the following algorithm will be followed:

- If the day is missing but the month and year are available, then the imputed day will be the last day of the month.
- If day and month are missing but year is available, then the imputed day and month will be the last day of the month for the last adequate disease assessment if they have the

same year.

6.2.2 Statistical Hypothesis for the Primary Efficacy Endpoint

The primary efficacy hypothesis to be tested is that there is no difference in PFS between FPA144 + mFOLFOX6 and placebo + mFOLFOX6. Using $S_T(t)$ and $S_C(t)$ to denote the PFS distribution functions of FPA144 + mFOLFOX6 and placebo + mFOLFOX6, respectively, the statistical hypotheses to be tested in this study will be:

H_0: $S_T(t) = S_C(t)$

H_1: $S_T(t) > S_C(t)$ (FPA144 + mFOLFOX6 is superior to placebo + mFOLFOX6 in terms of PFS)

6.2.3 Analysis of the Primary Efficacy Endpoint

The primary analysis of PFS will be performed using the Kaplan-Meier method for the ITT Analysis Set. The PFS distribution of 2 treatment groups will be compared using the stratified log-rank test, stratified by the stratification factors at randomization. Medians, Q1, Q3, the proportion of subjects who progression-free at 6, 9, and 12 months from randomization will be provided along with corresponding 95% confidence intervals (CI). The unstratified log-rank test will also be performed. Kaplan-Meier curves will be provided by treatment group.

In addition, the HR between the 2 treatment groups and its 95% CI will be estimated using the Cox proportional hazards regression model with treatment group as the only main effect and stratified by the stratification factors at randomization.

A listing will be provided for the information of subject PFS, including randomization date, date of PFS event or censor date.

6.2.4 Sensitivity Analysis of the Primary Efficacy Endpoint

To assess the robustness of the primary PFS results, the following sensitivity analyses will be performed:

- PFS will be analyzed by considering initiation of new anticancer therapy as a PFS event. Furthermore, PFS will not be censored by having ≥2 consecutive missing tumor assessments before documented progression or death or initiation of new anticancer therapy.

- PFS will be analyzed by considering clinical progression as a PFS event. The date of documented radiographic progression, clinical progression, or death, whichever is earlier, will be considered as PFS event date.

- PFS will be analyzed based on the Efficacy-Evaluable Analysis Set with the same analysis methods specified for the primary analysis.

Other methods, e.g., restricted mean survival time, may be considered for PFS as exploratory analysis if the proportional hazard assumption is not hold.

6.2.5 Exploratory Analysis of the Primary Efficacy Endpoint

Exploratory analysis will be performed to investigate the potential prognostic factors influencing PFS using the Cox regression model. The variables with prognostic potential will be included in the model to identify plausible significant factors on PFS. The potential variables include but not limit to the following:

- Age (<60 years and ≥60 years)
- Sex (male and female)
- 3 stratification factors at randomization: geographic region, prior treatment status, and administration of a single dose of mFOLFOX6 prior to enrollment
- Extent of disease (locally advanced and metastatic)
- Primary tumor site (gastric adenocarcinoma and gastroesophageal junction adenocarcinoma)
- Measurability of disease (measurable disease and non-measurable disease at baseline)
- Number of lesions at baseline (1-4 lesions and >4 lesions)
- Visceral metastasis (yes and no)
- Prior gastrectomy (yes and no)
- H score of IHC test (as continuous variable)

Each candidate variable will be preliminarily evaluated in the Cox regression model with treatment. Only the variables significant at the 0.4 level will be considered to build the multivariate model. A stepwise selection process with significance level of 0.3 for entering variables will then be applied to those candidate variables to identify the final subset of relevant covariates in the Cox regression model with treatment. The hazard ratio of the variables in the final set will be provided. Same analysis may be done on geographic subgroups.

PFS will also be analyzed based on the subgroups defined in Section 3.4. Same analysis as specified for the primary analysis will be performed, except that only the unstratified log-rank test will be provided and the hazard ratio estimated by the Cox proportional hazard model will not be adjusted by randomization stratification factors.

6.3 Secondary Efficacy Endpoints in Phase 2

6.3.1 Definition of the Secondary efficacy Endpoints

The secondary efficacy endpoints of this study are OS and ORR.

OS is defined as time from randomization until death from any cause. Subjects who are lost to follow-up or do not have a date of death will be censored at the last date that they were known to be alive. Subjects with confirmed death or alive status after the data cutoff date will be censored at the data cutoff date.

Every attempt will be made to ensure that complete death dates are recorded. In those rare instances where complete death dates are not recorded, the following algorithm will be used:

- If day is missing but the month and year are available, then the imputed day will be the midpoint of the month or the last assessment date + 1, whichever is later.
- If day and month are missing but year is available, then the imputed day and month will be 01Jan or the last day of the latest month that the subject was known to be alive if they have the same year, whichever is later.

ORR is defined as the proportion of subjects who achieve BOR of either CR or PR based on investigator assessment tumor lesions per RECIST v1.1. The BOR is the best response documented from randomization until the end of study or data cut-off date, first disease progression, death, start of new anti-cancer therapy, or last documented assessment before ≥2 consecutive missing tumor assessments, whichever is earlier. Subjects, who do not have sufficient baseline or on-study tumor status information to be adequately assessed for response status (ie, those with BOR of not evaluable [NE]), received anticancer therapy other than the study treatment, or missing ≥2 consecutive missing tumor assessments prior to achieving CR or PR, will be considered as non-responders and will be included in the denominators in calculations of response rates.

To preserve the overall type I error rate across the primary and secondary endpoints of the study at a 1-sided significance level of 0.1, the primary efficacy hypothesis must be rejected at the 1-sided 0.1 significance level before the efficacy hypotheses for the secondary efficacy endpoints can be tested. The 2 secondary endpoints will be tested sequentially at the 1-sided 0.1 significance level in the order of OS then ORR. If a null hypothesis is not rejected, formal sequential testing will be stopped and only the nominal significance level will be presented for the remaining endpoints.

6.3.2 Analysis of the Secondary Efficacy Endpoints

<u>**OS**</u>

The primary analysis of OS will be performed using the Kaplan-Meier method for the ITT

Analysis Set. The OS distribution of 2 treatment groups will be compared using the stratified log-rank test, stratified by the stratification factors at randomization. Medians, Q1, Q3, the proportion of subjects who are alive at 6, 12, and 15 months from randomization will be provided along with corresponding 95% CIs. The unstratified log-rank test will also be performed. Kaplan-Meier curves will be provided by treatment group.

In addition, the HR between the 2 treatment groups and its 95% CI will be estimated using the Cox proportional hazards regression model with treatment group as the only main effect and stratified by the stratification factors at randomization.

A listing will be provided for the information of subject OS, including randomization date, date of OS event or censor date.

To assess the robustness of the primary OS results, the analysis will be performed based on the Efficacy-Evaluable Analysis Set with the same analysis methods specified for the primary analysis as the sensitivity analysis.

Exploratory analysis for OS will be performed with the same methods for PFS as descried in Section 6.2.5. Multivariate model with potential prognostic factors influencing OS will be built using Cox regression model. Subgroup analysis will also be performed.

ORR

The analysis of ORR will be performed based on the ITT Analysis Set. ORR with corresponding 2-sided 95% CI based on Clopper-Pearson method along with each category of BOR will be summarized by treatment group. Patients who don't have any postbaseline adequate tumor assessments will be counted as non-responders. A conventional 2-sided 95% CI of the difference in ORR of the two treatment groups and the corresponding p-value will be calculated based on stratum-adjusted Cochran-Mantel-Haenszel (CMH) proportions (Koch, Carr, Amara, Stokes, & Uryniak, 1989):

$$\hat{p}_t - \hat{p}_c \pm Z_{1-\alpha/2} \cdot SE(\hat{p}_t - \hat{p}_c),$$

where

- p_t and p_c denote the response rate in FPA144 + mFOLFOX6 and placebo + mFOLFOX6 treatment group, respectively.
- $\hat{p}_t - \hat{p}_c = \frac{\sum W_h d_h}{\sum W_h}$, where $d_h = \hat{p}_{th} - \hat{p}_{ch}$ is the stratum-adjusted CMH proportion difference in stratum h ($h = 1, 2, \dots, K$).
- $W_h = \frac{n_{th} n_{ch}}{n_{th} + n_{ch}}$, is the weight based on the harmonic mean of sample size per treatment group for each stratum where n_{th} and n_{ch} are the sample sizes of each treatment group in stratum h.

- $SE(\hat{p}_t - \hat{p}_c) = \frac{\sqrt{\sum W_h^2 \left(\frac{\hat{p}^*_{th}(1-\hat{p}^*_{th})}{n_{th}-1} + \frac{\hat{p}^*_{ch}(1-\hat{p}^*_{ch})}{n_{ch}-1}\right)}}{\sum W_h}$, where $\hat{p}^*_{th} = \frac{m_{th}+0.5}{n_{th}+1}$, $\hat{p}^*_{ch} = \frac{m_{ch}+0.5}{n_{ch}+1}$, m_{th} and m_{ch} are the number of responders in stratum h of each treatment group.
- $Z_{(1-\alpha/2)}$ is the 100(1-α/2)th percentile of normal distribution, where $\alpha = 0.05$.

To assess the robustness of the primary ORR results, the analysis will be performed based on the Efficacy-Evaluable Analysis Set with the same analysis methods specified for the primary analysis as the sensitivity analysis.

Exploratory analysis will be performed to investigate the potential prognostic factors influencing ORR using the logistic regression model. The potential variables include but not limit to the ones that have been identified in Section 6.2.5.

Each candidate variable will be preliminarily evaluated in the logistic regression model with treatment. Only the variables significant at the 0.4 level will be considered to build the multivariate model. A stepwise selection process with significance level of 0.3 for entering variables will be applied to those candidate variables to identify the final subset of relevant covariates in the logistic regression model with treatment. The odds ratio of the variables in the final set will be provided.

ORR will also be analyzed based on the subgroups defined in Section 3.4. Same analysis as specified for the primary analysis will be performed, except for p-value of the difference in ORR will not be provided and the 95% CI of the difference in ORR will be calculated without adjusting by the randomization stratification factors.

6.4 Exploratory Efficacy Endpoints in Phase 2

6.4.1 Duration of Response

Duration of response is defined as the time from the first documented CR or PR to the earlier of the first documented PD or death from any cause. DOR will be evaluated using the investigator assessments based on subset of subjects in ITT Analysis Set who achieve a response. DOR will be summarized using Kaplan-Meier methods (median, Q1, Q3, and corresponding 95% CI).

In the analysis of DOR, data will be censored on the date of the last tumor assessment for subjects

- who do not have documented progression or die, or
- who start new anticancer therapy before documented progression or death without documented progression, or
- who have ≥2 consecutive missing tumor assessments before documented progression or

death without documented progression

6.4.2 Time to Response

Time to response (TTR) is defined as the interval from randomization to the first documented CR or PR. TTR will be evaluated in the subset of subjects in ITT Analysis Set who achieve a response. Descriptive statistics of TTR will be provided.

6.4.3 Change in Tumor Size

Tumor size based on the sum of the diameters of target lesions is collected in the eCRF through an MRI/CT scan at each tumor assessment visit. Change from baseline and the percent change from baseline will be determined for each post baseline assessment. The best change from baseline and the best percent change from baseline are defined as change or the percentage change from baseline to postbaseline minimum in tumor size while the assessment was taken prior to the time of initiation of anticancer treatment other than the study treatment or have ≥2 consecutive missing tumor assessments before documented progression or death without documented progression.

Change in tumor size will be analyzed for subjects in the ITT Analysis Set who have assessments at baseline and at least 1 post-baseline time point. Baseline, postbaseline maximum, postbaseline minimum, and their percent (%) change from baseline will be calculated and summarized. Waterfall plot will be provided for the best percent (%) change from baseline. A listing of target lesion will be provided.

6.4.4 Patient-Reported Outcome Assessments

Patient-Reported Outcome includes EORTC QLQ-C30 and EQ-5D-5L. The EORTC QLQ-C30 is a self-reported cancer health-related questionnaire. It includes five function domains (physical, role, emotional, cognitive, social), eight symptoms (fatigue, pain, nausea/vomiting, constipation, diarrhea, insomnia, dyspnea, and appetite loss), as well as global health/quality-of-life and financial difficulties. The EQ-5D-5L is a self-report questionnaire used to assess a subject’s general health quality of life consisting of five dimensions (mobility, self-care, usual activities, pain or discomfort, and anxiety or depression) as well as a VAS for overall health.

Data from EORTC QLQ-C30 and EQ-5D-5L will be scored, processed, and standardized according to their user manuals. Data will be analyzed using appropriate methods specified in the user manuals to account for incomplete questionnaires. The maximum improvement postbaseline and change from baseline in subscales and global scores of EORTC QLQ-C30 and EQ-5D VAS, and the minimum and maximum postbaseline in dimensions of EQ-5D-5L will be summarized descriptively by treatment groups for subjects in the ITT Analysis Set. By subject listings will be provided for EORTC QLQ-C30 and EQ-5D-5L.

6.4.5 ECOG PS

The ECOG PS score has a range from 0 (Fully active; able to carry on all pre-disease performance without restriction) to 5 (Dead). The best postbaseline performance status will be the lowest score and the worst postbaseline performance status will be the highest score after randomization. A listing of ECOG PS will be provided.

6.5 Changes from Protocol-Specified Efficacy Analysis

Analysis and summary are provided for time to response and change in tumor size.

7 SAFETY ANALYSES

Unless otherwise specified, all analyses will be performed using the Safety Analysis Set. The safety analysis for Phase 1 and Phase 2 portions will be performed and reported separately.

No formal comparisons of safety endpoints are planned.

7.1 Adverse Events and Deaths

7.1.1 Adverse Event Dictionary

All AEs will be coded to system organ class (SOC) and preferred term (PT) using MedDRA Version 20.1.

7.1.2 Adverse Event Severity

Adverse events are graded for severity by the investigators using the National Cancer Institute Common Toxicity Criteria for Adverse Events (NCI-CTCAE) Version 5. The severity grade of events for which the investigator did not record severity will be categorized as “missing” for tabular summaries and data listings.

7.1.3 Relationship of Adverse Event to Study Drug

A treatment related AE is an AE noted as related to FPA144/placebo, Oxaliplatin, Leucovorin, or 5FU by the investigator. Events for which the investigator did not record relationship to study drug will be considered related to study drug for summary purposes. However, by-subject data listings will show the relationship as missing.

7.1.4 Serious Adverse Events

Serious adverse events (SAEs) will be identified and captured as SAEs if the AEs met the definitions of SAEs that were specified in the study protocol.

7.1.5 Treatment-Emergent Adverse Events

A treatment-emergent AE (TEAE) is defined as an AE that was not present prior to the start date of study drug or was worsened during treatment and 28 days after permanent discontinuation of study drug. An AE that was present at treatment initiation but resolved and then reappeared and the event severity increase while the subject was on treatment is also a TEAE.

If the onset date of the AE is incomplete and the AE stop date is not prior to the first dosing date of study drug, then the month and year (or year alone if month is not recorded) of onset determine whether an AE is treatment emergent. The event is considered treatment emergent if both of the following 2 criteria are met:

- The AE onset is the same as or after the month and year (or year) of the first dosing date

of study drug, and

- The AE onset date is the same as or before the month and year (or year) of the date corresponding to 28 days after the date of the last dose of study drug

An AE with completely missing onset and stop dates, or with the onset date missing and a stop date later than the first dosing date of study drug, will be considered treatment emergent. In addition, an AE with the onset date missing and incomplete stop date with the same or later month and year (or year alone if month is not recorded) as the first dosing date of study drug will be considered treatment emergent.

7.1.6 Summary of Adverse Events and Deaths

The number and percentage of subjects who experienced at least 1 TEAE will be provided and summarized by SOC, PT, and treatment group. For other AEs described below, summaries will be provided by SOC, PT, maximum severity, and treatment group

- TEAE (both Phase 1 and Phase 2)
- TE SAE (both Phase 1 and Phase 2)
- Summary of TEAE of Grade 3-5 (both Phase 1 and Phase 2)
- TEAE related to FPA144/Placebo
- TEAE related to mFOLFOX6
- TE SAE related to FPA144/Placebo
- TE SAE related to mFOLFOX6
- TEAE leading to FPA144/Placebo treatment discontinuation
- TEAE leading to mFOLFOX6 treatment discontinuation
- TEAE leading to death (both Phase 1 and Phase 2)
- TEAE leading to dose reduction in FPA144/Placebo
- TEAE leading to dose reduction in mFOLFOX6
- TEAE leading to dose delayed in FPA144/Placebo
- TEAE leading to dose delayed in mFOLFOX6
- TEAE leading to infusion interruption in FPA144/placebo

- TEAE leading to infusion interruption in mFOLFOX6
- TEAE of dose limiting toxicity (Phase 1 only)

These summaries will be provided for the Phase 2 portion if not otherwise specified.

A brief, high-level summary of AEs described above will be provided for Phase 1 and Phase 2 portions by treatment group and by the number and percentage of subjects who experienced the above AEs.

Multiple events will be counted only once per subject in each summary. AEs will be summarized in alphabetic order of SOC and then by PT in descending order of total frequency within each SOC. For summaries by severity, the most severe severity will be used for those AEs that occurred more than once in a subject.

In addition to the above summary tables, TEAEs, TEAEs of Grade 3 or higher, TE treatment-related AEs, and TE SAEs will be summarized by PT only in descending order of total frequency.

All AE and recorded deaths for the safety population will be listed.

7.1.7 Adverse Events of Special Interest

An AE of special interest (AESI) (serious or non-serious) is an event of medical concern considered potentially associated to the investigational product or disease under study, for which ongoing monitoring and rapid communication by the investigator to the Sponsor is necessary. The following events are considered events of special interest in this study:

- Ocular events associated with symptomatic corneal involvement and symptomatic and asymptomatic retinal involvement: these events are defined as those AEs in the Standardised MedDRA Queries (SMQs) (Broad) of Corneal Disorders and Retina Disorders
- Events of hypersensitivity: these events are defined as those AE in the SMQ (Broad) of Hypersensitivity

Treatment-emergent AESIs of ocular events are defined as those AEs present not prior to the start date of study drug or was worsened during treatment and 100 days after permanent discontinuation of study drug, as collected from CRF. The treatment-emergent definition for general AEs is still applied for AESIs of hypersensitivity events.

Treatment-emergent AESIs will be summarized by PT and maximum CTCAE Grade for the Phase 2 portion.

In addition, time to first onset of ocular events of any grade, Grade 2 and above, and Grade 3 and

above will be summarized, respectively. Kaplan Meier estimates of the median, Q1, Q3, and the number of subjects with event and censored subjects will be provided. Time to first onset of ocular events is defined as the time from start of study treatment to the date of first incident of ocular events. In the absence of an ocular event, the censoring date will be the earliest from the following dates:

- Last dose date of FPA144/placebo + 100 days
- Death date
- Analysis cut-off date

In case when the AE onset date is incomplete and needs to be imputed, the following algorithm will be followed:

- If the day is missing but the month and year are available, then the imputed day will be the first day of the month or the first dosing date if they have the same month and year, whichever is later.
- If the day and month are missing but year is available, then the imputed day and month will be 01Jan or the first dosing date if they have the same year, whichever is later.

By subject listings of AESI will be provided.

7.1.8 Dose-Limiting Toxicity

The summary and listing of DLTs will be performed on the DLT-Evaluable Analysis Set for the Phase 1 portion. A summary of DLT will be provided by SOC, PT, and severity. All DLTs will be listed.

7.2 Clinical Laboratory Evaluations

Summaries of laboratory data will be provided in the Safety Analysis Set and will include data collected up to the last dose of study drug plus 28 days for subjects who have discontinued study drug, or all available data at the time of the final analysis data-cut for subjects who are ongoing at the time of the final analysis.

Treatment-emergent laboratory abnormalities are defined as values that increase at least 1 toxicity grade from baseline at any postbaseline time point. If the relevant baseline laboratory value is missing, any abnormality of at least Grade 1 observed within the time frame specified above will be considered treatment emergent.

The following summaries will be provided by lab test and treatment group. Subjects will be categorized according to the most severe postbaseline abnormality grade for a given laboratory test:

- Laboratory abnormalities (both Phase 1 and Phase 2)
- Grade 3 or 4 laboratory abnormalities (Phase 2 only)

Shift tables displaying subject counts and percentages classified by baseline grade and maximum grade on treatment will be provided for chemistry, hematology, and coagulation tests with CTCAE v5 grading for Phase 2 portion of the study.

By-subject listings of laboratory test results collected throughout the study will be provided for chemistry, hematology, and coagulation tests for both Phase 1 and Phase 2 portions of the study.

7.3 Body Weight and Vital Signs

Descriptive statistics will be provided for both Phase 1 and Phase 2 portions of the study by treatment group for body weight and vital signs as follows:

- Baseline
- Postbaseline maximum
- Postbaseline minimum
- Change and percentage change from baseline to postbaseline maximum
- Change and percentage change from baseline to postbaseline minimum

A baseline value is defined as the last available value collected on or prior to the first dose of study drug.

A by-subject listing of body weight and vital signs will be provided by subject ID and time point in chronological order.

7.4 Electrocardiograms

Subjects with abnormal ECG findings will be listed for both Phase 1 and Phase 2 portions of the study.

7.5 Other Safety Measures

Ocular examinations including fluorescein staining score, ocular symptoms, fundoscopy, ocular coherence tomography, visual acuity, intraocular pressure, slit lamp biomicroscopy, and confrontation visual field exams were performed at baseline and postbaseline in the study. The number of subjects shifted from Not Clinically Significant at baseline to Clinically Significant at any postbaseline assessment will be summarized by examination and treatment group. The summary will be provided for the Phase 2 portion of the study.

In addition, shift in fluorescein staining score from baseline to most extreme postbaseline grade will be presented for the Phase 2 portion of the study.

A by-subject listing of the ocular examinations will be provided for both Phase 1 and Phase 2 portions of the study.

By-subject listings for pregnancy report and substance use of tobacco and alcohol will be provided for both Phase 1 and Phase 2 portions of the study.

8 PHARMACOKINETIC AND IMMUNOGENICITY ANALYSES

8.1 Bioanalytical Methods

The PK and ADA samples will be analyzed by validated ligand binding assays. The bioanalysis will be performed at ICON Bioanalytical Laboratories and will be conducted in a manner consistent with the FDA and Organization for Economic Cooperation and Development (OECD) Good Laboratory Practices (GLP) principles as they apply to bioanalytical chemistry.

8.2 PK Analysis

8.2.1 PK Sample Collection

In the Phase 1 portion of the study, blood samples to determine serum FPA144 concentration will be acquired from each subject on C1D1 (predose, 15 minutes, 4 hours, 48 hours, and 168 hours postdose), C1D8 (Cohort 2 subjects only, predose, 15 minutes, and 4 hours postdose), C2D1 (predose, 15 minutes, and 48 hours postdose), C3D1 (predose and 15 minutes postdose), C4D1 (same as C3D1), C5D1 (same as C3D1), C7D1 (same as C3D1), C9D1 (same as C3D1), C10D1(same as C2D1) C11D1 (same as C3D1), every 8 Cycles starting from Cycle 15 (same as C3D1), and EOT.

In the Phase 2 portion of the study, blood samples to determine serum FPA144 concentration will be acquired from each subject on C1D1 (predose and 15 minutes postdose), C3D1 (predose and 15 minutes postdose), C5D1 (predose), C9D1 (predose), C17D1 (predose), and EOT.

8.2.2 Estimation of Pharmacokinetic Parameters

FPA144 pharmacokinetic parameters will be derived from the serum concentration versus time profiles for C1D1 from Phase 1. Individual PK parameter values will be derived using a non-compartmental analysis (NCA) method for IV Infusion in Phoenix WinNonlin (Plasma 200 – 202). Actual elapsed sampling times relative to start of FPA144 infusion will be used for all parameter estimations. Individual C_{max} and C_{trough} as well as their accumulation ratios (RA1 and RA2, respectively) for Phase 1 and Phase 2 will be reported for the cycles with data. The PK parameters are defined below:

Variable	Units	Definition
AUC_{inf}	d*µg/mL (d=day)	Area under the observed concentration-time curve from the time of dose administration extrapolated to infinity post first dose, based on the last observed quantifiable concentration: $AUC_{inf} = AUC_{last} + C_{last}/\lambda_z$ where Clast is the last observed quantifiable serum concentration.
AUC_{inf}/Dose	[d*µg/mL]/[mg/kg]	AUC_{inf} normalized by dose administered.
AUC_{0-7}	d*µg/mL	Area under the observed concentration-time curve from the time of dosing to Day 7 (0-168h) post first

		dose calculated by log-linear trapezoidal approximation.
DN-AUC_{0-7}	[d*µg/mL]/[mg/kg]	AUC_{0-7} normalized by dose administered.
AUC_{0-14}	d*µg/mL	Area under the observed concentration-time curve from the time of dosing to Day 14 (0-336h) calculated by log-linear trapezoidal approximation.
DN-AUC_{0-14}	[d*µg/mL]/(mg/kg]	AUC_{0-14} normalized by dose administered.
Clast	µg/mL	Last observed temporal quantifiable serum concentration for both Cohorts of Phase 1 post first dose (Day 14 for Cohort 2 of Phase 1 post second dose).
CL	mL/d/kg	Total body clearance calculated post first dose. CL = Dose/AUC_{inf}.
C_{max}	µg/mL	Maximum observed concentration determined from the observed concentration values post first dose.
C_{max} /Dose	[µg/mL]/[mg/kg]	C_{max} normalized by dose administered.
$C_{max\ n}$ (dose 2 to last dose)	µg/mL	Observed concentration associated with the sample at the end of the infusion for each dose excluding the one on Study Day 8 for Cohort 2 of Phase 1. n = the dose number.
$C_{max\ n}$/Dose	[µg/mL]/[mg/kg]	$C_{max\ n}$ normalized by dose administered. n = the dose number.
$C_{trough\ n}$ (dose 1 to last dose)	µg/mL	Concentration associated with the sample at the end of each dose interval excluding the one on Study Day 8 for Cohort 2 of Phase 1. n = the dose number.
$C_{trough\ n}$/Dose (dose 1 to last dose)	[µg/mL]/[mg/kg]	$C_{trough\ n}$ normalized by dose administered. n = the dose number.
C_{term}	µg/mL	Observed concentration associated with the sample obtained at the end of the study.
λ_z	d_{-1}	Terminal phase rate constant of concentration-time profile.
λ_z lower	d	Time associated with the last observed concentration used to calculate λ_z.
λ_z upper	d	Time associated with the first observed concentration used to calculate λ_z.
MRT	d	Mean residence time. MRT = (AUMC/AUC_{inf}) – T/2 where AUMC=Area Under Moments Curve and T= infusion duration.
# Points Lambdaz		Number of points used in computing λ_z.
RA1		Accumulation ratio based on measurement at the end of infusion of $C_{max\ 1}$ compared to $C_{max\ n}$ calculated as $C_{max\ n}/C_{max\ 1}$.
RA2		Accumulation ratio based on measurement of $C_{trough\ 1}$ compared to $C_{trough\ n}$ calculated as $C_{trough\ n}/C_{trough\ 1}$.
Adjusted r2		Goodness of fit statistic for calculating λ_z.

$t_{1/2}$	d	Terminal half-life calculated post first dose. $t_{1/2}$= ln(2)/λ_z.
t_{last}	d	Time of the last quantifiable concentration for both Cohorts of Phase 1 post first dose (for Cohort 2 of Phase 1 post second dose).
t_{max}	d	Time to reach observed concentration corresponding to T_{last} for both Cohorts of Phase 1 post first dose (for Cohort 2 of Phase 1 post second dose).
V_{ss}	mL/kg	Steady-state distribution volume projected post first dose. V_{ss}= CL*MRT.
V_z	mL/kg	Volume of distribution based on the terminal phase post first dose. V_z = Dose/(AUC_{inf}*λ_z)

In Phase 1, blood samples to determine serum FPA144 concentration before the second dose of 7.5 mg/kg on C1D8 were collected twice at the same time for some patients in Cohort 2 of Phase 1. One of the samples was named as Cycle 1 Day 8 pre-dose. Another one was named as unscheduled 1008.00002. Both samples were included in individual serum FPA144 concentration listing, but only the samples named as Cycle 1 Day 8 pre-dose were used to estimate any PK parameters as it is the protocol specified nominal timepoint.

Only data points that described the terminal elimination log-linear decline of the serum FPA144 concentrations are to be used in the regression equation for calculation of terminal elimination phase rate constant; C_{max} and any data point(s) in the distribution phase are not to be included in the calculation. A minimum of 3 points are to be used for determination of the terminal elimination phase rate constant. A general rule of adjusted $r^2 > 0.80$ will be considered as acceptable for calculation of the terminal elimination phase rate constant. If adjusted r^2 falls below 0.80, then the terminal elimination phase rate constant will be reported as not determined (ND) and that subject's AUC_{inf} and CL will be listed but excluded from descriptive summaries and statistical analysis. If the extrapolated AUC_{inf} is more than 20%, then both AUC_{inf} and CL will be listed but excluded from descriptive summaries and statistical analysis.

8.2.3 Assessment of Dose-Proportionality for Phase 1

Dose-proportionality will be assessed for C_{max}, AUC_{0-7}, and C_{trough} of C1D1 from Phase 1 at 6 mg/kg and 15 mg/kg using either a power model or a direct comparison of dose-normalized values. In power model, log(parameter) = a + b * log(dose), where a is the intercept, b is the slope and dose is actual dose in mg. Each log-transformed PK parameter will be fit with a power model with a fixed effect term for log-transformed dose. For each PK parameter, the slope and associated 90% CI will be presented. A minimum of 3 values per dose cohort must be available for a given parameter to estimate dose-proportionality with the power model. Dose-proportionality will be inferred if the 90% CI for the estimate of the slope contains 1.

8.2.4 Assessment of Steady-State Attainment for both Phase 1 and Phase 2

To assess steady-state attainment, graphical comparison will be performed using C_{max} and C_{trough}

of FPA144 for each Q2W cycle.

8.2.5 Assessment of Immunogenicity Impact on Serum FPA144 Concentration and Pharmacokinetic Parameters

No FPA144 treatment induced ADA positive patients were identified in Phase 1. The impact of ADA on FPA144 exposure from Phase 2 will be assessed if there are any confirmed treatment induced ADA positive patient using descriptive statistics to compare C_{max} and C_{trough} values between ADA positive and ADA negative patients at each dose level by cycle and by dose.

8.2.6 Summary of Serum Concentration data and Pharmacokinetic Parameters

Pharmacokinetic parameters will be generated for all subjects in the PK-Evaluable Analysis Set. Individual subject concentration data and individual subject PK parameters for FPA144 will be listed and summarized using descriptive statistics by Cohort and by Phase. Summary statistics (n, mean, StD, geometric mean, coefficient of variation of geometric mean [GeoCV], median, min, max, 95% CI) will be presented for both individual subject concentration data by time point and individual subject PK parameters by Cohort and by Phase. Non-continuous variables such as t_{max} will be summarized using n, median, minimum, and maximum. For summary statistics, BLQ values will be treated as specified in Section 3.6.1. Subjects in Phase 1 and Phase 2 portion of the study will be analyzed and summarized separately.

The following tables will be provided:

- Summary of FPA144 serum concentration (Phase 1 and Phase 2)
- Summary of FPA144 serum concentration with response and ADA status (Phase 2)
- Summary of pharmacokinetic parameters (Phase 1 and Phase 2)
- Summary of pharmacokinetic parameters with response and ADA status (Phase 2)

The following figures will be provided:

- Group mean (+/-StD) FPA144 serum concentration vs time profiles post first and second doses (Phase 1)
- Summary plot of C_{max} and C_{trough} versus time (Phase 1 and Phase 2)
- Individual scatter plot of FPA144 C_{max} and C_{trough} versus time (Phase 1 and Phase 2)
- Boxplot of FPA144 C_{max} and C_{trough} versus time with total patients, and with response and ADA status (Phase 2)

The following listings will be provided:

- Individual serum FPA144 concentration (Phase 1 and Phase 2)
- Individual Pharmacokinetic parameters (Phase 1)
- Individual Pharmacokinetic parameters with response and ADA status (Phase 2)

8.2.7 Quality Control Methods for PK data Analysis

The PK analysis will be subject to Quality Control (QC) review and reviewed by an independent pharmacokinetist at ICON.

8.3 Immunogenicity Analysis

8.3.1 Immunogenicity Samples Collection

In the phase 1 portion of the study, blood samples were collected before the infusion on Cycles 1, 2, 3, 7 and 10, and EOT to measure ADA for FPA144. In the phase 2 portion of the study, blood samples were collected before the infusion on Cycles 1, 2, 3, 5, 9, and 17, and EOT to measure ADA for FPA144.

8.3.2 Summary of Immunogenicity Results

The number (%) of subjects with the following anti-drug responses will be reported for immunogenicity subjects. The on-treatment period starts at first dose and beyond.

- Baseline FPA144 ADA-positive
- Baseline FPA144 ADA-negative
- FPA144 ADA-positive
- FPA144 ADA-negative

Postbaseline treatment induced ADA positive is derived as subjects with

- ADA negative at baseline and ADA positive at any postbaseline timepoint, or
- ADA positive at baseline and ADA positive with titer of at least 4-fold of the baseline titer at one or more postbaseline timepoint

The following table will be provided:

- Summary Statistics of Immunogenicity (Phase 2)

The following listings will be provided:

- Individual Immunogenicity Data (Phase 1 and Phase 2)

9 REFERENCES

Koch, G., Carr, G., Amara, I., Stokes, M., & Uryniak, T. (1989). *Categorical Data Analysis.* Chapter 13 in Berry, D.A. (ed.). Statistical Methodology in the Pharmaceutical Sciences. New York: Karcel Dekker, Inc.

10 SOFTWARE

SAS® Software Version 9.4. SAS Institute Inc., Cary, NC, USA

STATISTICAL ANALYSIS PLAN

**Study Title:**	FIGHT: A Phase 2 Randomized, Double-Blind, Controlled Study Evaluating FPA144 and Modified FOLFOX6 in Patients with Previously Untreated Advanced Gastric and Gastroesophageal Cancer: Phase 2 Preceded by Dose-Finding in Phase 1
**Name of Test Drug:**	FPA144
**Protocol Number:**	FPA144-004
**Protocol Version (Date):**	Amendment 4 (10 March 2021)
**Analysis Type:**	Follow-up Analysis
**Analysis Plan Version:**	1.0
**Analysis Plan Date:**	15 March 2021
**Analysis Plan Author:**	[redacted]

Approved by:

FivePrime

Lead Biostatistician (Print Name)	Signature	Date
Head of Biometrics (Print Name)	Signature	Date
Clinical Team Lead (Print Name)	Signature	Date
Head of Clinical Development (Print Name)	Signature	Date

TABLE OF CONTENTS

1 Background and Rationale 6
2 General Considerations 7
2.1 Analysis Sets 7
2.2 Subject Grouping 7
2.3 Stratification Factors 7
2.4 Examination of Subject Subgroups 7
2.5 Multiple Comparisons 7
2.6 Missing Data and Outliers 8
2.7 Analysis Visit Windows 8
3 Subject Disposition 9
3.1 Subject Enrollment and Disposition 9
3.2 Extent of Exposure and Adherence 9
4 Demographics and Baseline Characteristics 11
4.1 Demographics 11
4.2 Other Baseline Disease Characteristics 11
4.3 Medical History 11
4.4 Prior and Concomitant Medications 11
4.5 Prior Anticancer Therapy 11
5 Efficacy Analyses 12
5.1 Efficacy Endpoints 12
5.2 Analysis of Efficacy Endpoint 12
5.3 Other Efficacy Measures 12
6 Safety analyses 13
6.1 Adverse Events and Deaths 13
6.1.1 Adverse Event Dictionary 13
6.1.2 Adverse Event Severity 13
6.1.3 Relationship of Adverse Event to Study Drug 13
6.1.4 Serious Adverse Events 13
6.1.5 Treatment-Emergent Adverse Events 13
6.1.6 Summary of Adverse Events and Deaths 13
6.1.7 Adverse Events of Special Interest 14
6.2 Clinical Laboratory Evaluations 15
6.3 Body Weight and Vital Signs 16

6.4 Electrocardiograms 16
6.5 Other Safety Measures 16
7 References 18
8 software 19

LIST OF Abbreviations

ADA	anti-drug antibody
AE	adverse event
AESI	adverse event of special interest
ATC	Anatomical Therapeutic Chemical classification
AUC	area under the observed concentration-time curve
BMI	body mass index
BOR	best overall response
C_{max}	maximum observed concentration determined from the observed concentration values post first dose
C_{trough}	minimum observed concentration determined from the observed concentration values
CI	confidence interval
CL	total body clearance
CMH method	Cochran-Mantel-Haenszel method
CR	complete response
CSR	Clinical Study Report
CVT	computed tomography
DLT	dose-limited toxicity
DOR	duration of response
ECG	electrocardiogram
ECOG PS	Eastern Cooperative Oncology Group Performance Status
EORTC QLQ-C30	European Organization for Research and Treatment of Cancer Quality of Life Questionnaire Version 3.0
EOT	end of treatment
EQ-5D-5L	EuroQOL-5D-5L
GC	gastroesophageal cancer
GI	gastrointestinal tumors
GLP	Good Laboratory Practices
HR	hazard ratio
ITT	intent-to-treat
IXRS	interactive voice or web response system
LTFU	long-term follow-up
MedDRA	Medical Dictionary for Regulatory Activities
NCA	non-compartmental analysis method

NCI-CTCAE	National Cancer Institute Common Toxicity Criteria for Adverse Events
NE	not evaluable
OECD	Organization for Economic Cooperation and Development
ORR	objective response rate
OS	overall survival
PFS	progression-free survival
PK	pharmacokinetic
PR	partial response
PROs	patient-reported outcomes
PT	preferred term
Q1, Q3	first quantile, third quantile
Q2W	every 2 weeks
QOL	quality of life
RECIST v1.1	New Response Evaluation Criteria in Solid Tumors (version 1.1)
SAE	serious adverse event
SAP	Statistical Analysis Plan
SE	standard error
SMQ	standardised MedDRA queries
SOC	system organ class
StD	standard deviation
$t_{1/2}$	terminal half-life
TEAE	treatment-emergent adverse event
TFLs	Tables, Figures, and Listings
TTR	time to response
WHODD	World Health Organization Drug Dictionary

1 BACKGROUND AND RATIONALE

This statistical analysis plan (SAP) describes the statistical analysis methods and data presentations to be used in tables, figures and listings (TFLs) of the follow-up analysis in the clinical study report (CSR) for study FPA144-004. This SAP is based on the study protocol Amendment 4 dated 10 March 2021. The SAP will be finalized prior to data finalization for the follow-up analysis.

Study design and sample size remained unchanged (referred to the SAP for final analysis for detailed information). This follow-up analysis will be conducted with the Phase 2 portion of the study. It is considered as ad-hoc analysis, which will not be included in the type 1 error control as for the final analysis. The main objective of this follow-up analysis is to update the survival and safety information as supplement to the final analysis results.

The data up to 28 February 2021 will be cut and included in the analysis. Death and long-term follow-up data occur between the data cutoff date and data finalization date will be included only for overall survival derivation.

2 GENERAL CONSIDERATIONS

All statistical tabulations and analyses will be done using SAS®, Version 9.3 or higher.

Unless otherwise noted, continuous variables will be summarized using the number of subjects (n), mean, standard error (SE) or standard deviation (StD), median, minimum, and maximum; categorical variables will be summarized using the number and percentage of subjects in each category.

By-subject listings will be presented for all subjects in the Intent-to-Treat (ITT) Analysis Set (for Phase 2) and sorted by subject ID number, visit date, and time (if applicable). Data collected on log forms, such as AEs, will be presented in chronological order within the subject. The treatment group to which subjects were enrolled/randomized will be used in the listings. Age, sex at birth, race, and ethnicity will be included in the listings, as space permits.

The summaries of the efficacy data will be presented by treatment group. Unless otherwise specified, non-efficacy analyses will be descriptive and will be presented by treatment group and overall.

Unless stated otherwise, only the Phase 2 portion of the study will be summarized and listed.

2.1 Analysis Sets

The same definition of the analysis sets as stated in the SAP for final analysis is implemented in this follow-up analysis.

2.2 Subject Grouping

The same definition of the subject grouping as stated in the SAP for final analysis is implemented in this follow-up analysis.

2.3 Stratification Factors

The same stratification factors as stated in the SAP for final analysis is used in this follow-up analysis.

2.4 Examination of Subject Subgroups

The same subject subgroups as stated in the SAP for final analysis is considered in this follow-up analysis.

2.5 Multiple Comparisons

As this follow-up analysis is not included for type I error control, there is no adjustment made for multiple comparison.

2.6 Missing Data and Outliers

The missing data and outliers will be handled with the same method as stated in the SAP for final analysis.

2.7 Analysis Visit Windows

Study day and analysis visit window will be defined in the same way as stated in the SAP for final analysis.

3 SUBJECT DISPOSITION

3.1 Subject Enrollment and Disposition

A summary of subject disposition will be provided by treatment group. Percentages will be based on the ITT Analysis Set. The number of subjects will be provided for the same set of categories as in the final analysis.

In addition, summary of subject disposition will be provided in subgroups defined by Tumor IHC staining score of 2+ or 3+ in >=10% vs <10% of cells.

3.2 Extent of Exposure and Adherence

Descriptive statistics of extent of exposure will be presented by treatment group for each component of treatment (FPA144/placebo, Oxaliplatin, Leucovorin, 5FU) separately:

- Duration of exposure (weeks)
- Cumulative exposure by week
- Number of infusions
- Dose adherence
- Dose intensity

Total duration of exposure to study drug (in weeks) will be defined as (last available dosing date – first dosing date + 14)/7, regardless of any temporary interruptions in study drug administration.

Dose adherence is defined as 100* (Total study drug administered in mg/ Total study drug prescribed in mg during exposure to study drug). Total study drug prescribed is based on the data of "Prescribed Dose" collected on the study drug administration pages in EDC where per-protocol dose change is accounted.

Dose intensity is defined as 100*(Total study drug administered in adjusted unit [mg/kg or mg/m^2] / Total study drug expected to be administered during exposure to study drug in adjusted unit [mg/kg or mg/m^2]). Total study drug expected to be administered is based on the planned dose level specified in protocol, i.e., 15 mg/kg Q2W for FPA144/placebo (an additional of 7.5mg/kg on Cycle 1 Day 8), 85 mg/m^2 Q2W for oxaliplatin, 400 mg/m^2 Q2W for leucovorin, and 2800 mg/m^2 Q2W for 5-FU.

The number and percentage of subjects who have dose reduction, dose delay or interruption, and infusion interruption will be summarized with reasons.

Summaries of exposure will be performed with the Safety Analysis Set. A by-subject listing of

study drug administration will be provided.

In addition, summary of exposure will be provided in subgroups defined by Tumor IHC staining score of 2+ or 3+ in >=10% vs <10% of cells.

4 DEMOGRAPHICS AND BASELINE CHARACTERISTICS

4.1 Demographics

Demographic data will be summarized in the same way as in the final analysis.

In addition, summary of demographic will be provided in subgroups defined by Tumor IHC staining score of 2+ or 3+ in >=10% vs <10% of cells.

4.2 Other Baseline Disease Characteristics

Baseline will be summarized in the same way as in the final analysis.

4.3 Medical History

A summary of disease-specific medical history will be provided in the same way as in the final analysis as part of the disease-specific baseline characteristics.

Partial dates in deriving the time since diagnosis will be imputed in the same way as in the final analysis.

In addition, summary of disease-specific medical history will be provided in subgroups defined by Tumor IHC staining score of 2+ or 3+ in >=10% vs <10% of cells.

General medical history data will be coded according to Medical Dictionary for Regulatory Activities (MedDRA) Version 20.1.

4.4 Prior and Concomitant Medications

Prior and concomitant medications will be coded using the World Health Organization Drug Dictionary (WHODD) and classified according to Anatomical Therapeutic Chemical classification (ATC) codes levels 2 (therapeutic sublevel) and 4 (chemical sublevel).

Prior and concomitant medication will be defined and analyzed in the same way as in the final analysis.

4.5 Prior Anticancer Therapy

The prior anticancer therapy will be summarized in the same way as in the final analysis. The same imputation rule will be implemented for the partial dose date.

5 EFFICACY ANALYSES

Same as in the final analysis, the ITT Analysis Set will be used as the analysis set for the efficacy analysis.

5.1 Efficacy Endpoints

Efficacy analysis will only be performed for OS in this follow-up analysis.

The same definition of OS and partial date imputation rule as in the final analysis will be implemented.

5.2 Analysis of Efficacy Endpoint

Kaplan-Meier method will be used to provide the median, Q1, Q3, the proportion of subjects who are alive at 6, 12, and 15 months from randomization along with corresponding 95% CIs. In addition, the HR between the 2 treatment groups and its 95% CI will be estimated using the Cox proportional hazards regression model with treatment group as the only main effect and stratified by the stratification factors at randomization.

No hypothesis testing between treatment groups will be performed for OS.

To assess the robustness of the primary OS results, the analysis will be performed based on the Efficacy-Evaluable Analysis Set with the same analysis methods.

OS will also be analyzed based on the subgroups defined in Section 2.4. Same analysis as specified for the primary analysis will be performed, except that the hazard ratio estimated by the Cox proportional hazard model will not be adjusted by randomization stratification factors.

5.3 Other Efficacy Measures

The subsequent anticancer therapy after subjects discontinued study treatments are be collected and coded using the World Health Organization Drug Dictionary (WHODD) and classified according to Anatomical Therapeutic Chemical classification (ATC) codes levels 2 (therapeutic sublevel) and 4 (chemical sublevel).

A summary of subsequent anticancer therapy will be provided. In addition to the overall summary, the subsequent anticancer therapy will also be summarized by lines of therapy and by subgroups defined by Tumor IHC staining score of 2+ or 3+ in >=10% vs <10% of cells.

6 SAFETY ANALYSES

Same as in the final analysis, the Safety Analysis Set will be used as the analysis set for the safety analysis.

No formal comparisons of safety endpoints will be conducted.

6.1 Adverse Events and Deaths

6.1.1 Adverse Event Dictionary

All AEs will be coded to system organ class (SOC) and preferred term (PT) using MedDRA Version 20.1.

6.1.2 Adverse Event Severity

Adverse events are graded for severity by the investigators using the National Cancer Institute Common Toxicity Criteria for Adverse Events (NCI-CTCAE) Version 5. The severity grade of events for which the investigator did not record severity will be categorized as “missing” for tabular summaries and data listings.

6.1.3 Relationship of Adverse Event to Study Drug

A treatment related AE is an AE noted as related to FPA144/placebo, Oxaliplatin, Leucovorin, or 5FU by the investigator. Events for which the investigator did not record relationship to study drug will be considered related to study drug for summary purposes. However, by-subject data listings will show the relationship as missing.

6.1.4 Serious Adverse Events

Serious adverse events (SAEs) will be identified and captured as SAEs if the AEs met the definitions of SAEs that were specified in the study protocol.

6.1.5 Treatment-Emergent Adverse Events

The same definition of treatment-emergent AE (TEAE) and data handling rules as in the final analysis is used in this follow-up analysis. The same criteria will be implemented to define TEAE while the partial or completely missing date occurs.

6.1.6 Summary of Adverse Events and Deaths

The same summaries as in the final analysis will be provided for TEAE. In addition, the following summaries will be provided in subgroups defined by Tumor IHC staining score of 2+ or 3+ in >=10% vs <10% of cells:

- TEAE Overall summary

- TEAE by PT
- TEAE with Grade 3 or above
- TE SAE
- TEAE leading to FPA144/Placebo treatment discontinuation
- TEAE leading to death

A summary by severity with the following grouped PTs will also be provided:

- ALT, AST, and transaminases increased
- Mucosal inflammation and stomatitis

6.1.7 Adverse Events of Special Interest

An AE of special interest (AESI) (serious or non-serious) is an event of medical concern considered potentially associated to the investigational product or disease under study, for which ongoing monitoring and rapid communication by the investigator to the Sponsor is necessary. The following events are considered events of special interest in this study:

- Ocular events associated with symptomatic corneal involvement and symptomatic and asymptomatic retinal involvement: these events are defined as those AEs in the Standardised MedDRA Queries (SMQs) (Broad) of Cornea Disorders and Retinal Disorders. Summaries will be provided with SMQs of both Cornea Disorders and Retinal Disorders and SMQ of Cornea Disorders alone with be provided separately.
- Events of hypersensitivity: these events are defined as those AE in the SMQ (Broad) of Hypersensitivity

Treatment-emergent AESIs of ocular events are defined as those AEs present not prior to the start date of study drug or was worsened during treatment and 100 days after permanent discontinuation of study drug, as collected from CRF. The treatment-emergent definition for general AEs is still applied for AESIs of hypersensitivity events.

Treatment-emergent AESIs will be summarized by PT and maximum CTCAE Grade. The following summaries will be provided in subgroups defined by Tumor IHC staining score of 2+ or 3+ in >=10% vs <10% of cells:

- TE AESI with Cornea Disorders (SMQ) and Retinal Disorders (SMQ)
- TE AESI with Cornea Disorders (SMQ)
- TE AESI leading to drug discontinuation of FPA144/Placebo with Cornea Disorders

(SMQ) and Retinal Disorders (SMQ)

- TE AESI leading to drug discontinuation of FPA144/Placebo with Cornea Disorders (SMQ)

The first onset of ocular events will be summarized over time by the following categories:

- First dose of FPA144/Placebo to Week 8
- Week 9 to Week 16
- Week 17 to Week 24
- Week 25 onward

Summaries of the time to onset and resolution of ocular events will be provided for TE AESIs and TE AESIs leading to FPA144/Placebo discontinuation. The number and percentage of subjects who experienced ocular event will be summarized with resolution categories. Time to first onset and resolution of ocular events will be analyzed by crude estimate and Kaplan-Meier method. The time to onset and time to resolution are defined as followed:

- Time to onset (days) = AE start date of the first occurrence or maximum follow-up date – first dose date + 1
- Time to resolution (days) = AE end date or maximum follow-up date – AE start date + 1

The maximum follow-up date is the minimum of last study participation date, death date, withdraw consent date, and data cutoff date. The time to resolution will only be calculated among subjects with ocular event onset. In the absence of an ocular event, the censoring date for time to onset will be the earliest from the following dates:

- Last dose date of FPA144/placebo + 100 days
- Death date
- Withdraw consent date
- Analysis cut-off date

6.2 Clinical Laboratory Evaluations

CTCAE v5 grading will be implemented for laboratory grading. The same criteria as in the final analysis will be applied for inclusion of laboratory data in the summaries. The treatment-emergent laboratory abnormalities are defined in the same way as in the final analysis.

The following summaries will be provided by lab test and treatment group:

- Grade 3 or 4 laboratory abnormalities
- Shift by baseline grade and maximum grade

6.3 Body Weight and Vital Signs

The same summary of body weight and vital signs as in the final analysis will provided.

6.4 Electrocardiograms

A listing of subjects with abnormal ECG findings will be provided.

6.5 Other Safety Measures

Ocular examinations including fluorescein staining score, ocular symptoms, fundoscopy, ocular coherence tomography, visual acuity, intraocular pressure, slit lamp biomicroscopy, and confrontation visual field exams were performed at baseline and postbaseline in the study. The number of subjects shifted from Not Clinically Significant at baseline to Clinically Significant at any postbaseline assessment will be summarized by examination and treatment group.

Shift in fluorescein staining score from baseline to most extreme postbaseline grade will be presented.

In addition, analysis of fluorescein staining score and visual acuity will be performed. Fluorescein staining score is collected in EDC per assessment schedule specified in the protocol. Subjects with at least one measurement of fluorescein staining score will be included in the analysis.

The data of visual acuity measures is not included in EDC initially, but collected retrospectively by soliciting the available visual acuity measures (with or without correction, whichever available) as part of the protocol specified eye examination. The measurements of visual acuity are converted to log-MAR scale for analysis. As bias may be introduced in the analysis due to correction, only subjects with corrected visual acuity in each assessment, or with visual acuity better than 20/50 (Snellen scale) in each assessment regardless of correction, or with change from baseline less than 0.1 (log-Mar scale) regardless of correction in any postbaseline assessment, or subjects with any postbaseline corrected visual acuity of 20/50 (Snellen scale) or worse will be included in the analysis. The analysis will be provided for both unilateral, i.e., taking the best visual acuity measurement among the two eyes in each assessment, and bilateral, i.e., taking the worst visual acuity measurement among the two eyes in each assessment, respectively.

In the analysis of fluorescein staining score and visual acuity, summaries of the time to worsening onset and resolution will be provided, respectively. The number and percentage of subjects who experienced worsening defined by fluorescein staining score and visual acuity deterioration will be summarized with resolution categories. Time to first onset and resolution of

worsening will be analyzed by crude estimate and Kaplan-Meier method. The time to worsening onset and time to resolution are defined in a similar way as for AESI provided in Section 6.1.7, while visit date is used instead of AE start or end date in the derivation.

A by-subject listing of the ocular examinations will be provided.

7 REFERENCES

Koch, G., Carr, G., Amara, I., Stokes, M., & Uryniak, T. (1989). *Categorical Data Analysis.* Chapter 13 in Berry, D.A. (ed.). Statistical Methodology in the Pharmaceutical Sciences. New York: Karcel Dekker, Inc.

8 SOFTWARE

SAS® Software Version 9.4. SAS Institute Inc., Cary, NC, USA
